# Benzyne-Promoted, 1,2-*cis*-Selective *O*-Glycosylation with Benzylchalcogenoglycoside Donors

**DOI:** 10.1021/acs.orglett.3c03502

**Published:** 2023-11-16

**Authors:** Tiffany Duong, Erik Alvarez Valenzuela, Justin R. Ragains

**Affiliations:** Department of Chemistry, Louisiana State University, Baton Rouge, Louisiana 70806, United States

## Abstract

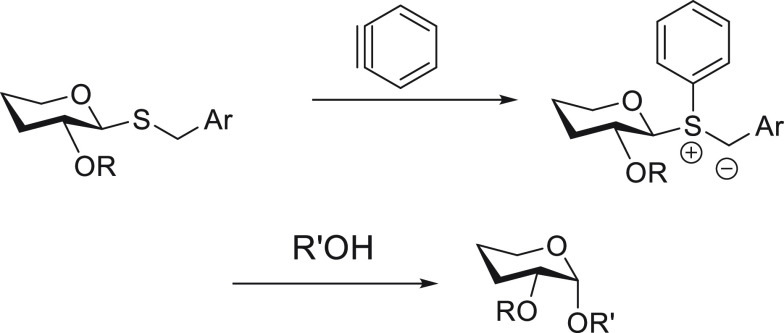

Here, we show that the reaction of benzylchalcogenoglycosides with benzyne in the presence of alcohols results in highly 1,2-*cis*-selective *O*-glycosylation in a solvent-dependent manner. Thioglycosides, selenoglycosides, and alcohols with a range of nucleophilicities lead to a productive reaction, and unusual protecting groups, auxiliary groups, and additives are avoided.

Synthetic oligosaccharides and other *O*-glycosides are important molecules for the development of, *inter alia*, glycoconjugate vaccines,^1,2^ glycan arrays,^3^ and drugs.^4^ While substantial progress has been made in the area of chemoenzymatic synthesis of glycosides,^5^ chemical synthesis remains a critical area of development in this field. Of the many longstanding problems in the area of *O*-glycoside synthesis, (1) the development of user-friendly protocols that enable iterative synthesis of oligosaccharides with an operational simplicity rivaling that of solid-phase peptide synthesis^6^ and (2) the development of highly 1,2-*cis*-selective *O*-glycosylations^7,8^ are particularly important for the advancement of the field. The latter challenge has been addressed frequently with the use of specific protecting group patterns, designer participating groups, and indirect multistep processes. Approaches that avoid these strategies are coveted.

Thioglycoside donors, which typically bear either an alkylthio- or arylthio- leaving group at the anomeric position, are particularly amenable to multistep synthesis of *O*-glycosides.^9,10^ They are stable to most conditions used in multistep *O*-glycoside synthesis, are easily synthesized, and have tunable reactivity.^10^ Because of their stability, however, thioglycoside activation typically requires the implementation of highly reactive electrophiles or reagent cocktails (e.g., NIS/HOTf, DMTST, MeOTf, PhSOTf). We and others have been successful in the development of alternative photochemical approaches to thio/selenoglycoside activation^11−19^ while others have enjoyed considerable success in the area of electrochemical activation.^20^ However, the *in situ* generation of highly reactive and short-lived electrophiles that then react with thio/selenoglycosides is an underexplored area that interests us.

To address the third topic, we imagined the mechanistic scenario outlined in Scheme 1. Fluoride-promoted generation of benzyne (**2**) from 2-trimethylsilylphenyl trifluoromethanesulfonate (Kobayashi’s reagent)^21^ would precede reaction with thioglycoside (**1**) sulfur to generate betaine **3**.^22−24^

**Scheme 1:**
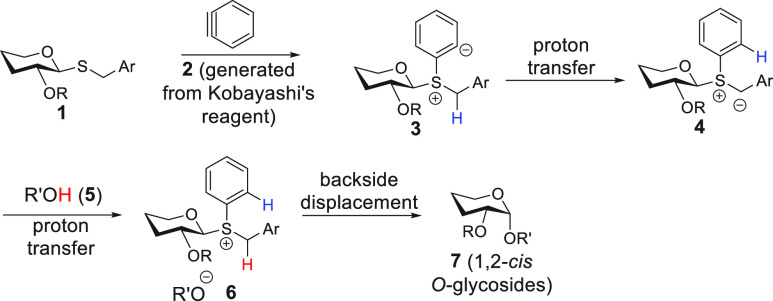
Benzyne-Mediated, 1,2-*cis*-Selective *O*-Glycosylation

Proton transfer from benzylic methylene to generate sulfur ylide **4** would be facile. Subsequent diffusion of alcohol acceptor into the **4**-containing solvent cage would then need to pre-empt unwanted Stevens rearrangement of ylide to *C*-glycoside.^25,26^ Proton transfer from alcohol to **4** would then lead to ion pair **6** and *O*-glycoside formation. Wan and co-workers have provided an elegant approach to glycosyl sulfur ylide generation en route to *O*-glycosylation using Rh catalysis and diazoester substrates,^26^ and Turnbull and co-workers have reported on the limited formation of acetyl *O*-glycosides using benzyne generated under oxidative conditions from 1-aminobenzotriazole.^27^*Nevertheless, this is an underexplored mechanistic approach that provides the tantalizing possibility of high 1,2-cis-selectivity according to the conversion of****6****to****7****by exclusive backside attack.* Herein, we report on our initial results in this area where we have observed stereospecific processes based on the inversion of initial chalcogenoglycoside stereochemistry. Contrary to previous approaches involving implementation of specialized protecting groups^28,29^ or auxiliaries,^30,31^ we demonstrate user-friendly and highly 1,2-*cis*-selective *O*-glycoside formation at room temperature. This protocol is contingent on commercially available reagents, judiciously chosen solvents, and benzylthioglycoside or benzylselenoglycoside donors. Further, donors are synthesized with a facility that is comparable to those of the more commonly used arylthioglycosides.

In our initial study (Scheme 2), we used perbenzylated donors **8a**–**8d** (1.3 equiv) and reacted them with C6 d-glucose acceptor **9** (1 equiv). We chose Kobayashi’s reagent (**10**, 2 equiv) as a benzyne precursor and screened, *inter alia*, various fluoride sources (3.9 equiv) and solvents at room temperature (18–22 °C) for reaction times of 48 h. Benzyl thioglycoside **8a** in the presence of KF/18-C-6 in 1,4-dioxane afforded 20% of disaccharide **11** with a 1,2-*cis*/1,2-*trans* ratio of 5:1 (entry 1) while methyl *tert*-butyl ether (MTBE) as solvent afforded disaccharide **11** in an improved 37% yield with a 1,2-*cis*/1,2-*trans* ratio of 5:1 (entry 2). Phenylthioglycoside **8b** under the entry 1 conditions provided only traces of **11** (detectable by TLC, entry 3). Switching to MeCN as solvent (entry 4) necessitated CsF as an activator for best results and afforded **11** in 22% but, perhaps not surprisingly given the 1,2-*trans* selectivity often promoted by nitrile solvents,^32^ with a reversal of selectivity. Use of 1,2-dichloroethane (DCE, entry 5) provided results that were comparable to those of entry 2 while CH_2_Cl_2_ provided inferior results (entry 6) and DMF and THF provided no detectable product **11** (data not shown).

**Scheme 2:**
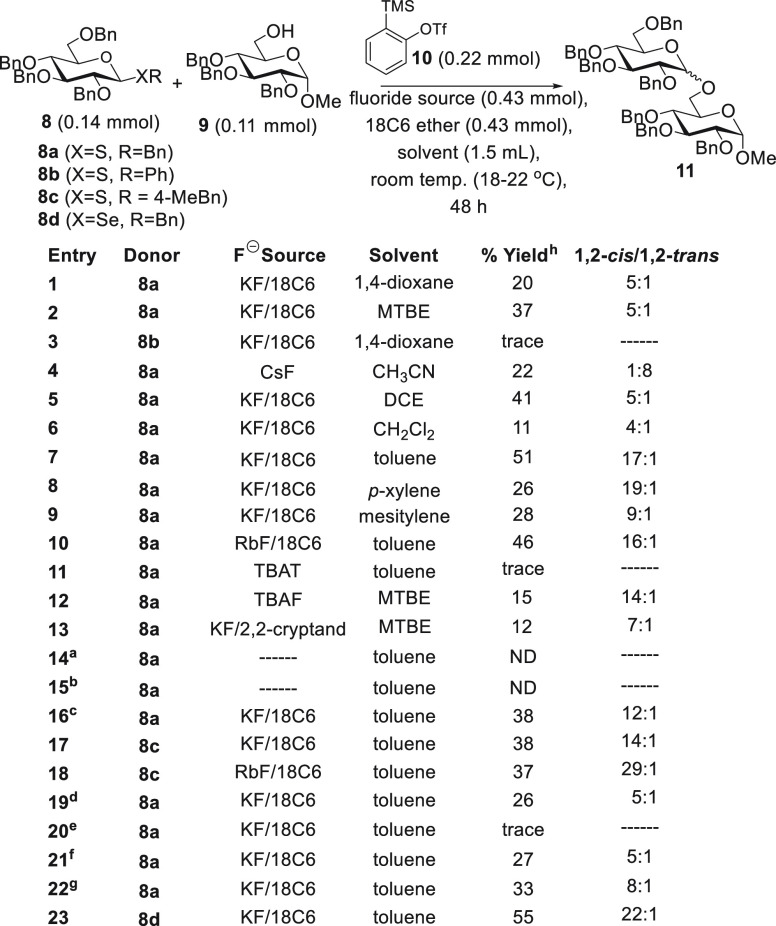
Optimization Study Cs_2_CO_3_/18-C-6 instead of a fluoride source. K_2_CO_3_/18-C-6 instead of a fluoride source. Kobayashi’s reagent (**10**), KF, 18-C-6 in 10 equiv. (1.1 mmol). Experiment conducted at −65 to −50 °C. Experiment conducted at 60 °C. 3 equiv of donor **8a** (0.33 mmol). 0.11 mmol of donor **8a** and 0.22 mmol of acceptor **9**. Isolated yields, ND (none of the desired product **11** was detected in the reaction mixture); “trace” indicates that **11** was detected by TLC but not abundant enough to isolate with chromatography.

Our biggest breakthrough came with the screening of toluene. In the event, the use of toluene resulted in a 51% yield of **11** with a 17:1 selectivity in favor of 1,2-*cis* (entry 7). The solvents *p*-xylene and mesitylene provided comparable selectivities but lower yields (entries 8 and 9). Switching to RbF as the fluoride source provided comparable yields and little improvement in selectivity over entry 9 conditions (entry 10). At this stage, we were concerned about sluggish reactions (reaction times of 48 h were necessary while longer reaction times provided no benefit) during the 48 h period of reaction. We thus screened alternative fluoride sources including tetrabutylammonium difluorotrimethylsilicate (TBAT, entry 11), TBAF (entry 12), and KF/2,2-cryptand (entry 13), the latter two of which gave better results using MTBE as solvent. Implementation of carbonate bases instead of fluoride sources for the activation of Kobayashi’s reagent per the observations of others^33^ resulted in no formation of **11** (entries 14 and 15). Likewise, using excesses of KF/18-C-6 and Kobayashi’s reagent (e.g., 10 equiv, entry 16) surprisingly provided no improvement over entry 7. Presently, we are aware of two processes that the as-formed benzyne undergoes: reaction with sulfur (as is evident with the isolation of glycosylation products) and reaction with alcohol hydroxyl (*vide infra*). Dimerization or oligomerization of benzyne is not readily evident given the complexity of the aromatic region in ^1^H NMR spectra of crude reaction mixtures.

Continuing studies involved synthesis and screening of electron-donating-group-containing *p*-methylbenzylthioglycoside **8c** under the entry 7 conditions (entry 17). This resulted in a 38% yield and 14:1 1,2-*cis*/1,2-*trans* selectivity, while use of RbF/18-C-6 as the fluoride source under otherwise identical conditions provided comparable yields but improved selectivity (entry 18). We also synthesized a trifluoromethylated analogue of **8c**. Purification of this compound proved to be difficult, and results were inferior to those for the entry 7 conditions (data not shown). We also considered temperature (−65 to −50 and 60 °C, entries 19 and 20), use of larger excesses of donor **8a** (entry 21), excesses of acceptor **9** (entry 22), and dilution of reaction mixtures (data not shown); however, all of these attempts provided inferior results to those of entry 7. Likewise, preparation of reaction mixtures with the most rigorous possible exclusion of water that we could achieve (i.e., in a glovebox and with activated 4 Å molecular sieves) provided no improvement. Finally (entry 23), we attempted the entry 7 conditions using benzylselenoglycoside **8d** and observed an improved yield (55%) and selectivity (22:1) over those observed with thioglycoside **8a**. We observed improved performance of **8d** over **8a** with respect to yield and selectivity in a subsequent substrate scope study (*vide infra*). Results in Scheme 2 and Table S1 in the Supporting Information provide a sampling of the efforts made toward solving this problem.

Observation of the unreacted donor and acceptor and alternative fates of the as-formed benzyne characterized the outcome of our experiments. We were unable to produce any evidence for unwanted Stevens rearrangement product^25,26^ through either mass spectrometry or NMR analysis. We did observe small amounts of phenyl ether **12** in reaction mixtures and especially when employing an excess of acceptor **9** (Scheme 3). We were also able to scale the protocol up to 1 mmol under the modified conditions (Scheme 3).

**Scheme 3:**
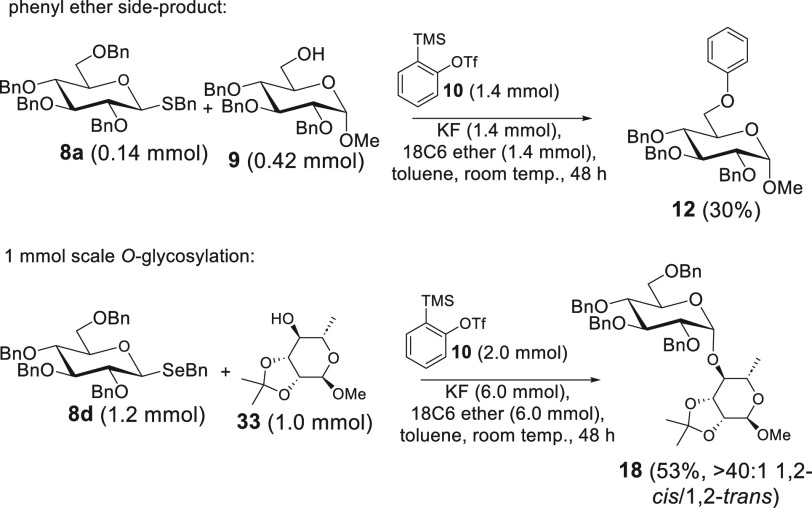
Additional Observations

We elected to perform a substrate scope study, and the results proved to be illuminating. These results are shown in Scheme 4. Reaction of **8a/d** with glucose diacetonide resulted in the highest yields of *O*-glycosides (**13**) observed in this study and no observed 1,2-*trans* products (designated here as “>40:1”, entry 1). d-Galactose diacetonide (entry 2) and thiophenyl glucoside (demonstrating the orthogonality of phenylthio glycosides, entry 3), both C6 acceptors, gave similar results compared to entry 1.

**Scheme 4:**
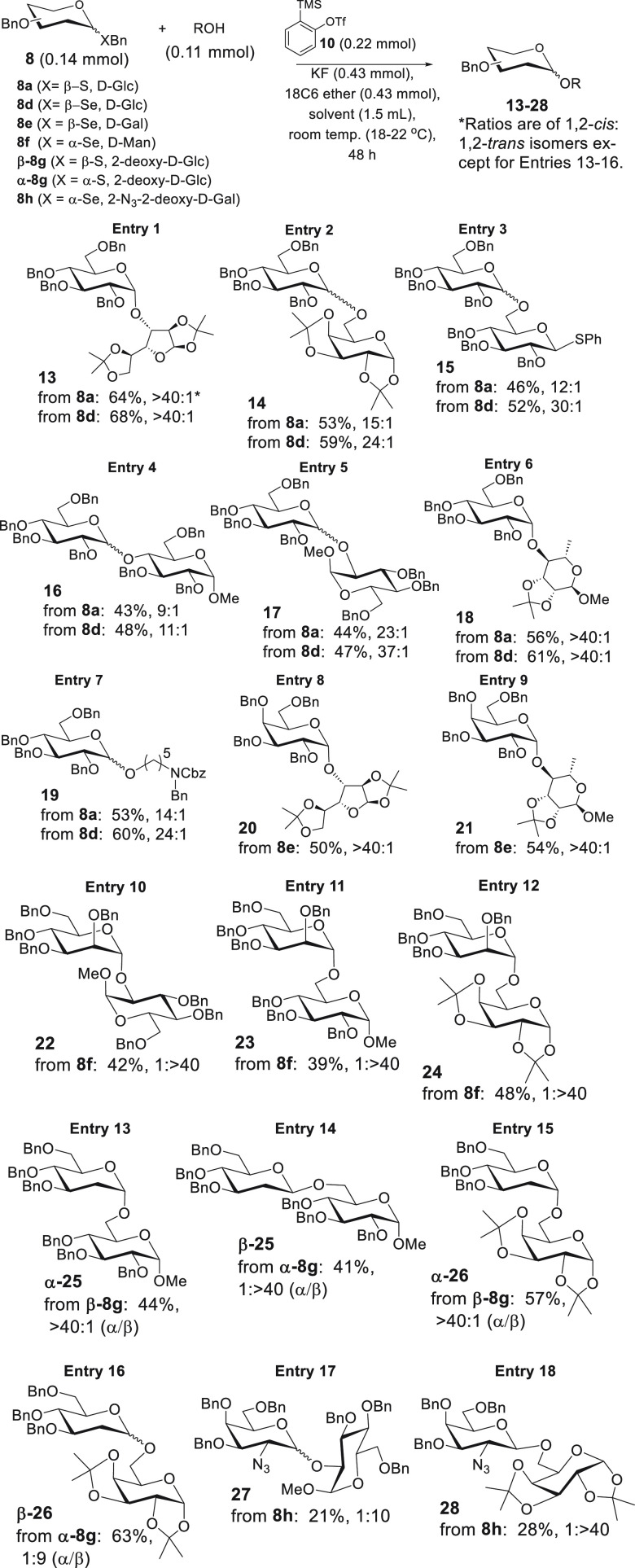
Substrate Scope Study

We then elected to screen unreactive C4 and C2 acceptors^34,35^ (entries 4 and 5) and were surprised by results that were quite similar, from the standpoint of isolated yield, to those obtained using the primary acceptors in entries 2 and 3. *These results corroborate the notion, implied earlier, that the selectivity of benzyne and recovery of unreacted substrates are currently the most significant impediments to the development of benzyne-mediated O-glycosylation.* Reactivity of acceptors does not appear to be a factor. In our opinion, the mere fact that we can obtain even modest yields in entries 4 and 5 shows the promise of this approach. l-Rhamnose acetonide (entry 6) gave exceptionally high selectivity (>40:1). Finally, we performed *O*-glycosylation with the “linker” acceptor *N-*benzyl-*N-*carbobenyloxy-5-aminopentan-1-ol which is a notoriously poor acceptor for the development of 1,2-*cis* processes due to its high reactivity relative to “sugar” acceptors (entry 7).^29,34,35^ In this event, glycosylation of this acceptor occurred with yields comparable to those of other entries and with selectivities of 14:1 and 24:1 1,2-*cis*/1,2-*trans* when using donors **8a** and **8d**, respectively.

With the success of the aforementioned *O*-glucoside syntheses, we decided to study other systems (Scheme 4). Starting with d-galactose donor **8e** (entries 8, 9), we generated products with 1,2-*cis* as the only observed isomer. Subsequently, we synthesized α-selenobenzyl mannoside **8f** hypothesizing that inversion would result in synthetically challenging 1,2-*cis*-mannosides (entries 10–12). Unfortunately, all reactions proved highly selective for the 1,2-*trans* products, demonstrating that steric bias leads to alternative mechanistic pathways. We next synthesized 2-deoxy-d-glucose donors β-**8g** and α-**8g** which could further serve as probes for the stereoinversion hypothesis in Scheme 1. *To our delight, β-***8g***led to high proportions of α-2-deoxyglycosides (entries 13,15) while α-***8g***led to β-2-deoxyglycosides (entries 14, 16).* To further corroborate these observations, we synthesized α-selenobenzyl 2-azido-2-deoxy-d-galactoside **8h** which also furnished high proportions of β-glycosidic products (entries 17, 18). Taken together, these results bolster the stereoinversion hypothesis in Scheme 1. Nevertheless, sterically biasing substituents, as in the case of the mannosides, may lead to alternative pathways.

In conclusion, we have developed a mechanistically novel approach to *O*-glycosylation in which benzyne serves as a critical intermediate in thio/selenoglycoside activation. This user-friendly protocol has proven to be stereospecific as well as highly 1,2-*cis*-selective in many cases without resorting to special protecting groups, auxiliaries, additives, or expensive reagents. Instead, high selectivity is dependent on solvent polarity, which also corroborates the mechanistic hypothesis in Scheme 1. Ion pair **6** is expected to react exclusively by backside attack without leakage to more S_N_1-like pathways at low dielectric constants provided that there is no strong steric bias.

This work represents our initial exploration of this area. Reaction of Kobayashi’s reagent with fluoride is certainly not the only approach to benzyne generation. Other methods and approaches to this problem are feasible, and many of these will be explored during the course of the ongoing work.

## Data Availability

The data underlying this study are available in the published article and its Supporting Information.

## Supporting Information Available

Experimental procedures, characterization data, ^1^H and ^13^C spectra (PDF)
