# Supplementary material for: Benzyne-Promoted, 1,2-cis-Selective O-Glycosylation with Benzylchalcogenoglycoside Donors
Source: Org Lett. 2023 Nov 16;25(47):8526–9. doi: 10.1021/acs.orglett.3c03502 (PMC10696609; doi:10.1021/acs.orglett.3c03502)

Supplemental Information

Benzyne-Promoted, 1,2-*cis*-Selective *O*-Glycosylation with Benzylchalcogenoglycoside Donors

Tiffany Duong, Erik Alvarez Valenzuela, and Justin Ragains

Department of Chemistry

Louisiana State University

232 Choppin Hall

Baton Rouge, LA 70803

Table of Contents

General Information……………………………………………………………………………S3

Estimation of Anomeric Ratios…………………………………………………………………S4

Experimental Procedures and Characterization of Glycosyl Donors…………………S5-S23

Glycosylation Procedures……………………………………………………………………S23-S24

Optimization Study……………………………………………………………………………S24-S26

Information on Alcohol Acceptors…………………………………………………………S26

Representative Procedures for the Substrate Scope Study…………………………..S26-S36

Experimental Procedure and Characterization of Isolated Side Product…………...S36-S37

Determination of Configuration at the Anomeric Center……………………………..S37

References……………………………………………………………………………………...S37-S39

NMR (^{1}H, ^{13}C) Spectra of Glycosyl Donors and Isolated Side Product………………S40-S74

^{1}H NMR Estimation of Anomeric Ratios – Purified Products

- **^{1}H NMR of Purified Products – Optimization Study……………..…..…….……S75-S93**
- **^{1}H NMR of Purified Products – Substrate Scope Study…...……………..…..S94-S120**

^{1}H NMR Estimation of Anomeric Ratios – Crude Reaction Mixtures

- **^{1}H NMR of Crude Reaction Mixtures – Optimization Study………………...S121-S139**
- **^{1}H NMR of Crude Reaction Mixtures – Substrate Scope Study……………S140-S165**

General Information

All reactions were performed under N_2 atmosphere via vacuum purge backfill done three times. Dried solvents (CH_2Cl, CH_3CN, THF, and DMF) were used directly from a Pure-Solv 400-5 solvent purification system. Anhydrous 1,4-dioxane solvent was obtained through distillation from sodium-benzophenone ketyl. All remaining solvents were purchased from commercial sources and dried over 3 Å molecular sieves (J.T. Baker) for a minimum of 72 hours prior to use. Reagents were purchased from commercial sources (Alfa Aesar, Acros Organics, Matrix Scientific, Sigma Aldrich, TCI Chemicals) and used without further purification. Column chromatography was performed using 60 Å silica gel (SiliCycle). Preparative TLC was performed on glass-backed 60 Å silica gel with F254 indicator (SiliCycle). Analytical TLC was performed using aluminum backed 60 Å silica gel with F254 indicator (MilliporeSigma). Compound visualization on TLC was performed using a hand-held UV lamp (254 nm) and with *p*-anisaldehyde staining. ^{1}H NMR and ^{13}C NMR were performed using a Bruker AV-400 or Bruker AV-500 NMR spectrometer. HRMS was performed using an Agilent 6210 electrospray time-of-flight mass spectrometer. Optical rotation values were obtained using a JASCO P-2000 instrument. Deuterated solvents were obtained from Cambridge Isotope Labs.

Estimation of Anomeric Ratios

^{1}H NMR of anomeric mixtures in both the crude and purified samples were obtained using 16 scans with relaxation delays set to 20 seconds. 1,2-*cis* to 1,2-*trans* ratios of glycosylation product mixtures were determined using ^{1}H NMR integration of distinct signals from both the 1,2-*cis* and 1,2-*trans* products.

In the case of glycosylation reactions using acceptor **9** bearing a free hydroxyl at C6 and a methyl aglycone at the reducing end, anomeric ratios were determined according to the following set of commands using the GSD algorithm (deconvolution) in MestReNova:

1. Processing > Phase Correction > Automatic
2. Processing > Baseline Correction > Full Auto (Bernstein Polynomials)
3. Analysis > Peak Picking > Options > Methods = GSD, Refinement Level = 5 fitting, Optimized for Peaks = average > OK
4. Analysis > Integration > Options > Calculation Method = Sum, Source = Autodetect, Algorithm = Peak Picking, Minimum Area = 3.00% > OK
5. Analysis > Peak Picking > Automatic
6. View > Tables > NMR – Peaks > OK

A GSD table containing all the peaks with their respective height, width and area is generated. For glycosylation products using acceptor **9**, we analyzed the reducing-end aglycone methyl signals for both the 1,2-*cis* and 1,2-*trans* products (located at 3.35 ppm for the 1,2-*cis* anomer and 3.32 ppm for the 1,2-*trans* anomer). Areas of these signals were used to determine anomeric ratios.

Experimental Procedures and Characterization of Glycosyl Donors

Preparation of Glucosyl Donor 8a

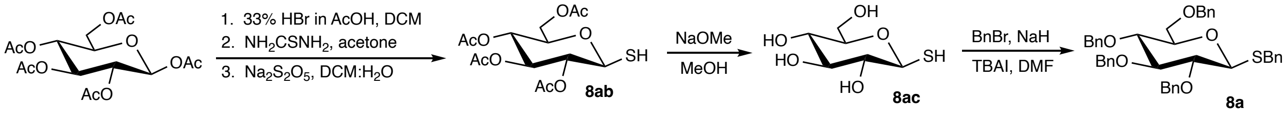

Synthesis of 8ab

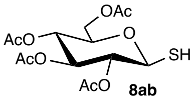

A solution of β-D-glucose pentaacetate (10.0 g, 25.6 mmol) in dichloromethane (100 mL) was cooled to 0 °C, capped with a septum and placed under nitrogen atmosphere. A canula was used to add the solution of 33% hydrogen bromide in acetic acid (25.0 mL, 217 mmol) dropwise over a period of 5 minutes. Upon completion of addition, the ice bath was removed. The reaction gradually warmed to room temperature and stirred for 3 hours. After this time, the reaction was cooled to 0 °C and slowly quenched using ice cold water (100 mL). The crude reaction mixture was transferred to a separatory funnel and the organic layer was collected and washed with saturated sodium bicarbonate (1 x 100 mL) and brine (1 x 100 mL). The organic layer was dried over sodium sulfate and concentrated to afford the glucosyl bromide intermediate as an orange oil (9.97 g, 24.2 mmol). Glucosyl bromide was dissolved in acetone (100 mL) and treated with thiourea (3.14 g, 41.3 mmol). The mixture was refluxed at 70 °C under nitrogen atmosphere for 3 hours. After this time, the reaction was cooled to room temperature and the crude mixture was vacuum filtered and washed with acetone to afford the glucosyl thiouronium salt as white solid (9.61 g, 19.7 mmol). Thiouronium salt was then dissolved in a mixture of dichloromethane and water (3:2, 200 mL) and treated with sodium metabisulfite (4.87 g, 25.6 mmol). The reaction mixture was heated to 70 °C in an oil bath for 3 hours. After this time, the reaction was removed from the oil bath and cooled to room temperature. The crude mixture was transferred to a

separatory funnel and the aqueous and organic layers were separated. The aqueous layer was extracted with dichloromethane (1 x 100 mL) and the combined organic layers were dried over sodium sulfate and concentrated. The crude reaction mixture was purified via flash column chromatography using a solvent gradient of 10-45% ethyl acetate in hexanes to afford β-mercaptoglucoside intermediate **8ab** as a white solid (5.94 g, 64%). ^{1}H NMR spectra matched previously published spectra.[1]

Synthesis of 8ac

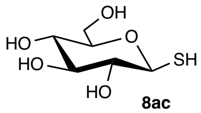

8ac

8ab (5.94 g, 16.3 mmol) was dissolved in methanol (50 mL) and treated with dropwise addition of 5M sodium methoxide (0.47 mL, 2.4 mmol) at room temperature. The reaction was monitored via thin layer chromatography and showed completion after stirring for 30 minutes. Upon completion the reaction was quenched using Dowex® 50WX8 200-400 mesh ion-exchange resin (Acros Organics) until the pH was neutral (indicated by pH strips). The mixture was vacuum filtered, and the mother liquor was collected and concentrated to afford intermediate **8ac** as a pale orange oil (3.10 g, 97%) requiring no further purification. ^{1}H NMR spectra matched previously published spectra.[2]

Synthesis of 8a

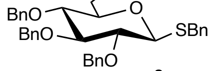

8a

8ac (3.10 g, 15.8 mmol) was dissolved in 100 mL of DMF. The reaction was cooled to 0 °C and allowed to stir for 20 minutes. After this time, TBAI (1.17 g, 3.17 mmol) was added and sodium hydride (60% suspension in mineral oil, 3.80 g, 95 mmol) was carefully added portion wise. The

reaction was capped with a septum and placed under nitrogen atmosphere. Lastly, benzyl bromide (18.8 mL, 158 mmol) was added dropwise using a syringe (over the period of 2 minutes). The reaction was allowed to gradually warm to room temperature and stirred overnight for 18 hours. After this time, the reaction was cooled to 0 °C and slowly quenched with ice cold water (300 mL). The crude reaction mixture was transferred to a separatory funnel and the aqueous layer was extracted with ethyl acetate (3 x 200 mL). The combined organic layers were dried over sodium sulfate and concentrated. The crude reaction mixture was purified via flash column chromatography using a solvent gradient of 5-15% ethyl acetate in hexanes to afford glucosyl donor **8a** as a pale-yellow solid (8.73 g, 85%). ^{1}H NMR spectra matched previously published spectra.[3]

Preparation of Glucosyl Donor 8b

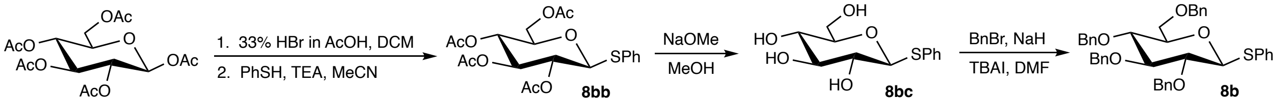

Synthesis of 8bb

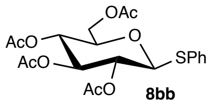

A solution of β-D-glucose pentaacetate (10.0 g, 25.6 mmol) in dichloromethane (100 mL) was cooled to 0 °C, capped with a septum and placed under nitrogen atmosphere. A canula was used to add the solution of 33% hydrogen bromide in acetic acid (25.0 mL, 217 mmol) dropwise over a period of 5 minutes. The ice bath was removed, and the reaction gradually warmed to room temperature and stirred for 3 hours. After this time, the reaction was cooled to 0 °C and slowly quenched using ice cold water (100 mL). The crude reaction mixture was transferred to a separatory funnel and the organic layer was collected and washed with sodium bicarbonate (1 x 100 mL) and brine (1 x 100 mL). The organic layer was dried over sodium sulfate and

concentrated to afford the glucosyl bromide intermediate as an orange oil (9.21 g, 22.4 mmol). The crude oil was dissolved in acetonitrile (50 mL) and cooled to 0 °C. Thiophenol (5.10 mL, 50.0 mmol) was added, followed by the dropwise addition of triethylamine (3.43 mL, 24.6 mmol) over a period of 1 minute. The reaction was allowed to gradually warm to room temperature after removal of the ice bath. Thin layer chromatography showed completion of the reaction after 1 hour. The reaction was quenched with water (100 mL) and transferred to a separatory funnel. The aqueous layer was extracted with dichloromethane (3 x 50 mL) and washed with brine (1 x 50 mL). The combined organic layers were dried over sodium sulfate and concentrated. The crude reaction mixture was purified via flash column chromatography using a solvent gradient of 10-35% ethyl acetate in hexanes to afford **8bb** as a white soild (7.75 g, 69%, 2 steps). ^{1}H NMR spectra matched previously published spectra.[4]

Synthesis of 8bc

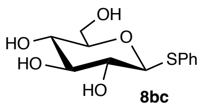

8bc

8bb (7.75 g, 17.6 mmol) was dissolved in methanol (50 mL) and treated with 5M sodium methoxide (0.50 mL, 2.5 mmol) at room temperature. The reaction was monitored via thin layer chromatography and showed completion after stirring for 20 minutes. Upon completion the reaction was quenched using Dowex® 50WX8 200-400 mesh ion-exchange resin (Acros Organics) until the pH was neutral (indicated by pH strips). The mixture was vacuum filtered, and the mother liquor was collected and concentrated to afford intermediate **8bc** as a pale orange oil (4.51 g, 94%) requiring no further purification. ^{1}H NMR spectra matched previously published spectra.[4]

Synthesis of 8b

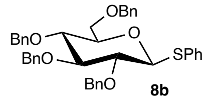

8b

Glucosyl intermediate **8bc** (4.51 g, 16.5 mmol) was dissolved in 100 mL of DMF. The reaction was cooled to 0 °C and allowed to stir for 20 minutes. After this time, TBAI (1.06 g, 2.87 mmol) was added and 60% sodium hydride in oil (3.17 g, 79.3 mmol) was carefully added portion wise. The reaction was capped with a septum and placed under nitrogen atmosphere. Lastly, benzyl bromide (15.7 mL, 132 mmol) was added dropwise using a syringe. The ice bath was then removed and the reaction was allowed to gradually warm to room temperature and stirred for 18 hours. After this time, the reaction was cooled to 0 °C and slowly quenched with ice cold water (300 mL). The crude reaction mixture was transferred to a separatory funnel and the organic layer was extracted using ethyl acetate (3 x 200 mL) and washed with ice cold water (2 x 300 mL). The combined organic layers were dried over sodium sulfate and concentrated. The crude reaction mixture was purified via flash column chromatography using a solvent gradient of 5-15% ethyl acetate in hexanes to afford glucosyl donor **8b** as a white solid (8.73 g, 84%). ^{1}H NMR spectra matched previously published spectra.[3]

Preparation of Glucosyl Donor 8c

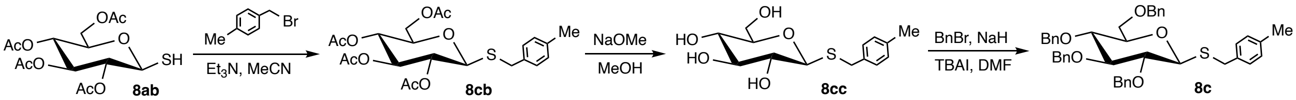

Synthesis of 8cb

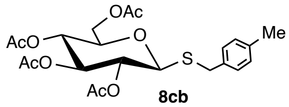

8cb

β-D-glucose pentaacetate (4.43 g, 12.2 mmol) was dissolved in acetonitrile (80 mL) and treated with 4-methylbenzyl bromide (3.20 g, 17.3 mmol) at room temperature. Triethylamine (1.70 mL,

12.2 mmol) was then added dropwise using a syringe. The reaction was monitored via thin layer chromatography and showed completion after stirring for 10 minutes. The reaction was quenched with water (50 mL) and the crude mixture was transferred to a separatory funnel. The aqueous layer was extracted with dichloromethane (2 x 100 mL). The combined organic layers were dried over sodium sulfate and concentrated. The crude reaction mixture was purified via flash column chromatography using a solvent gradient of 10-25% ethyl acetate in hexanes to afford **8cb** (4.97 g, 87%) as a colorless oil.

^{1}H NMR (500 MHz, CDCl$_3$) ***δ*** 7.19 (m, 2H), 7.13 (m, 2H), 5.15 (m, 1H), 5.09 (m, 2H), 4.28 (d, *J* = 10.0 Hz, 1H), 4.25 (d, *J* = 5.2 Hz, 1H) 4.16 (dd, *J* = 12.4 Hz, *J* = 2.4 Hz, 1H), 3.92 (d, *J* = 12.8 Hz, 1H) 3.81 (d, *J* = 12.9 Hz, 1H), 3.60 (m, 1H), 2.35 (s, 3H), 2.11 (s, 3H), 2.01 (m, 6H), 1.99 (s, 3H). **^{13}C NMR (125 MHz, CDCl$_3$)** ***δ*** 170.6, 170.2, 169.4, 169.4, 137.1, 133.6, 129.3, 129.0, 81.8, 75.8, 73.8, 69.8, 68.4, 62.2, 33.5, 21.1, 20.8, 20.7, 20.6, 20.6. **HRMS (ESI) m/z**: **[M + Na]+** calcd for $C_{22}H_{28}O_9SNa$ 491.13462; found 491.13453. **$[\alpha]_D^{25}$** = -96.8 (*c* 0.01, CH_2Cl_2). **IR (cm^{-1})** 1739, 1367, 1254, 1218, 1088, 1033, 914, 811, 752, 681, 621, 515, 469, 434.

Synthesis of 8cc

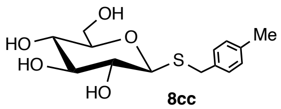

8cc

A solution of **8cb** (4.97 g, 10.6 mmol) in methanol (50 mL) was treated with the dropwise addition of 5M sodium methoxide (0.30 mL, 1.5 mmol) at room temperature. The reaction was monitored via thin layer chromatography and showed completion after stirring for 15 minutes. Upon completion the reaction was quenched using Dowex® 50WX8 200-400 mesh ion-exchange resin (Acros Organics) until the pH was neutral (indicated by pH strips). The mixture was vacuum filtered, and the mother liquor was collected and concentrated to afford intermediate **8cc** as a pale orange oil (2.91 g, 91%) requiring no further purification.

^{1}H NMR (500 MHz, CDCl$_3$) δ 7.27 (d, *J* = 10.0 Hz, 2H), 7.14 (d, *J* = 9.8 Hz, 2H), 4.19 (d, *J* = 11.5 Hz, 1H), 4.03 (d, *J* = 16.1 Hz, 1H), 3.92 (dd, *J* = 15.1 Hz, *J* = 2.8 Hz, 1H), 3.84 (d, *J* = 16.1 Hz, 1H), 3.71 (dd, *J* = 15.2 Hz, *J* = 7.6 Hz, 1H), 3.34-3.32 (m, 3H), 3.30-3.18 (m, 5H). 2.32 (s, 3H). **^{13}C NMR (125 MHz, CDCl$_3$) δ** 136.3, 134.9, 128.8, 128.6, 83.8, 80.6, 78.3, 72.9, 70.2, 61.6, 32.7, 19.7. **HRMS (ESI) m/z**: **[M + Na]+** calcd for $C_{14}H_{20}O_5SNa$ 323.09237; found 323.09171. **$[\alpha]_D^{25}$** = -21.5° (*c* 0.01, MeOH). **IR (cm^{-1})** 3338, 2919, 1513, 1276, 1019, 881, 817, 524.

Synthesis of 8c

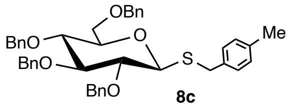

8c

Glucosyl intermediate **8cc** (2.91 g, 9.69 mmol) was dissolved in 100 mL of DMF. The reaction was cooled to 0 °C and allowed to stir for 20 minutes. After this time, TBAI (0.63 g, 1.7 mmol) was added and 60% sodium hydride in oil (1.86 g, 46.5 mmol) was carefully added portion wise. The reaction was capped with a septum and placed under nitrogen atmosphere. Lastly, benzyl bromide (9.22 mL, 77.6 mmol) was added using a syringe. The ice bath was removed, and the reaction was allowed to gradually warm to room temperature and stirred for 20 hours. After this time, the reaction was cooled to 0 °C and slowly quenched with ice cold water (300 mL). The crude reaction mixture was transferred to a separatory funnel and the aqueous layer was extracted using ethyl acetate (3 x 200 mL) and washed with ice cold water (2 x 300 mL). The combined organic layers were dried over sodium sulfate and concentrated. The crude reaction mixture was purified via flash column chromatography using a solvent gradient of 5-15% ethyl acetate in hexanes to afford glucosyl donor **8c** as a white solid (4.96 g, 77%).

^{1}H NMR (400 MHz, CDCl$_3$) δ 7.38-7.22 (m, 20 H), 7.17 (m, 2H), 7.08 (d, *J* = 6.1 Hz, 2H), 4.88-4.77 (m, 6H), 4.69-4.62 (m, 1H), 4.55 (t, *J* = 9.5 Hz, 2H), 4.26 (d, *J* = 9.7 Hz, 1H), 3.99 (d, *J* = 10.4 Hz, 1H), 3.86 (d, *J* = 10.3 Hz, 1H), 3.75 (m, 1H), 3.60 (m, 1H), 3.49 (m, 1H), 3.40 (m, 1H),

2.31 (s, 3H). **^{13}C NMR (125 MHz, CDCl$_3$) *δ*** 138.5, 138.2, 138.0, 137.9, 136.7, 134.4, 129.2, 129.1, 128.4, 128.4, 128.4, 128.3, 127.9, 127.8, 127.8, 127.8, 127.7, 127.6, 127.6, 86.6, 83.1, 81.7, 78.9, 78.0, 75.7, 75.3, 75.0, 73.4, 69.1, 34.0, 21.1. **HRMS (ESI) m/z**: **[M + Na]+** calcd for $C_{42}H_{44}O_5SNa$ 683.28017; found 683.27993. **$[\alpha]_D^{25}$** = -21.5° (*c* 0.01, CH_2Cl_2). **IR (cm^{-1})** 3028, 2868, 1495, 1452, 1358, 1080, 804, 738, 693, 464.

Preparation of Glucosyl Donor 8d

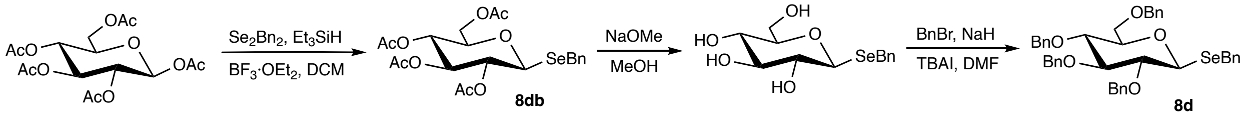

Synthesis of 8db

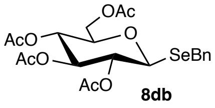

A solution of β-D-glucose pentaacetate (10.0 g, 25.6 mmol) in dichloromethane was treated with dibenzyl diselenide (6.12 g, 18.0 mmol) and boron trifluoride etherate (4.74 mL, 38.4 mmol) at room temperature. After stirring for 10 minutes, triethylsilane (6.13 mL, 38.4 mmol) was added and the reaction was allowed to stir at room temperature for 24 hours. The reaction was quenched with water (100 mL) and the crude mixture was transferred to a separatory funnel. The aqueous layer was extracted with dichloromethane (2 x 200 mL). The combined organic layers were dried over sodium sulfate and concentrated. The crude reaction mixture was purified via flash column chromatography using a solvent gradient of 10-20% ethyl acetate in hexanes to afford **8db** as a white solid (10.4 g, 81%). ^{1}H NMR spectra matched previously published spectra.[5]

Synthesis of 8dc

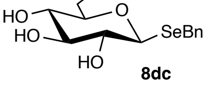

8db (10.4 g, 20.7 mmol) was dissolved in methanol (60 mL) and treated with sodium methoxide (0.60 mL, 3.0 mmol) at room temperature. The reaction was monitored via thin layer chromatography and showed completion after stirring for 15 minutes. Upon completion the reaction was quenched using Dowex® 50WX8 200-400 mesh ion-exchange resin (Acros Organics) until the pH was neutral (indicated by pH strips). The mixture was vacuum filtered, and the mother liquor was collected and concentrated to afford intermediate **8dc** as a pale orange oil (6.63 g, 96%) requiring no further purification. ^{1}H NMR spectra matched previously published spectra.[6]

Synthesis of 8d

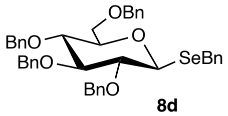

8d

Glucosyl intermediate **8dc** (6.63 g, 19.9 mmol) was dissolved in 100 mL of DMF. The reaction was cooled to 0 °C and allowed to stir for 20 minutes. After this time, TBAI (1.28 g, 3.46 mmol) was added and 60% sodium hydride in oil (3.84 g, 96 mmol) was carefully added portion wise. The reaction was capped with a septum and placed under nitrogen atmosphere. Lastly, benzyl bromide (19.0 mL, 160 mmol) was added dropwise using a syringe over a period of 3 minutes. The reaction was allowed to gradually warm to room temperature and stirred for 18 hours. After this time, the reaction was cooled to 0 °C and slowly quenched with ice cold water (300 mL). The crude reaction mixture was transferred to a separatory funnel and the aqueous layer was extracted using ethyl acetate (3 x 200 mL) and washed with ice cold water (2 x 300 mL). The combined organic layers were dried over sodium sulfate and concentrated. The crude reaction mixture was purified via flash column chromatography using a solvent gradient of 5-10% ethyl acetate in hexanes to afford glucosyl donor **8d** as a white solid (10.6 g, 77%). ^{1}H NMR spectra matched previously published spectra.[7]

Preparation of Galactosyl Donor 8e

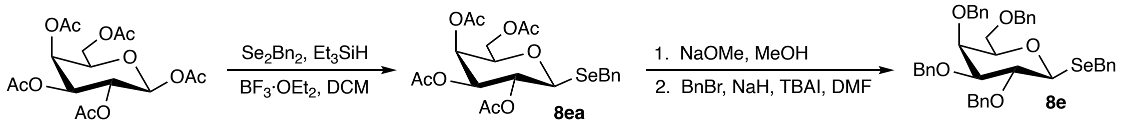

Synthesis of 8ea

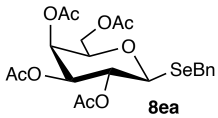

A solution of β-D-galactose pentaacetate (4.84 g, 12.4 mmol) in dichloromethane (20 mL) was treated with dibenzyl diselenide (3.02 g, 8.88 mmol) and boron trifluoride etherate (1.6 mL, 13 mmol) at room temperature. After stirring for 10 minutes, triethylsilane (2.1 mL, 13 mmol) was added and the reaction was allowed to stir at room temperature for 24 hours. The reaction was quenched with water (50 mL) and the crude mixture was transferred to a separatory funnel. The aqueous layer was extracted with dichloromethane (2 x 50 mL). The combined organic layers were dried over sodium sulfate and concentrated. The crude reaction mixture was purified via flash column chromatography using a solvent gradient of 5-15% ethyl acetate in hexanes to afford **8ea** as a white solid (4.10 g, 92%).

^{1}H NMR (400 MHz, CDCl$_3$) *δ* 7.31-7.27 (m, 4H), 7.25-7.22 (m, 1H), 5.43-5.42 (m, 1H), 5.36-5.31 (m, 1H), 4.98 (dd, *J* = 9.9 Hz, *J* = 3.4 Hz, 1H), 4.54 (d, *J* = 10.2 Hz, 1H), 4.18-4.08 (m, 2H), 4.06 (d, *J* = 11.6 Hz, 1H), 3.94 (d, *J* = 11.6 Hz, 1H), 3.84-3.80 (m, 1H), 2.16 (s, 3H), 2.08 (s, 3H), 2.01 (s, 3H), 1.97 (s, 3H). **^{13}C NMR (125 MHz, CDCl$_3$) *δ*** 170.4, 170.2, 170.0, 169.7, 138.0, 129.1, 128.6, 127.1, 77.5, 75.6, 71.6, 67.8, 67.4, 61.6, 26.7, 20.8, 20.7, 20.7, 20.6. **HRMS (ESI)**: m/z **[M+Na]+** calcd for $C_{21}H_{26}O_9SeNa$ 525.0639 found 525.0640. **[α]$_D^{25}$** = -64.4° (*c* 0.01, CH_2Cl_2). **IR (cm^{-1})** 1738, 1365, 1233, 1209, 1072, 1043, 954, 908, 760, 698, 624, 529, 464, 421.

Synthesis of 8e

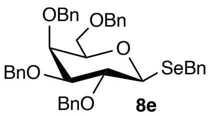

8e

A solution of **8ea** (2.96 g, 5.90 mmol) in methanol (50 mL) was treated with the dropwise addition of 5M sodium methoxide (0.17 mL, 0.85 mmol) at room temperature. The reaction was monitored via thin layer chromatography and showed completion after stirring for 15 minutes. Upon completion, the reaction was quenched using Dowex® 50WX8 200-400 mesh ion-exchange resin (Acros Organics) until the pH was neutral (indicated by pH strips). The mixture was vacuum filtered, and the mother liquor was collected and concentrated to afford the deprotected intermediate tetraol as a pale orange oil (1.97 g) requiring no further purification. Tetraol was dissolved in 100 mL of DMF. The reaction mixture was cooled to 0 °C and allowed to stir for 20 minutes. After this time, TBAI (0.39 g, 1.1 mmol) was added and 60% sodium hydride in oil (1.42 g, 35.5 mmol) was carefully added portion wise. The reaction was capped with a septum and placed under nitrogen atmosphere. Lastly, benzyl bromide (7.0 mL, 59 mmol) was added dropwise using a syringe (over a period of 2 minutes). The reaction was allowed to gradually warm to room temperature and stirred for 18 hours. After this time, the reaction was cooled to 0 °C and slowly quenched with ice cold water (300 mL). The crude reaction mixture was transferred to a separatory funnel and the aqueous layer was extracted using ethyl acetate (3 x 200 mL). The combined organic layers were dried over sodium sulfate and concentrated. The crude reaction mixture was purified via flash column chromatography using a solvent gradient of 5-10% ethyl acetate in hexanes to afford galactosyl donor **8e** as a yellow oil (3.31 g, 81%, 2 steps).

^{1}H NMR (400 MHz, $CDCl_3$) *δ* 7.33-7.25 (m, 22H), 7.21-7.14 (m, 3H), 4.97 (d, *J* = 11.5 Hz, 1H), 4.73-4.66 (m, 4H), 4.63 (d, *J* = 11.6 Hz, 1H), 4.55 (d, *J* = 9.8 Hz, 1H), 4.50-4.43 (m, 2H), 4.01-3.91 (m, 4H), 3.64-3.61 (m, 2H), 3.53-3.48 (m, 2H). **^{13}C NMR (125 MHz, $CDCl_3$) *δ*** 139.2, 138.7, 138.3, 138.1, 137.8, 128.4, 128.4, 128.3, 128.3, 138.3, 138.3, 128.2, 128.2, 128.0, 127.9, 127.8,

127.8, 127.7, 127.7, 127.7, 127.6, 127.5, 127.5, 127.4, 126.6, 84.2, 78.8, 78.5, 78.4, 75.5, 74.6, 73.8, 73.5, 72.6, 68.7, 26.2. **HRMS (ESI)**: m/z **[M+NH4]+** calcd for $C_{41}H_{42}O_5SeNH_4$ 712.2541 found 712.2539. **$[\alpha]_D^{25}$** = -19.3° (*c* 0.01, CH_2Cl_2). **IR (cm^{-1})** 3028, 2862, 1495, 1453, 1359, 1087, 733, 695, 463.

Preparation of Mannosyl Donor 8f

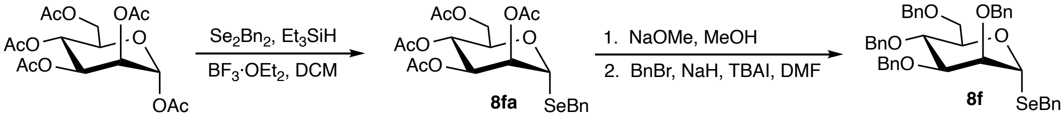

Synthesis of 8fa

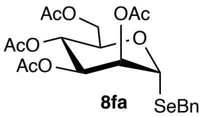

A solution of α-D-mannose pentaacetate (5.34 g, 13.7 mmol) in dichloromethane (10 mL) was treated with dibenzyl diselenide (3.33 g, 9.79 mmol) and boron trifluoride etherate (1.20 mL, 9.72 mmol) at room temperature. After stirring for 5 minutes, triethylsilane (2.50 mL, 15.7 mmol) was added and the reaction was allowed to stir at room temperature for 24 hours. The reaction was quenched with water (50 mL) and the crude mixture was transferred to a separatory funnel. The aqueous layer was extracted with dichloromethane (2 x 50 mL). The combined organic layers were dried over sodium sulfate and concentrated. The crude reaction mixture was purified via flash column chromatography using a solvent gradient of 0-15% ethyl acetate in hexanes to afford **8fa** as a white solid (2.00 g, 41%).

^{1}H NMR (400 MHz, $CDCl_3$) *δ* 7.31 (m, 4H), 7.25 (m, 1H), 5.48 (s, 1H), 5.37 (d, *J* = 2.0 Hz, 1H), 5.32 (d, *J* = 9.5 Hz, 1H), 5.29 (dd, *J* = 10.0 Hz, *J* = 3.2 Hz, 1H), 4.35-4.30 (m, 2H), 4.00 (d, *J* = 9.9 Hz, 1H), 3.91 (d, *J* = 12.2 Hz, 1H), 3.85 (d, *J* = 12.2 Hz, 1H), 2.13 (s, 3H), 2.11 (s, 3H), 2.05 (s, 3H), 1.97 (s, 3H). **^{13}C NMR (125 MHz, $CDCl_3$) *δ*** 170.6, 169.8, 169.7, 138.0, 129.0, 128.7,

127.2, 77.4, 71.2, 70.7, 70.1, 66.2, 62.3, 27.9, 20.9, 20.8, 20.7, 20.6. **HRMS (ESI)**: m/z **[M+Na]+** calcd for $C_{21}H_{26}O_9SeNa$ 525.0640 found 525.0645. **$[\alpha]_D^{25}$** = +130.1° (*c* 0.01, CH_2Cl_2). **IR (cm^{-1})** 1736, 1369, 1241, 1214, 1098, 1046, 976, 917, 840, 744, 698, 627, 532, 497, 452.

Synthesis of 8f

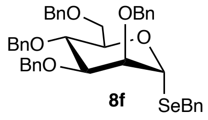

8f

A solution of **8fa** (2.00 g, 3.99 mmol) in methanol (50 mL) was treated with the dropwise addition of 5M sodium methoxide (0.11 mL, 0.55 mmol) at room temperature. The reaction was monitored via thin layer chromatography and showed completion after stirring for 20 minutes. Upon completion, the reaction was quenched using Dowex® 50WX8 200-400 mesh ion-exchange resin (Acros Organics) until the pH was neutral (indicated by pH strips). The mixture was vacuum filtered, and the mother liquor was collected and concentrated to afford the deprotected intermediate tetraol as a pale orange oil (1.33 g) requiring no further purification. The tetraol was dissolved in 100 mL of DMF. The reaction mixture was cooled to 0 °C and allowed to stir for 20 minutes. After this time, TBAI (0.39 g, 1.1 mmol) was added and 60% sodium hydride in mineral oil (1.45 g, 36.0 mmol) was carefully added portion wise. The reaction was capped with a septum and placed under nitrogen atmosphere. Lastly, benzyl bromide (3.0 mL, 25 mmol) was added dropwise using a syringe (over a period of 1 minute). The reaction was allowed to gradually warm to room temperature and stirred for 18 hours. After this time, the reaction was cooled to 0 °C and slowly quenched with ice cold water (500 mL). The crude reaction mixture was transferred to a separatory funnel, and the aqueous layer was extracted using ethyl acetate (3 x 50 mL). The combined organic layers were dried over sodium sulfate and concentrated. The crude reaction mixture was purified via flash column chromatography using a solvent gradient of 5-10% ethyl acetate in hexanes to afford mannosyl donor **8f** as a yellow oil (1.71 g, 62%, 2 steps).

^{1}H NMR (400 MHz, CDCl$_3$) ***δ*** 7.36-7.27 (m, 20H), 7.25-7.17 (m, 5H), 5.67 (d, *J* = 1.5 Hz, 1H), 4.90 (d, *J* = 10.8 Hz, 1H), 4.68 (d, *J* = 12.1 Hz 1H), 4.62 (d, *J* = 12.5 Hz 1H) , 4.54-4.47 (m, 5H), 4.05-4.02 (m, 2H), 3.87 (d, *J* = 12.1 Hz, 1H), 3.82-3.77 (m, 4H), 3.68 (m, 1H). **^{13}C NMR (125 MHz, CDCl$_3$) *δ*** 139.0, 138.5, 138.4, 138.1, 137.9, 129.0, 128.5, 128.4, 128.3, 128.3, 128.0, 127.9, 127.9, 127.8, 127.7, 127.6, 127.6, 127.5, 126.8, 80.8, 78.3, 76.6, 75.2, 74.9, 74.1, 73.3, 72.0, 71.5, 69.0, 27.6. **HRMS (ESI)**: m/z **[M+Na]+** calcd for $C_{41}H_{42}O_5SeNa$ 717.2095 found 717.2089. **$[\alpha]_D^{25}$** = +127.1° (*c* 0.01, CH_2Cl_2). **IR (cm^{-1})** 3028, 2861, 1494, 1453, 1364, 1089, 1025, 790, 735, 696, 457.

Preparation of 2-Deoxy Glucosyl Donor 8g

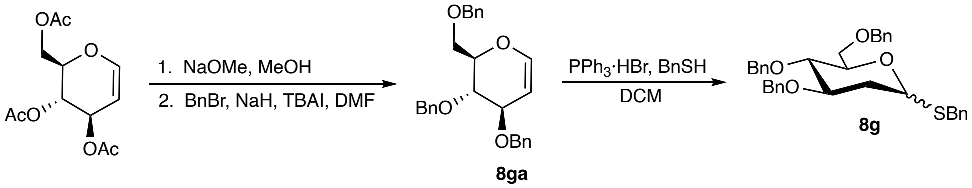

Synthesis of 8ga

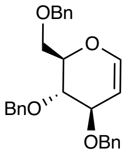

8ga

Tri-*O*-acetyl-D-glucal (10.0 g, 36.8 mmol) was dissolved in methanol (50 mL) and treated with dropwise addition of 5M sodium methoxide (1.0 mL, 5.0 mmol) at room temperature. The reaction was monitored via thin layer chromatography and showed completion after stirring for 30 minutes. Upon completion, the reaction was quenched using Dowex® 50WX8 200-400 mesh ion-exchange resin (Acros Organics) until the pH was neutral (indicated by pH strips). The mixture was vacuum filtered, and the mother liquor was collected and concentrated to afford the deprotected intermediate as a pale orange oil (5.35 g) requiring no further purification. Deprotected intermediate triol was dissolved in 100 mL of DMF. The reaction mixture was cooled to 0 °C and

allowed to stir for 20 minutes. After this time, TBAI (2.7 g, 7.4 mmol) was added and 60% sodium hydride in mineral oil (5.30 g, 221 mmol) was carefully added portion wise. The reaction mixture was then capped with a septum and placed under nitrogen atmosphere. Lastly, benzyl bromide (26.0 mL, 219 mmol) was added dropwise using a syringe (over a period of 3 minutes). The reaction was allowed to gradually warm to room temperature and stirred for 24 hours. After this time, the reaction was cooled to 0 °C and slowly quenched with ice cold water (500 mL). The crude reaction mixture was transferred to a separatory funnel and the aqueous layer was extracted using ethyl acetate (3 x 50 mL). The combined organic layers were dried over sodium sulfate and concentrated. The crude reaction mixture was purified via flash column chromatography using a solvent gradient of 0-20% ethyl acetate in hexanes to afford **8ga** as a white solid (14.0 g, 91%). ^{1}H NMR spectra matched previously published spectra.[29]

Synthesis of 8g

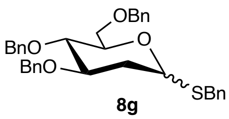

8g

Tri-*O*-benzyl-D-glucal **8ga** (4.53 g, 10.9 mmol) and triphenylphosphine hydrogen bromide (3.10 g, 9.03 mmol) were dissolved in anhydrous dichloromethane (20 mL). Benzyl mercaptan (2.6 mL, 22 mmol) was added with a syringe (in one portion). The reaction mixture was heated in an oil bath preheated to 40 °C for 1.5 hour. After this time, the reaction was removed from heat and allowed to cool to room temperature. The crude mixture was diluted with 100 mL of dichloromethane and the organic layer was washed with 100 mL of saturated sodium bicarbonate and 100 mL of water. The combined organic layers were dried over sodium sulfate and concentrated. The crude reaction mixture was purified via flash column chromatography using a solvent gradient of 0-4% ethyl acetate in hexanes to afford **8g** as a white solid (3.39 g, 53%, 1:2.1,

α:β). **α-8g** and **β-8g** were isolated during the purification of **8g** using the flash column chromatography conditions detailed above.

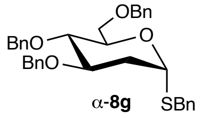

α-**8g**

^{1}H NMR (500 MHz, CDCl$_3$) *δ* 7.36-7.27 (m, 16H), 7.25-7.18 (m, 4H), 5.29 (d, *J* = 5.7 Hz, 1H), 4.88 (d, *J* = 10.9 Hz, 1H), 4.67-4.59 (m, 4H), 4.53-4.50 (m, 2H), 4.13 (m, 1H), 3.93 (m, 1H), 3.78-3.72 (m, 2H), 3.65-3.54 (m, 2H), 2.21 (dd, *J* = 13.6 Hz, *J* = 5.2 Hz, 1H), 2.03 (m, 1H). **^{13}C NMR (125 MHz, CDCl$_3$) *δ*** 140.9, 138.5, 138.4, 138.2, 138.1, 129.0, 128.6, 128.4, 128.4, 128.3, 128.3, 127.9, 127.9, 127.7, 127.6, 127.6, 127.6, 127.0, 126.9, 79.3, 78.5, 78.1, 74.9, 73.4, 71.8, 71.1, 68.8, 35.4, 34.6. **HRMS (ESI)**: m/z **[M+Na]+** calcd for $C_{34}H_{36}O_4SNa$ 563.2232 found 563.2222 found. **[α]$_D^{25}$** = +166.6° (*c* 0.01, CH_2Cl_2). **IR (cm^{-1})** 2962, 1495, 1453, 1362, 1087, 1011, 794, 736, 699, 405. **α-8g** is a colorless oil.

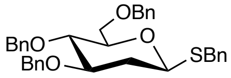

β-**8g**

^{1}H NMR (500 MHz, CDCl$_3$) *δ* 7.38-7.27 (m, 16H), 7.25-7.19 (m, 4H), 4.90 (d, *J* = 10.9 Hz, 1H), 4.66-4.60 (m, 3H), 4.57-4.52 (m, 2H), 4.22 (d, *J* = 11.6 Hz, 1H), 4.01 (d, *J* = 13.2 Hz, 1H), 3.80-3.70 (m, 3H), 3.60-3.55 (m, 1H) 3.51 (t, *J* = 8.9 Hz, 1H), 3.38 (m, 1H), 2.27 (dd, *J* = 12.6 Hz, *J* = 5.4 Hz, 1H), 1.77 (q, *J* = 11.9 Hz, 1H). **^{13}C NMR (125 MHz, CDCl$_3$) *δ*** 138.4, 138.4, 138.3, 137.8, 129.1, 128.5, 128.4, 128.4, 128.0, 127.7, 127.7, 127.5, 127.0, 80.8, 79.2, 78.1, 77.5, 75.0, 73.4, 71.4, 69.6, 36.2, 34.3. **HRMS (ESI)**: m/z **[M+Na]+** calcd for $C_{34}H_{36}O_4SNa$ 563.2232 found 563.2214. **[α]$_D^{25}$** = -103.1° (*c* 0.01, CH_2Cl_2). **IR (cm^{-1})** 2963, 1422, 1263, 1090, 1009, 865, 802, 734, 704, 404. **β-8g** is a white solid.

Preparation of 2-Azido Galactosyl Donor 8h

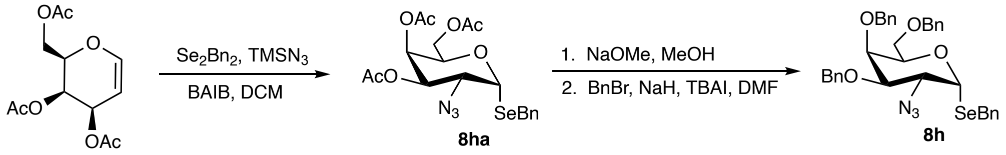

Synthesis of 8ha

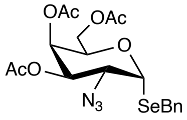

8ha

Tri-*O*-acetyl-D-galactal (2.40 g, 8.82 mmol) was dissolved in dichloromethane (20 mL). The reaction mixture was placed in an acetonitrile/dry ice bath and cooled to -60 °C. Dibenzyl diselenide (3.0 g, 8.8 mmol) and bis(acetoxy)iodobenzene (BAIB) (2.84 g, 8.82 mmol) were both added in one portion. Lastly, trimethylsilyl azide (2.4 mL, 18 mmol) was added dropwise using a syringe. The reaction was removed from the ice bath and stirred for 24 hours at room temperature. After this time, the reaction was quenched with saturated sodium bicarbonate (1 x 50 mL) and transferred to a separatory funnel. The organic layer was collected, dried over sodium sulfate and concentrated. The crude reaction mixture was purified via silica gel flash column chromatography using a solvent gradient of 5-25% ethyl acetate in hexanes to afford **8ha** (3.25 g, 76%) as a yellow solid.

^{1}H NMR (400 MHz, $CDCl_3$) *δ* 7.31-7.30 (m, 4H), 7.25-7.21 (m, 1H), 5.65 (d, *J* = 7.0 Hz, 1H), 5.45-5.44 (m, 1H), 5.11 (dd, *J* = 13.4 Hz, *J* = 4.1 Hz, 1H), 4.57 (t, *J* = 8.6 Hz, 1H), 4.18-4.12 (m, 3H), 3.83-3.73 (m, 2H), 2.16 (s, 3H), 2.07 (s, 3H), 2.03 (s, 3H). **^{13}C NMR (125 MHz, $CDCl_3$) *δ*** 170.3, 169.9, 169.6, 138.2, 128.9, 128.6, 127.0, 78.3, 71.5, 68.7, 67.2, 61.8, 58.4, 25.9, 20.7, 20.6, 20.6. **HRMS (ESI)**: m/z **[M+Na]+** calcd for $C_{19}H_{23}N_3O_7SeNa$ 508.0587; found 508.0589. **$[\alpha]_D^{25}$** = +248.1 (*c* 0.01, CH_2Cl_2). **IR (cm^{-1})** 2109, 1744, 1367, 1211, 1029, 942, 905, 758, 696, 608, 548, 441.

Synthesis of 8h

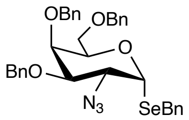

8h

A solution of **8ha** (3.25 g, 6.71 mmol) in methanol (50 mL) was treated with the dropwise addition of 5M sodium methoxide (0.20 mL, 1.0 mmol) at room temperature. The reaction was monitored via thin layer chromatography and showed completion after stirring for 15 minutes. Upon completion, the reaction was quenched using Dowex® 50WX8 200-400 mesh ion-exchange resin (Acros Organics) until the pH was neutral (indicated by pH strips). The mixture was vacuum filtered, and the mother liquor was collected and concentrated to afford the deprotected intermediate tetraol as a pale orange oil (2.39 g) requiring no further purification. Tetraol was dissolved in 100 mL of DMF. The reaction was cooled to 0 °C and allowed to stir for 20 minutes. After this time, TBAI (0.45 g, 1.2 mmol) was added and 60% sodium hydride in mineral oil (0.96 g, 24 mmol) was carefully added portion wise. The reaction was capped with a septum and placed under nitrogen atmosphere. Lastly, benzyl bromide (4.75 mL, 40.0 mmol) was added dropwise using a syringe (over a period of 2 minutes). The reaction was allowed to gradually warm to room temperature and stirred for 24 hours. After this time, the reaction was cooled to 0 °C and slowly quenched with ice cold water (300 mL). The crude reaction mixture was transferred to a separatory funnel and the aqueous layer was extracted using ethyl acetate (3 x 50 mL). The combined organic layers were dried over sodium sulfate and concentrated. The crude reaction mixture was purified via flash column chromatography using a solvent gradient of 5-10% ethyl acetate in hexanes to afford 2-azido galactosyl donor **8h** as a yellow oil (2.52 g, 60%).

^{1}H NMR (400 MHz, CDCl$_3$) *δ* 7.40-7.22 (m, 15H), 7.19-7.15 (m, 5H), 5.64 (d, *J* = 5.5 Hz, 1H), 4.89 (d, *J* = 11.3 Hz, 1H), 4.70 (s, 1H), 4.54 (d, J = 11.4 Hz, 1H), 4.51-4.42 (m, 3H), 4.27-4.23 (m, 2H), 3.97 (d, *J* = 2.7 Hz, 1H), 3.83, (d, *J* = 12.0 Hz, 1H), 3.74 (dd, *J* = 10.2 Hz, *J* = 2.7 Hz,

1H), 3.70 (d, J = 11.9 Hz, 1H), 3.60-3.50 (m, 2H). **^{13}C NMR (125 MHz, $CDCl_3$) δ** 138.9, 138.2, 137.8, 137.4, 129.0, 128.5, 128.4, 128.3, 128.2, 128.0, 127.8, 127.8, 126.7, 80.6, 79.4, 74.8, 73.6, 73.1, 72.4, 71.6, 68.9, 60.7, 25.9. **HRMS (ESI)**: m/z **[M+Na]+** calcd for $C_{34}H_{35}N_3O_4SeNa$ 652.1690 found 652.1684. **$[\alpha]_D^{25}$** = +192.4° (*c* 0.01, CH_2Cl_2). **IR (cm^{-1})** 3028, 2866, 2107, 1494, 1453, 1363, 1348, 1095, 1027, 734, 695, 459.

Glycosylation Procedures

General Glycosylation Procedure A

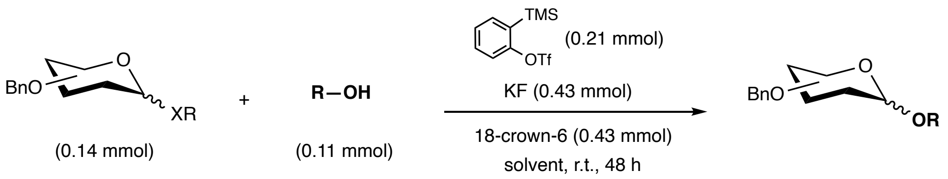

In an oven dried 4 mL Wheaton vial with a stir bar, 0.14 mmol (1.3 equiv.) of donor (**8a-8h**) was added followed by the addition of 0.11 mmol of acceptor (1.0 equiv.), 0.43 mmol of 18-crown-6 ether (4.0 equiv.), and 0.43 mmol of potassium fluoride (4.0 equiv.). The vial was capped with a septum and flushed with nitrogen gas for 5 minutes. 1.5 mL of anhydrous solvent was added with the nitrogen line still attached. Finally, 52 µL (0.21 mmol, 2.0 equiv.) of 2-(trimethylsilyl)phenyl trifluoromethanesulfonate (Kobayashi's reagent) was added dropwise using a gas tight micro syringe. The reaction was allowed to stir for 48 hours at room temperature (18-22 °C). After this time, the crude reaction mixture was concentrated and purified using flash column chromatography.

1 mmol Scale *O*-Glycosylation

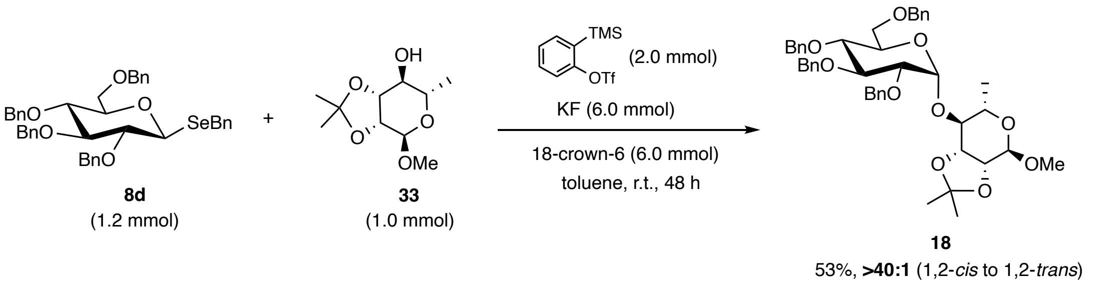

Donor **8d** (1.2 mmol, 0.84 g) and acceptor **33** (1.0 mmol, 0.22 g) were added to a 100 mL flame-dried round-bottom flask containing a dried stir bar. Then, potassium fluoride (6.0 mmol, 0.35 g) and 18-crown-6 ether (6.0 mmol, 1.6 g) were added. The round-bottom flask was then capped with a rubber septum and vacuum-purged and backfilled with nitrogen three times. Anhydrous toluene (13.8 mL) was added with a syringe followed by dropwise syringe addition of 2-(trimethylsilyl)phenyl trifluoromethanesulfonate (2.0 mmol, 0.50 mL). The reaction was stirred at room temperature (18-22 °C) for 48 hours before being concentrated under vacuum and purified by flash column chromatography (0-20% ethyl acetate in hexanes) to give disaccharide product **18** as a colorless oil (0.39 g, 53%, 1,2-*cis* to 1,2-*trans* ratio of >40:1).

Optimization Study

Table S1: Reaction Optimization

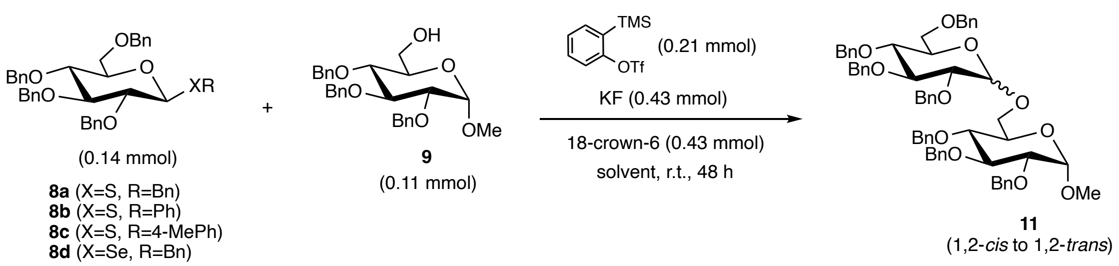

Entry	Donor	Solvent	Fluoride Source	Selectivity	Yield
1	**8a**	1,4-dioxane	KF/18-crown-6	5:1	20%
2	**8b**	1,4-dioxane	KF/18-crown-6	--	trace
3	**8a**	MTBE	KF/18-crown-6	5:1	37%
4	**8a**	1,2-dichloroethane	KF/18-crown-6	5:1	41%
5	**8a**	MeCN	CsF	1:8	22%

6	**8b**	MeCN	CsF	1:10	44%
7	**8a**	THF	KF/18-crown-6	--	trace
8	**8b**	THF	KF/18-crown-6	--	trace
9	**8a**	DCM	KF/18-crown-6	4:1	11%
10	**8b**	DCM	KF/18-crown-6	--	trace
11	**8a**	toluene	KF/18-crown-6	17:1	51%
12	**8b**	toluene	KF/18-crown-6	--	trace
13	**8a**	toluene	RbF/18-crown-6	16:1	46%
14	**8a**	*p*-xylene	KF/18-crown-6	19:1	26%
15	**8a**	mesitylene	KF/18-crown-6	9:1	28%
16	**8a**	cyclohexane	KF/-18-crown-6	12:1	34%
17	**8a**	pentane	KF/18-crown-6	6:1	23%
18	**8a**	DMF	KF/18-crown-6	--	no rxn
19	**8a**	toluene	TBAF	--	trace
20	**8a**	MTBE	TBAF	14:1	15%
21	**8c**	toluene	KF/18-crown-6	14:1	38%
22	**8c**	toluene	RbF/18-crown-6	29:1	37%
23	**8c**	MTBE	KF/18-crown-6	14:1	33%
24	**8a**	toluene	CsF/18-crown-6	--	trace
25	**8a**	MTBE	CsF/18-crown-6	--	trace
26	**8a**	toluene	KF/2,2-cryptand	--	no rxn
27	**8a**	MTBE	KF/2,2-cryptand	7:1	12%
28	**8a**[a]	toluene	KF/18-crown-6	12:1	38%
29	**8a**[b]	toluene	KF/18-crown-6	9:1	31%
30	**8a**[c]	toluene	KF/18-crown-6	5:1	27%
31	**8a**[d]	toluene	KF/18-crown-6	8:1	33%
32	**8a**[e]	toluene	KF/18-crown-6	5:1	26%
33	**8a**[f]	toluene	KF/18-crown-6	--	trace
34	**8a**	hexanes	KF/18-crown-6	10:1	26%
35	**8a**[g]	toluene	--	--	trace
36	**8a**[h]	toluene	--	--	trace
37	**8a**	toluene	TBAT	--	trace
38	**8d**	toluene	KF/18-crown-6	22:1	55%

Yields indicated as "trace" were determined via thin layer chromatography side-by-side co-spotting of the disaccharide product with the crude reaction mixture.

a: Kobayashi's reagent, KF and18-crown-6 in 10.0 equiv. (1.1 mmol); ***b***: donor in 2.0 equiv. (0.22 mmol); ***c***: donor in 3.0 equiv. (0.33 mmol); ***d***: donor in 1.0 equiv. (0.11 mmol) and acceptor in 2.0 equiv. (0.22 mmol); ***e***: reaction stirred in an acetonitrile and dry ice bath (-50 - -65 °C); ***f***: reaction refluxed in an oil bath at 60 °C; ***g***: attempted base-mediated benzyne generation using Cs_2CO_3

and 18-crown-6[21]; ***h***: attempted base-mediated benzyne generation using K_2CO_3 and 18-crown-6[21]

Information on Alcohol Acceptors

The following acceptors were synthesized according to the respective references or purchased commercially as indicated.

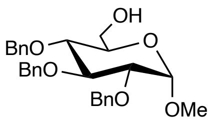

9[8]

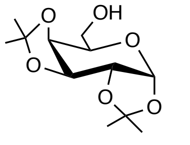

29
(purchased from Acros Organics)

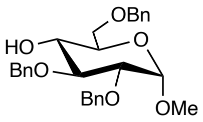

30[9]

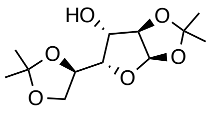

31
(purchased from TCI Chemicals)

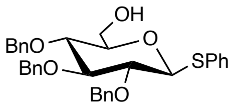

32[10]

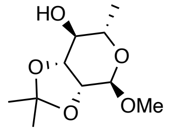

33[11]

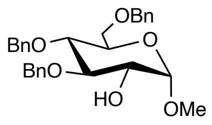

34[12]

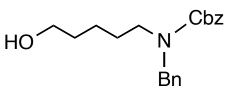

35[13]

Representative Procedures for the Substrate Scope Study

Glycosylation using acceptor **9** with glucosyl donor **8a** or **8d** to afford disaccharide **11**

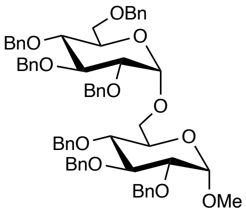

11

Following general glycosylation procedure A, glucosyl donor **8a** (91 mg) or **8d** (97 mg) (0.14 mmol), 50 mg of acceptor **9** (0.11 mmol), 114 mg of 18-crown-6 (0.430 mmol), 25 mg of potassium fluoride (0.43 mmol), 52 µL of 2-(trimethylsilyl)phenyl trifluoromethanesulfonate (0.21 mmol) and 1.5 mL of toluene were used. The crude reaction mixture was purified via flash column chromatography (5-15% ethyl acetate in hexanes) to afford disaccharide **11** as a colorless oil

(55.3 mg, 51%, 1,2-*cis* to 1,2-*trans* ratio of 17:1 when using donor **8a** and 59.4 mg, 55%,1,2-*cis* to 1,2-*trans* ratio of 22:1 when using donor **8d**). ^{1}H NMR spectra matched previously published spectra.[9]

Glycosylation using acceptor **31** with glucosyl donor **8a** or **8d** to afford disaccharide **13**

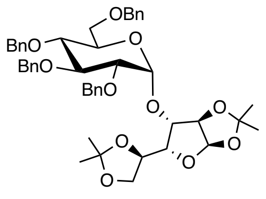

13

Following general glycosylation procedure A, glucosyl donor **8a** (91 mg) or **8d** (97 mg) (0.14 mmol), 29 mg of acceptor **31** (0.11 mmol), 114 mg of 18-crown-6 (0.430 mmol), 25 mg of potassium fluoride (0.43 mmol), 52 µL of 2-(trimethylsilyl)phenyl trifluoromethanesulfonate (0.21 mmol) and 1.5 mL of toluene were used. The crude reaction mixture was purified via flash column chromatography (5-15% ethyl acetate in hexanes) to afford disaccharide **13** as a yellow oil (55.4 mg, 64%, 1,2-*cis* to 1,2-*trans* ratio of >40:1 when using donor **8a** and 58.7 mg, 68%,1,2-*cis* to 1,2-*trans* ratio of >40:1 when using donor **8d**). ^{1}H NMR spectra matched previously published spectra.[16]

Glycosylation using acceptor **29** with glucosyl donor **8a** or **8d** to afford disaccharide **14**

14

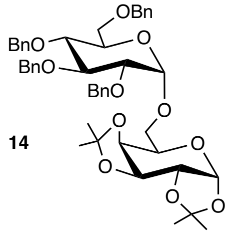

Following general glycosylation procedure A, glucosyl donor **8a** (91 mg) or **8d** (97 mg) (0.14 mmol), 29 mg of acceptor **29** (0.11 mmol), 114 mg of 18-crown-6 (0.430 mmol), 25 mg of potassium fluoride (0.43 mmol), 52 µL of 2-(trimethylsilyl)phenyl trifluoromethanesulfonate (0.21

mmol) and 1.5 mL of toluene were used. The crude reaction mixture was purified via flash column chromatography (5-15% ethyl acetate in hexanes) to afford disaccharide **14** as a colorless oil (46.0 mg, 53%, 1,2-*cis* to 1,2-*trans* ratio of 15:1 when using donor **8a** and 50.4 mg, 59%,1,2-*cis* to 1,2-*trans* ratio of 24:1 when using donor **8d**). ^{1}H NMR spectra matched previously published spectra.[14]

Glycosylation using acceptor **32** with glucosyl donor **8a** or **8d** to afford disaccharide **15**

15

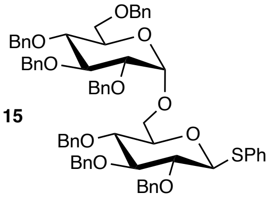

Following general glycosylation procedure A, glucosyl donor **8a** (91 mg) or **8d** (97 mg) (0.14 mmol), 60 mg of acceptor **32** (0.11 mmol), 114 mg of 18-crown-6 (0.430 mmol), 25 mg of potassium fluoride (0.43 mmol), 52 μL of 2-(trimethylsilyl)phenyl trifluoromethanesulfonate (0.21 mmol) and 1.5 mL of toluene were used. The crude reaction mixture was purified via flash column chromatography (5-15% ethyl acetate in hexanes) to afford disaccharide **15** as a colorless oil (53.7 mg, 46%, 1,2-*cis* to 1,2-*trans* ratio of 12:1 when using donor **8a** and 61.1 mg, 52%,1,2-*cis* to 1,2-*trans* ratio of 30:1 when using donor **8d**). ^{1}H NMR spectra matched previously published spectra.[17]

Glycosylation using acceptor **30** with glucosyl donor **8a** or **8d** to afford disaccharide **16**

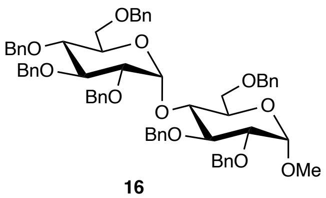

16

Following general glycosylation procedure A, glucosyl donor **8a** (91 mg) or **8d** (97 mg) (0.14 mmol), 50 mg of acceptor **30** (0.11 mmol), 114 mg of 18-crown-6 (0.430 mmol), 25 mg of

potassium fluoride (0.43 mmol), 52 µL of 2-(trimethylsilyl)phenyl trifluoromethanesulfonate (0.21 mmol) and 1.5 mL of MTBE were used. The crude reaction mixture was purified via flash column chromatography (5-15% ethyl acetate in hexanes) to afford disaccharide **16** as a colorless oil (47.0 mg, 43%, 1,2-*cis* to 1,2-*trans* ratio of 9:1 when using donor **8a** and 52.5 mg, 48%,1,2-*cis* to 1,2-*trans* ratio of 11:1 when using donor **8d**). ^{1}H NMR spectra matched previously published spectra.[15]

Glycosylation using acceptor **34** with glucosyl donor **8a** or **8d** to afford disaccharide **17**

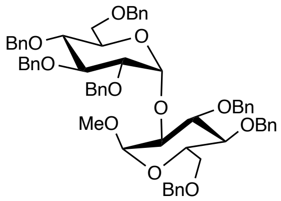

17

Following general glycosylation procedure A, glucosyl donor **8a** (91 mg) or **8d** (97 mg) (0.14 mmol), 50 mg of acceptor **34** (0.11 mmol), 114 mg of 18-crown-6 (0.430 mmol), 25 mg of potassium fluoride (0.43 mmol), 52 µL of 2-(trimethylsilyl)phenyl trifluoromethanesulfonate (0.21 mmol) and 1.5 mL of MTBE were used. The crude reaction mixture was purified via flash column chromatography (5-15% ethyl acetate in hexanes) to afford disaccharide **17** as a colorless oil (48.4 mg, 44%, 1,2-*cis* to 1,2-*trans* ratio of 23:1 when using donor **8a** and 51.1 mg, 47%,1,2-*cis* to 1,2-*trans* ratio of 37:1 when using donor **8d**). ^{1}H NMR spectra matched previously published spectra.[19]

Glycosylation using acceptor **33** with glucosyl donor **8a** or **8d** to afford disaccharide **18**

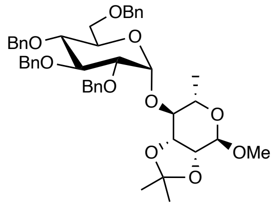

18

Following general glycosylation procedure A, glucosyl donor **8a** (91 mg) or **8d** (97 mg) (0.14 mmol), 24 mg of acceptor **33** (0.11 mmol), 114 mg of 18-crown-6 (0.430 mmol), 25 mg of potassium fluoride (0.43 mmol), 52 μL of 2-(trimethylsilyl)phenyl trifluoromethanesulfonate (0.21 mmol) and 1.5 mL of toluene were used. The crude reaction mixture was purified via flash column chromatography (5-15% ethyl acetate in hexanes) to afford disaccharide **18** as a colorless oil (45.6 mg, 56%, 1,2-*cis* to 1,2-*trans* ratio >40:1 when using donor **8a** and 49.8 mg, 61%,1,2-*cis* to 1,2-*trans* ratio >40:1 when using donor **8d**). ^{1}H NMR spectra matched previously published spectra.[18]

Glycosylation using acceptor **35** with glucosyl donor **8a** or **8d** to afford **19**

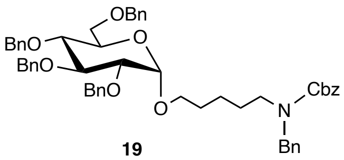

19

Following general glycosylation procedure A, glucosyl donor **8a** (91 mg) or **8d** (97 mg) (0.14 mmol), 36 mg of acceptor **35** (0.11 mmol), 114 mg of 18-crown-6 (0.430 mmol), 25 mg of potassium fluoride (0.43 mmol), 52 μL of 2-(trimethylsilyl)phenyl trifluoromethanesulfonate (0.21 mmol) and 1.5 mL of toluene were used. The crude reaction mixture was purified via flash column chromatography (5-15% ethyl acetate in hexanes) to afford disaccharide **19** as a colorless oil (50.0 mg, 53%, 1,2-*cis* to 1,2-*trans* ratio of 14:1 when using donor **8a** and 56.0 mg, 60%,1,2-*cis* to 1,2-*trans* ratio of 24:1 when using donor **8d**). ^{1}H NMR spectra matched previously published spectra.[20]

Glycosylation using acceptor **31** with galactosyl donor **8e** to afford **20**

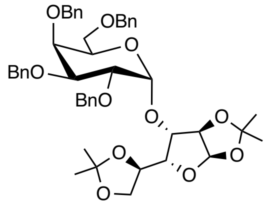

20

Following general glycosylation procedure A, galactosyl donor **8e** (97 mg, 0.14 mmol), 29 mg of acceptor **31** (0.11 mmol), 114 mg of 18-crown-6 (0.430 mmol), 25 mg of potassium fluoride (0.43 mmol), 52 µL of 2-(trimethylsilyl)phenyl trifluoromethanesulfonate (0.21 mmol) and 1.5 mL of toluene were used. The crude reaction mixture was purified via flash column chromatography (5-15% ethyl acetate in hexanes) to afford disaccharide **20** as a colorless oil (43.0 mg, 50%, 1,2-*cis* to 1,2-*trans* ratio of >40:1). ^{1}H NMR spectra matched previously published spectra.[26]

Glycosylation using acceptor **33** with galactosyl donor **8e** to afford **21**

21

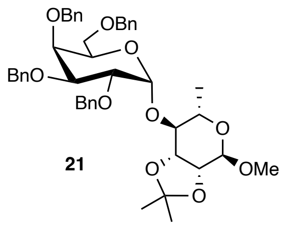

Following general glycosylation procedure A, galactosyl donor **8e** (97 mg, 0.14 mmol), 24 mg of acceptor **33** (0.11 mmol), 114 mg of 18-crown-6 ether (0.430 mmol), 25 mg of potassium fluoride (0.43 mmol), 52 µL of 2-(trimethylsilyl)phenyl trifluoromethanesulfonate (0.21 mmol) and 1.5 mL of toluene were used. The crude reaction mixture was purified via flash column chromatography (5-15% ethyl acetate in hexanes) to afford disaccharide **21** as a colorless oil (44.0 mg, 54%, 1,2-*cis* to 1,2-*trans* ratio of >40:1). ^{1}H NMR spectra matched previously published spectra.[25]

Glycosylation using acceptor **34** with mannosyl donor **8f** to afford **22**

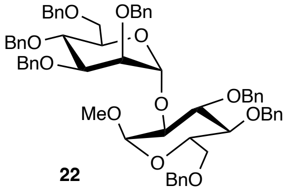

22

Following general glycosylation procedure A, mannosyl donor **8f** (97 mg, 0.14 mmol), 50 mg of acceptor **34** (0.11 mmol), 114 mg of 18-crown-6 ether (0.430 mmol), 25 mg of potassium fluoride (0.43 mmol), 52 μL of 2-(trimethylsilyl)phenyl trifluoromethanesulfonate (0.21 mmol) and 1.5 mL of MTBE were used. The crude reaction mixture was purified via flash column chromatography (5-15% ethyl acetate in hexanes) to afford disaccharide **22** as a colorless oil (45.6 mg, 42%, 1,2-*cis* to 1,2-*trans* ratio of 1:>40). ^{1}H NMR spectra matched previously published spectra.[22]

Glycosylation using acceptor **9** with mannosyl donor **8f** to afford **23**

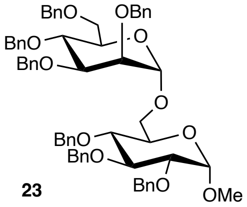

23

Following general glycosylation procedure A, mannosyl donor **8f** (97 mg, 0.14 mmol), 50 mg of acceptor **9** (0.11 mmol), 114 mg of 18-crown-6 (0.430 mmol), 25 mg of potassium fluoride (0.43 mmol), 52 μL of 2-(trimethylsilyl)phenyl trifluoromethanesulfonate (0.21 mmol) and 1.5 mL of toluene were used. The crude reaction mixture was purified via flash column chromatography (5-15% ethyl acetate in hexanes) to afford disaccharide **23** as a colorless oil (42.3 mg, 39%, 1,2-*cis* to 1,2-*trans* ratio of 1:>40). ^{1}H NMR spectra matched previously published spectra.[23]

Glycosylation using acceptor **29** with mannosyl donor **8f** to afford **24**

24

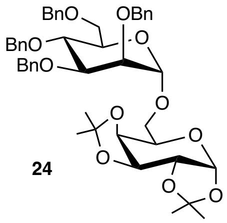

Following general glycosylation procedure A, mannosyl donor **8f** (97 mg, 0.14 mmol), 29 mg of acceptor **29** (0.11 mmol), 114 mg of 18-crown-6 (0.430 mmol), 25 mg of potassium fluoride (0.43 mmol), 52 µL of 2-(trimethylsilyl)phenyl trifluoromethanesulfonate (0.21 mmol) and 1.5 mL of toluene were used. The crude reaction mixture was purified via flash column chromatography (5-15% ethyl acetate in hexanes) to afford disaccharide **24** as a colorless oil (41.3 mg, 48%, 1,2-*cis* to 1,2-*trans* ratio of 1:>40). ^{1}H NMR spectra matched previously published spectra.[24]

Glycosylation using acceptor **9** with 2-deoxy glucosyl donor **β-8g** to afford α–**25**

α-**25**

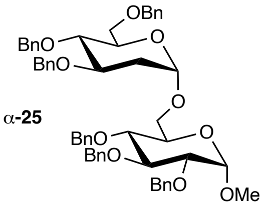

Following general glycosylation procedure A, 2-deoxy glucosyl donor **β-8g** (76 mg) (0.14 mmol), 50 mg of acceptor **9** (0.11 mmol), 114 mg of 18-crown-6 (0.430 mmol), 25 mg of potassium fluoride (0.43 mmol), 52 µL of 2-(trimethylsilyl)phenyl trifluoromethanesulfonate (0.21 mmol) and 1.5 mL of toluene were used. The crude reaction mixture was purified via flash column chromatography (5-15% ethyl acetate in hexanes) to afford disaccharide **α-25** as a colorless oil (42.6 mg, 44%, alpha only, >40:1). ^{1}H NMR spectra matched previously published spectra.[27]

Glycosylation using acceptor **9** with 2-deoxy glucosyl donor **α–8g** to afford **β–25**

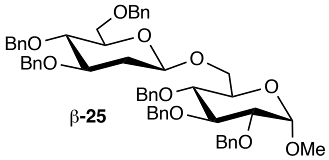

β-**25**

Following general glycosylation procedure A, 2-deoxy glucosyl donor **α–8g** (76 mg, 0.14 mmol), 50 mg of acceptor **9** (0.11 mmol), 114 mg of 18-crown-6 (0.430 mmol), 25 mg of potassium fluoride (0.43 mmol), 52 μL of 2-(trimethylsilyl)phenyl trifluoromethanesulfonate (0.21 mmol) and 1.5 mL of toluene were used. The crude reaction mixture was purified via flash column chromatography (5-15% ethyl acetate in hexanes) to afford disaccharide **β–25** as a colorless oil (39.7 mg, 41%, beta only, >40:1). ^{1}H NMR spectra matched previously published spectra.[27]

Glycosylation using acceptor **29** with 2-deoxy glucosyl donor **β-8g** to afford **α–26**

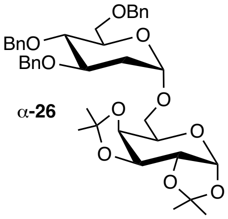

α-**26**

Following general glycosylation procedure A, 2-deoxy glucosyl donor **β-8g** (76 mg, 0.14 mmol), 29 mg of acceptor **29** (0.11 mmol), 114 mg of 18-crown-6 (0.430 mmol), 25 mg of potassium fluoride (0.43 mmol), 52 μL of 2-(trimethylsilyl)phenyl trifluoromethanesulfonate (0.21 mmol) and 1.5 mL of toluene were used. The crude reaction mixture was purified via flash column chromatography (5-15% ethyl acetate in hexanes) to afford disaccharide **α–26** as a colorless oil (42.4 mg, 57%, alpha only, >40:1). ^{1}H NMR spectra matched previously published spectra.[27]

Glycosylation using acceptor **29** with 2-deoxy glucosyl donor **α–8g** to afford **β–26**

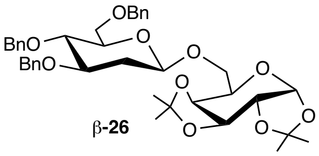

β-**26**

Following general glycosylation procedure A, 2-deoxy glucosyl donor **α–8g** (76 mg, 0.14 mmol), 29 mg of acceptor **29** (0.11 mmol), 114 mg of 18-crown-6 (0.430 mmol), 25 mg of potassium fluoride (0.43 mmol), 52 μL of 2-(trimethylsilyl)phenyl trifluoromethanesulfonate (0.21 mmol) and 1.5 mL of toluene were used. The crude reaction mixture was purified via flash column chromatography (5-15% ethyl acetate in hexanes) to afford disaccharide **β–26** as a colorless oil (46.9 mg, 63%, alpha to beta ratio of 1:9). ^{1}H NMR spectra matched previously published spectra.[27]

Glycosylation using acceptor **34** with 2-azido galactosyl donor **8h** to afford **27**

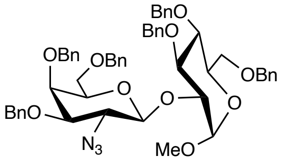

27

Following general glycosylation procedure A, 2-azido galactosyl donor **8h** (88 mg, 0.14 mmol), 50 mg of acceptor **34** (0.11 mmol), 114 mg of 18-crown-6 (0.430 mmol), 25 mg of potassium fluoride (0.43 mmol), 52 μL of 2-(trimethylsilyl)phenyl trifluoromethanesulfonate (0.21 mmol) and 1.5 mL of MTBE were used. The crude reaction mixture was purified via flash column chromatography (5-15% ethyl acetate in hexanes) to afford disaccharide **27** as a yellow oil (21.0 mg, 21%, 1,2-*cis* to 1,2-*trans* ratio of 1:10). ^{1}H NMR spectra matched previously published spectra.[28]

Glycosylation using acceptor **29** with 2-azido galactosyl donor **8h** to afford **28**

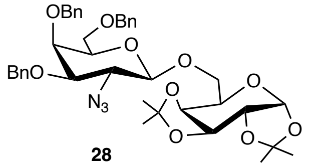

28

Following general glycosylation procedure A, 2-azido galactosyl donor **8h** (88 mg, 0.14 mmol), 29 mg of acceptor **29** (0.11 mmol), 114 mg of 18-crown-6 (0.430 mmol), 25 mg of potassium fluoride (0.43 mmol), 52 µL of 2-(trimethylsilyl)phenyl trifluoromethanesulfonate (0.21 mmol) and 1.5 mL of toluene were used. The crude reaction mixture was purified via flash column chromatography (5-15% ethyl acetate in hexanes) to afford disaccharide **28** as a yellow oil (22.1 mg, 28%, 1,2-*cis* to 1,2-*trans* ratio of 1:>40). ^{1}H NMR spectra matched previously published spectra.[28]

Experimental Procedure and Characterization of the Isolated Side Product (12)

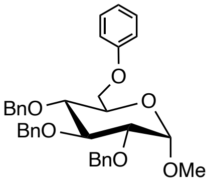

12

In a 4mL Wheaton vial, 91 mg of glucosyl donor **8a** (0.14 mmol), 171 mg of acceptor **9** (0.420 mmol), 370 mg of 18-crown-6 (1.40 mmol), and 81 mg of potassium fluoride (1.4 mmol) were added. The vial was capped with a septum and flushed with nitrogen gas for 5 minutes. 0.6 mL of dry toluene was added with the nitrogen line still attached. Finally, 340 µL (1.40 mmol) of 2-(trimethylsilyl)phenyl trifluoromethanesulfonate was added dropwise using a gas tight micro syringe. The reaction was allowed to stir for 48 hours at room temperature (18-22 °C). The crude reaction mixture was purified via flash column chromatography (5-15% ethyl acetate in hexanes) to afford **12** as a colorless oil (22.7 mg, 30%).

^{1}H NMR (400 MHz, $CDCl_3$) *δ* 7.36-7.22 (m, 12H), 7.17-7.15 (m, 3H), 6.95-6.91 (m, 2H), 6.88-6.86 (m, 3H), 5.02 (d, *J* = 10.9 Hz, 1H), 4.88-4.84 (m, 3H), 4.79 (s, 1H), 4.70 (d, *J* = 12.1 Hz, 1H), 4.65 (d, *J* = 3.6 Hz, 1H), 4.53 (d, *J* = 10.9 Hz, 1H), 4.12-4.10 (m, 1H), 4.06 (t, *J* = 9.2 Hz, 1H), 3.93-3.89 (m, 1H), 3.76 (t, *J* = 8.9 Hz, 1H), 3.63 (dd, *J* = 9.6 Hz, *J* = 3.6 Hz, 1H), 3.38 (s, 3H). **^{13}C NMR (125 MHz, $CDCl_3$) *δ*** 158.5, 138.7, 138.1, 138.0, 129.4, 128.5, 128.4, 128.4, 128.1, 128.0, 128.0, 127.9, 127.8, 127.6, 121.0, 114.6, 98.2, 82.1, 79.9, 77.5, 77.3, 75.8, 75.2, 73.4, 69.2, 66.4, 55.2. **HRMS (ESI) m/z**: **[M + Na]+** calcd for $C_{34}H_{36}O_6Na$ 563.24041; found 563.24080. **$[\alpha]_D^{25}$** = +25.4° (*c* 0.01, CH_2Cl_2). **IR (cm^{-1})** 2925, 1598, 1495, 1453, 1361, 1243, 1070, 911, 737, 695, 462.

Determination of Configuration at the Anomeric Center

Configuration at the anomeric center was determined through comparison to already-published ^{1}H NMR data (both spectra and tabulated data) since all the *O*-glycosylation products are known to the literature. All references are included herein for compounds **11** (ref. 9), **13** (ref. 16), **14** (ref. 14), **15** (ref. 17), **16** (ref. 15), **17** (ref. 19), **18** (ref. 18), **19** (ref. 20), **20** (ref. 26), **21** (ref. 25), **22** (ref. 22), **23** (ref. 23), **24** (ref. 24), **α-25** (ref. 27), **β-25** (ref. 27), **α-26** (ref. 27), **β-26** (ref. 27), **27** (ref. 28), and **28** (ref. 28).

References

1) Doyle, L.M.; O'Sullivan, S.; Di Salvo, C.; McKinney, M.; McArdle, P.; Murphy, P.V. *Organic Letters* **2017**, *19* (21), 5802-5805.
2) Brachet, E.; Brion, J.; Messaoudi, S.; Alami, M. *Advanced Synthesis & Catalysis* **2013**, *355* (2-3), 477-490.
3) Liu, X.; Zhang, B.; Gu, X.; Chen, G.; Chen, L.; Wang, X.; Xiong, B.; You, Q.; Chen, Y.; Shen, J. *Carbohydrate Research* **2014**, *398* (29), 45-49.

4) Holmstrom, T.; Pedersen, C. *Journal of Organic Chemistry* **2019**, *84* (21), 13242-13251.

5) Manna, T.; Misra, A. *Organic & Biomolecular Chemistry* **2019**, *38* (17), 8902-8912.

6) Kumar, A.; Illyes, T.; Kover, K.; Szilagyi, L. *Carbohydrate Research* **2012**, *360*, 8-18.

7) Zhu, F.; O'Neill, S.; Rodriguez, J.; Walczak, M. *Angewandte Chemie* **2018**, *57* (24), 7091-7095.

8) Viuff, A.; Besenbacher, L.; Kamori, A.; Jensen, M.; Kilian, M.; Kato, A.; Jensen, H. *Organic & Biomolecular Chemistry* **2015**, *37* (13), 9637-9658.

9) Xia, M.; Yao, W.; Meng, X.; Qing, L.; Zhong, L. *Tetrahedron Letters* **2017**, *24* (14), 2389-2392.

10) Tani, S.; Sawadi, S.; Kojima, M.; Akai, S.; Sato, K. *Tetrahedron Letters* **2007**, *48* (17), 3103-3104.

11) Liptak, A.; Imre, J.; Nanasi, P. *Carbohydrate Research* **1981**, *1*, 154-156.

12) Lecourt, T.; Herault, A.; Pearce, A.; Sollogoub, M.; Sinay, P. *Chemistry Europe* **2004**, *10* (12), 2960-2971.

13) Mandal, S.; Ganesh, N.; Sadowska, J.; Bundle, D. *Organic & Biomolecular Chemistry* **2017**, *18* (15), 3874-3883.

14) Zhuo, M.; Wilbur, D.; Kwan, E.; Bennett, C. *Journal of the American Chemical Society* **2019**, *141* (42), 16743-16754.

15) Wen, P.; Crich, D. *Organic Letters* **2017** *19* (9), 2402-2405.

16) Zhang, C.; Zuo, H.; Lee, G.; Zou, Y.; Dang, Q.; Houk, K.; Niu, D. *Nature Chemistry* **2022**, *14*, 686-694.

17) (a) Chu, A.; Nguyen, H.; Sisel, J.; Minciunescu, A.; Bennett, C. *Organic Letters* **2013**, *15* (10), 2566-2569. (b) Kitowski, A.; Jimenez-Moreno, E.; Salvado, M.; Mestre, J.; Castillon, S.; Jimenez-Oses, G.; Boutureira, O.; Bernardes, G. *Organic Letters* **2017**, *19* (19), 5490-5493.

18) Trinderup, H.; Madsen, L.; Press, L.; Madsen, M.; Jensen, H. *The Journal of Organic Chemistry* **2022**, *87* (21), 13763-13789.

19) Wang, L. Overkleeft, H.; van der Marel, G.; Codée, J. *Journal of the American Chemical Society* **2018**, *140* (13), 4632-4638.

20) Zhang, Y.; He, H.; Chen, Z.; Huang, Y.; Xiang, G.; Li, P.; Yang, X.; Lu, G.; Xiao, G. *Angewandte Chemie International Edition*, **2021** *60*, (22), 12597-12606.

21) Yoshida, S.; Hazama, Y.; Sumida, Y.; Yano, T.; Hosoya, T. *Molecules* **2015**, *20* (6), 10131-10140.

22) Geringer, S.A.; Singh, Y.; Hoard, D.J.; Demchenko, A.V. *European Journal of Chemistry* **2020**, *26* (36), 8053-8063.

23) Ma, X.; Zheng, Z.; Fu, Y.; Zhu, X.; Peng, L.; Zhang, L. *Journal of the American Chemical Society* **2021** *143* (31), 11908-11913.

24) Kim, K.S.; Fulse, D.B.; Baek, J.Y.; Lee, B-Y.; Jeon, H.B. *Journal of the American Chemical Society* **2008** *130* (26), 8537-8547.

25) Mukhopadhyay, B.; Cura, P.; Kartha, K.P.; Botting, C.; Field, R.A. *Organic & Biomolecular Chemistry* **2005**, *3* (19), 3468-3470.

26) Grayson, E.; Ward, S.; Hall, A.; Rendle, P.; Gamblin, D.; Batsanov, A.; Davis, B. *Journal of Organic Chemistry* **2005**, 70 (*24*), 9740-9754.

27) Hsu, M.; Liu, Y.; Lin, S.; Wang, C. *Beilstein Journal of Organic Chemistry* **2016**, *12*, 1758-1764.

28) Singh, Y.; Wang, T.; Demchenko, A. *European Journal of Organic Chemistry* **2019**, *37*, 6413-6416.

29) Moons, S.J.; Mensink, R.; Bruekers, J.; Vercammen, M.; Jansen, L.M.; Boltje, T.J. *Journal of Organic Chemistry* **2019**, 84 (7), 4486-4500.

NMR (^{1}H, ^{13}C) Spectra of Glycosyl Donors and Isolated Side Product

8ab

^{1}H NMR, 400 MHz, $CDCl_3$

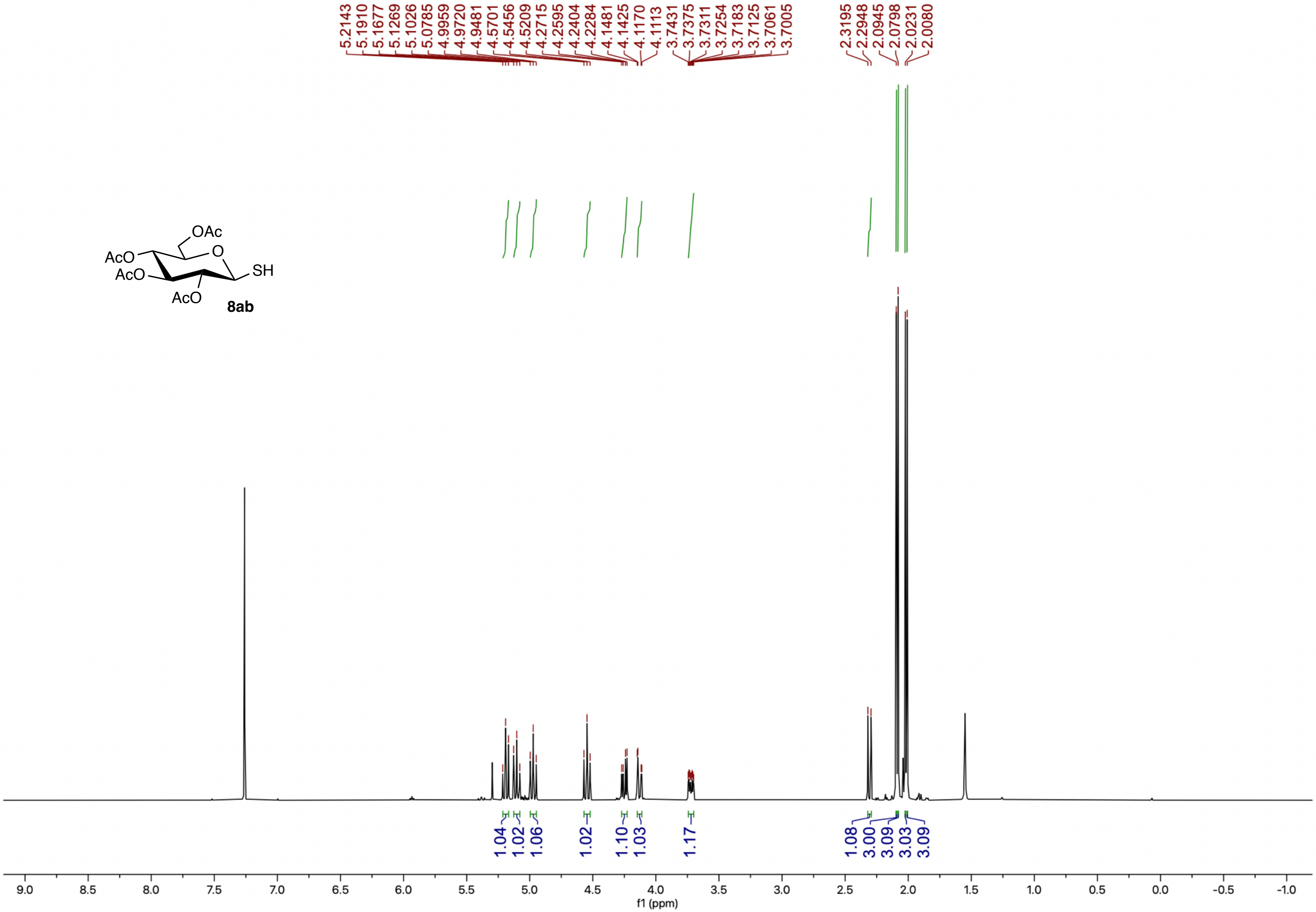

8ac

^{1}H NMR, 400 MHz, MeOD

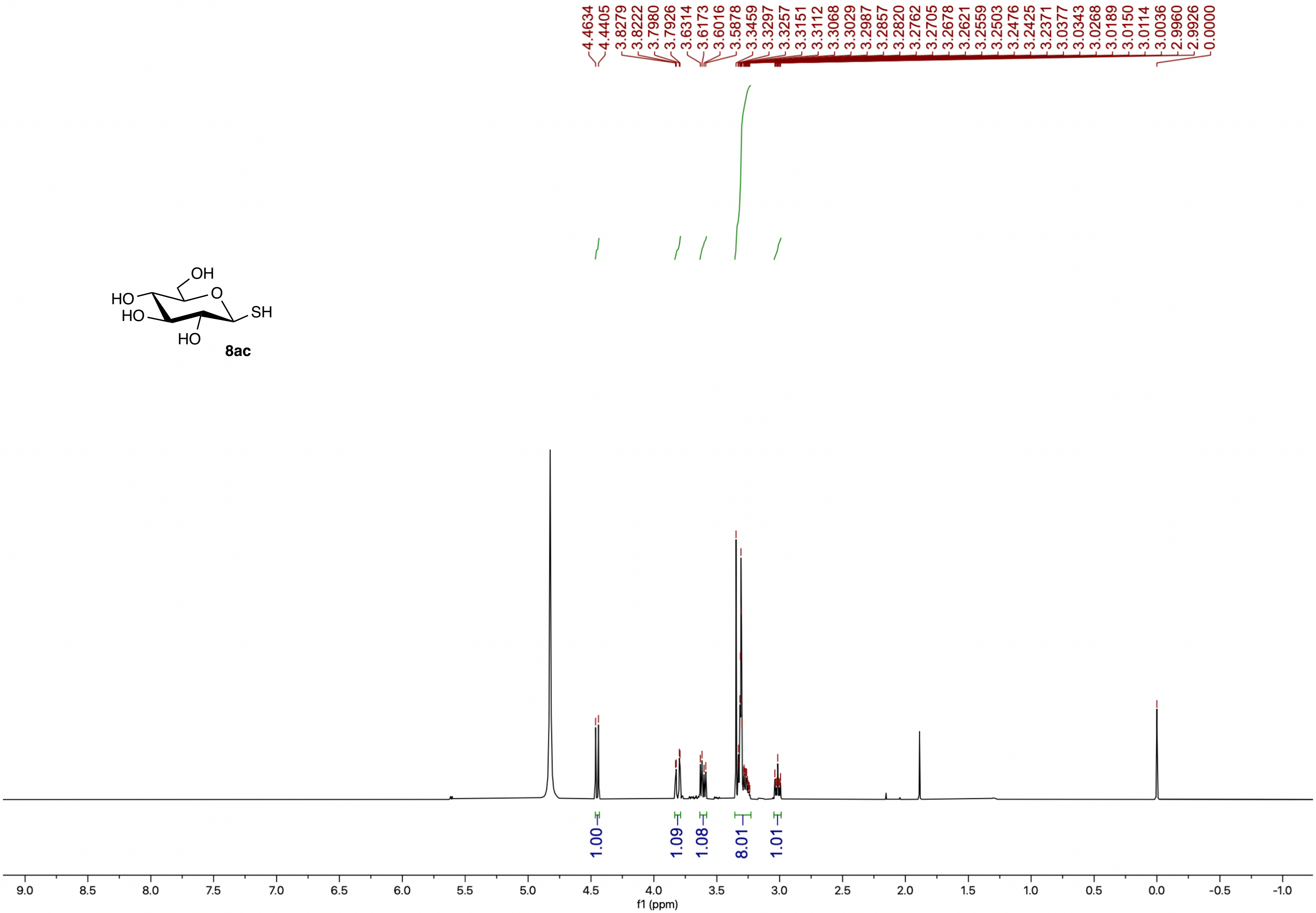

8a

^{1}H NMR, 400 MHz, $CDCl_3$ with 0.03% TMS

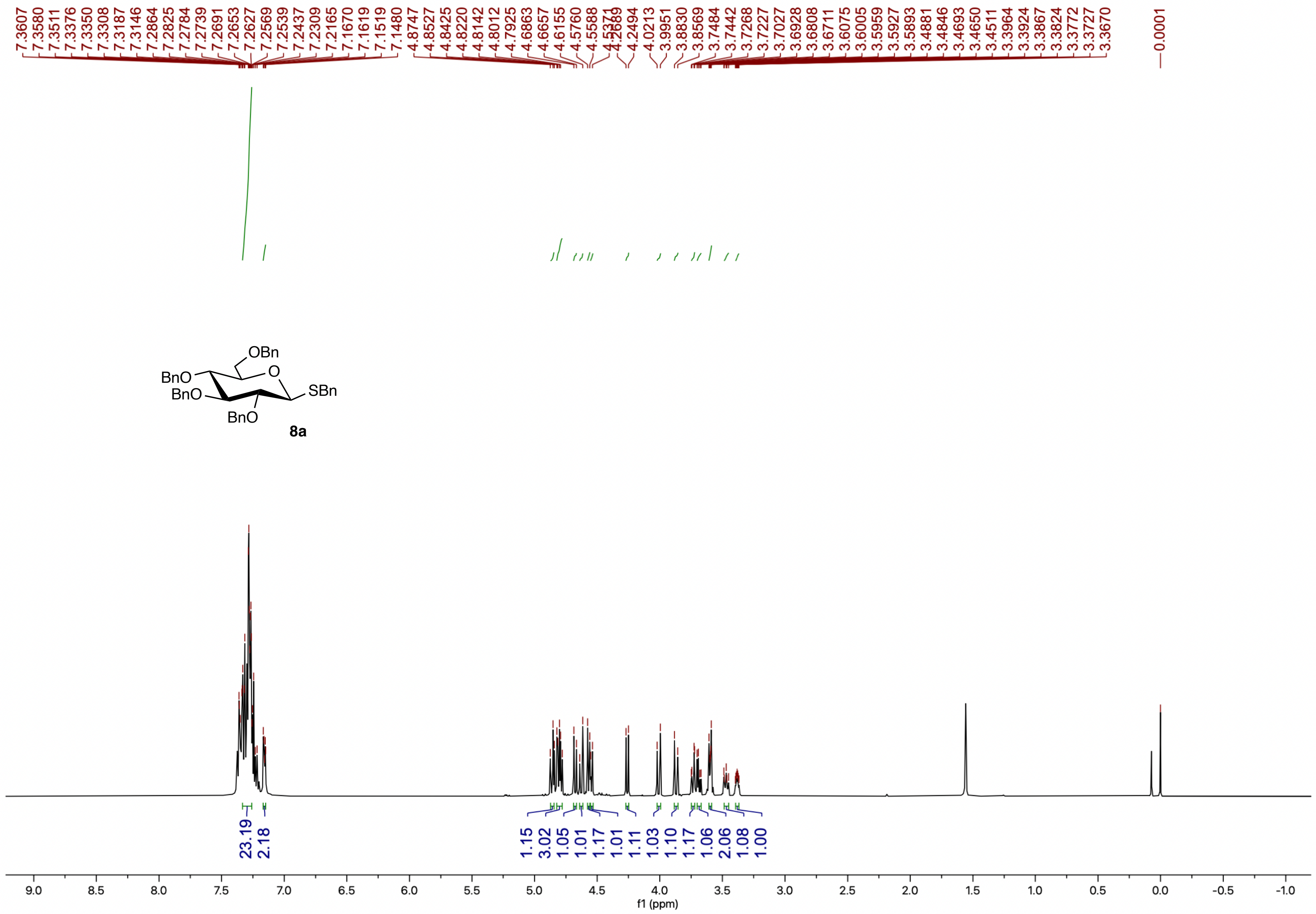

8bb

^{1}H NMR, 400 MHz, $CDCl_3$ with 0.03% TMS

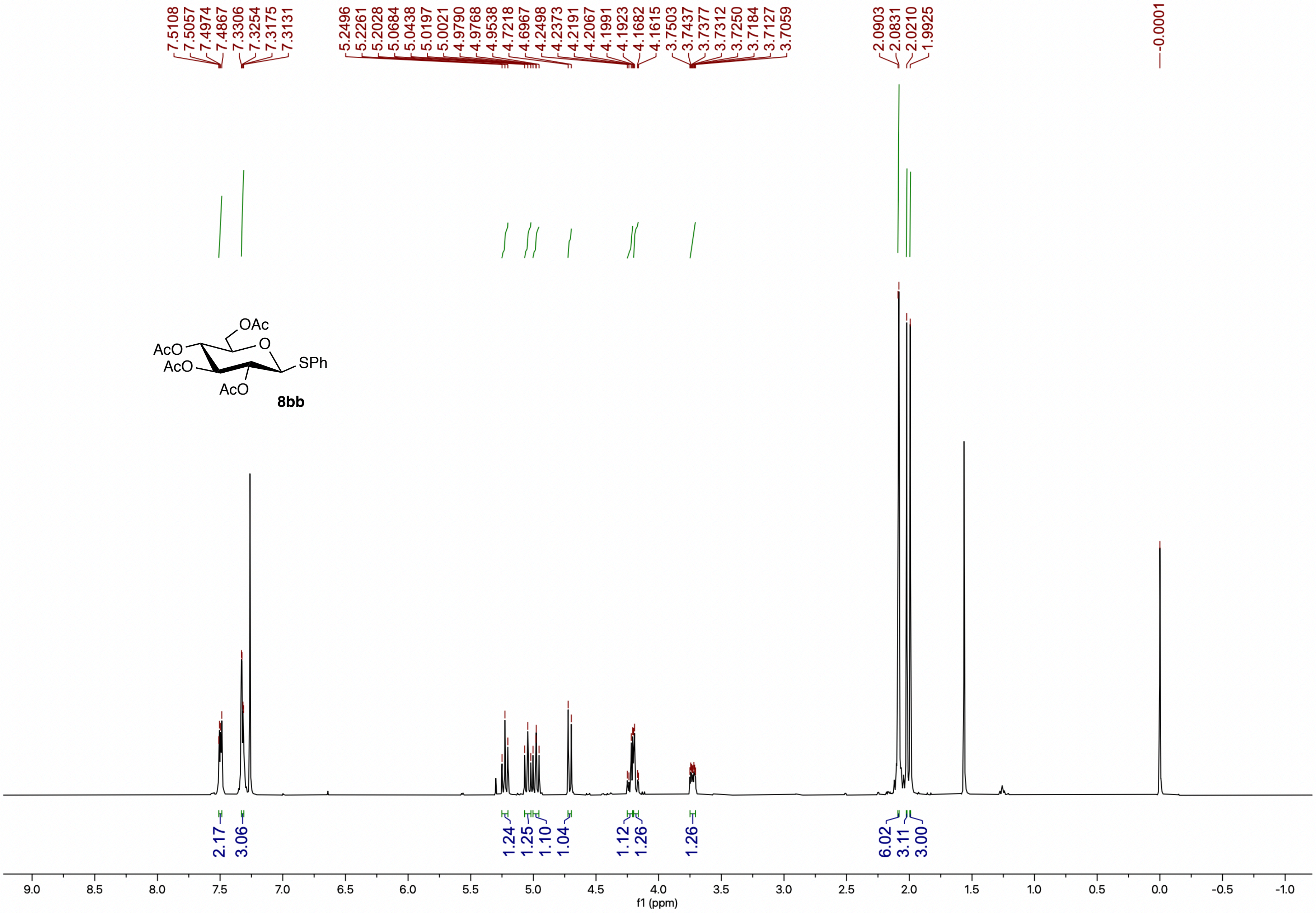

8bc

^{1}H NMR, 400 MHz, DMSO

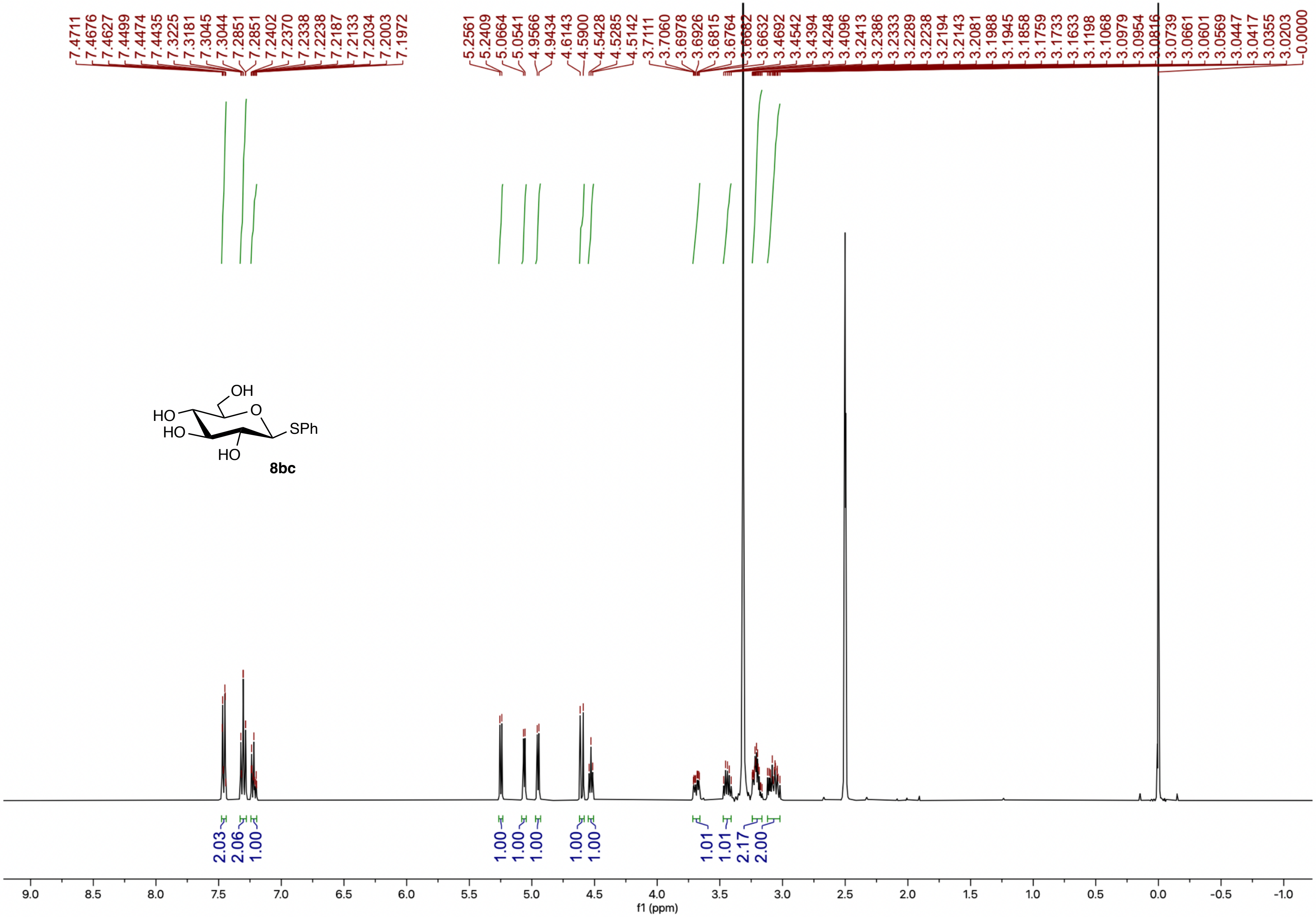

8bc

8b

^{1}H NMR, 400 MHz, $CDCl_3$ with 0.03% TMS

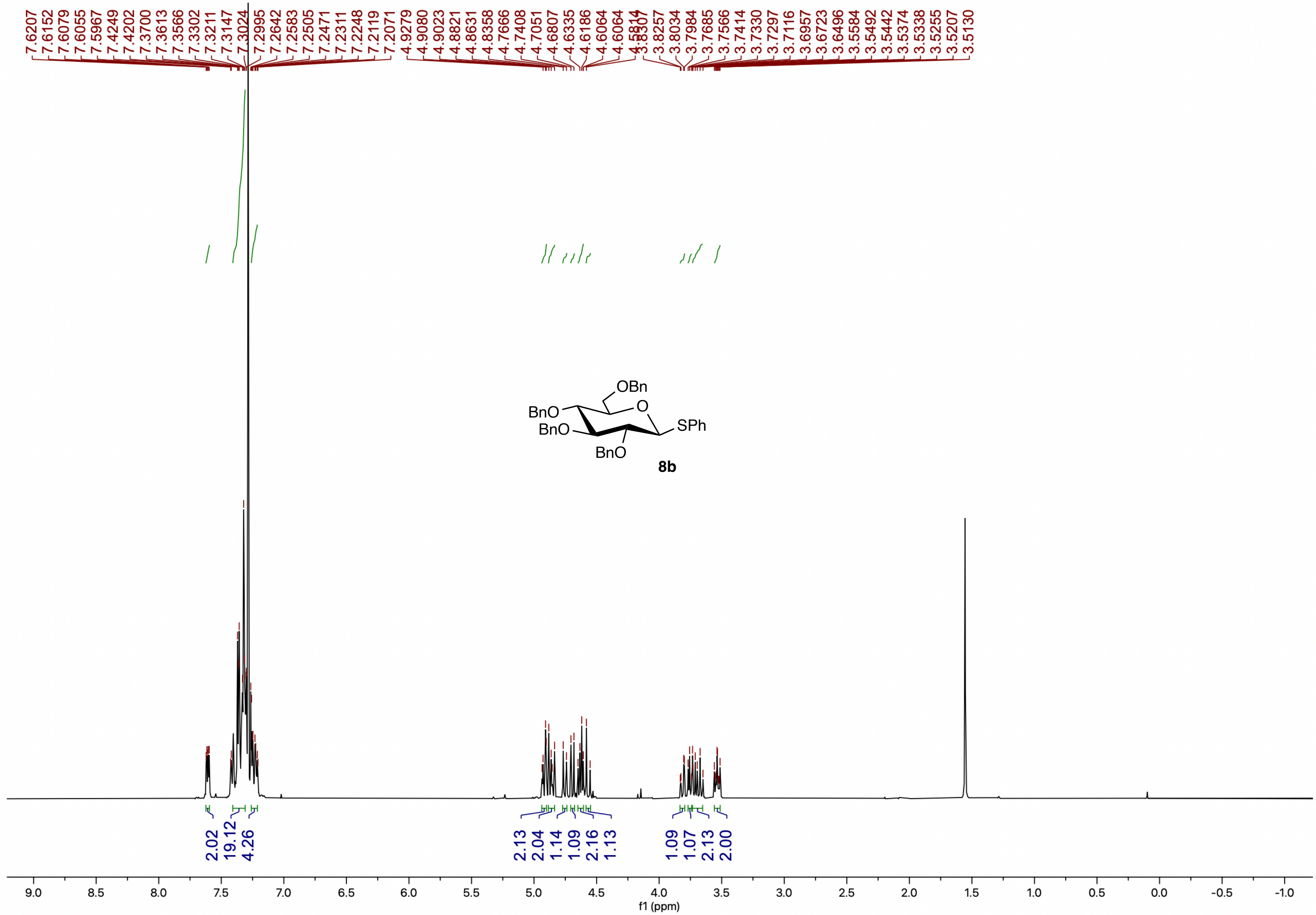

8cb

^{1}H NMR, 500 MHz, $CDCl_3$ with 0.03% TMS

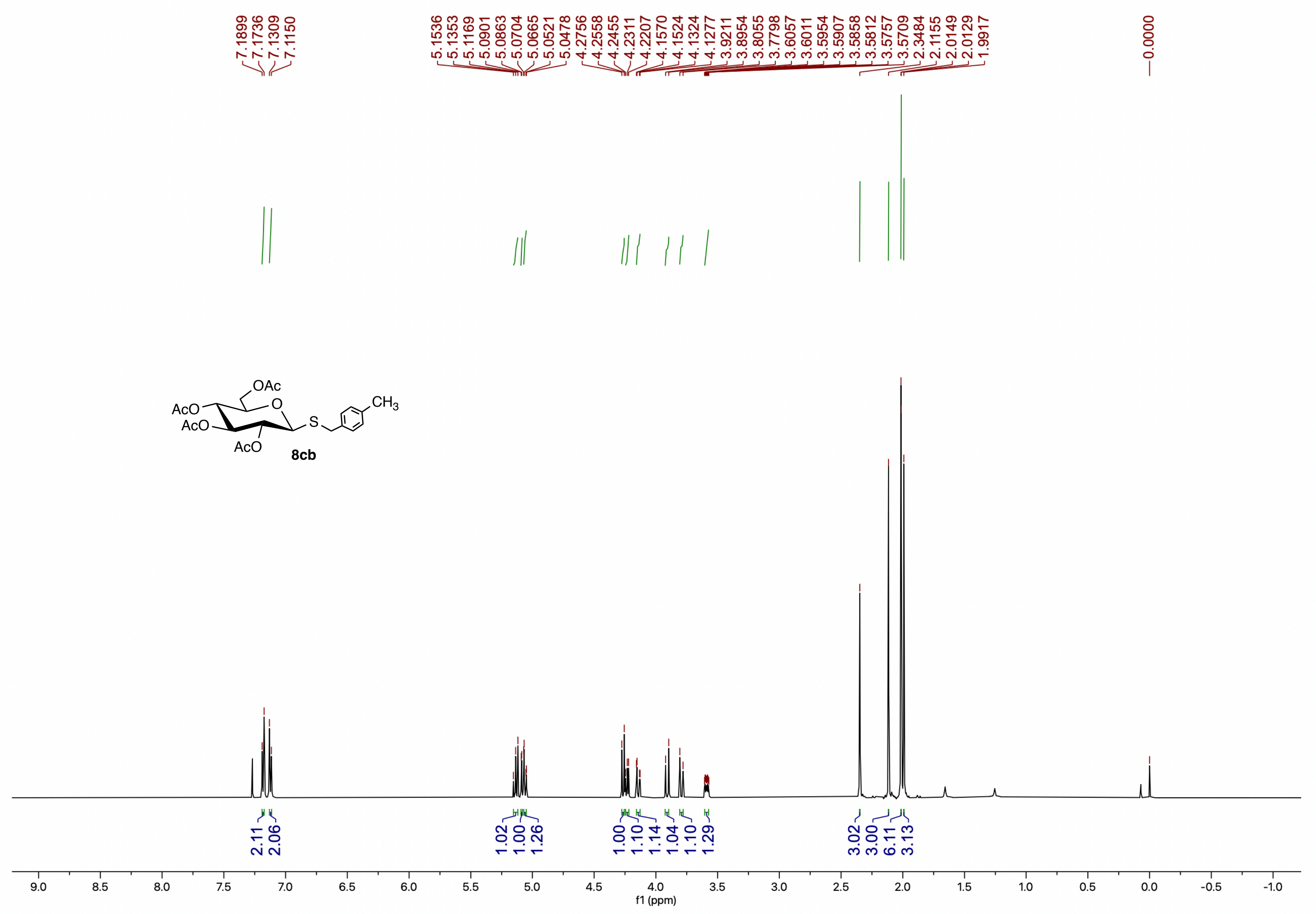

8cb

^{13}C NMR, 125 MHz, $CDCl_3$ with 0.03% TMS

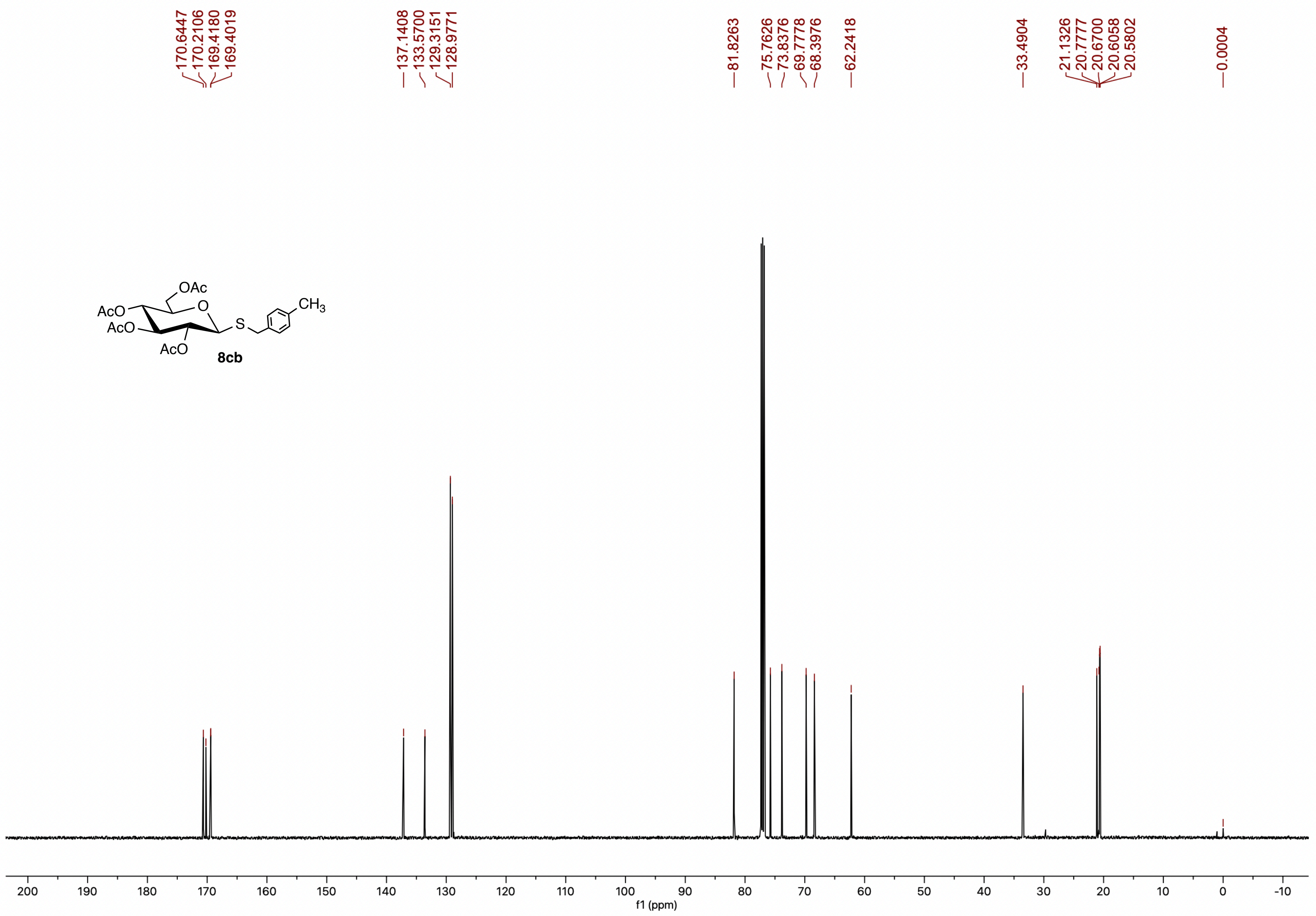

8cc

^{1}H NMR, 400 MHz, MeOD

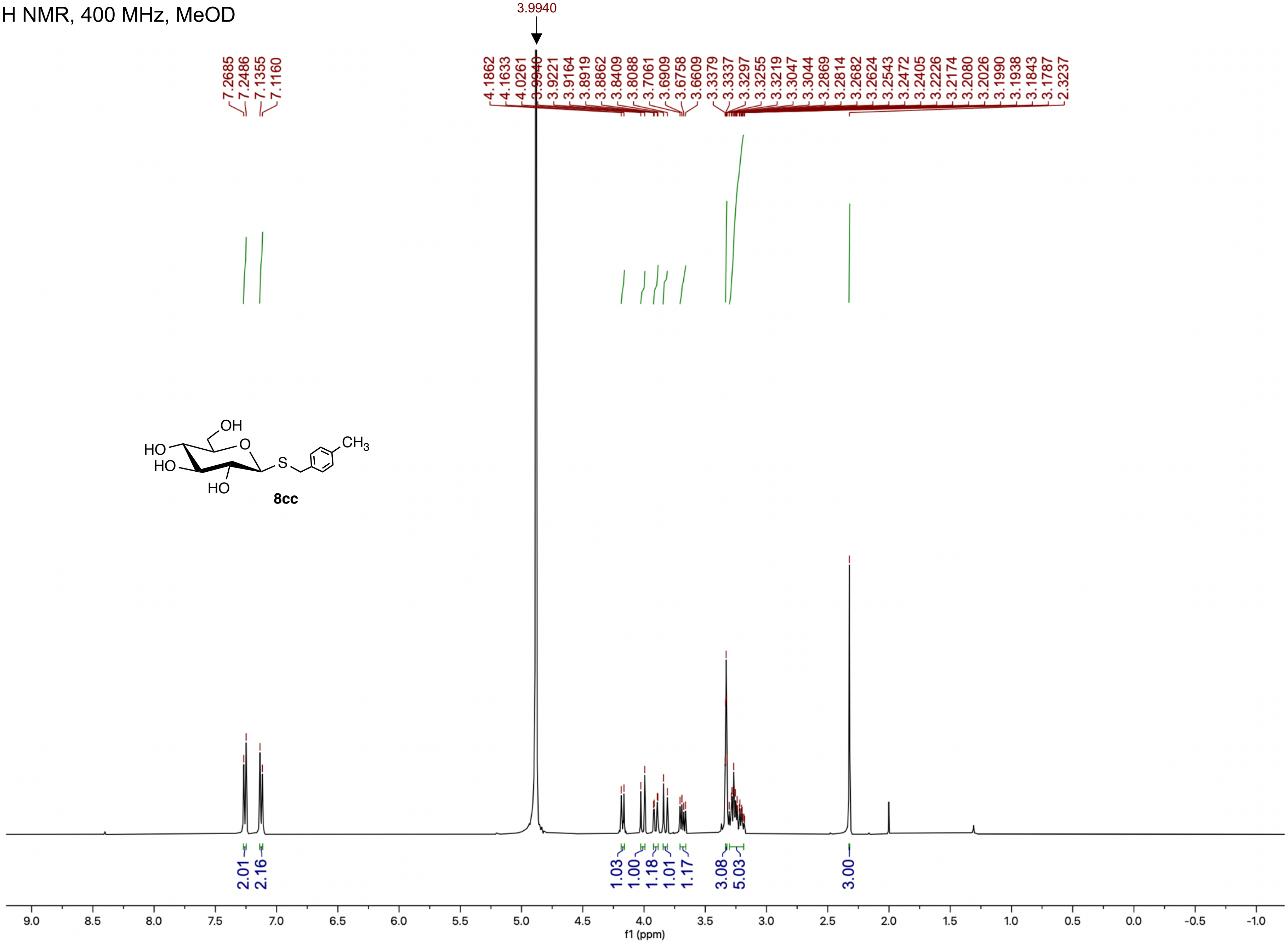

8cc

8cc

^{13}C NMR, 125 MHz, MeOD

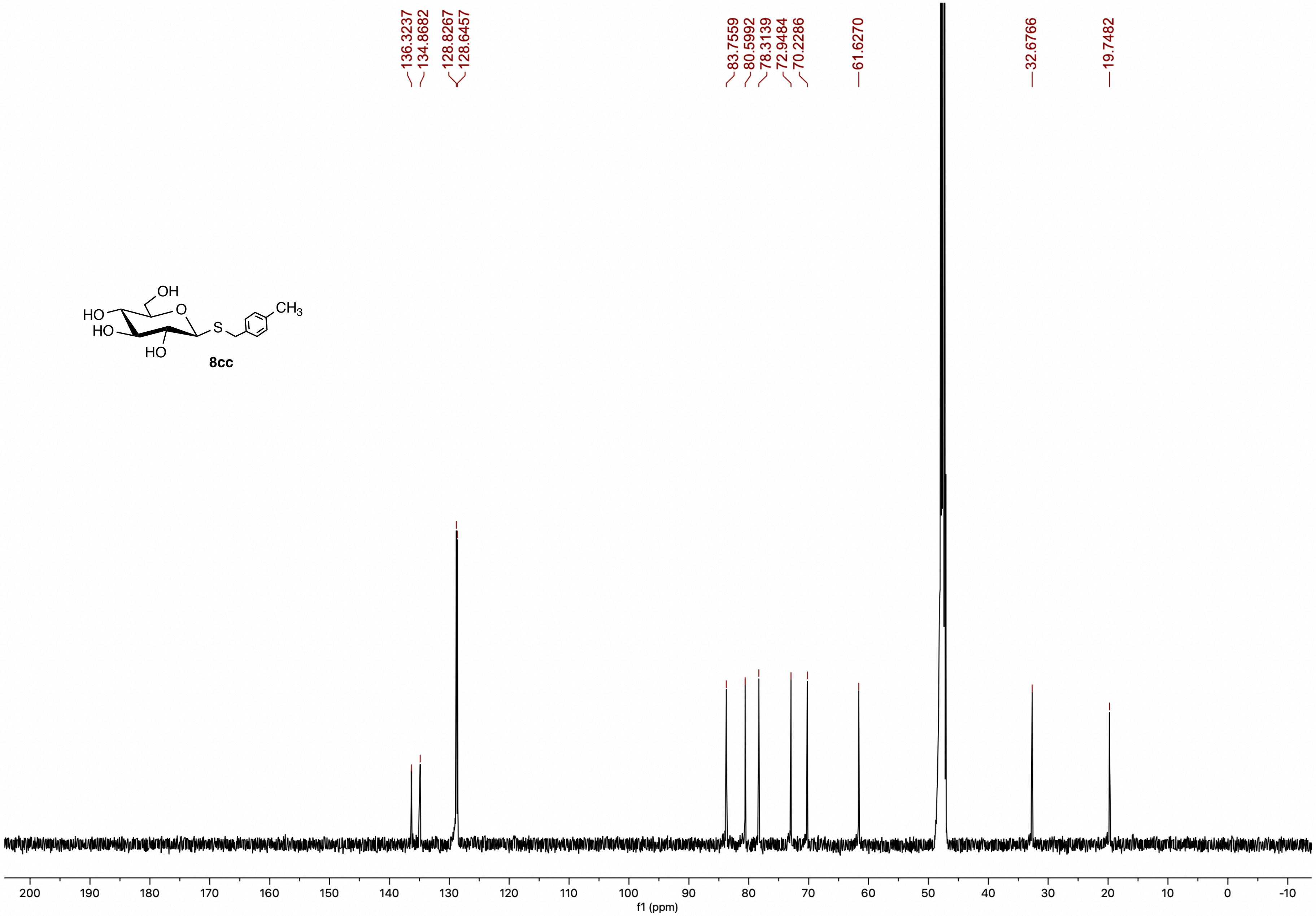

8c

^{1}H NMR, 400 MHz, $CDCl_3$ with 0.03% TMS

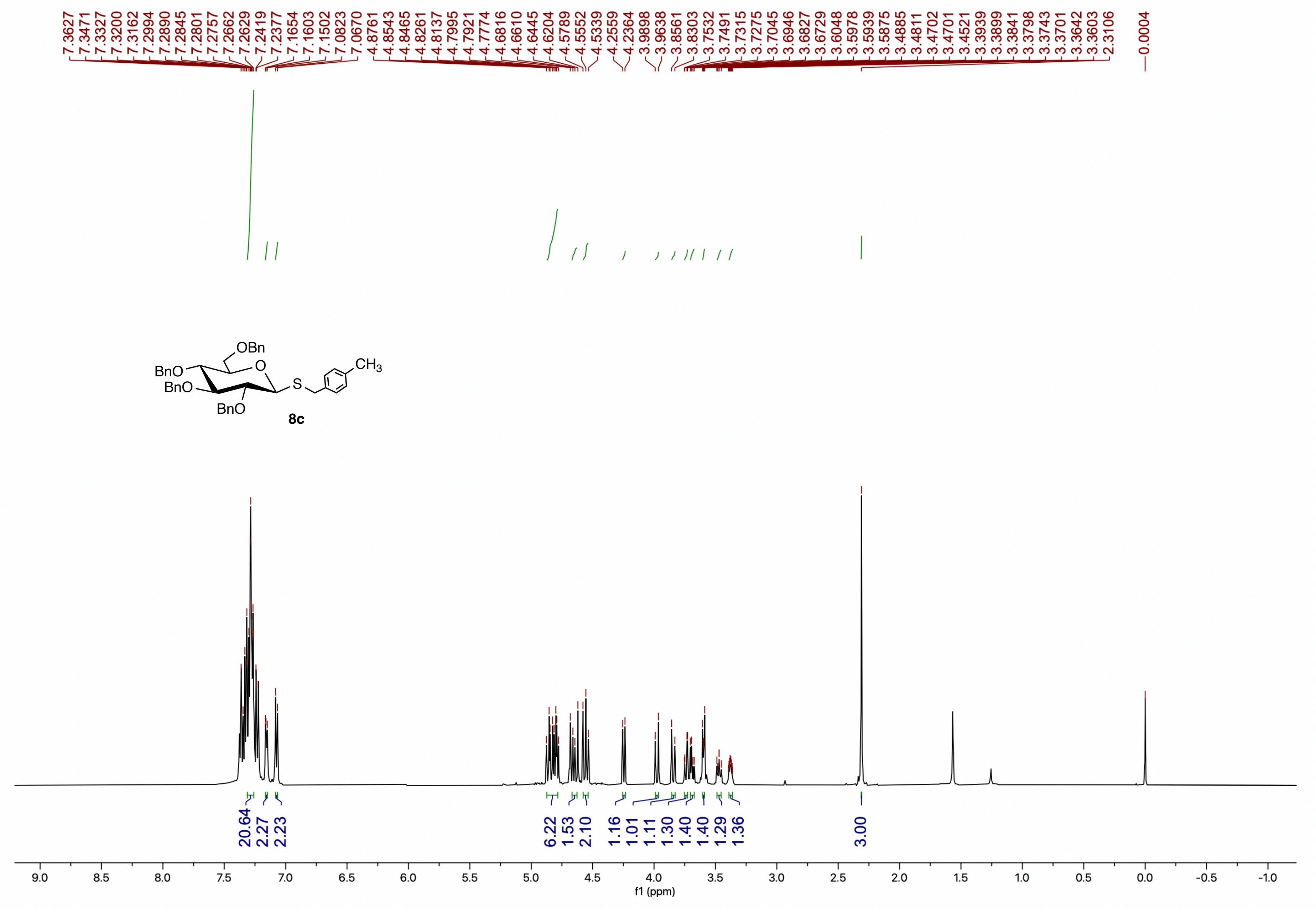

8c

^{13}C NMR, 125 MHz, $CDCl_3$ with 0.03% TMS

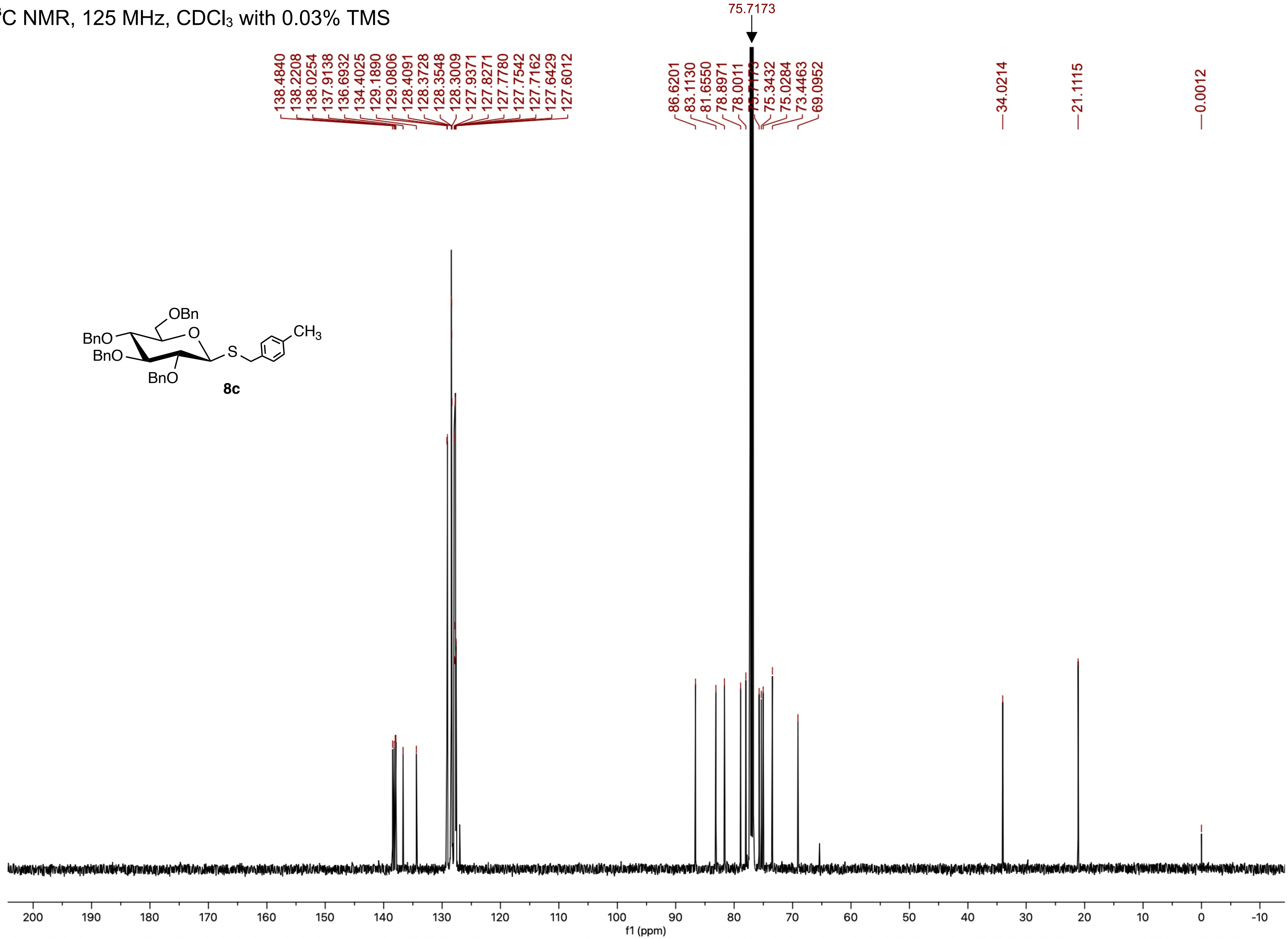

8db

^{1}H NMR, 400 MHz, $CDCl_3$ with 0.03% TMS

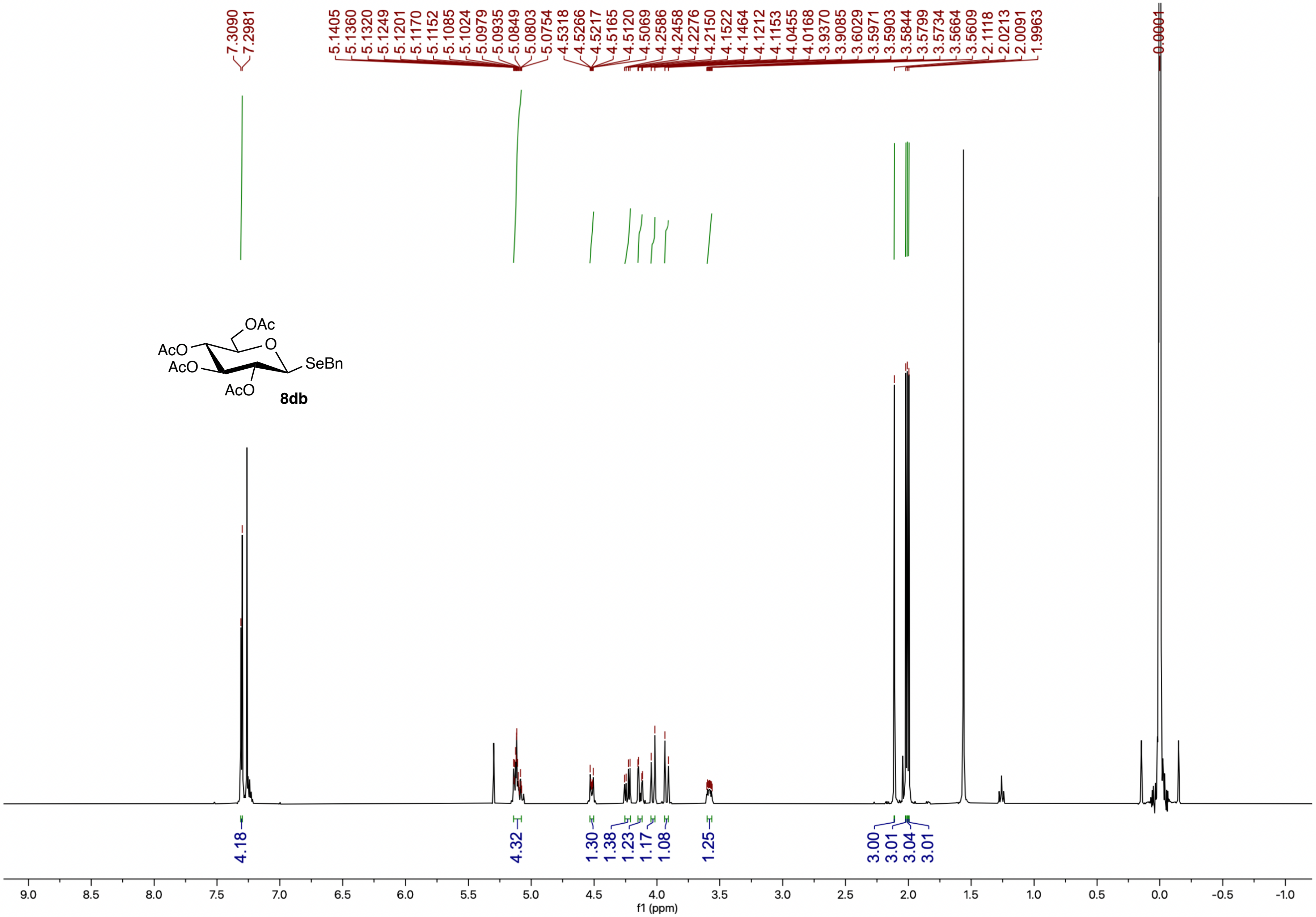

8dc

^{1}H NMR, 400 MHz, MeOD

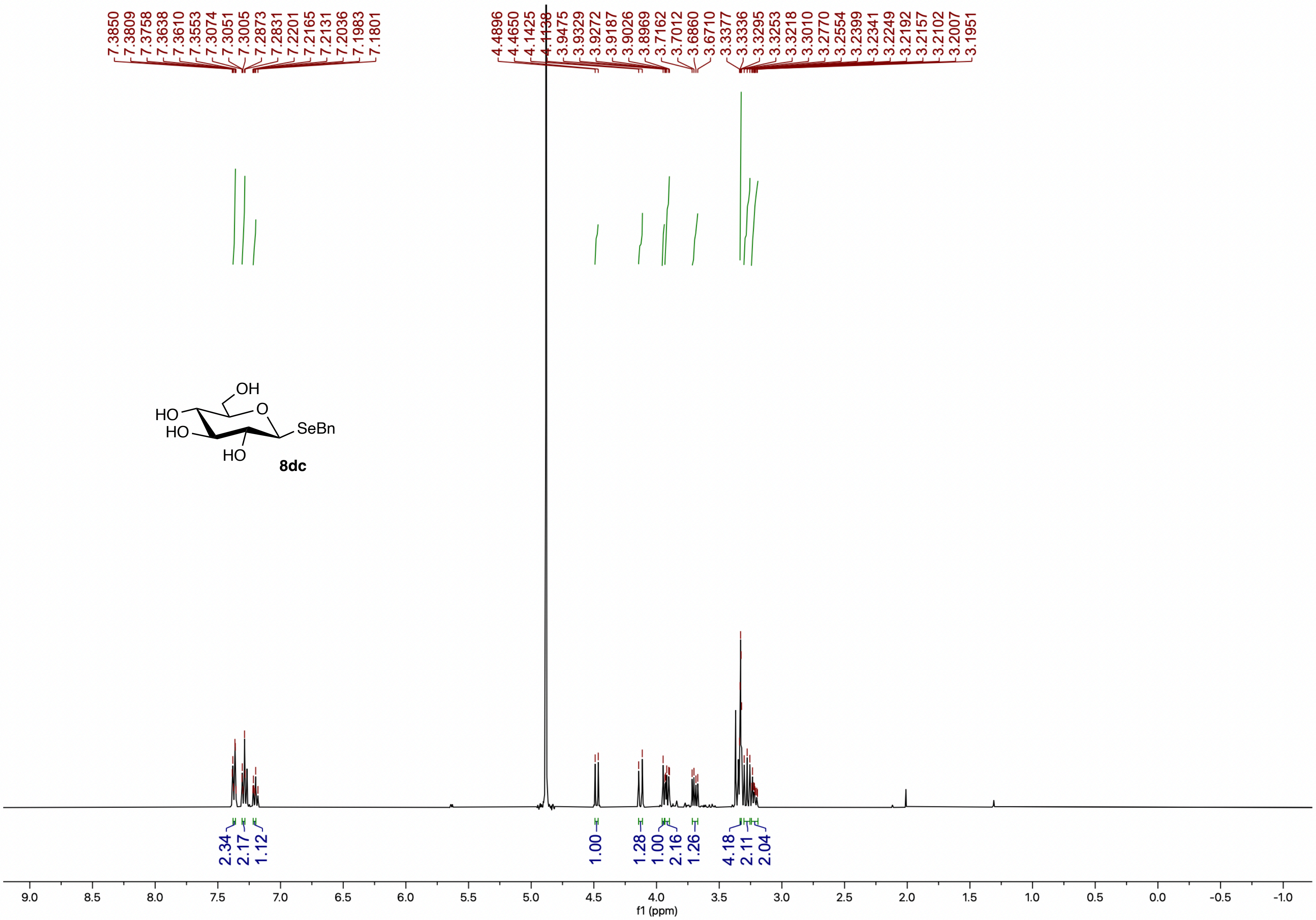

8d

^{1}H NMR, 400 MHz, $CDCl_3$ with 0.03% TMS

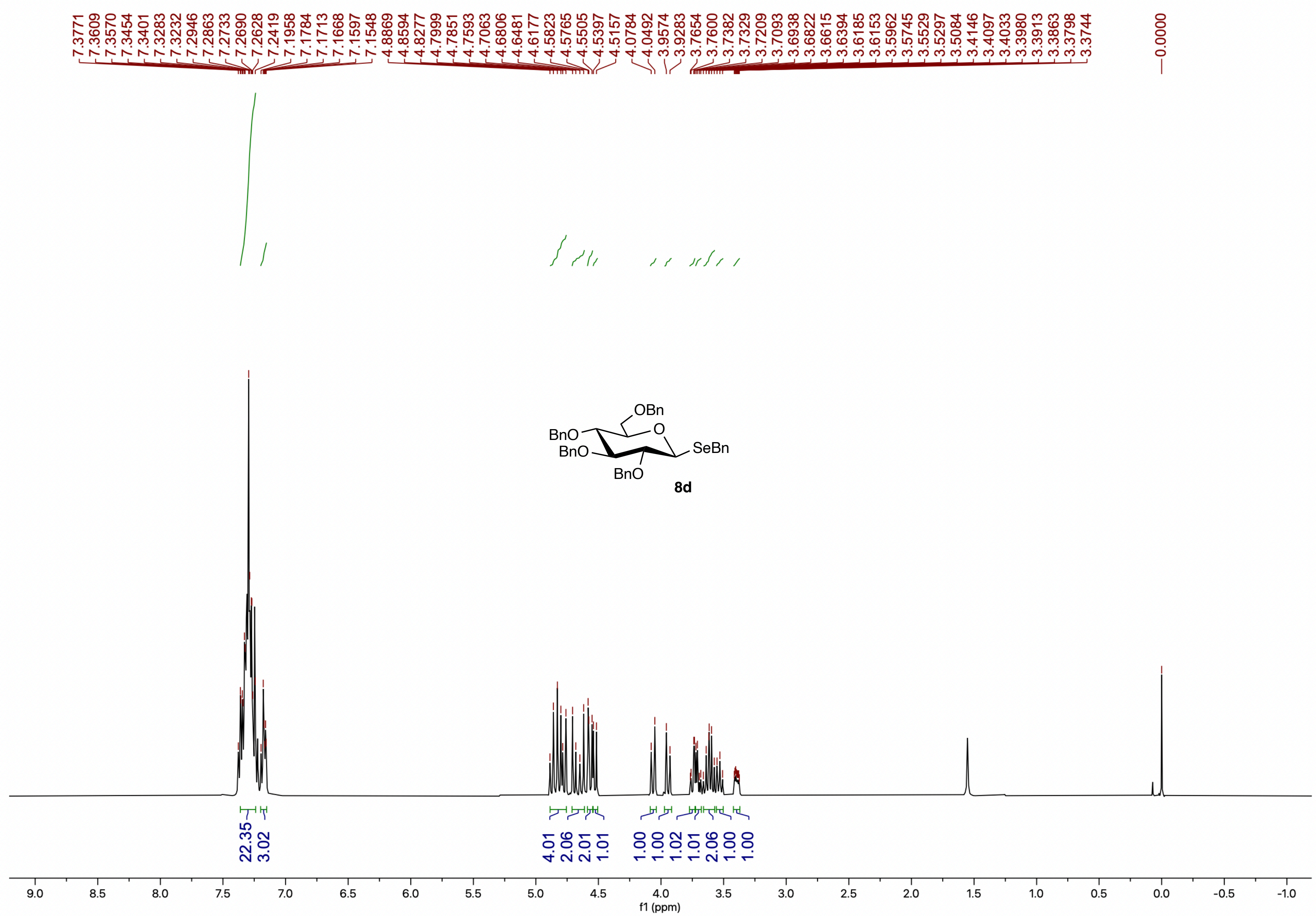

8ea

^{1}H NMR, 500 MHz, $CDCl_3$ with 0.03% TMS

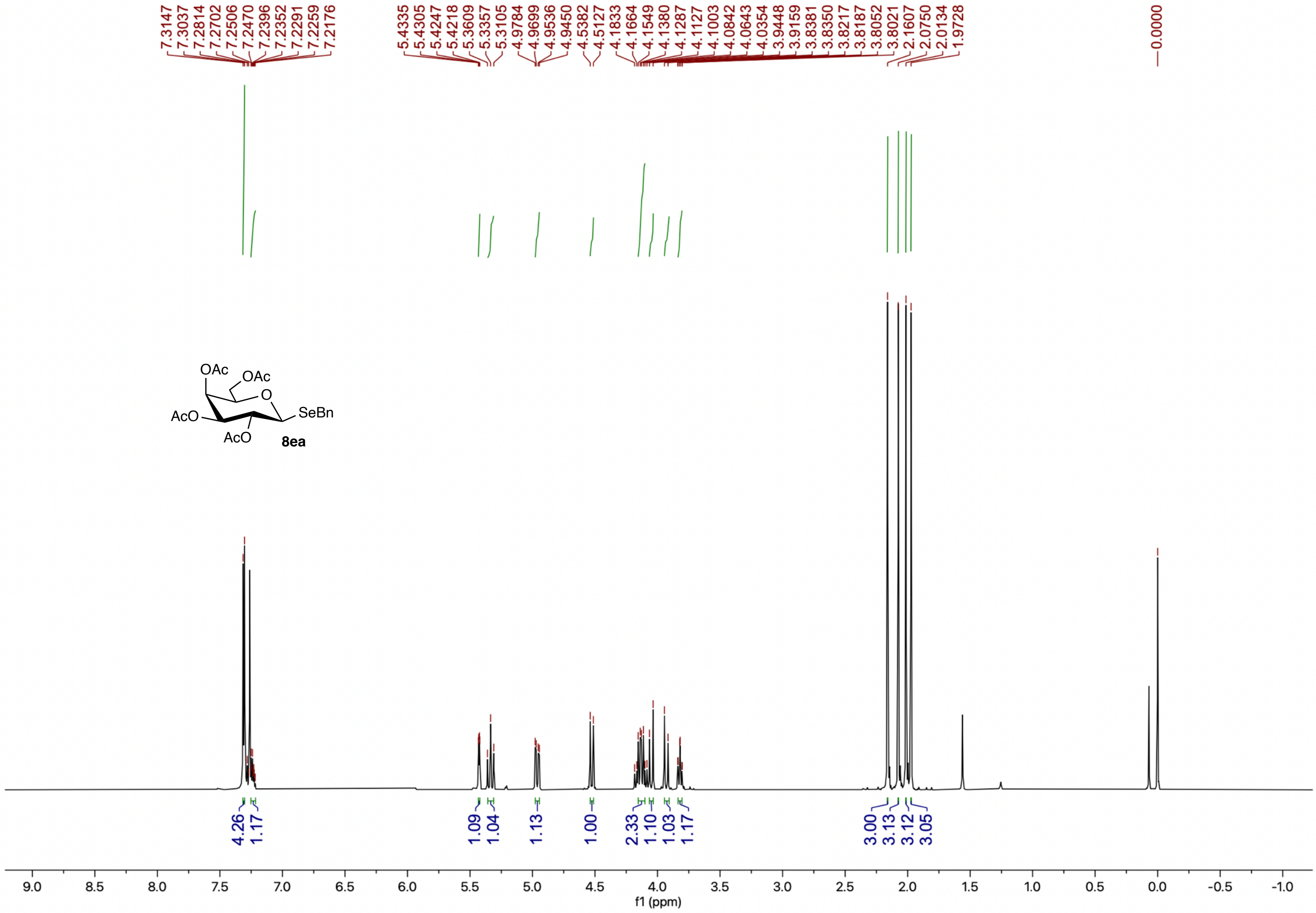

8ea

8ea
^{13}C NMR, 125 MHz, $CDCl_3$ with 0.03% TMS

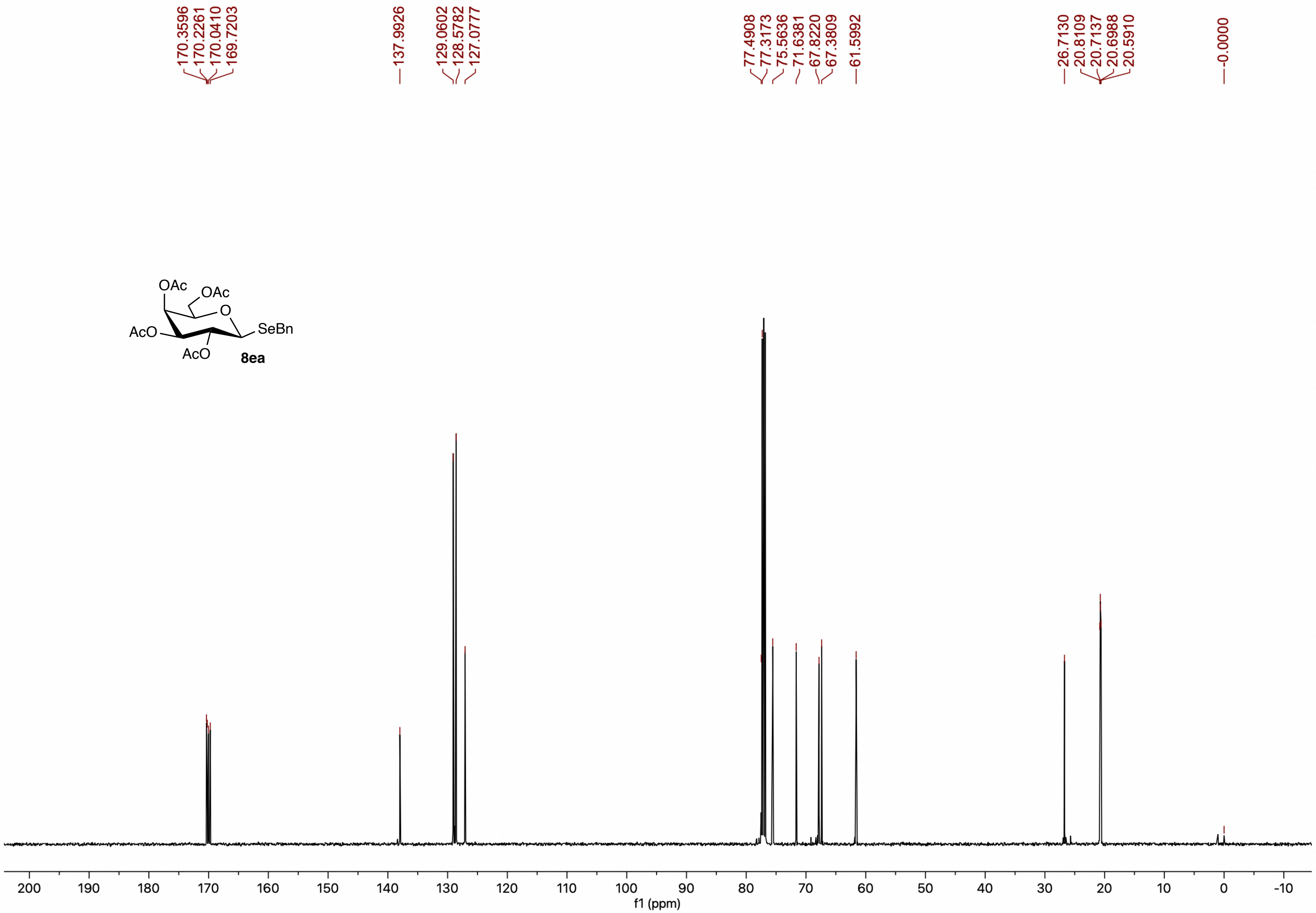

8e

^{1}H NMR, 400 MHz, $CDCl_3$ with 0.03% TMS

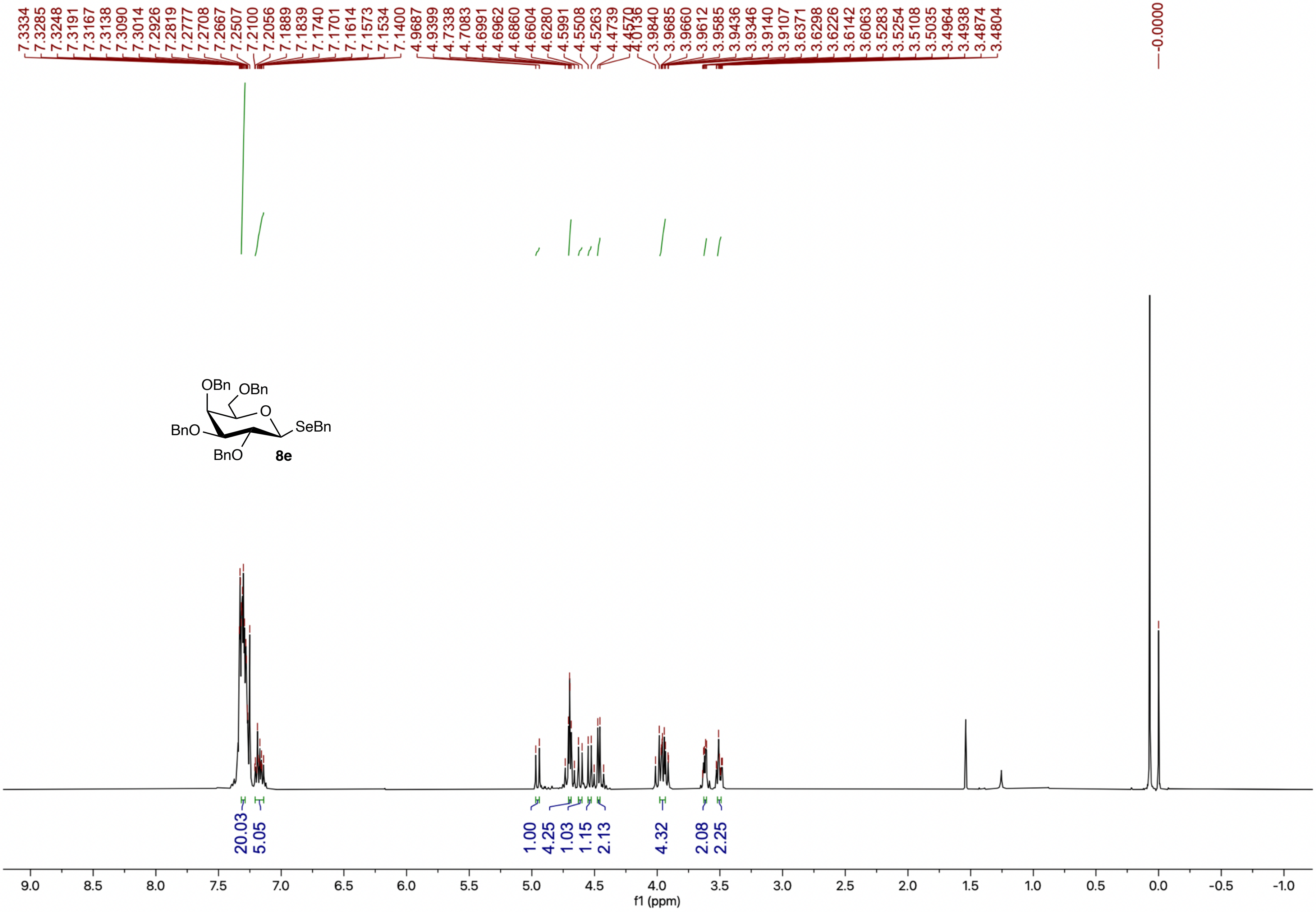

8e

^{13}C NMR, 125 MHz, $CDCl_3$ with 0.03% TMS

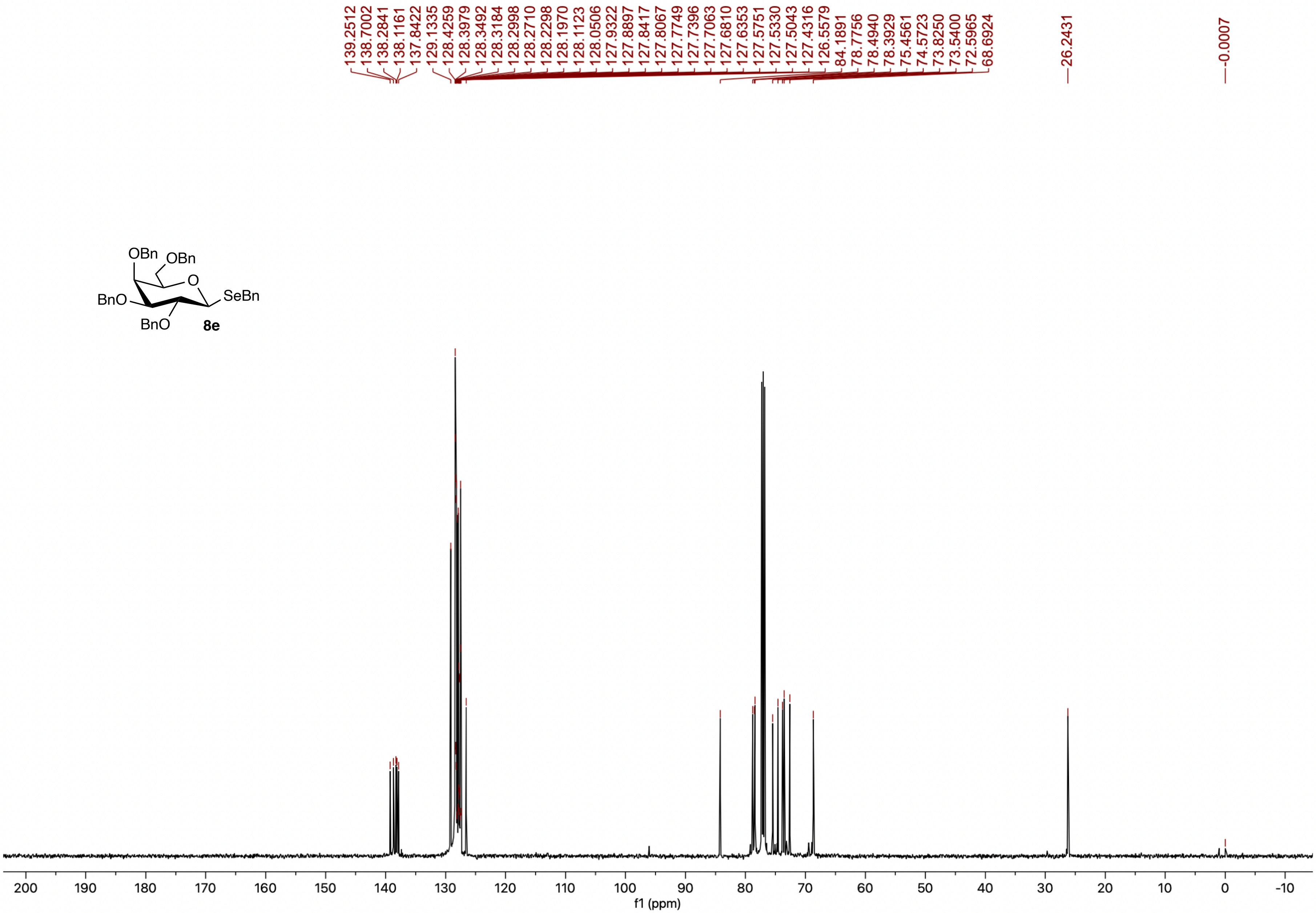

8fa
^{1}H NMR, 500 MHz, $CDCl_3$ with 0.03% TMS

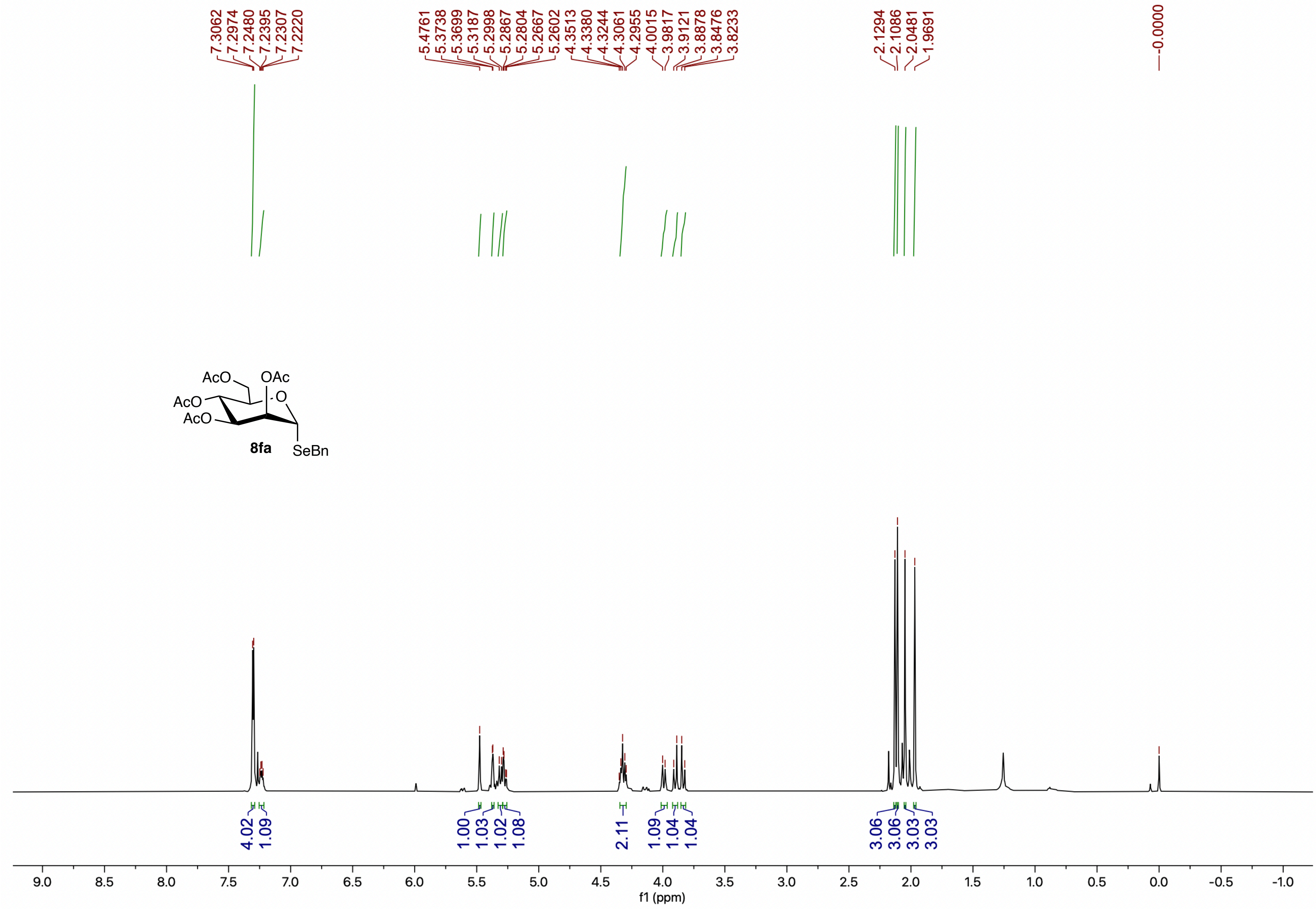

8fa

^{13}C NMR, 125 MHz, $CDCl_3$ with 0.03% TMS

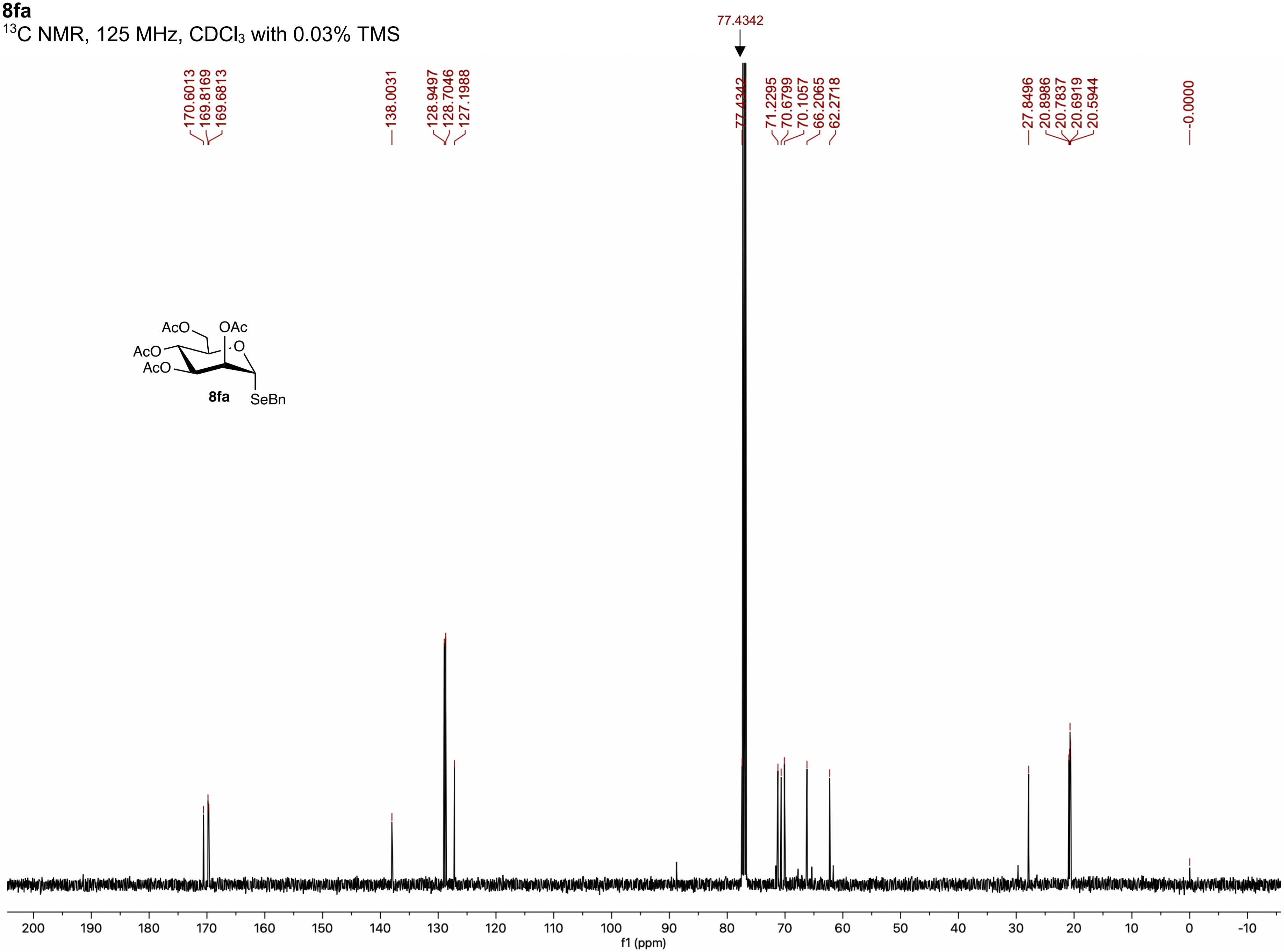

8f

^{1}H NMR, 500 MHz, $CDCl_3$ with 0.03% TMS

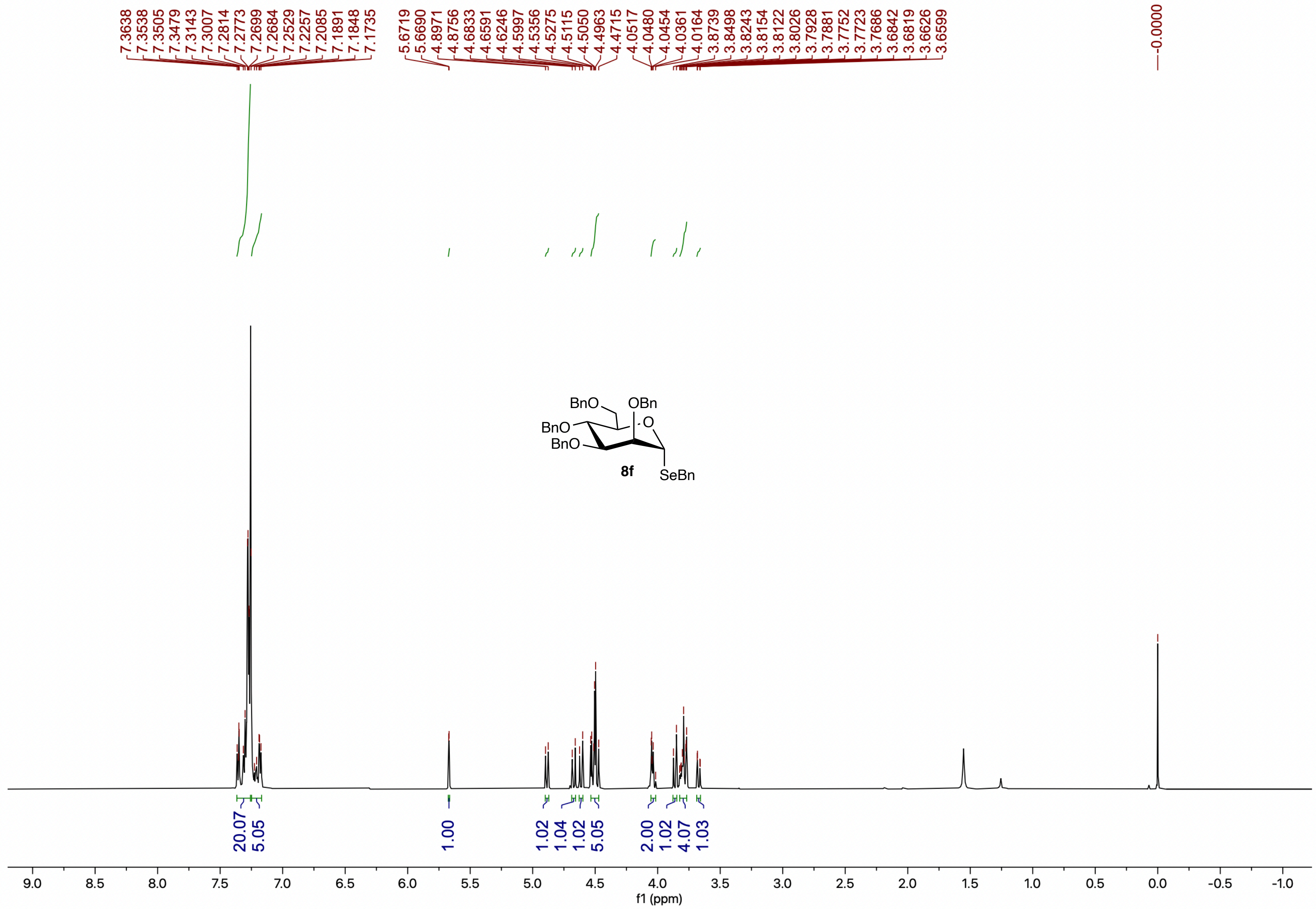

8f

^{13}C NMR, 125 MHz, $CDCl_3$ with 0.03% TMS

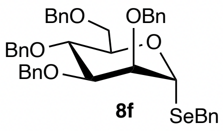

8f

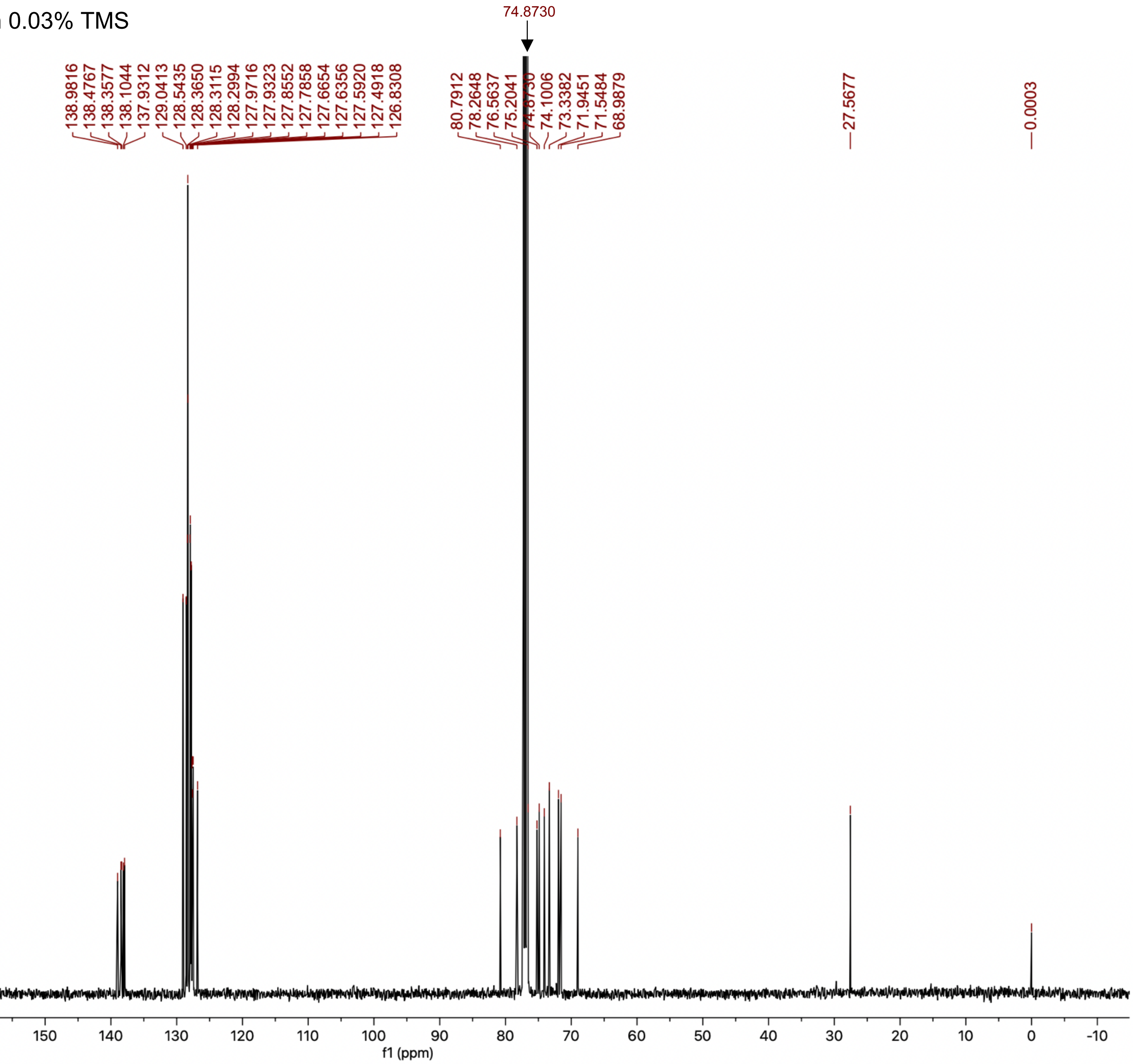

8ga
^{1}H NMR, 400 MHz, $CDCl_3$ with 0.03% TMS

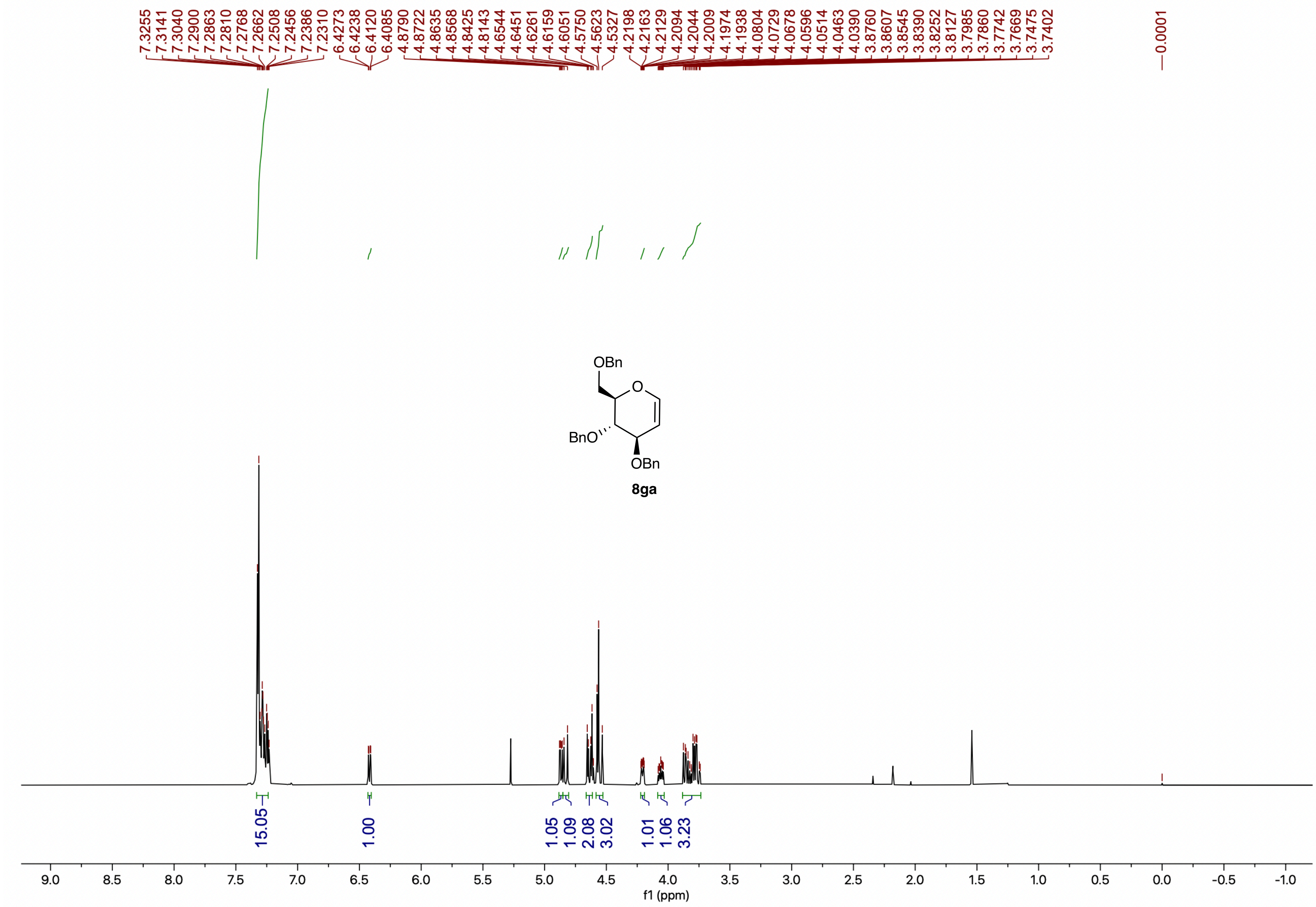

α-8g
^{1}H NMR, 500 MHz, $CDCl_3$ with 0.03% TMS

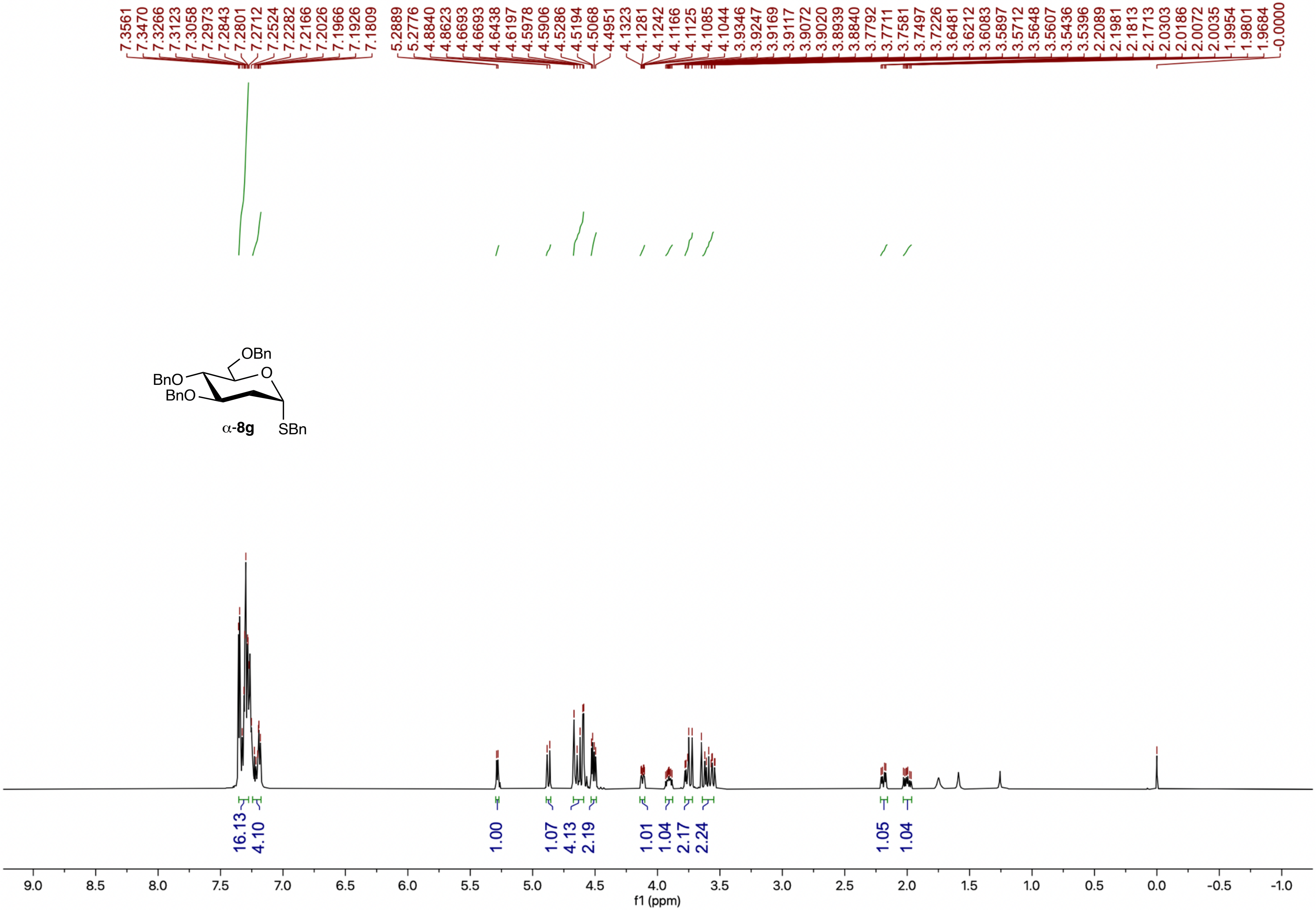

α-8g
^{13}C NMR, 125 MHz, $CDCl_3$ with 0.03% TMS

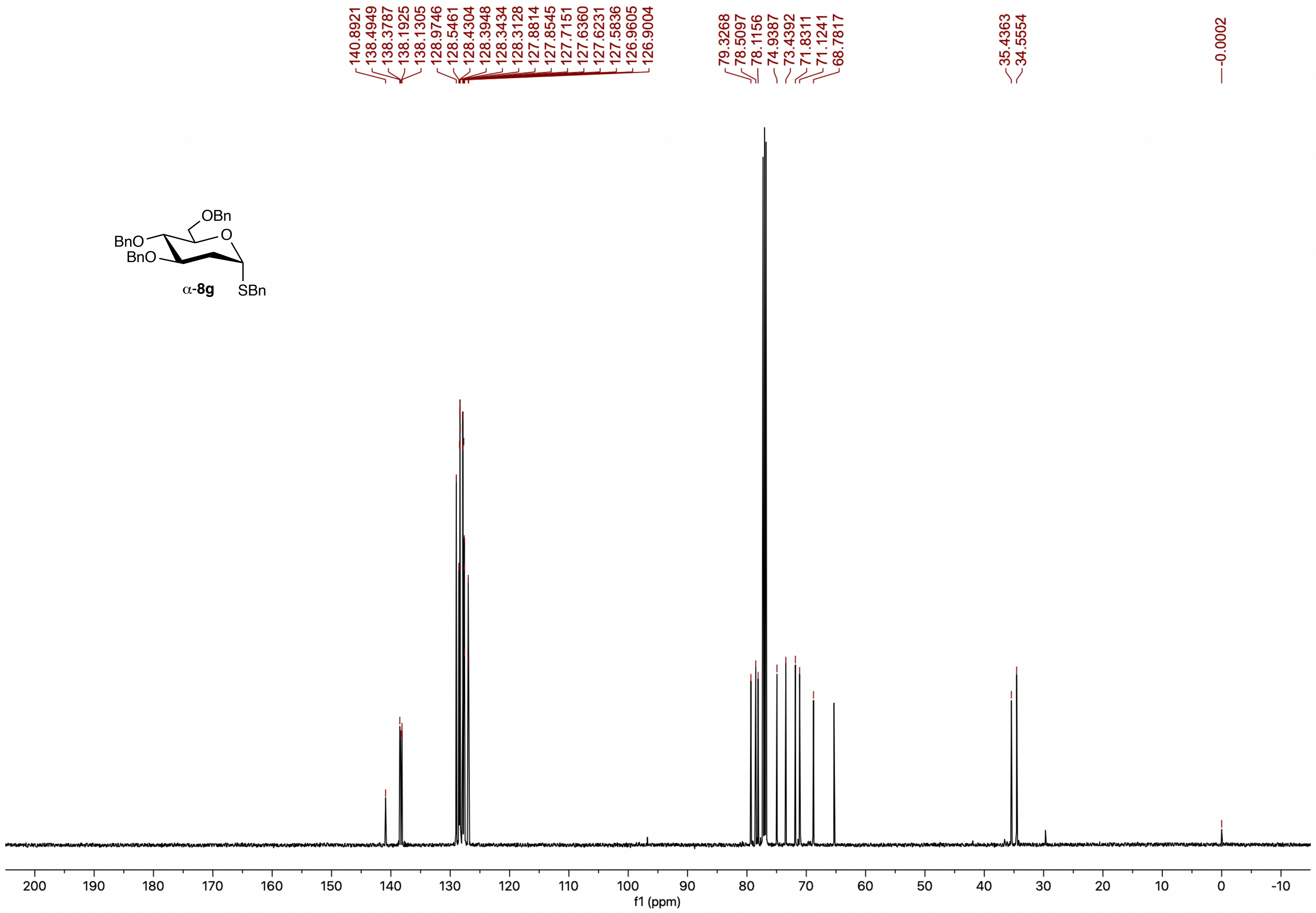

β-8g
^{1}H NMR, 500 MHz, $CDCl_3$ with 0.03% TMS

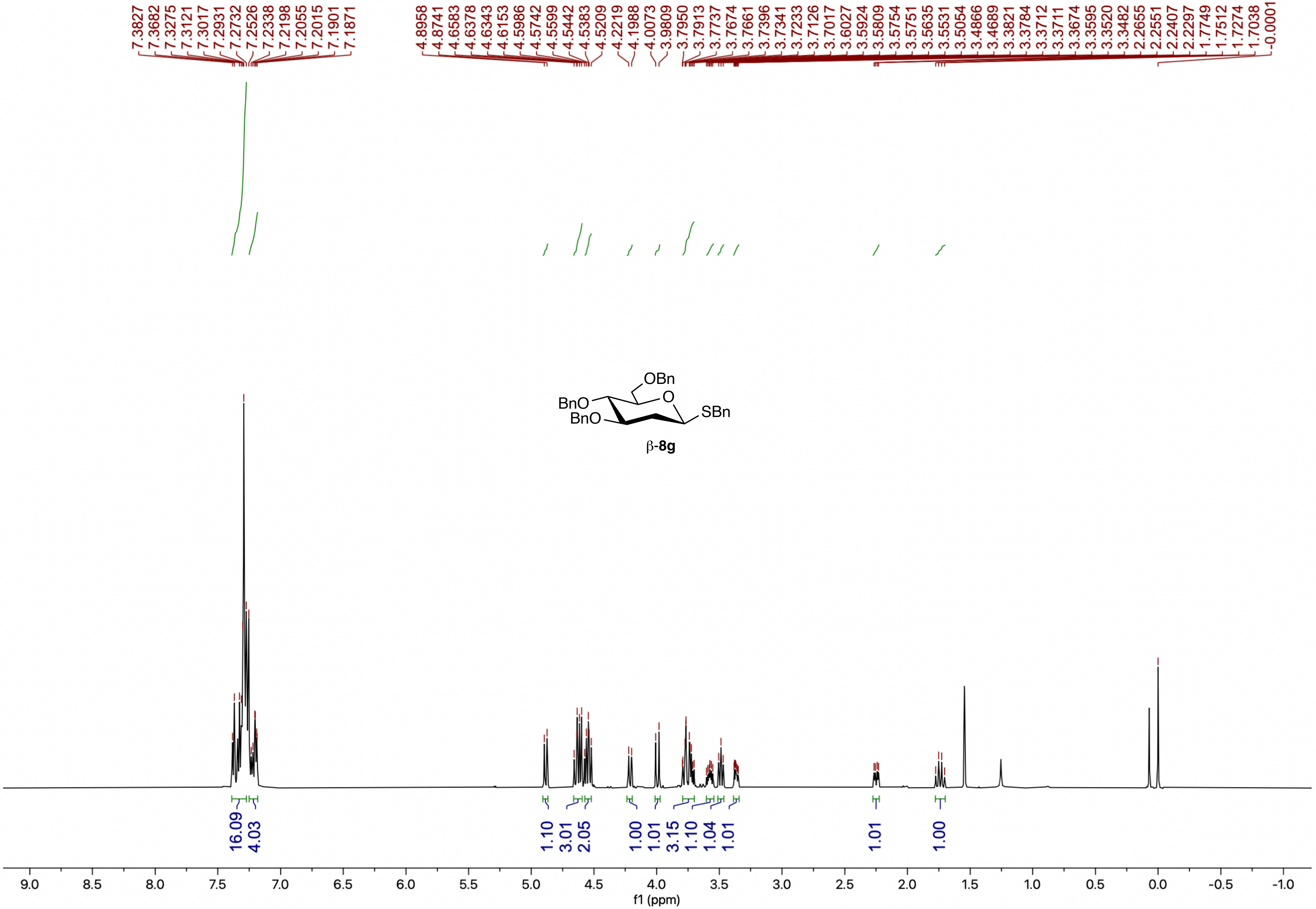

β-8g
^{13}C NMR, 125 MHz, $CDCl_3$ with 0.03% TMS

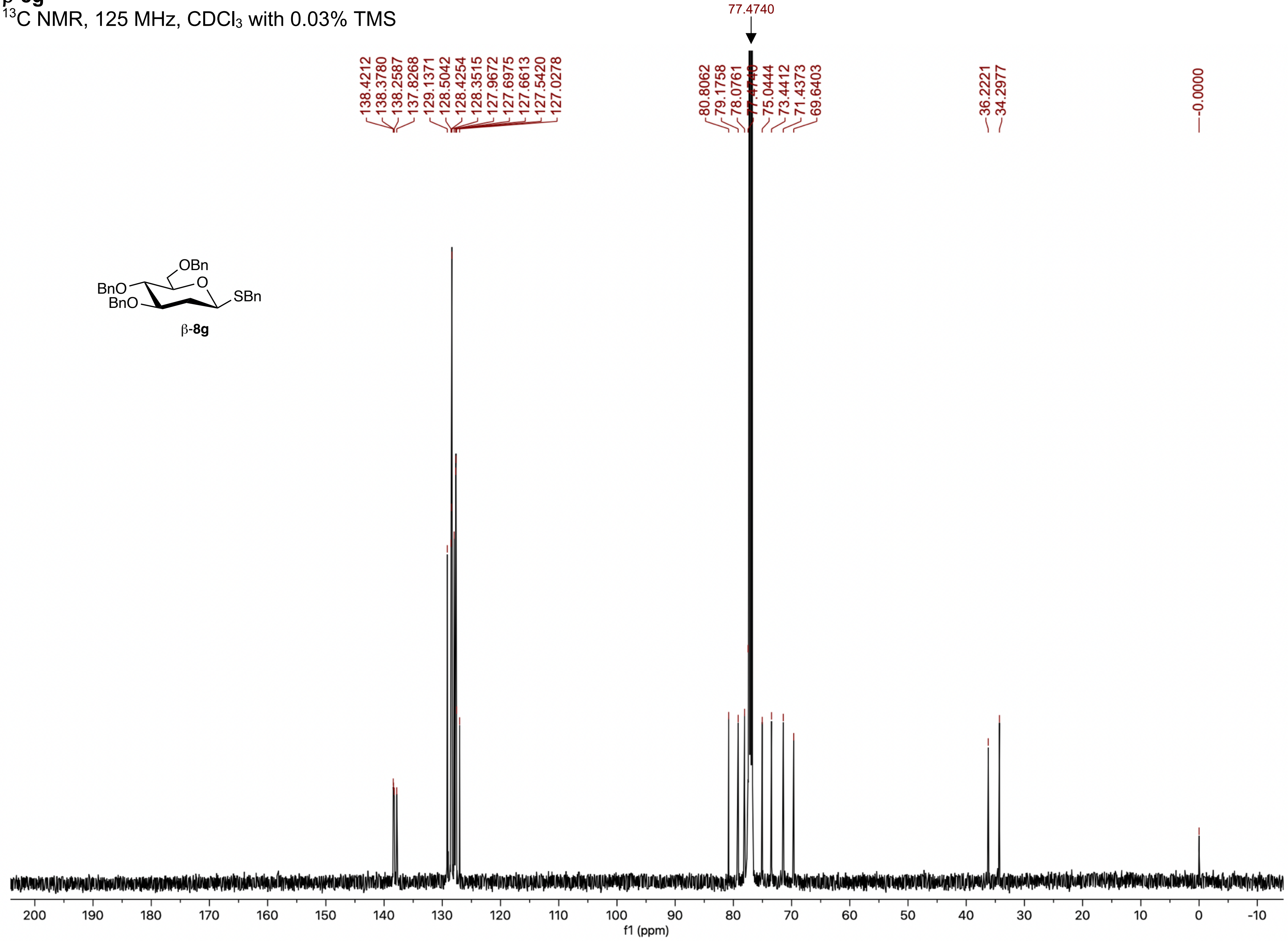

8ha
^1H NMR, 400 MHz, $CDCl_3$ with 0.03% TMS

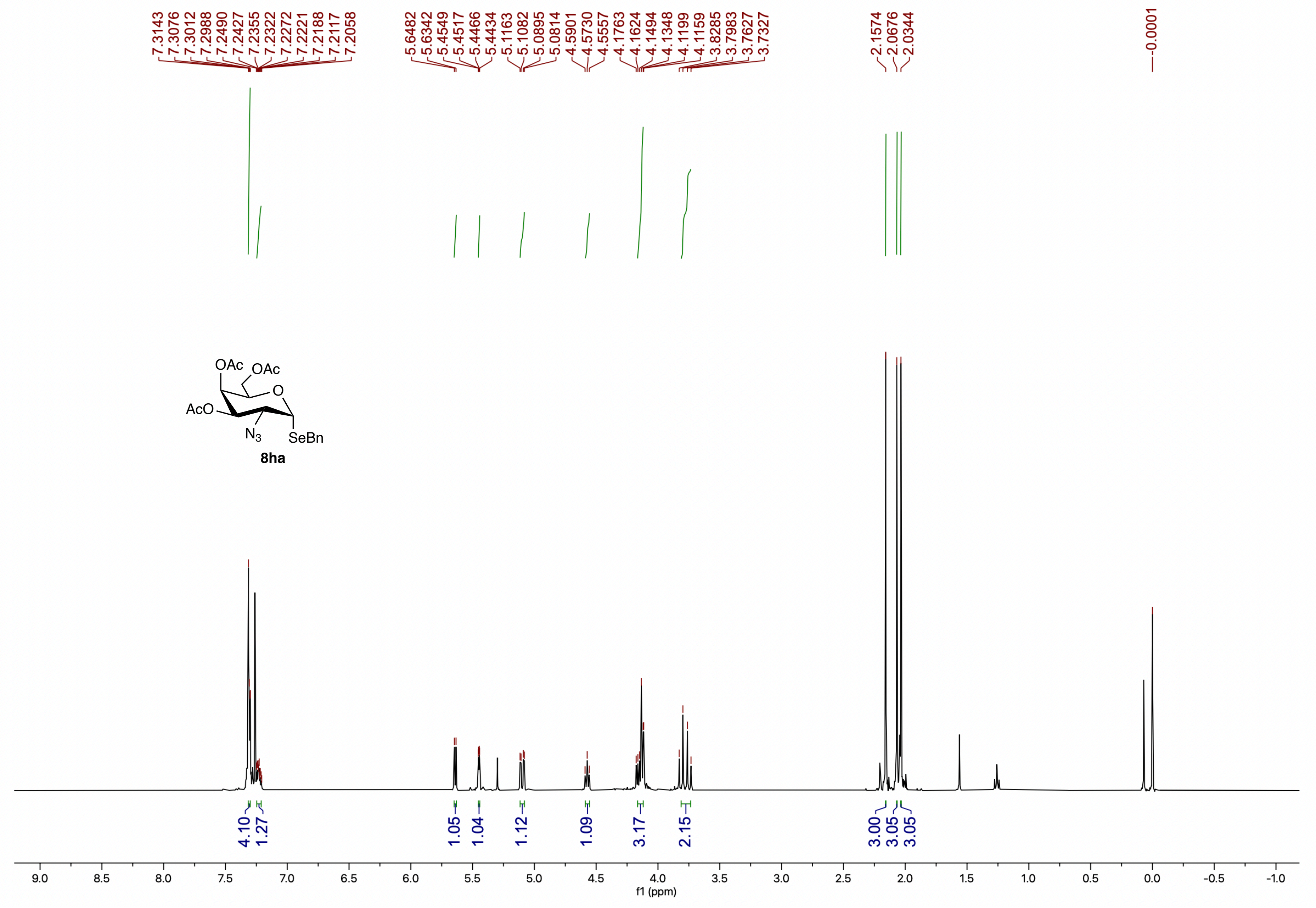

8ha
^{13}C NMR, 125 MHz, $CDCl_3$ with 0.03% TMS

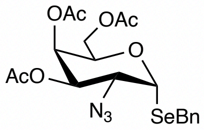

8ha

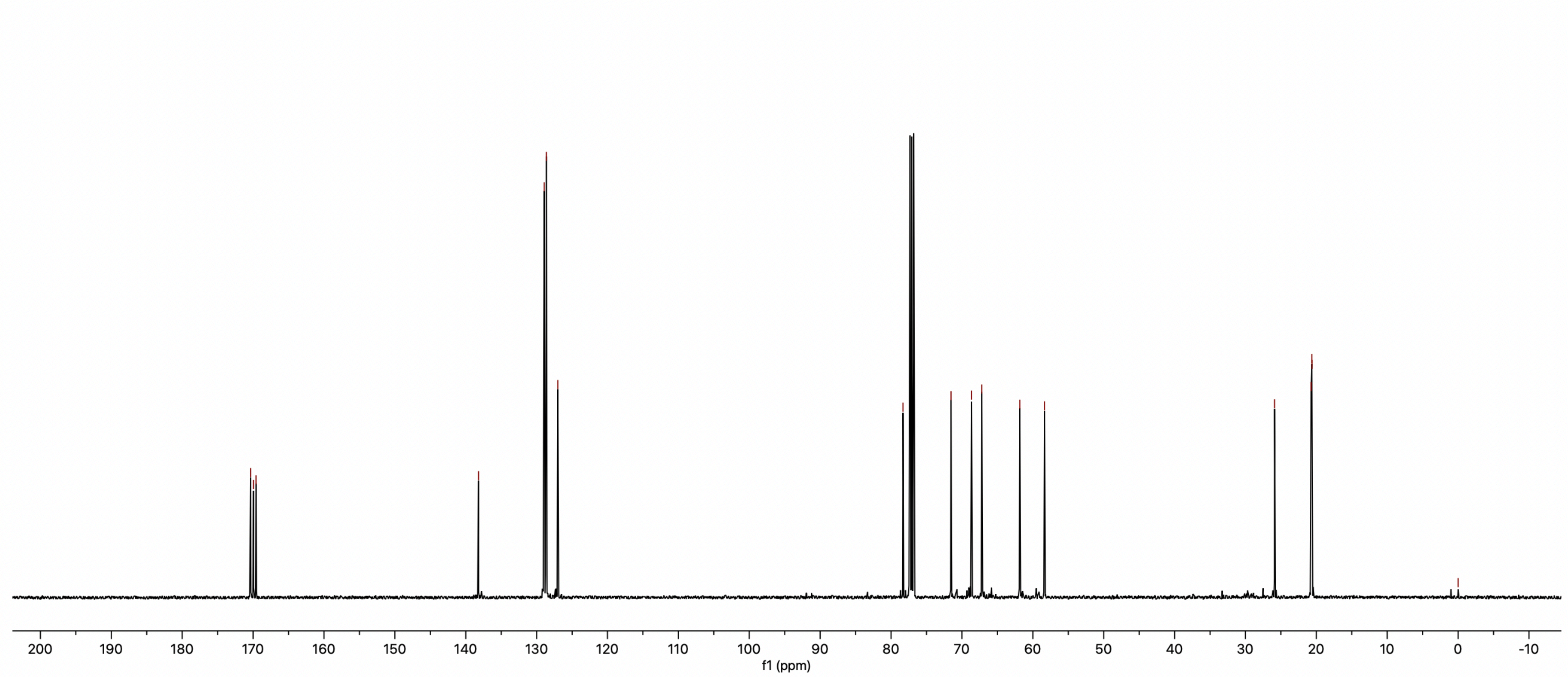

8h

^{1}H NMR, 400 MHz, $CDCl_3$ with 0.03% TMS

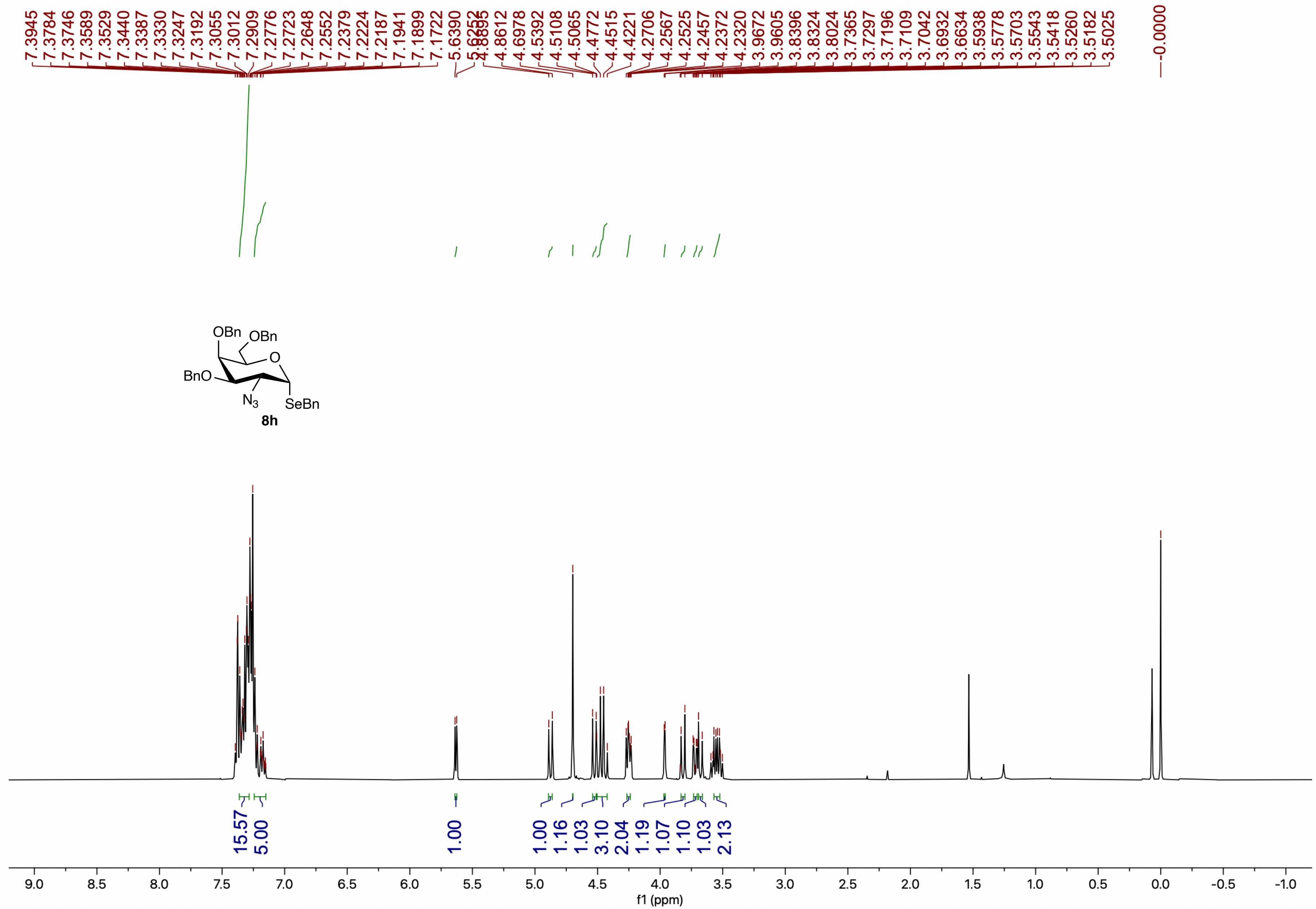

8h

8h

^{13}C NMR, 125 MHz, $CDCl_3$ with 0.03% TMS

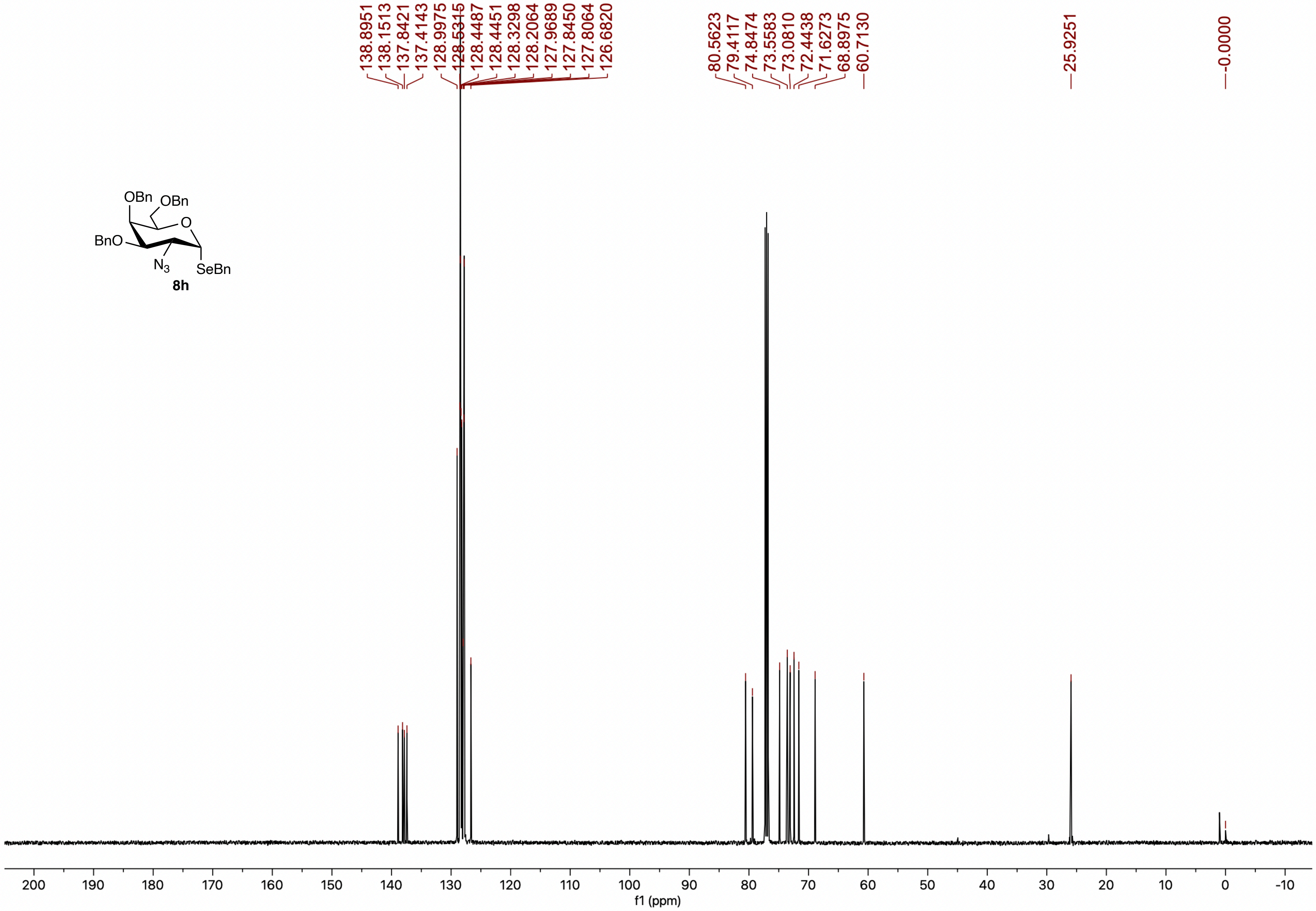

12

^{1}H NMR, 400 MHz, $CDCl_3$ with0.03% TMS

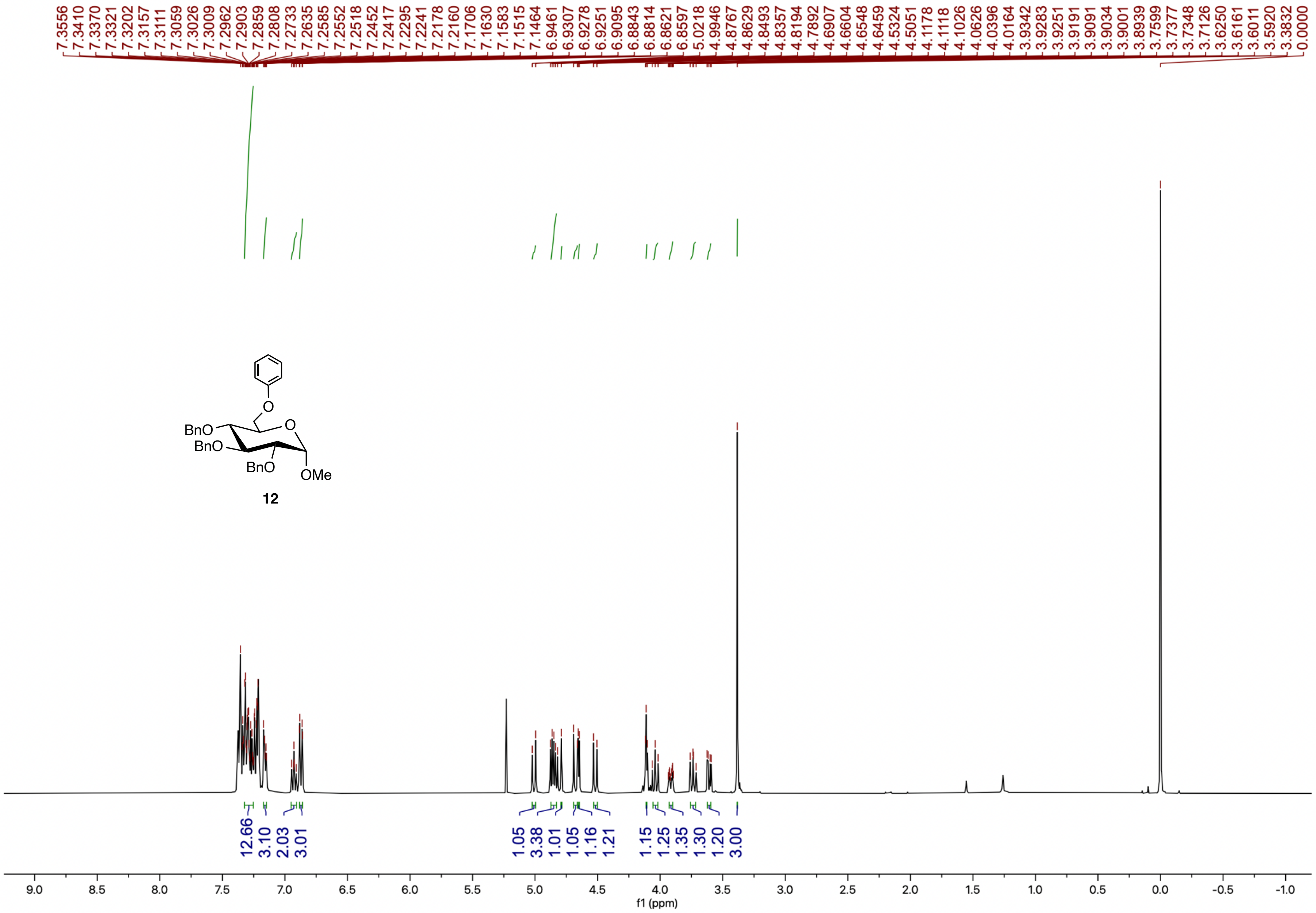

12

^{13}C NMR, 125 MHz, $CDCl_3$ with 0.03% TMS

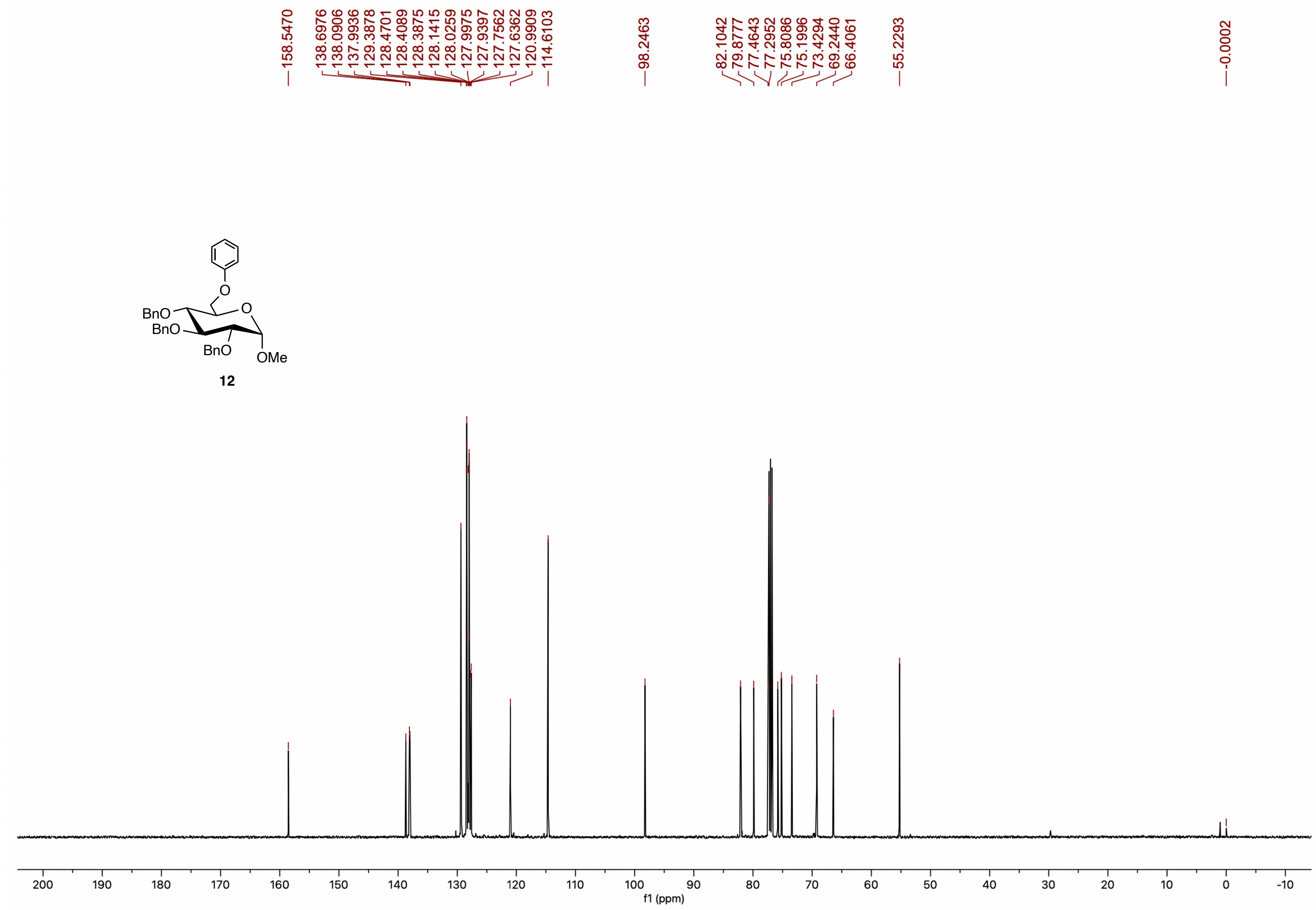

^{1}H NMR Estimation of Anomeric Ratios for the Optimization Study

Scheme 2, Entry 1, purified

^{1}H NMR, 400 MHz, $CDCl_3$ with 0.03% TMS

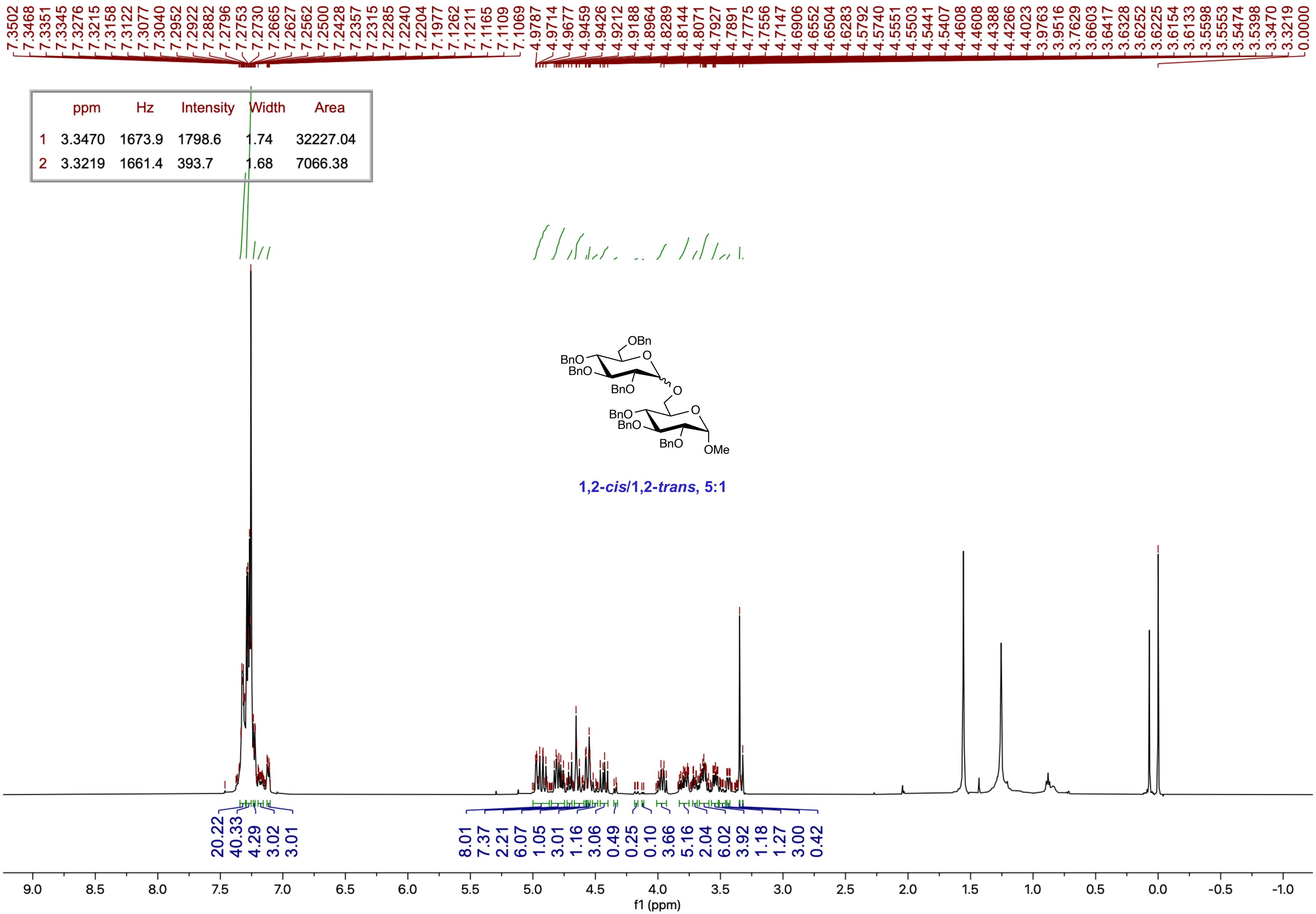

Scheme 2, Entry 2, purified

^1H NMR, 400 MHz, $CDCl_3$ with 0.03% TMS

	ppm	Hz	Intensity	Width	Area
1	3.3469	1339.2	2846.9	2.32	85524.71
2	3.3221	1329.3	483.0	2.45	16507.52

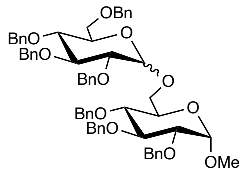

1,2-*cis*/1,2-*trans*, 5:1

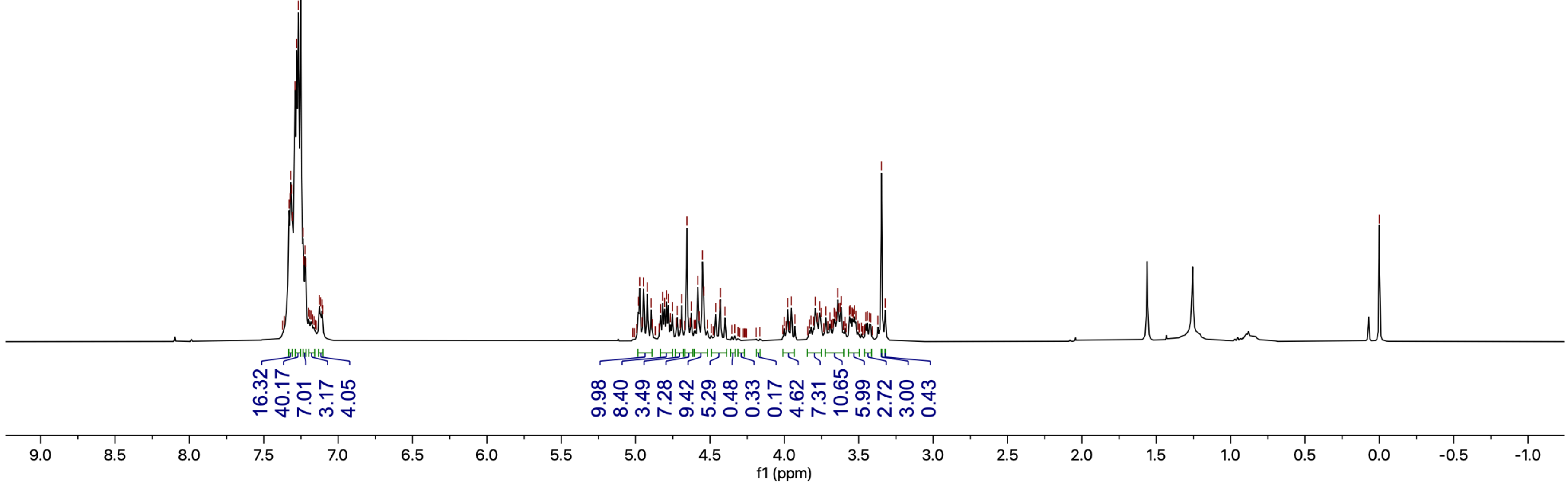

Scheme 2, Entry 4, purified

^{1}H NMR, 400 MHz, $CDCl_3$ with 0.03% TMS

	ppm	Hz	Intensity	Width	Area
1	3.3472	1674.0	376.0	2.14	8772.86
2	3.3223	1661.6	3460.5	2.14	72910.29

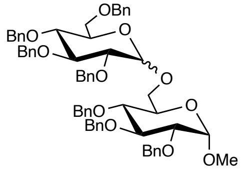

1,2-*cis*/1,2-*trans*, 1:8

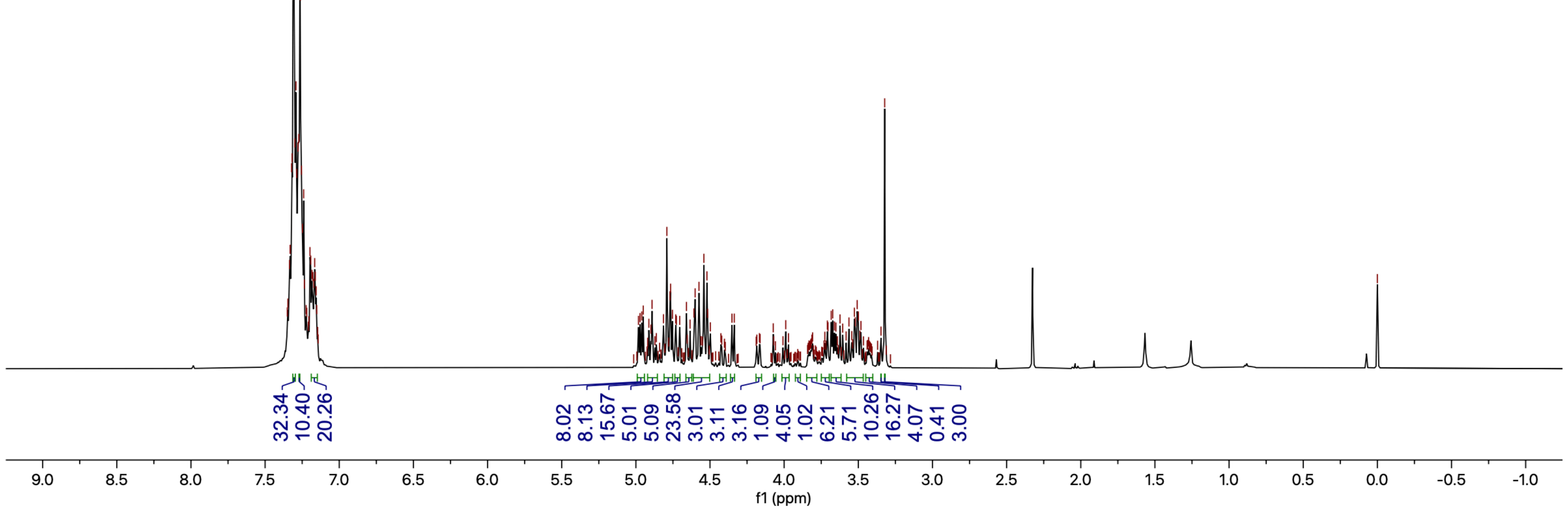

Scheme 2, Entry 5, purified

^{1}H NMR, 400 MHz, $CDCl_3$ with 0.03% TMS

	ppm	Hz	Intensity	Width	Area
1	3.3474	1339.4	4182.7	1.58	81085.30
2	3.3224	1329.4	802.1	1.53	16000.27

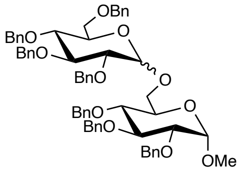

1,2-*cis*/1,2-*trans*, 5:1

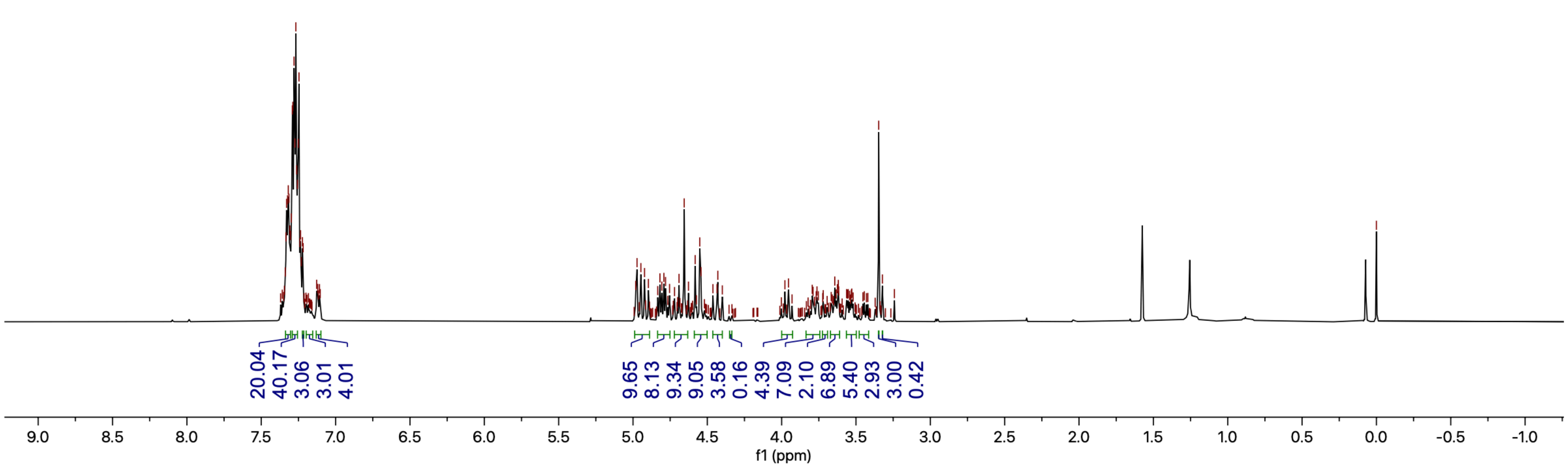

Scheme 2, Entry 6, purified

^{1}H NMR, 400 MHz, $CDCl_3$ with 0.03% TMS

	ppm	Hz	Intensity	Width	Area
1	3.3480	1674.4	521.6	5.01	22312.75
2	3.3233	1662.1	117.6	5.26	5163.63

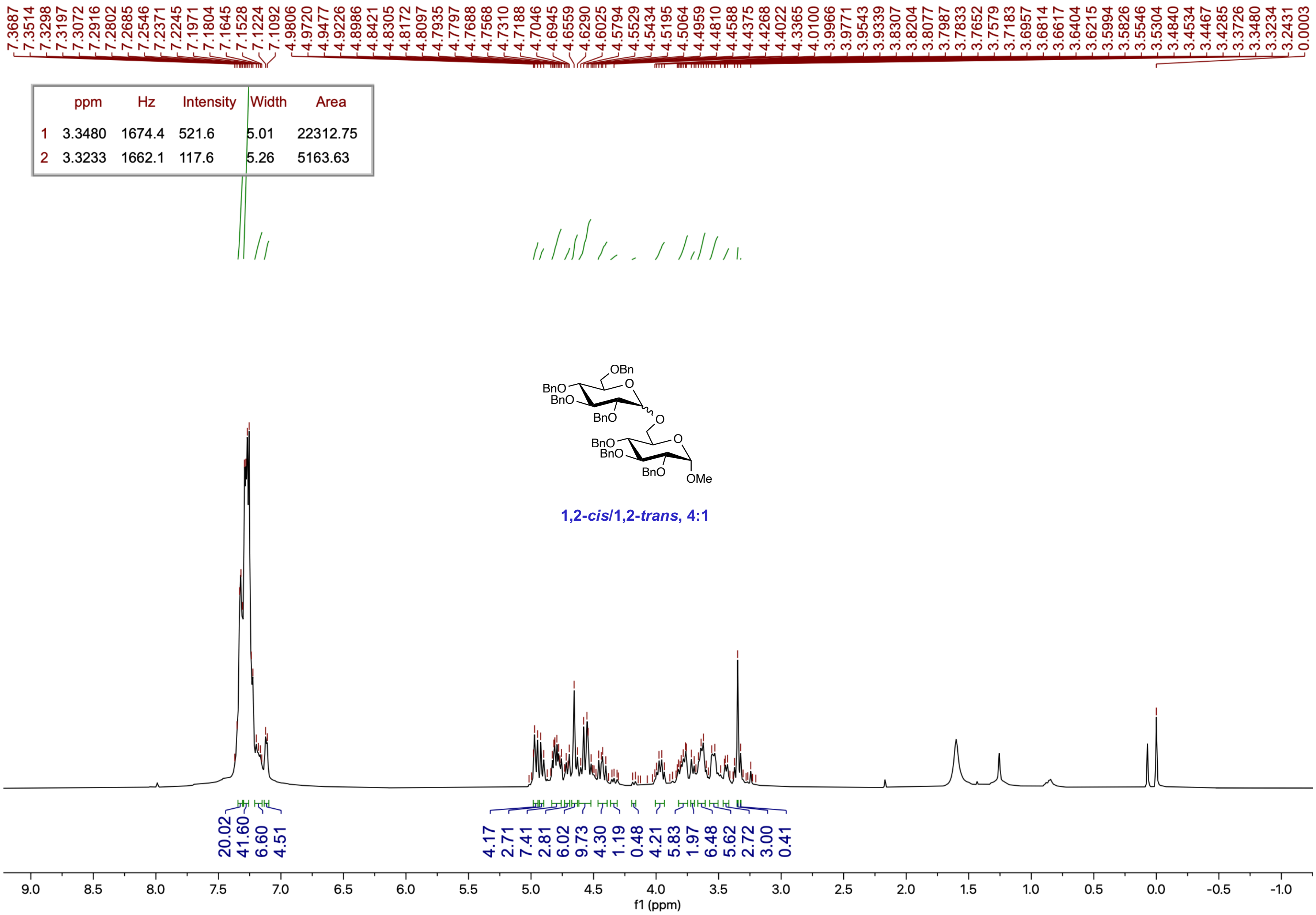

Scheme 2, Entry 7, purified

^{1}H NMR, 400 MHz, $CDCl_3$ with 0.03% TMS

	ppm	Hz	Intensity	Width	Area
1	3.3484	1339.8	4462.2	1.94	102851.89
2	3.3238	1330.0	276.7	1.75	6038.42

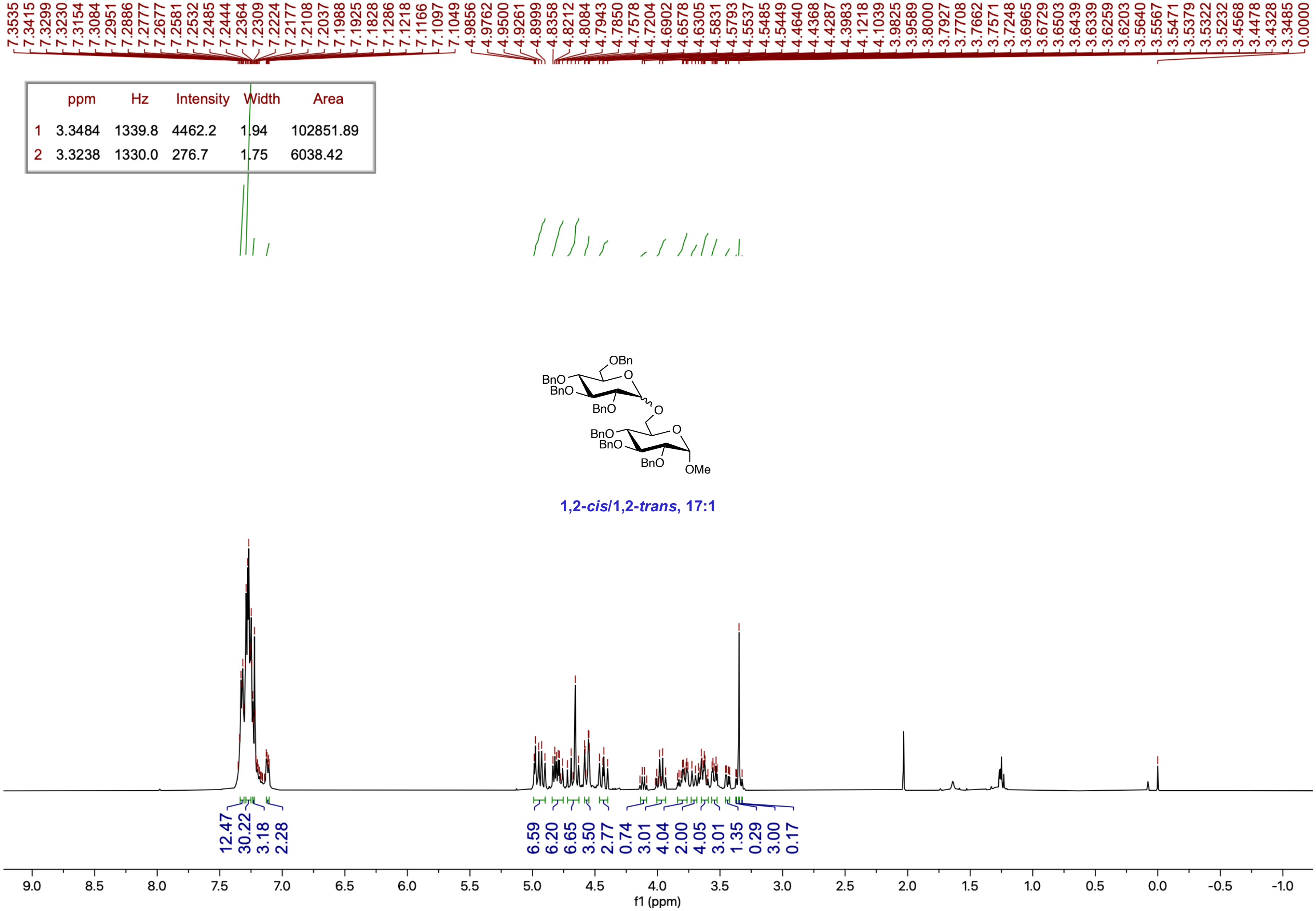

1,2-*cis*/1,2-*trans*, 17:1

Scheme 2, Entry 8, purified

^{1}H NMR, 400 MHz, $CDCl_3$ with 0.03% TMS

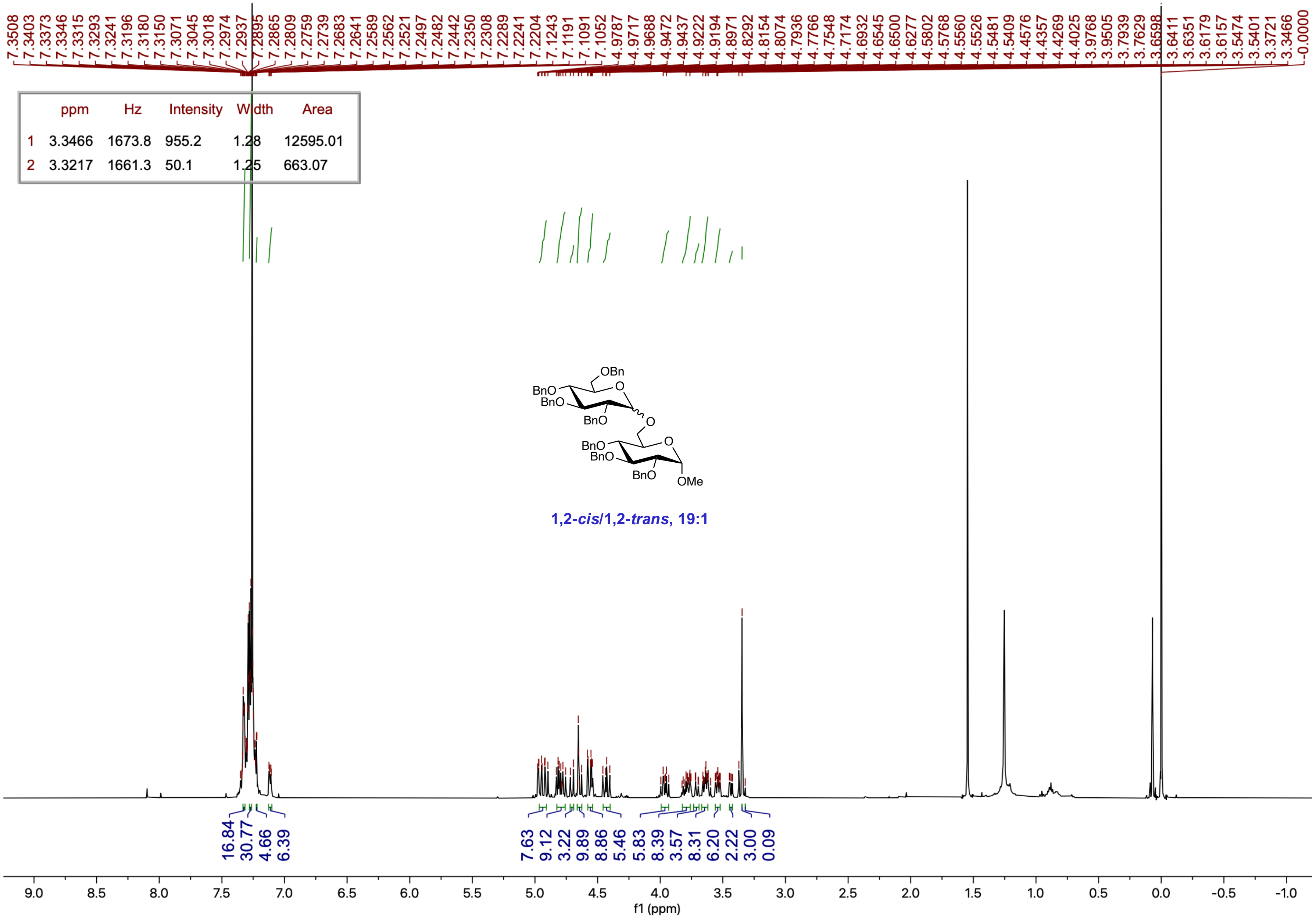

	ppm	Hz	Intensity	Width	Area
1	3.3466	1673.8	955.2	1.28	12595.01
2	3.3217	1661.3	50.1	1.25	663.07

Scheme 2, Entry 9, purified

^{1}H NMR, 400 MHz, $CDCl_3$ with 0.03% TMS

	ppm	Hz	Intensity	Width	Area
1	3.3481	1674.5	2485.9	3.28	78213.53
2	3.3234	1662.1	207.6	3.82	8030.95

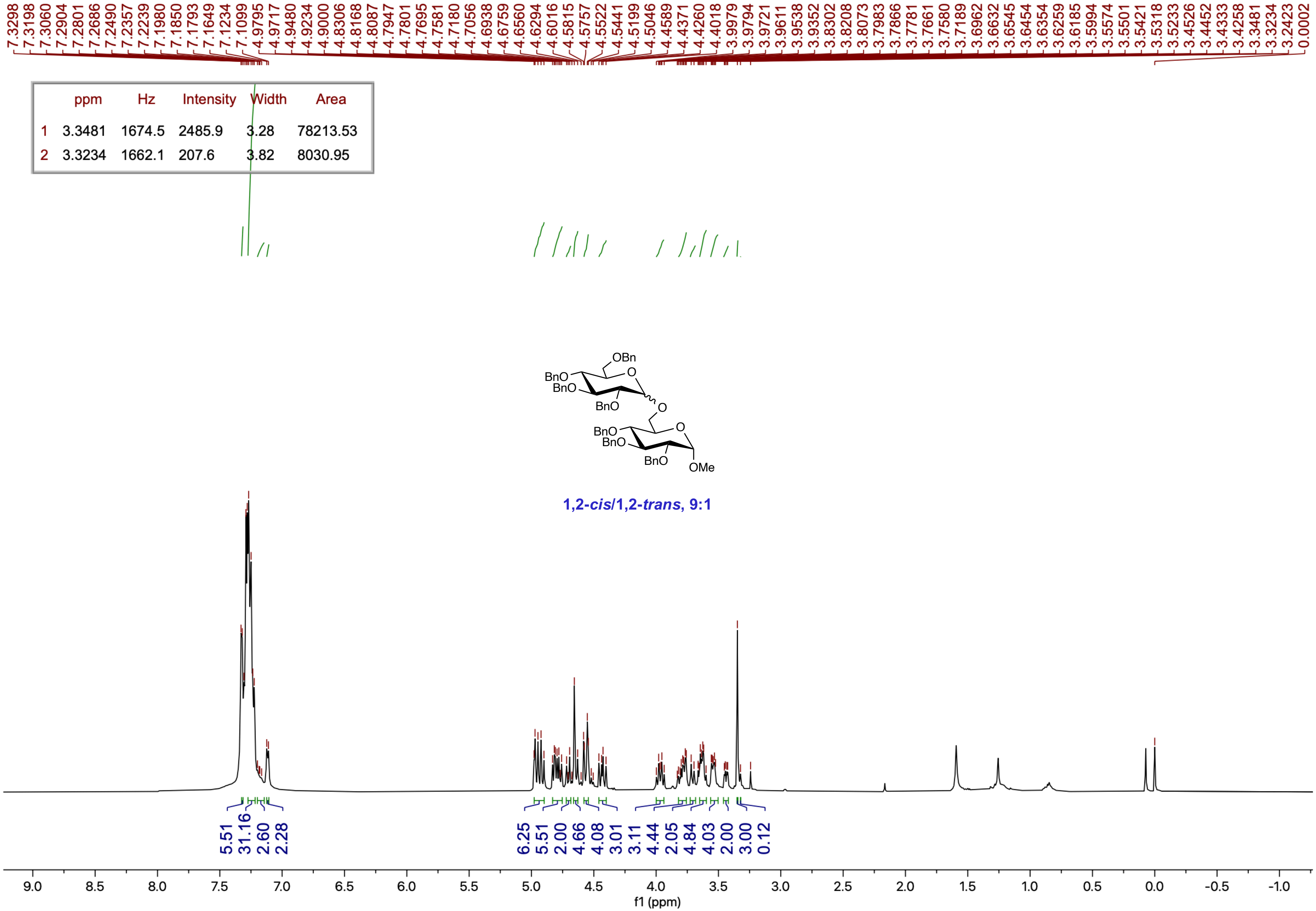

1,2-*cis*/1,2-*trans*, 9:1

Scheme 2, Entry 10, purified

^{1}H NMR, 400 MHz, $CDCl_3$ with 0.03% TMS

	ppm	Hz	Intensity	Width	Area
1	3.3468	1339.1	2132.1	1.32	35275.50
2	3.3218	1329.1	140.2	1.22	2148.21

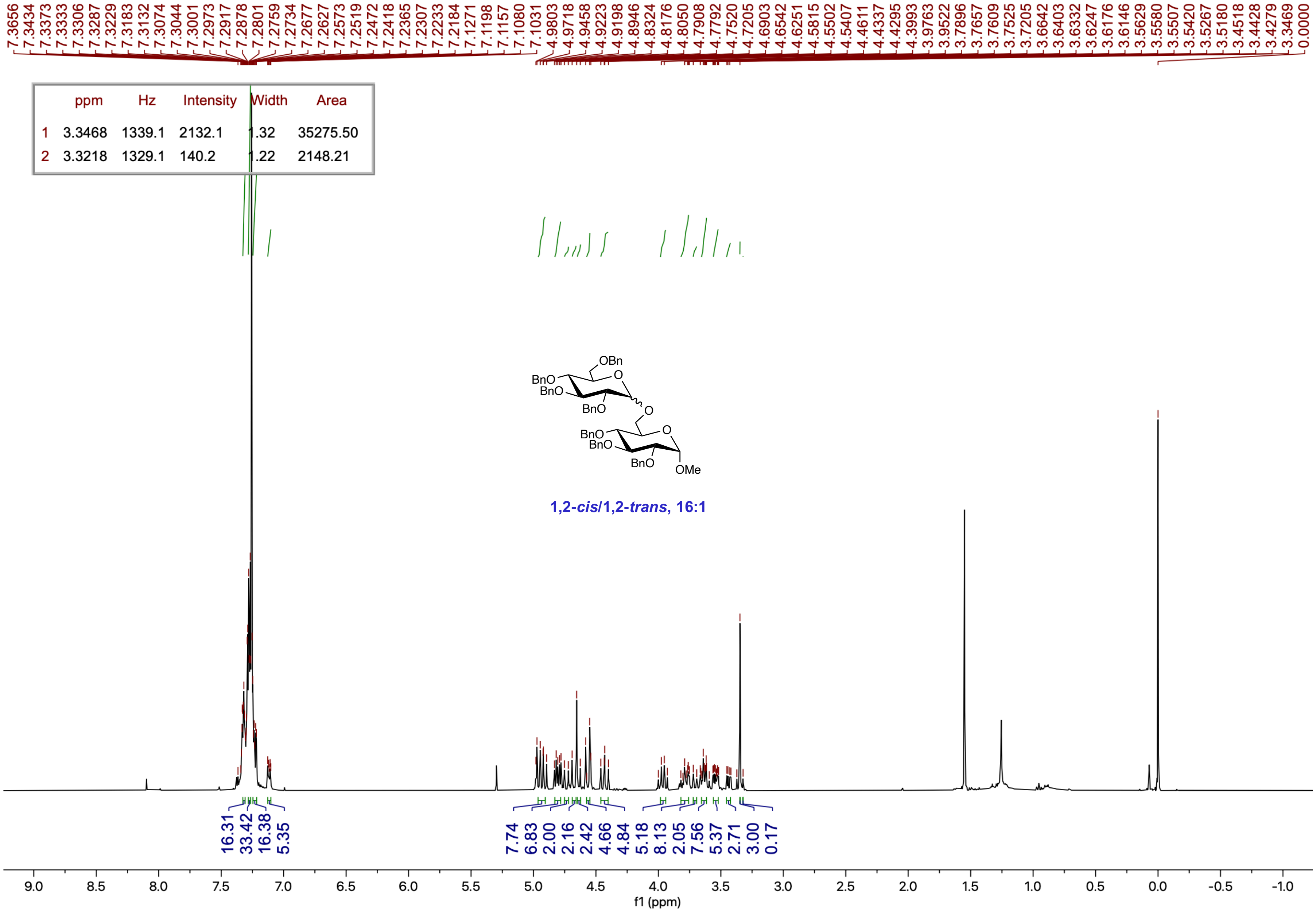

1,2-*cis*/1,2-*trans*, 16:1

Scheme 2, Entry 12, purified

^{1}H NMR, 400 MHz, $CDCl_3$ with 0.03% TMS

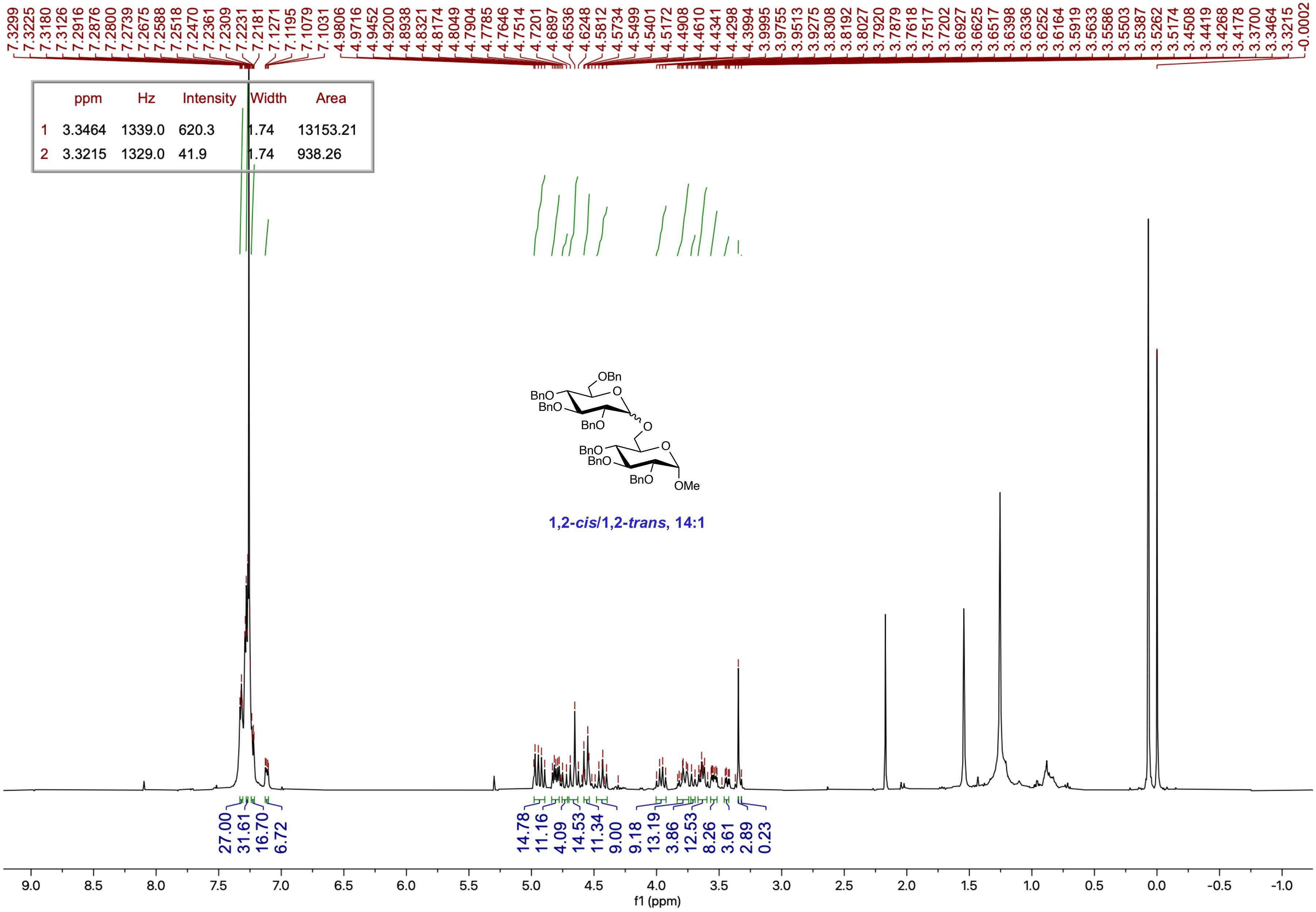

Scheme 2, Entry 13, purified
^{1}H NMR, 400 MHz, $CDCl_3$ with 0.03% TMS

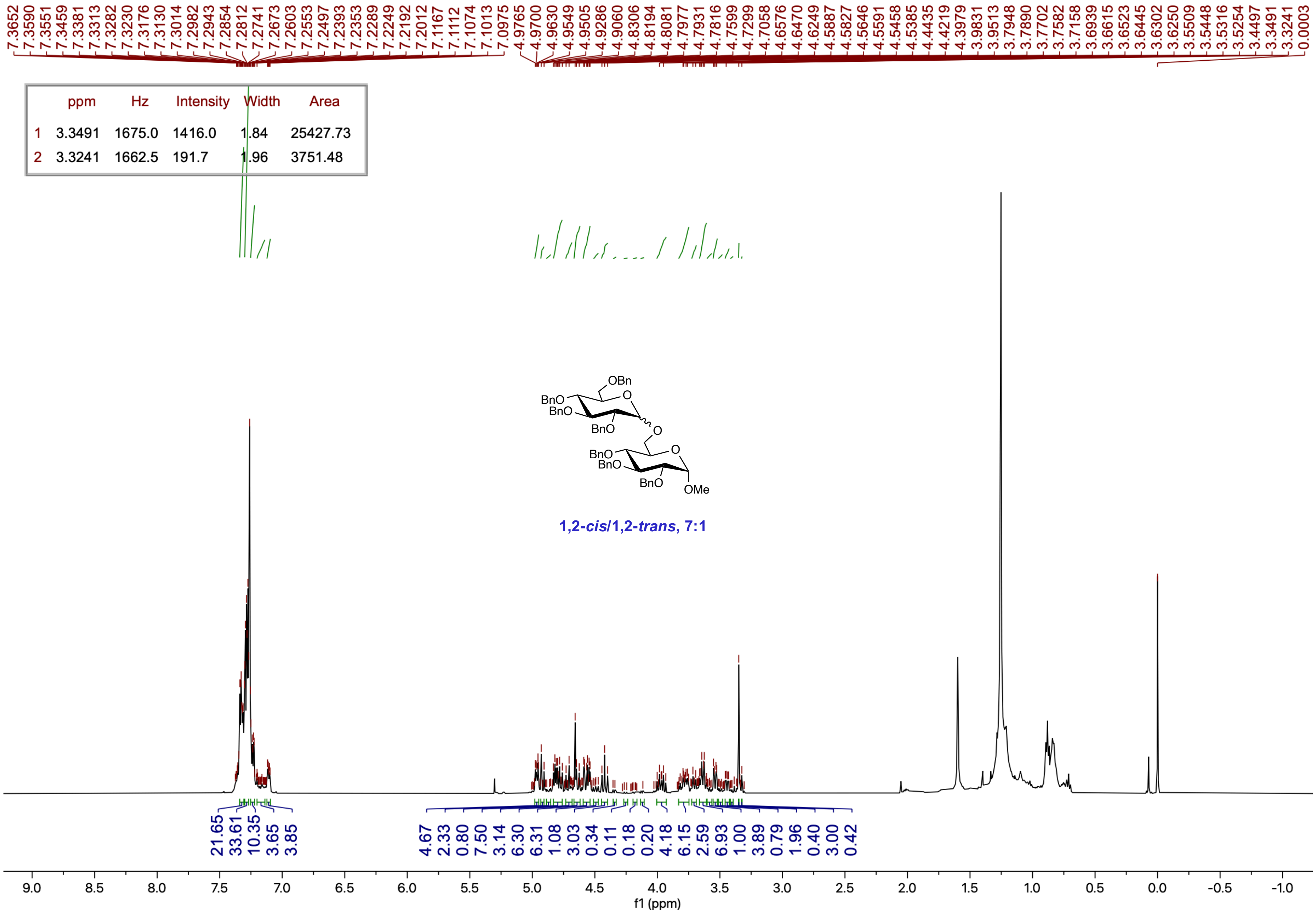

Scheme 2, Entry 16, purified
^{1}H NMR, 400 MHz, $CDCl_3$ with 0.03% TMS

	ppm	Hz	Intensity	Width	Area
1	3.3471	1674.0	3054.5	1.68	50579.29
2	3.3222	1661.5	260.3	1.67	4373.46

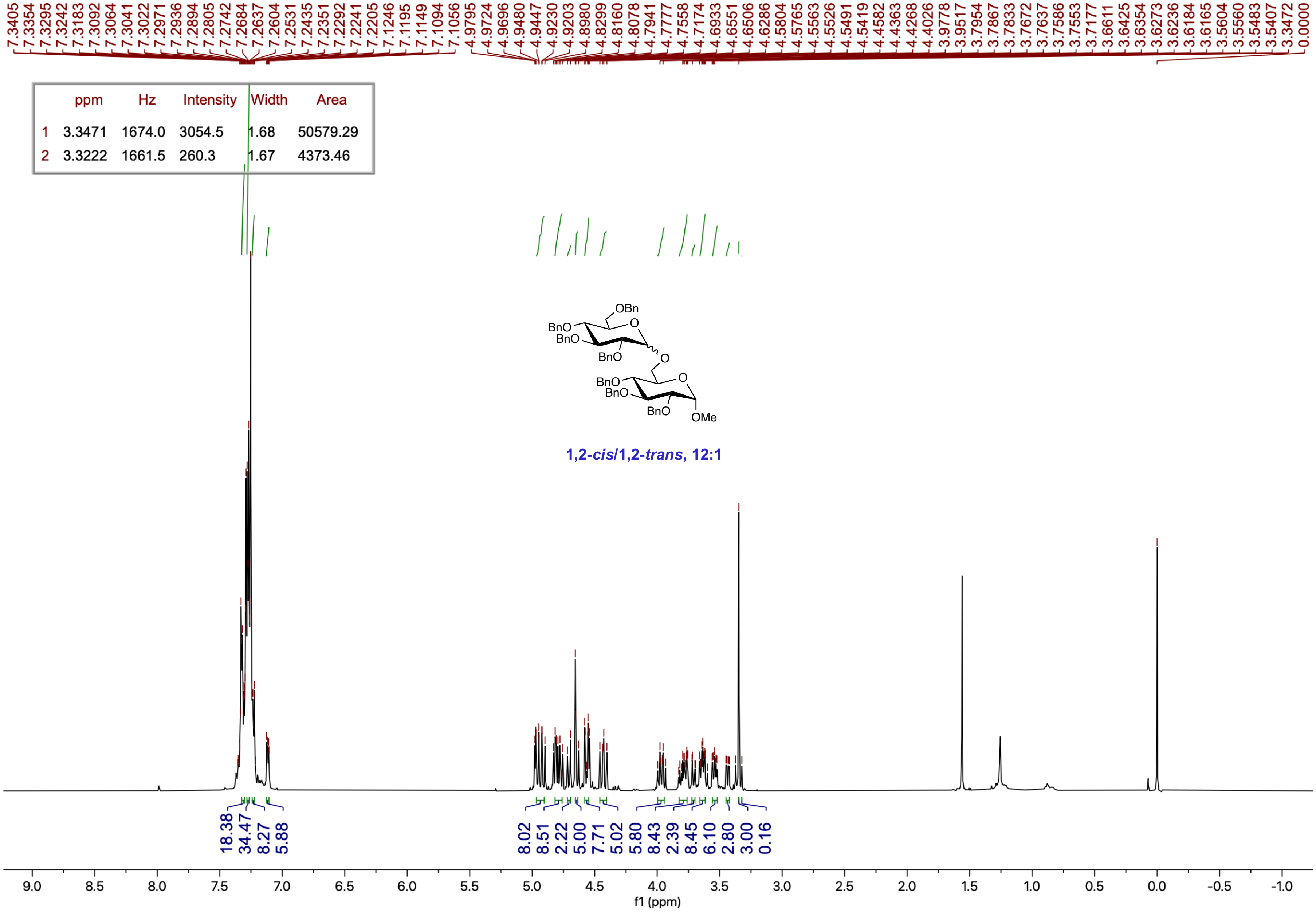

1,2-*cis*/1,2-*trans*, 12:1

Scheme 2, Entry 17, purified
^{1}H NMR, 400 MHz, $CDCl_3$ with 0.03% TMS

	ppm	Hz	Intensity	Width	Area
1	3.3471	1339.3	3296.9	1.53	63241.71
2	3.3220	1329.3	211.2	1.53	4373.04

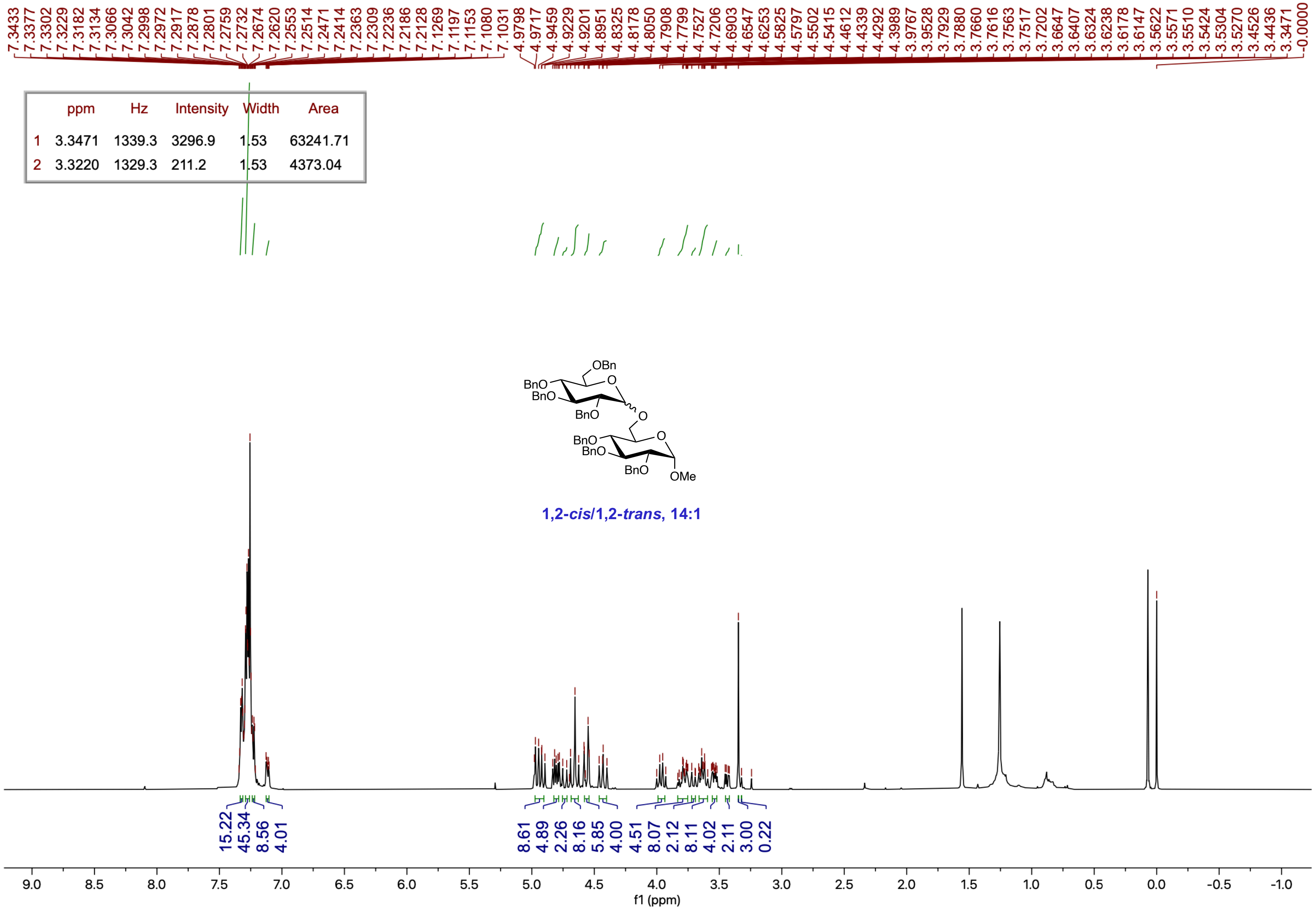

Scheme 2, Entry 18, purified
^{1}H NMR, 400 MHz, $CDCl_3$ with 0.03% TMS

	ppm	Hz	Intensity	Width	Area
1	3.3472	1339.3	3981.4	1.56	76542.49
2	3.3221	1329.3	142.6	1.42	2660.01

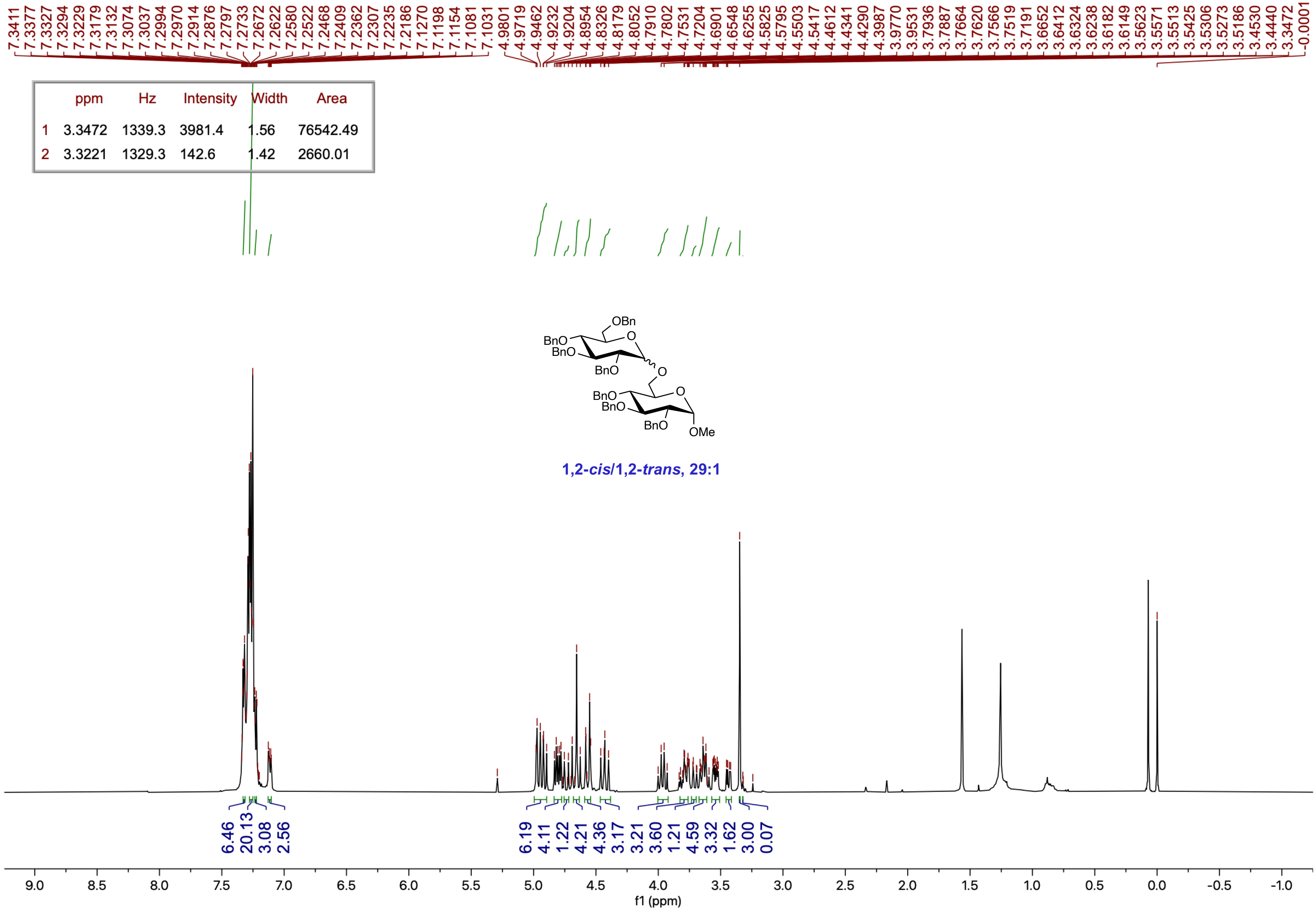

1,2-*cis*/1,2-*trans*, 29:1

Scheme 2, Entry 19, purified

^{1}H NMR, 400 MHz, $CDCl_3$ with 0.03% TMS

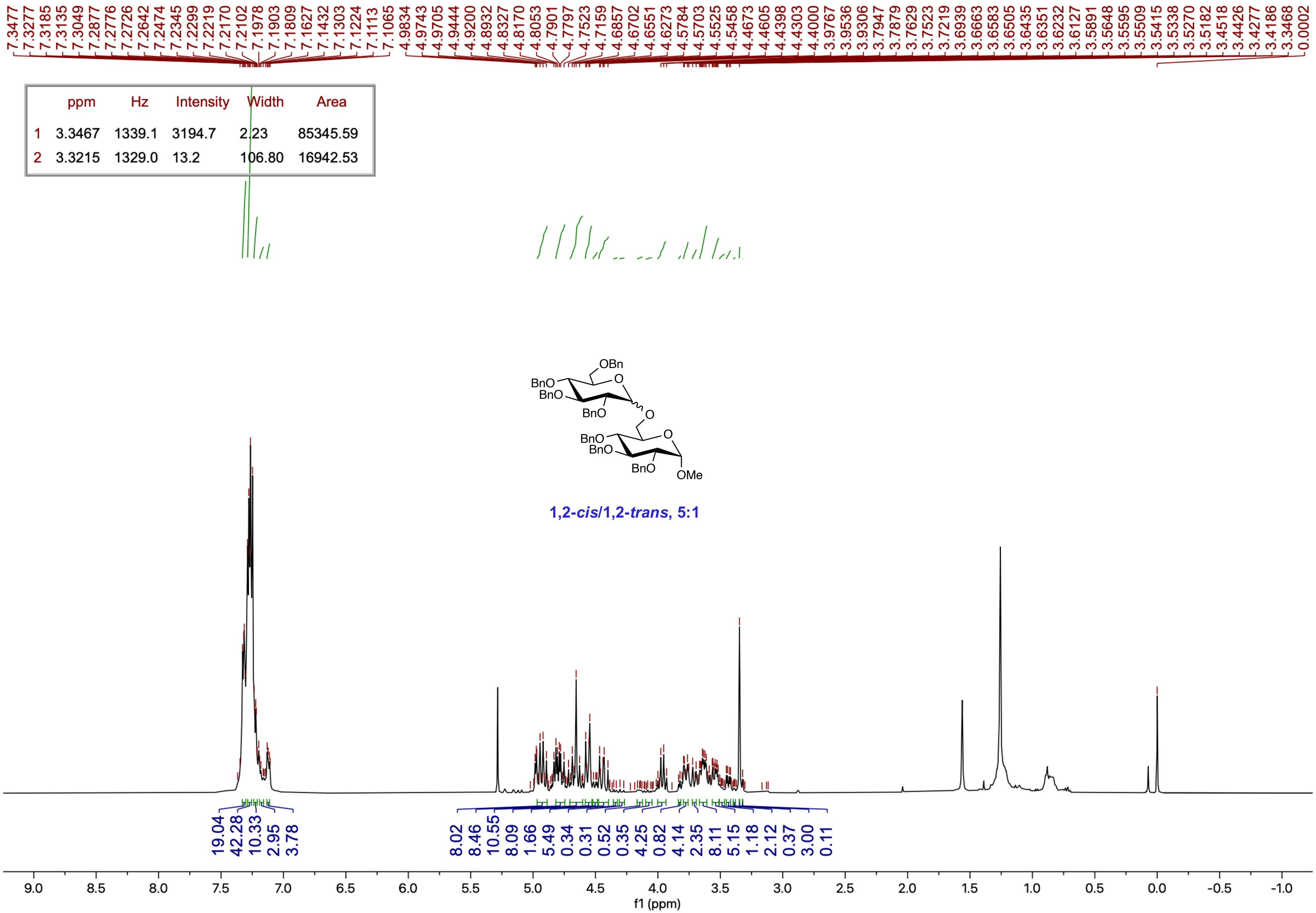

	ppm	Hz	Intensity	Width	Area
1	3.3467	1339.1	3194.7	2.23	85345.59
2	3.3215	1329.0	13.2	106.80	16942.53

1,2-*cis*/1,2-*trans*, 5:1

Scheme 2, Entry 21, purified
^{1}H NMR, 400 MHz, $CDCl_3$ with 0.03% TMS

	ppm	Hz	Intensity	Width	Area
1	3.3472	1674.0	2081.3	3.72	78720.14
2	3.3222	1661.5	360.2	3.85	15409.20

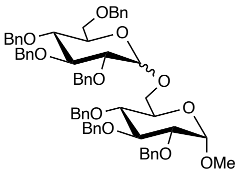

1,2-*cis*/1,2-*trans*, 5:1

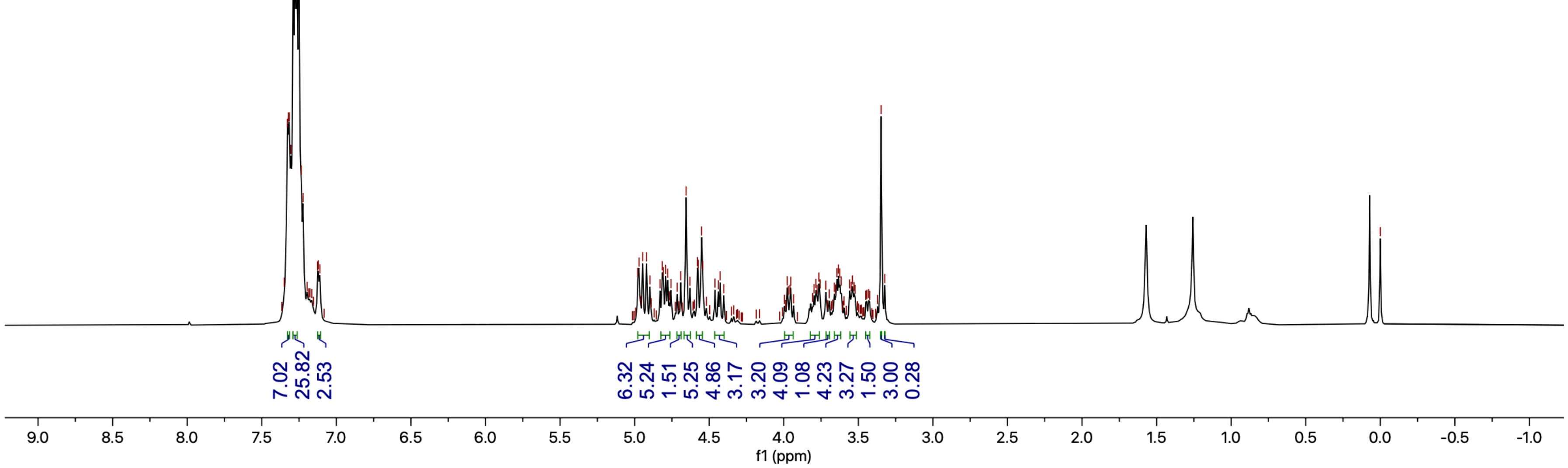

Scheme 2, Entry 22, purified
^1H NMR, 400 MHz, $CDCl_3$ with 0.03% TMS

	ppm	Hz	Intensity	Width	Area
1	3.3474	1339.4	1939.1	4.28	105625.90
2	3.3226	1329.5	221.6	4.73	14055.27

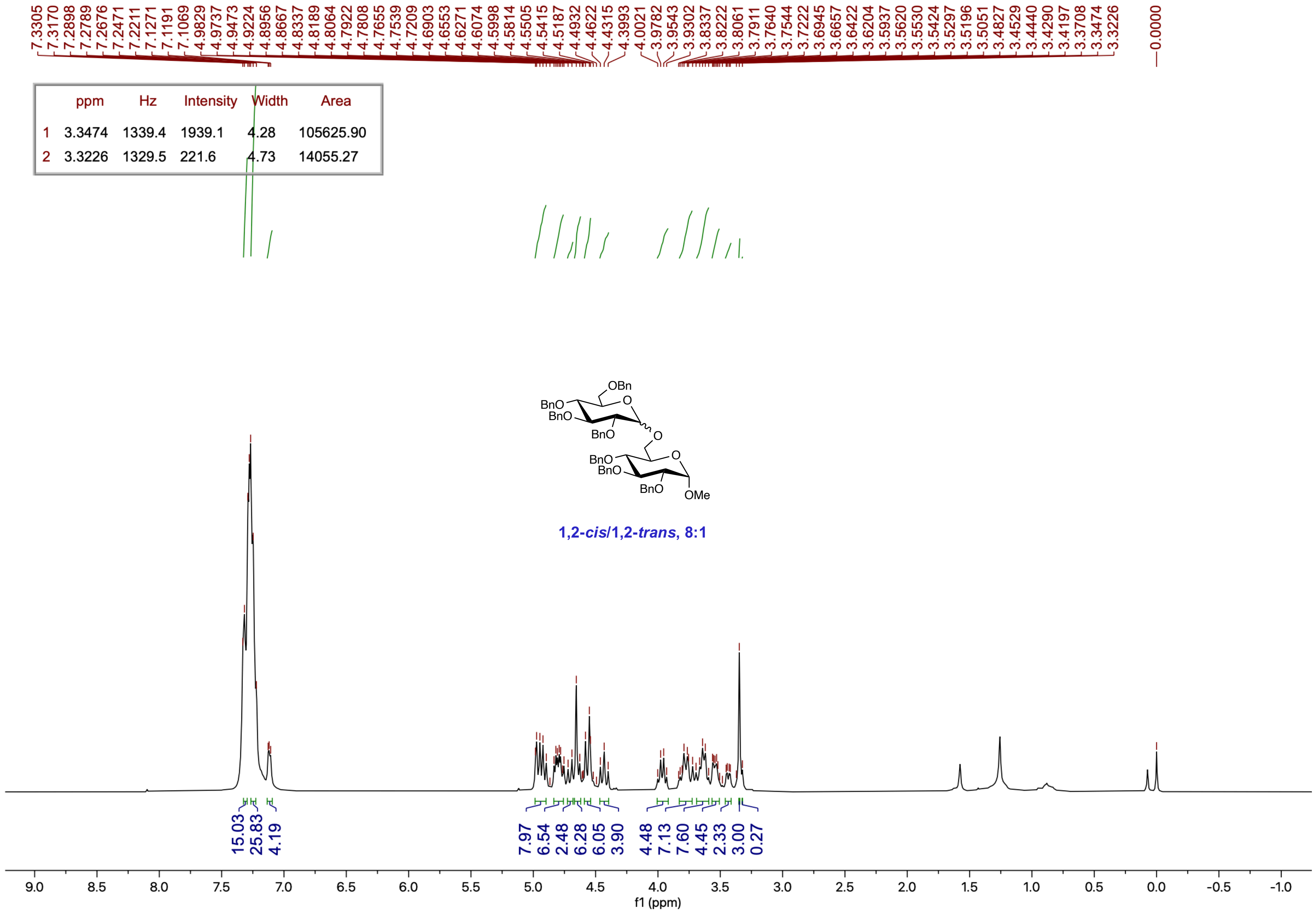

1,2-*cis*/1,2-*trans*, 8:1

Scheme 2, Entry 23, purified
^{1}H NMR, 400 MHz, $CDCl_3$ with 0.03% TMS

	ppm	Hz	Intensity	Width	Area
1	3.3473	1339.4	5732.7	1.33	96285.91
2	3.3224	1329.4	249.3	1.34	4304.76

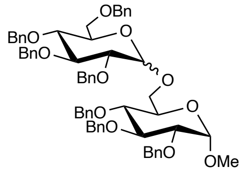

1,2-*cis*/1,2-*trans*, 22:1

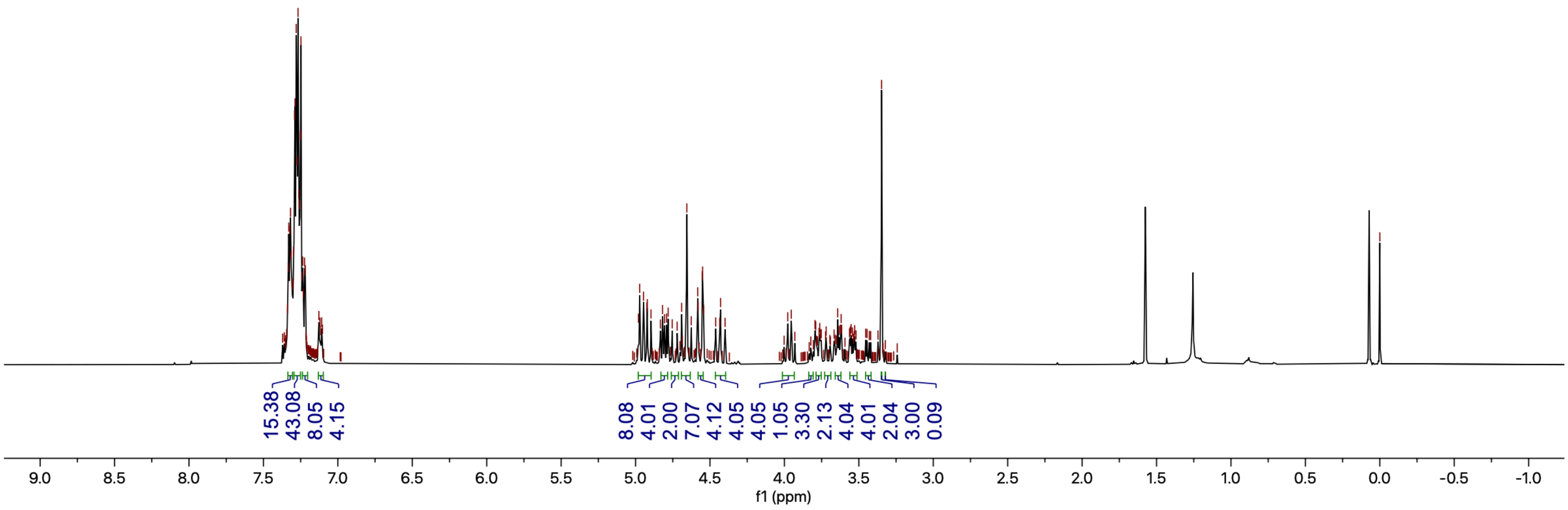

^{1}H NMR Estimation of Anomeric Ratios for the Substrate Scope Study

Scheme 4, Entry 1 (**13**, from **8a**), **purified**
^{1}H NMR, 400 MHz, $CDCl_3$ with 0.03% TMS

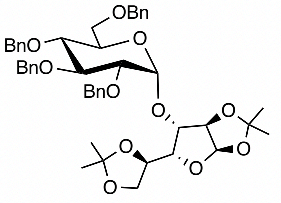

13

1,2-*cis*/1,2-*trans*, >40:1

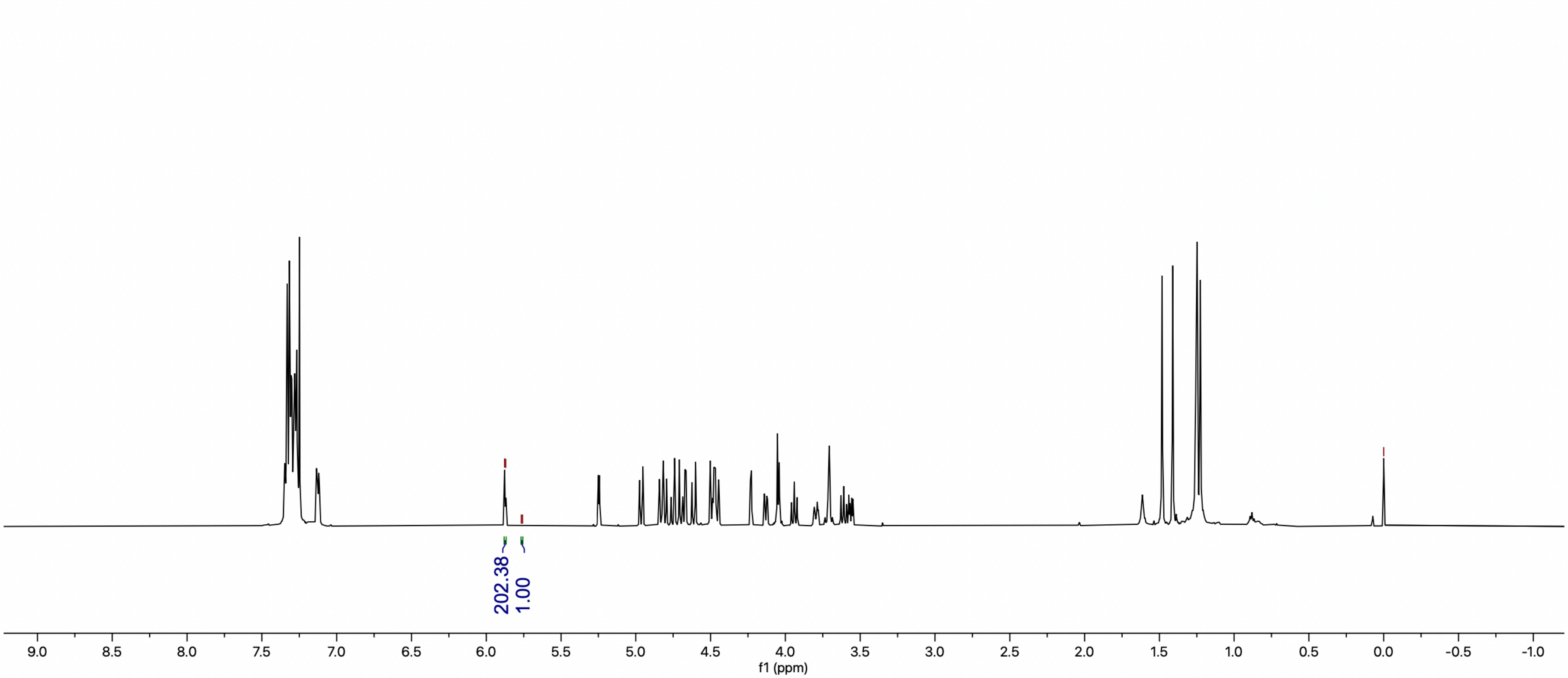

Scheme 4, Entry 1 (**13**, from **8d**), **purified**

^{1}H NMR, 400 MHz, $CDCl_3$ with 0.03% TMS

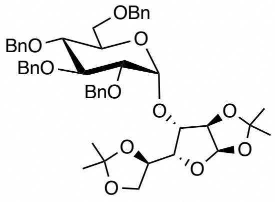

13

1,2-*cis*/1,2-*trans*, >40:1

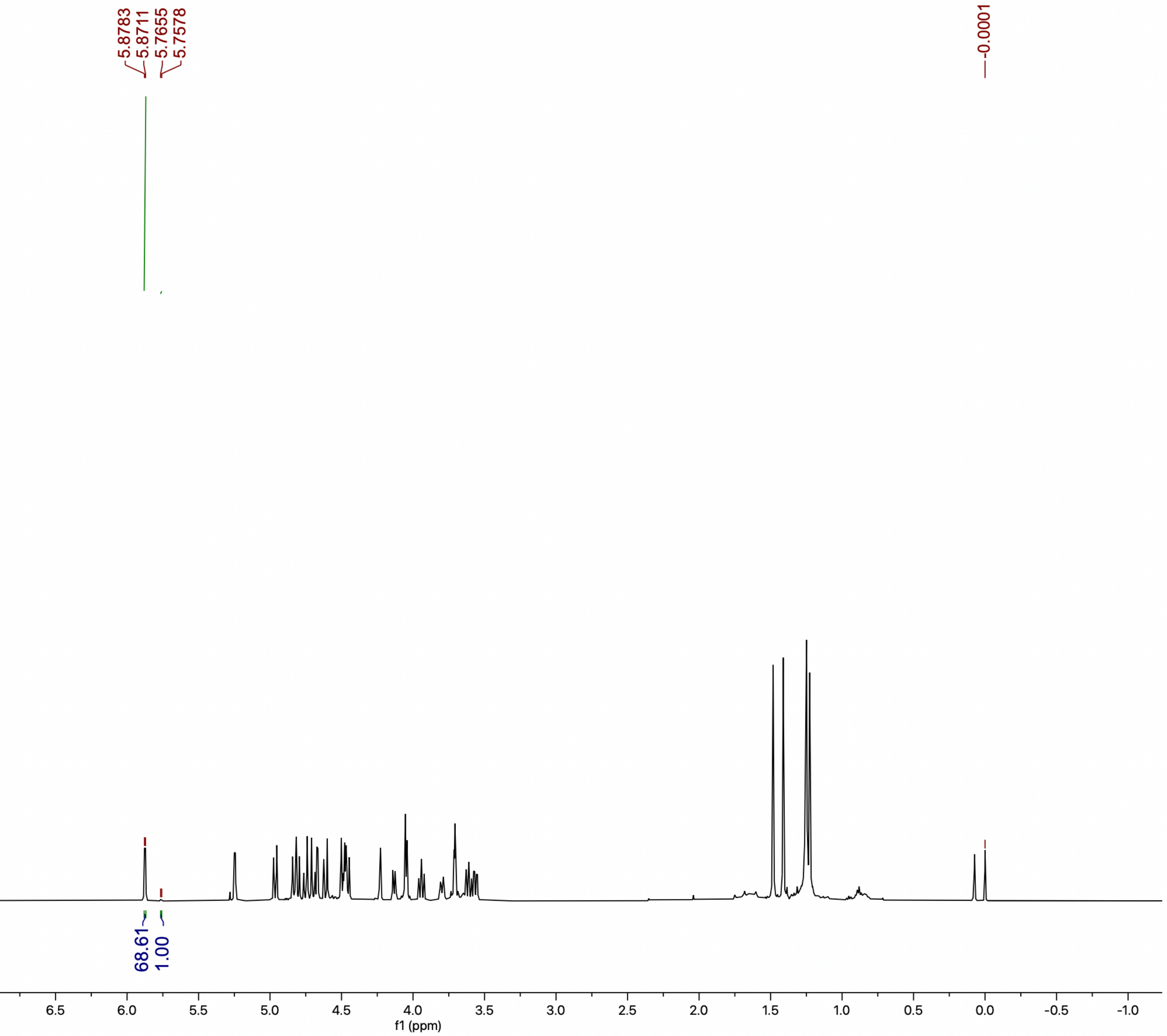

Scheme 4, Entry 2 (**14**, from **8a**), **purified**

^{1}H NMR, 400 MHz, $CDCl_3$ with 0.03% TMS

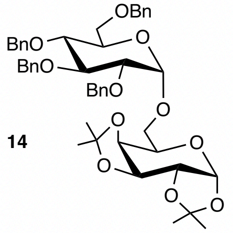

14

1,2-*cis*/1,2-*trans*, 15:1

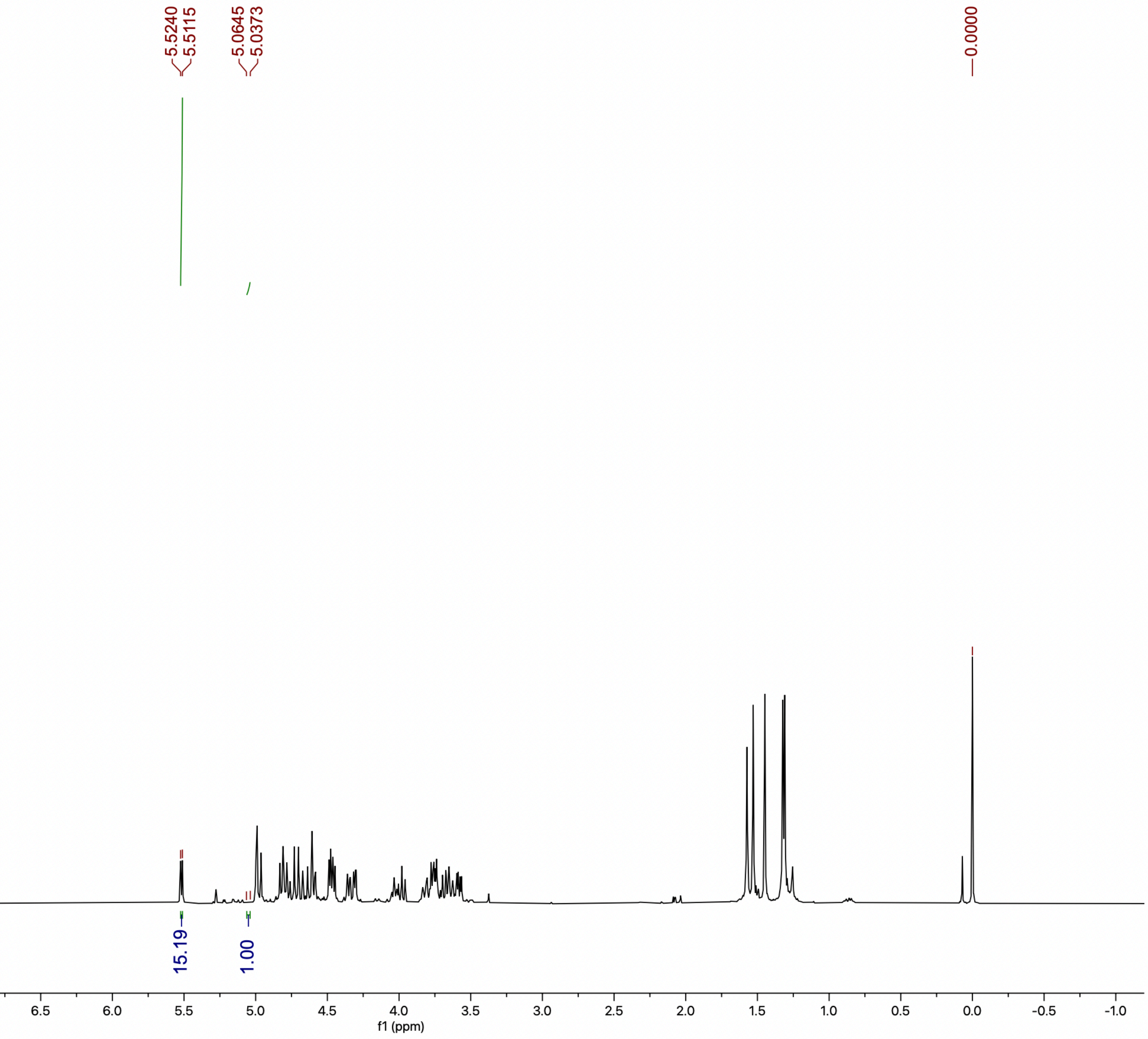

Scheme 4, Entry 2 (**14**, from **8d**), **purified**
^{1}H NMR, 400 MHz, $CDCl_3$ with 0.03% TMS

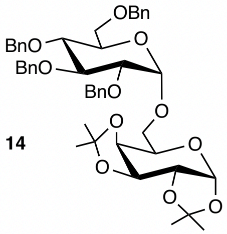

14

1,2-cis/1,2-trans, 24:1

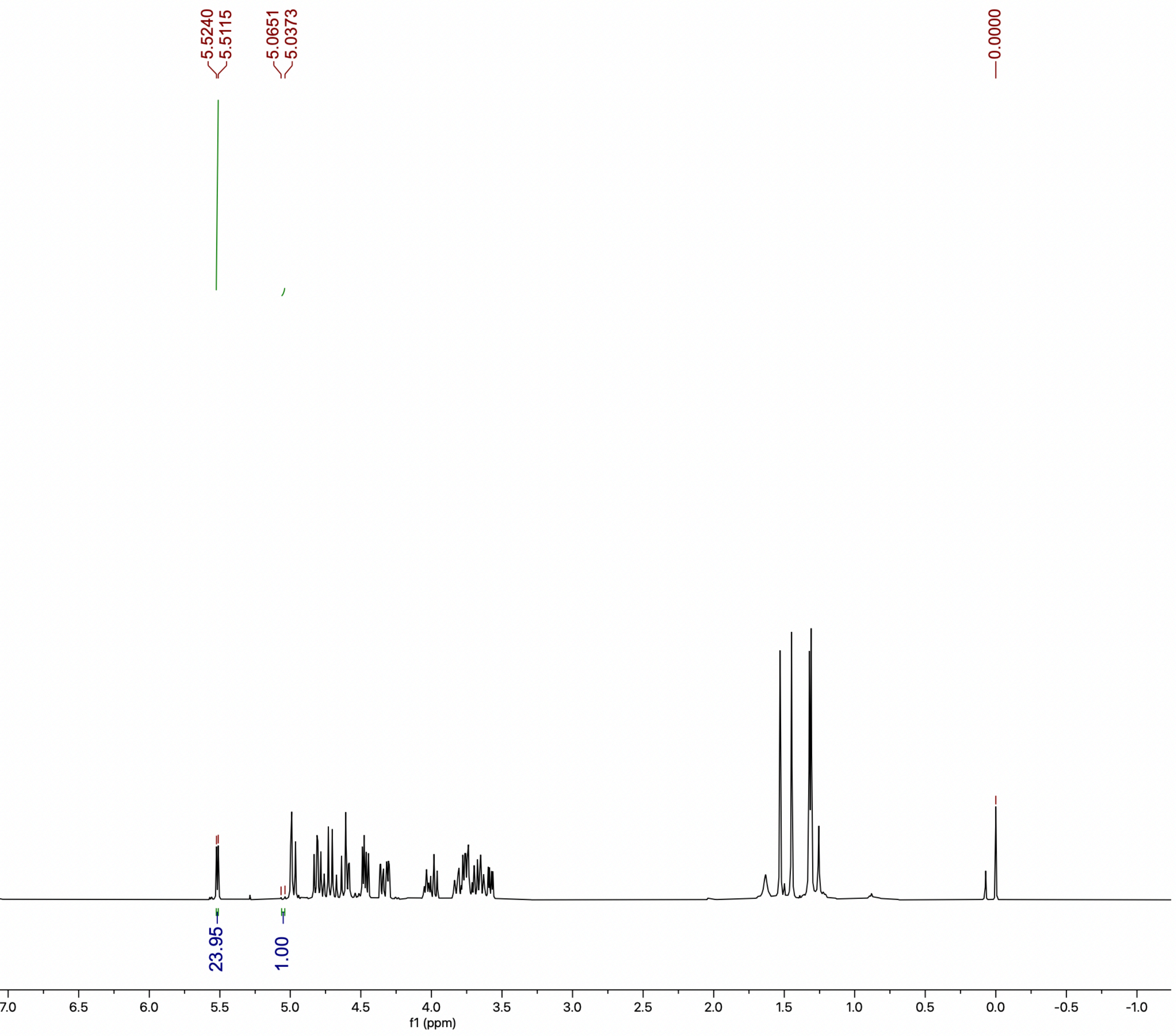

Scheme 4, Entry 3 (**15**, from **8a**), **purified**
^{1}H NMR, 400 MHz, $CDCl_3$ with 0.03% TMS

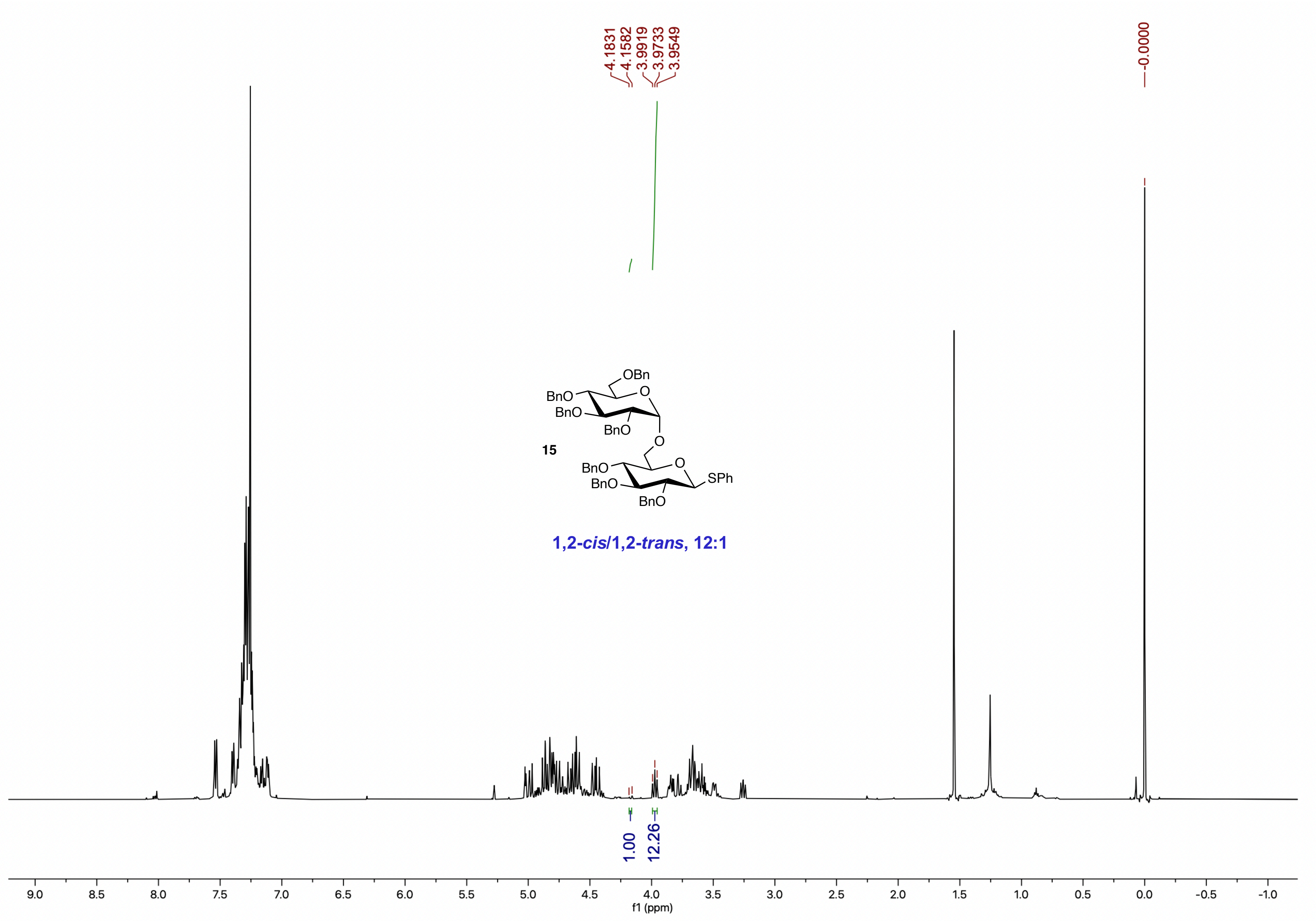

Scheme 4, Entry 3 (**15**, from **8d**), **purified**

^1H NMR, 400 MHz, $CDCl_3$ with 0.03% TMS

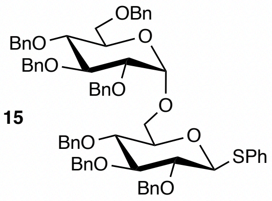

15

1,2-*cis*/1,2-*trans*, 30:1

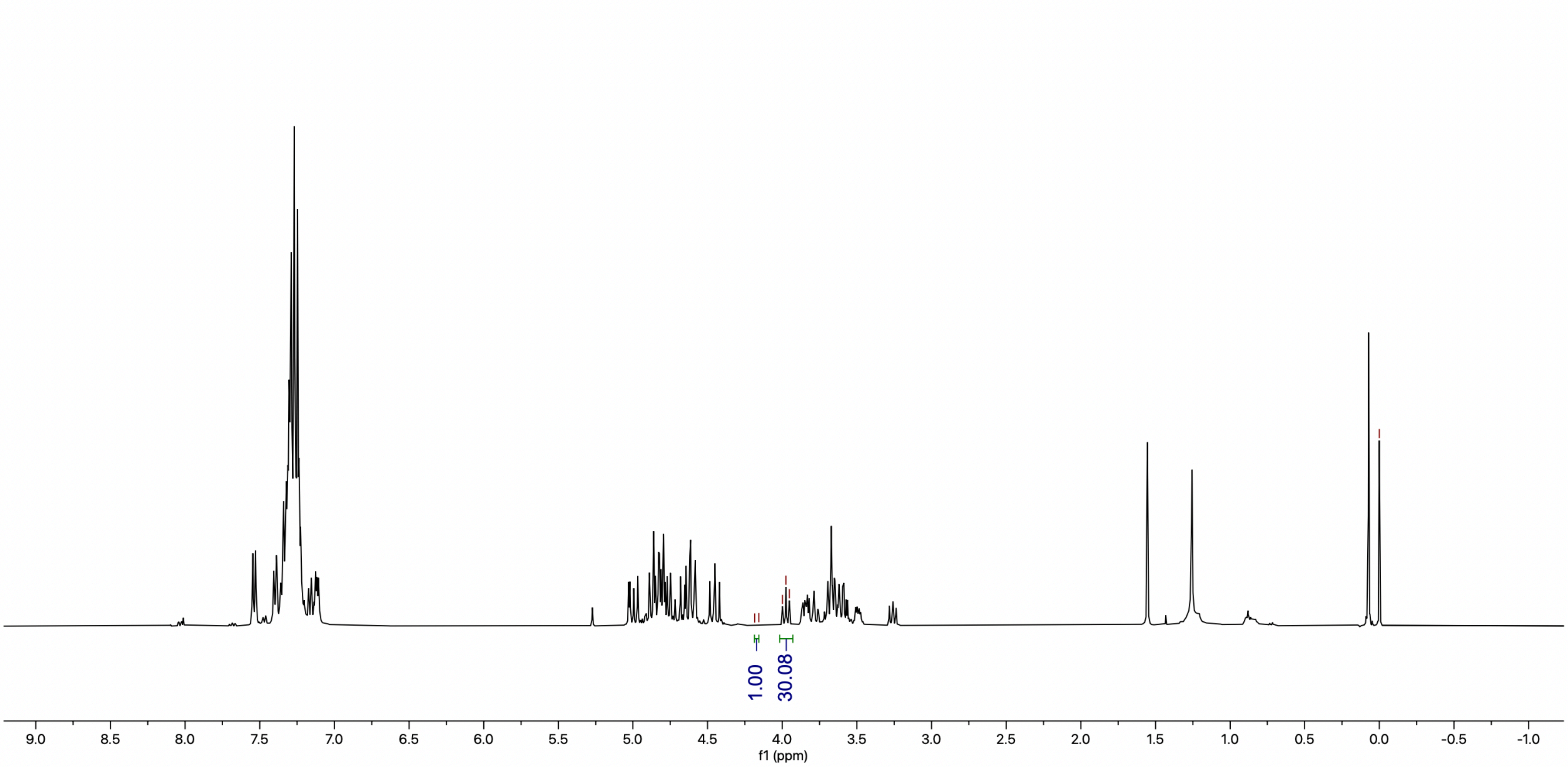

Scheme 4, Entry 4 (**16**, from **8a**), **purified**

^{1}H NMR, 400 MHz, $CDCl_3$ with 0.03% TMS

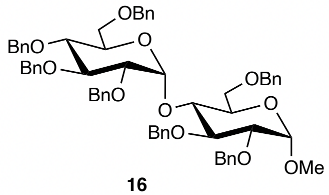

16

1,2-*cis*/1,2-*trans*, 9:1

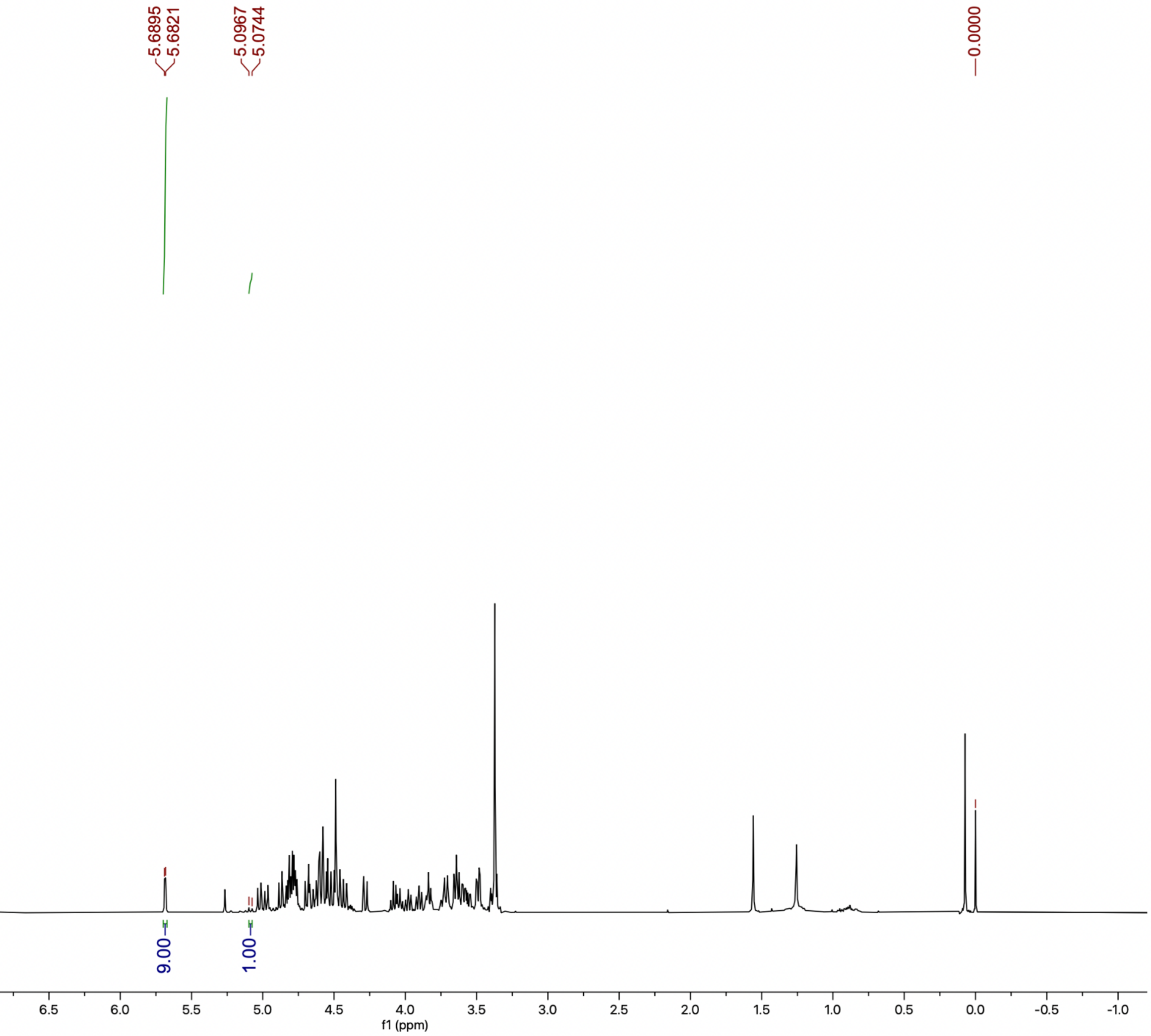

Scheme 4, Entry 4 (**16**, from **8d**), **purified**
^{1}H NMR, 400 MHz, $CDCl_3$ with 0.03% TMS

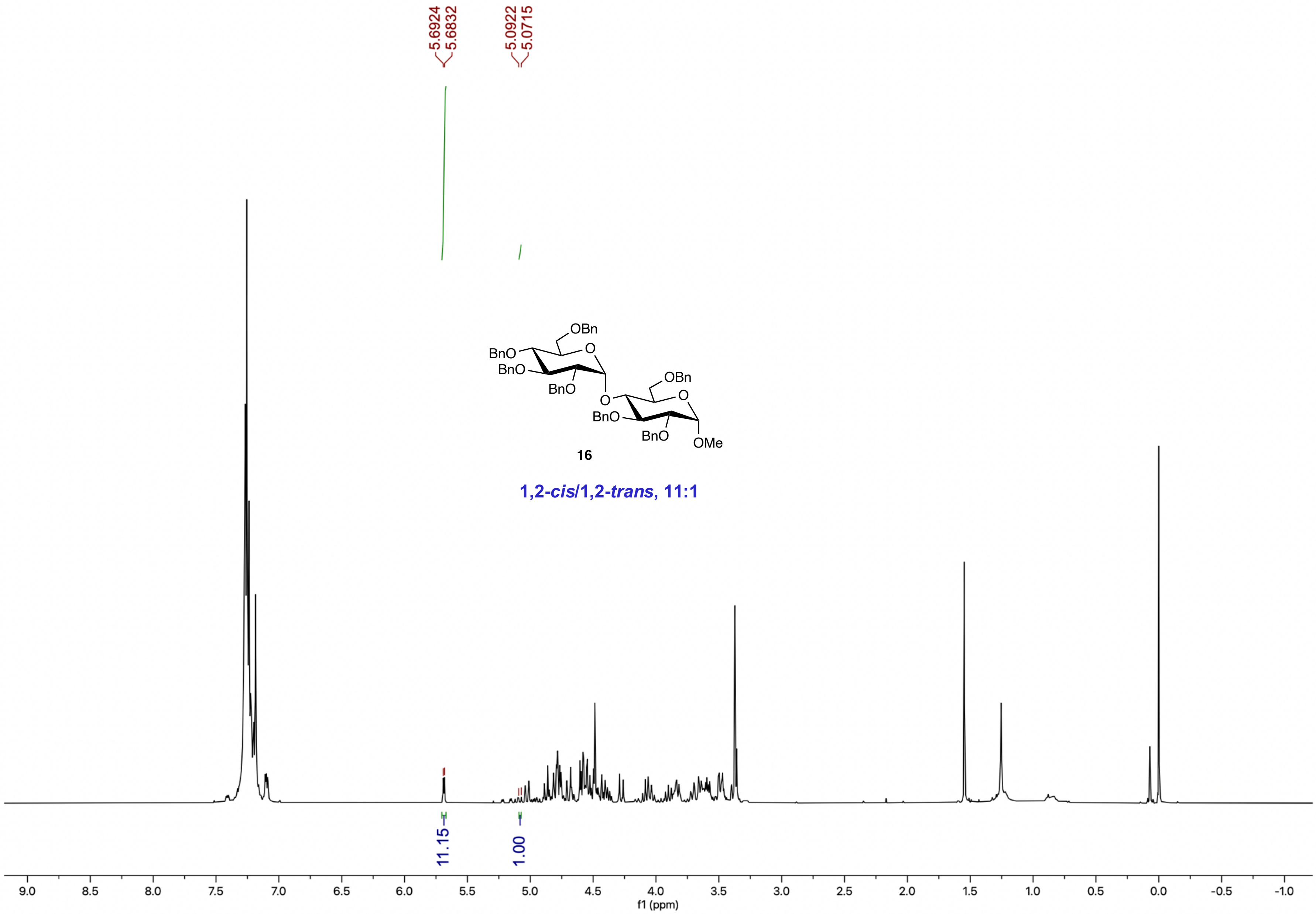

Scheme 4, Entry 5 (**17**, from **8a**), **purified**

^{1}H NMR, 400 MHz, $CDCl_3$ with 0.03% TMS

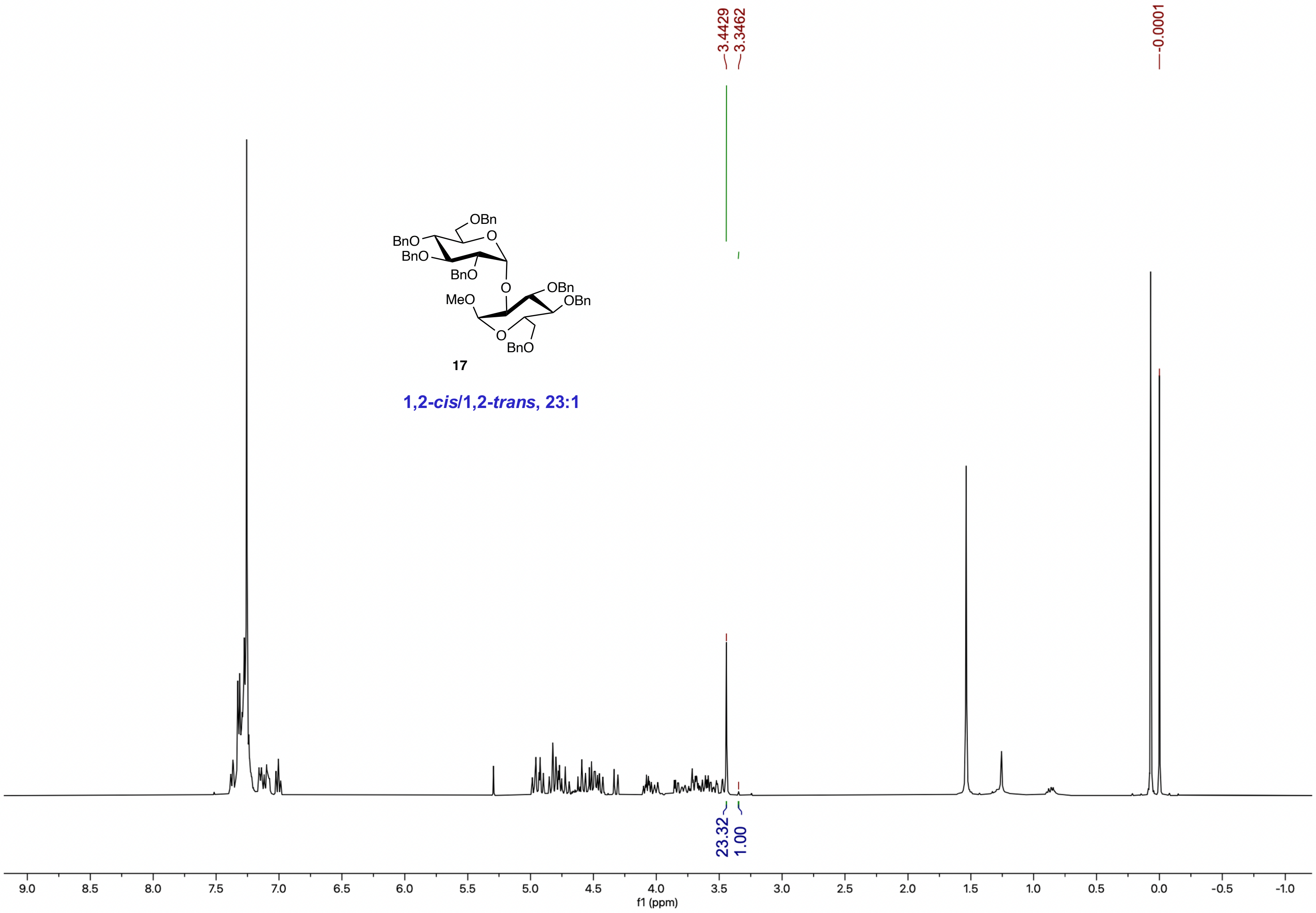

Scheme 4, Entry 5 (17, from 8d), purified
^{1}H NMR, 400 MHz, $CDCl_3$ with 0.03% TMS

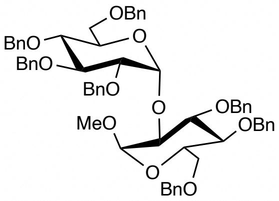

17

1,2-cis/1,2-trans, 37:1

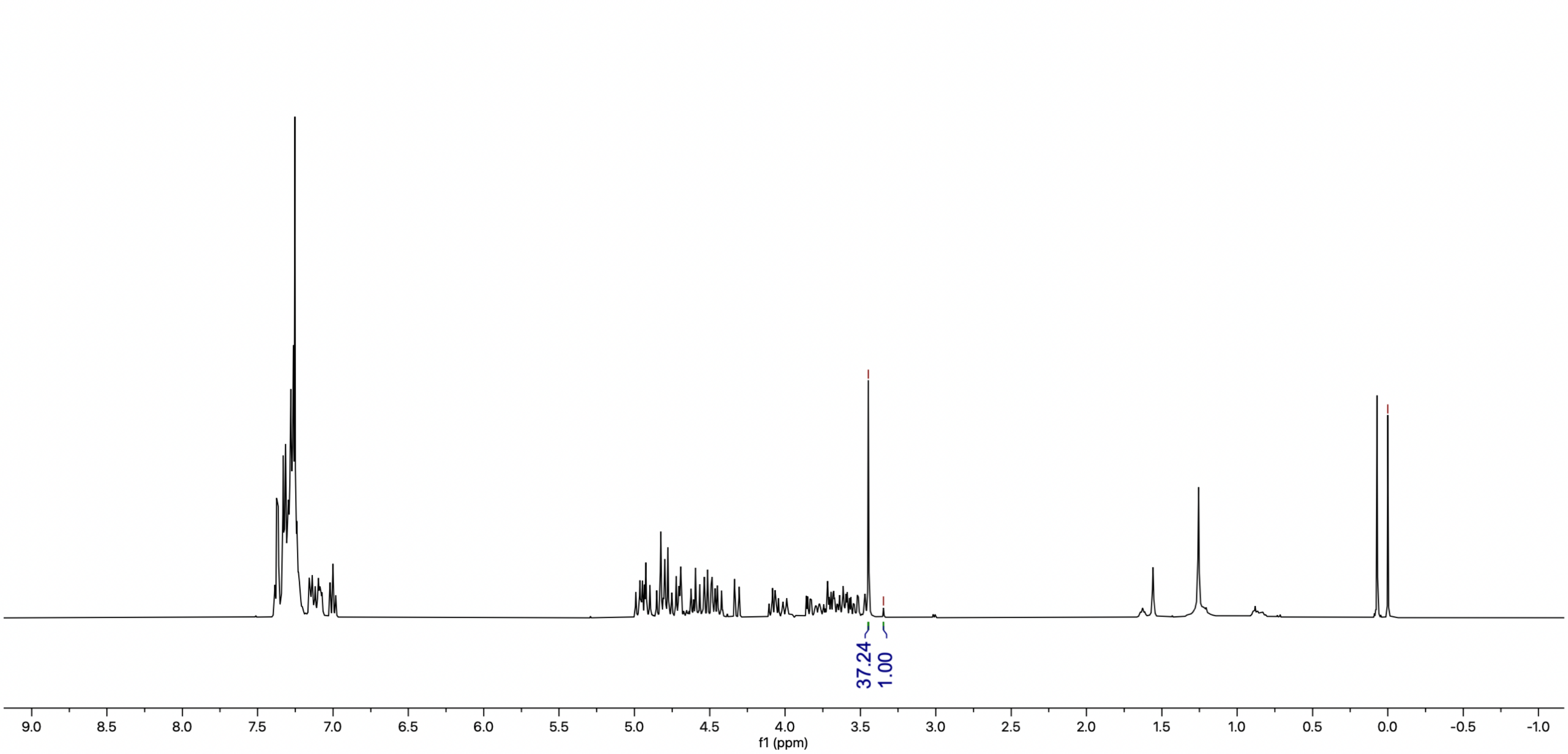

Scheme 4, Entry 6 (**18**, from **8a**), **purified**
^{1}H NMR, 400 MHz, $CDCl_3$ with 0.03% TMS

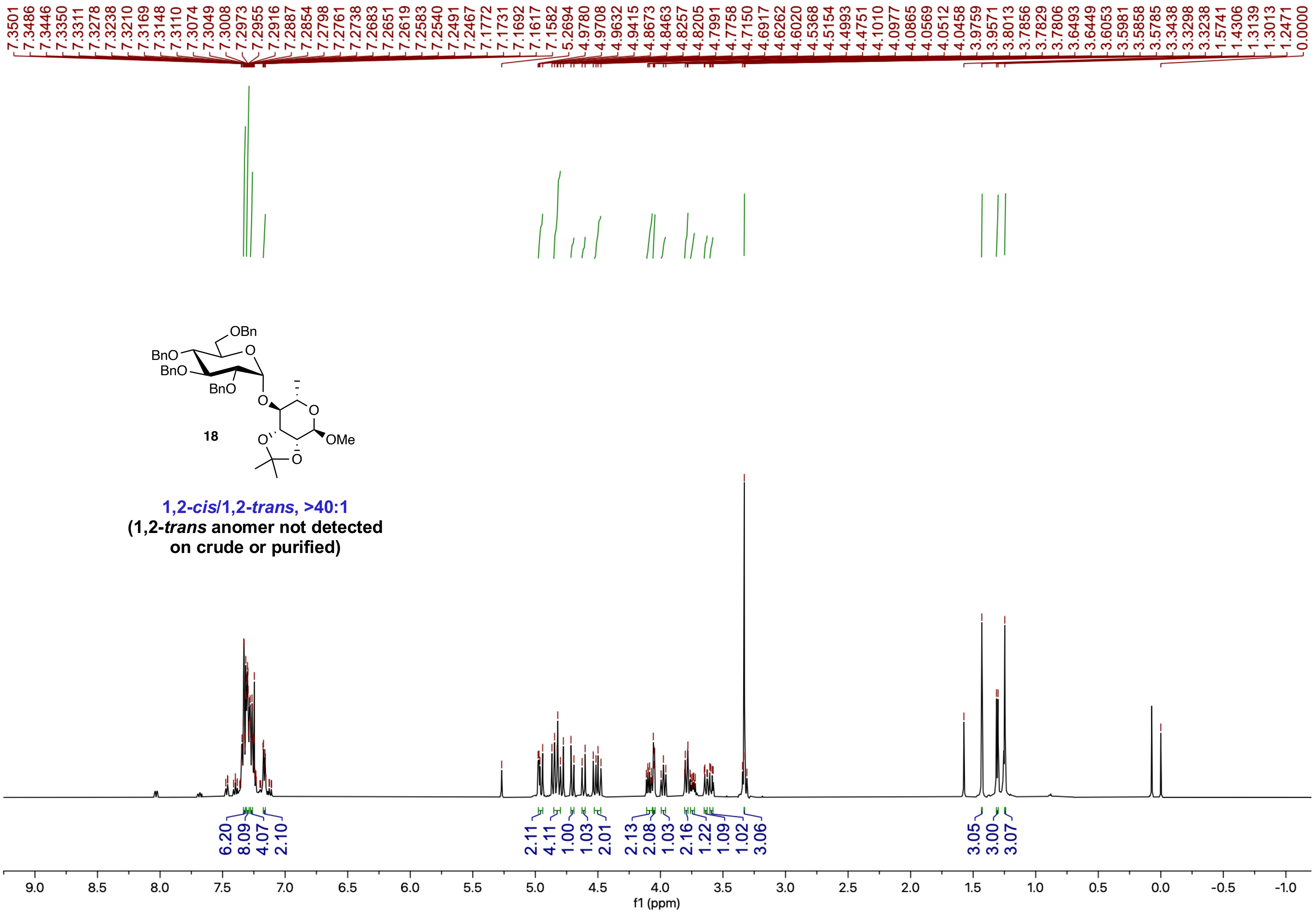

Scheme 4, Entry 6 (**18**, from **8d**), **purified**

^{1}H NMR, 400 MHz, $CDCl_3$ with 0.03% TMS

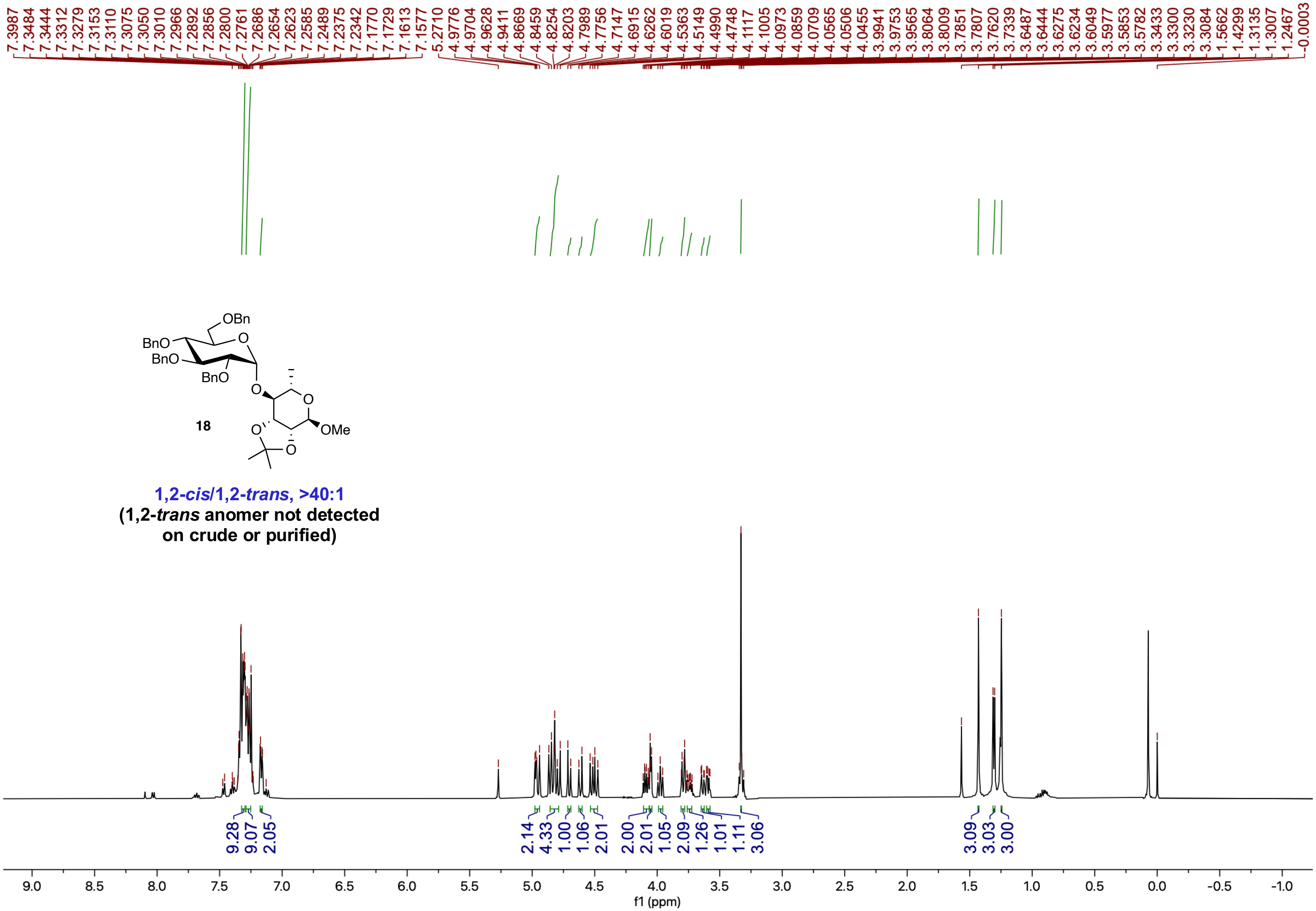

Scheme 4, Entry 7 (**19**, from **8a**), **purified**
^1H NMR, 400 MHz, $CDCl_3$ with 0.03% TMS

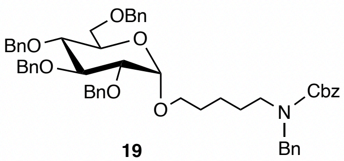

19

1,2-*cis*/1,2-*trans*, 14:1

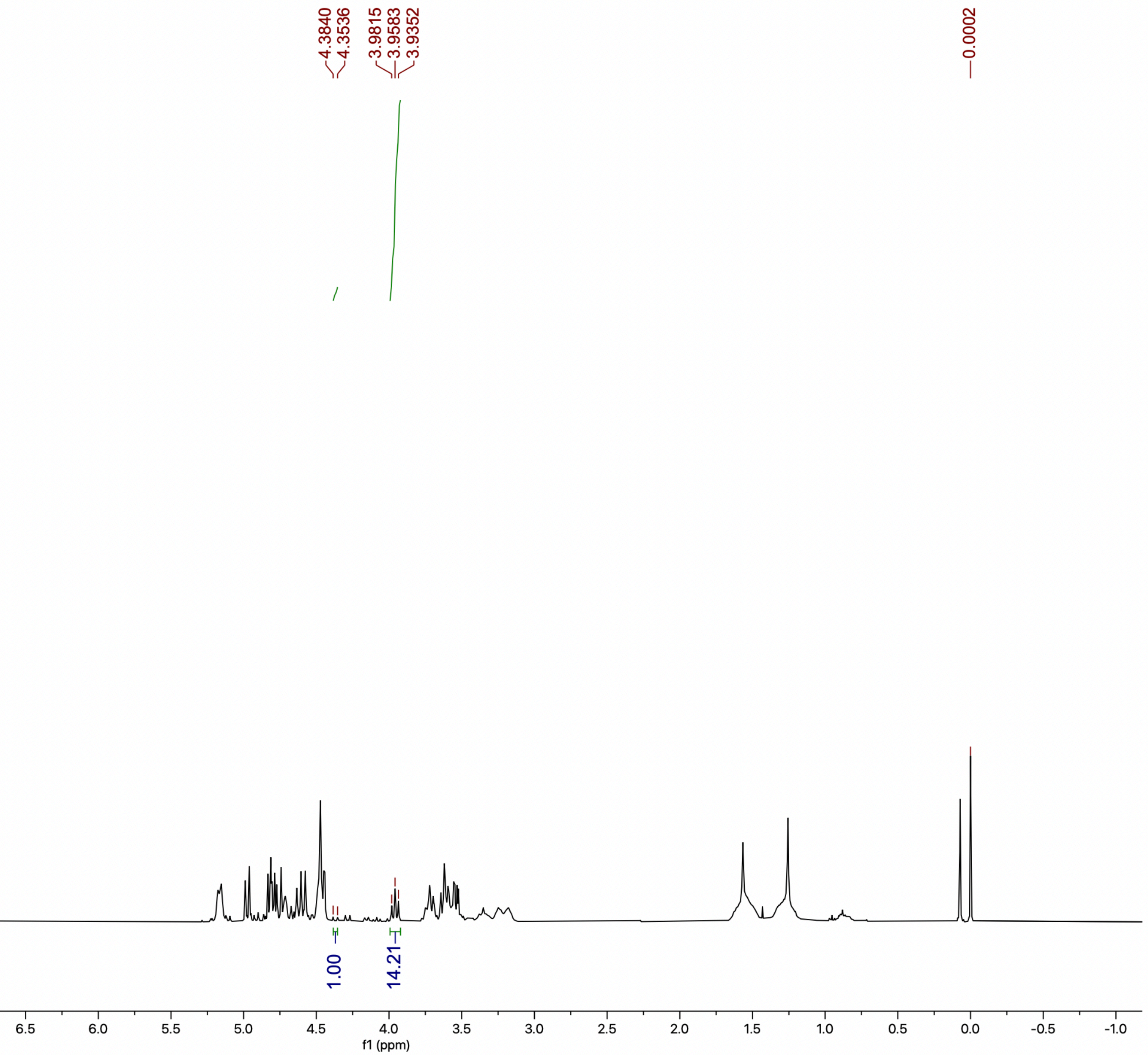

Scheme 4, Entry 7 (**19**, from **8d**), **purified**
^{1}H NMR, 400 MHz, $CDCl_3$ with 0.03% TMS

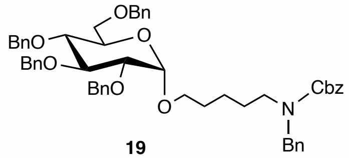

19

1,2-cis/1,2-trans, 24:1

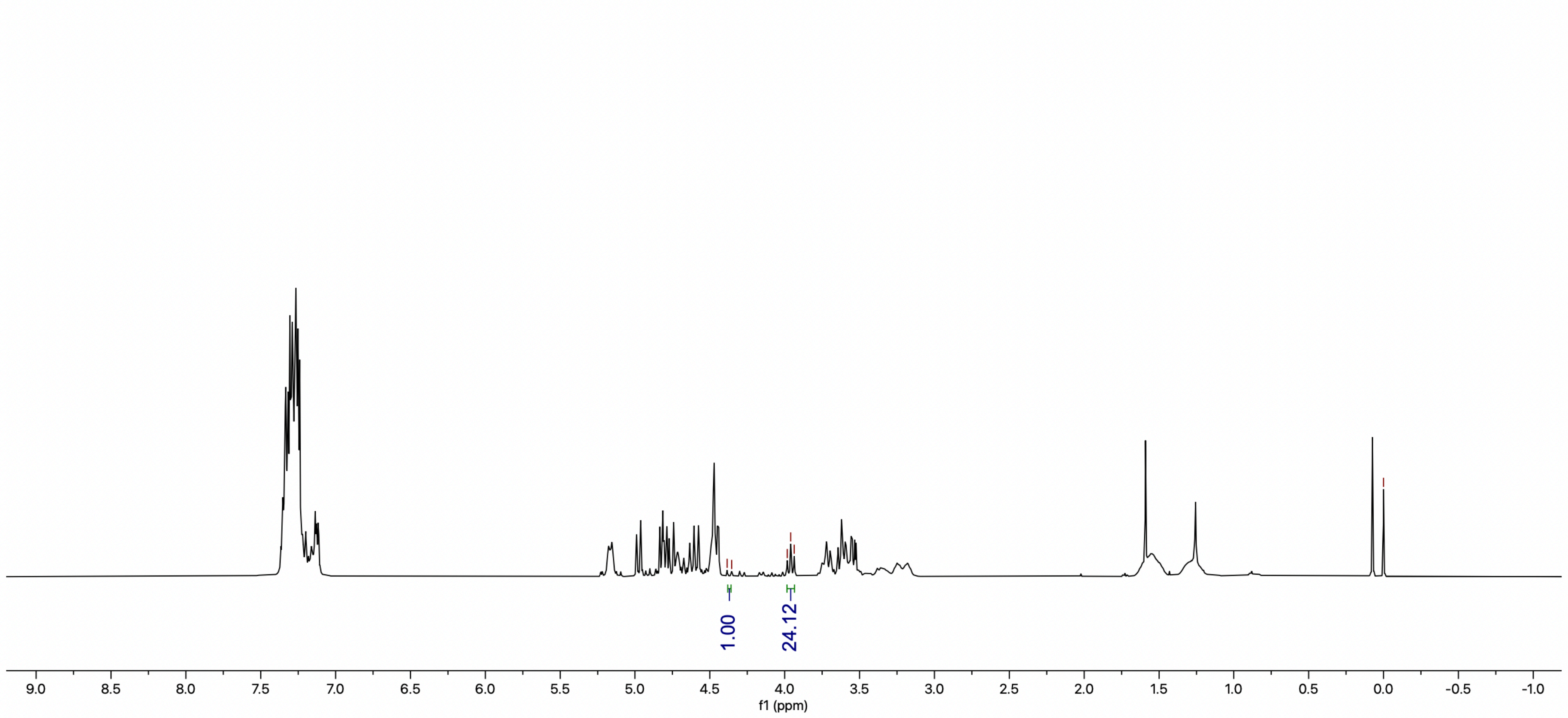

Scheme 4, Entry 8 (**20**, from **8e**), **purified**
^{1}H NMR, 400 MHz, $CDCl_3$ with 0.03% TMS

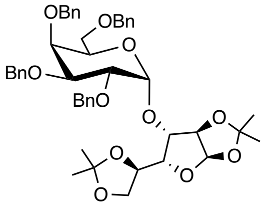

20

1,2-cis/1,2-trans, >40:1

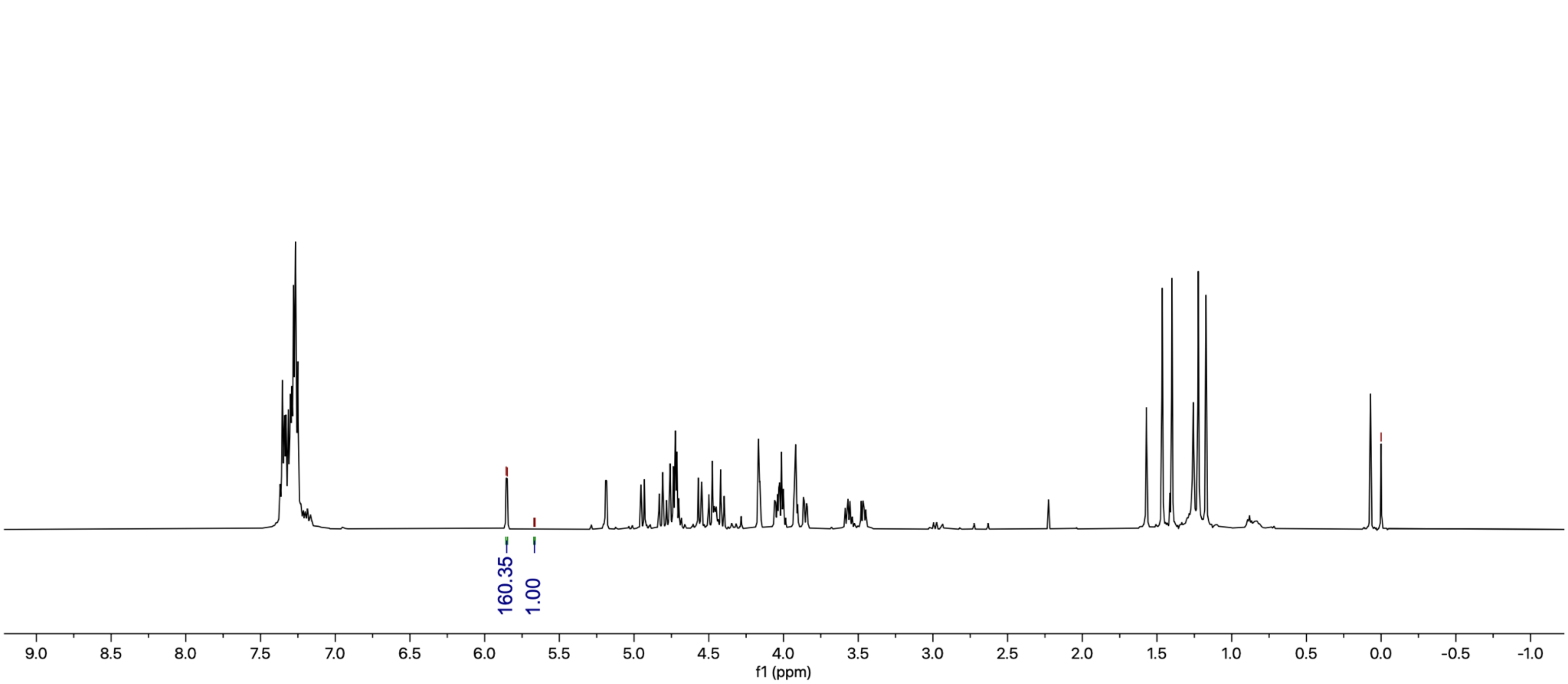

Scheme 4, Entry 9 (**21**, from **8e**), **purified**
^{1}H NMR, 400 MHz, $CDCl_3$ with 0.03% TMS

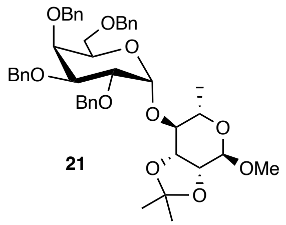

21

1,2-*cis*/1,2-*trans*, >40:1

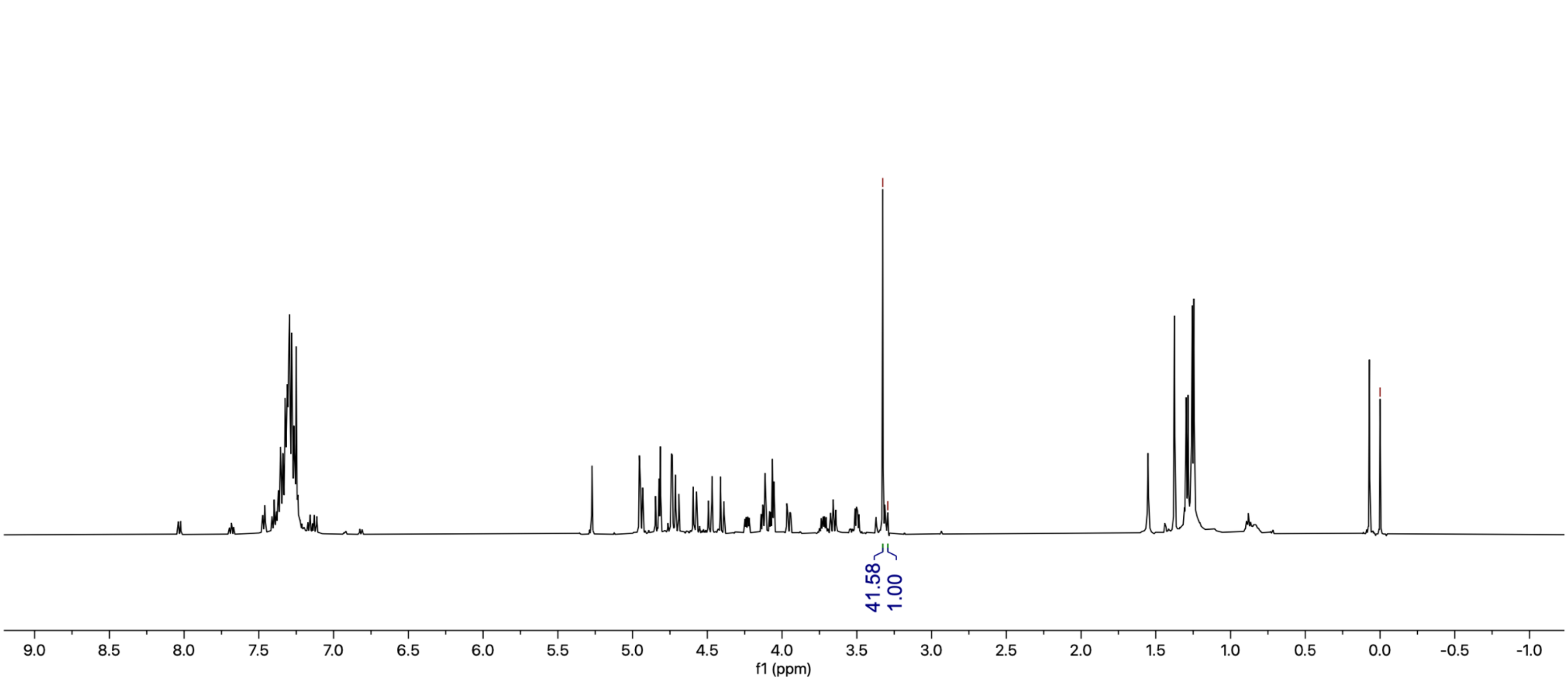

Scheme 4, Entry 10 (**22**, from **8f**), **purified**
^{1}H NMR, 400 MHz, $CDCl_3$ with 0.03% TMS

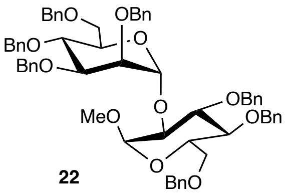

22

1,2-*cis*/1,2-*trans*, 1:>40

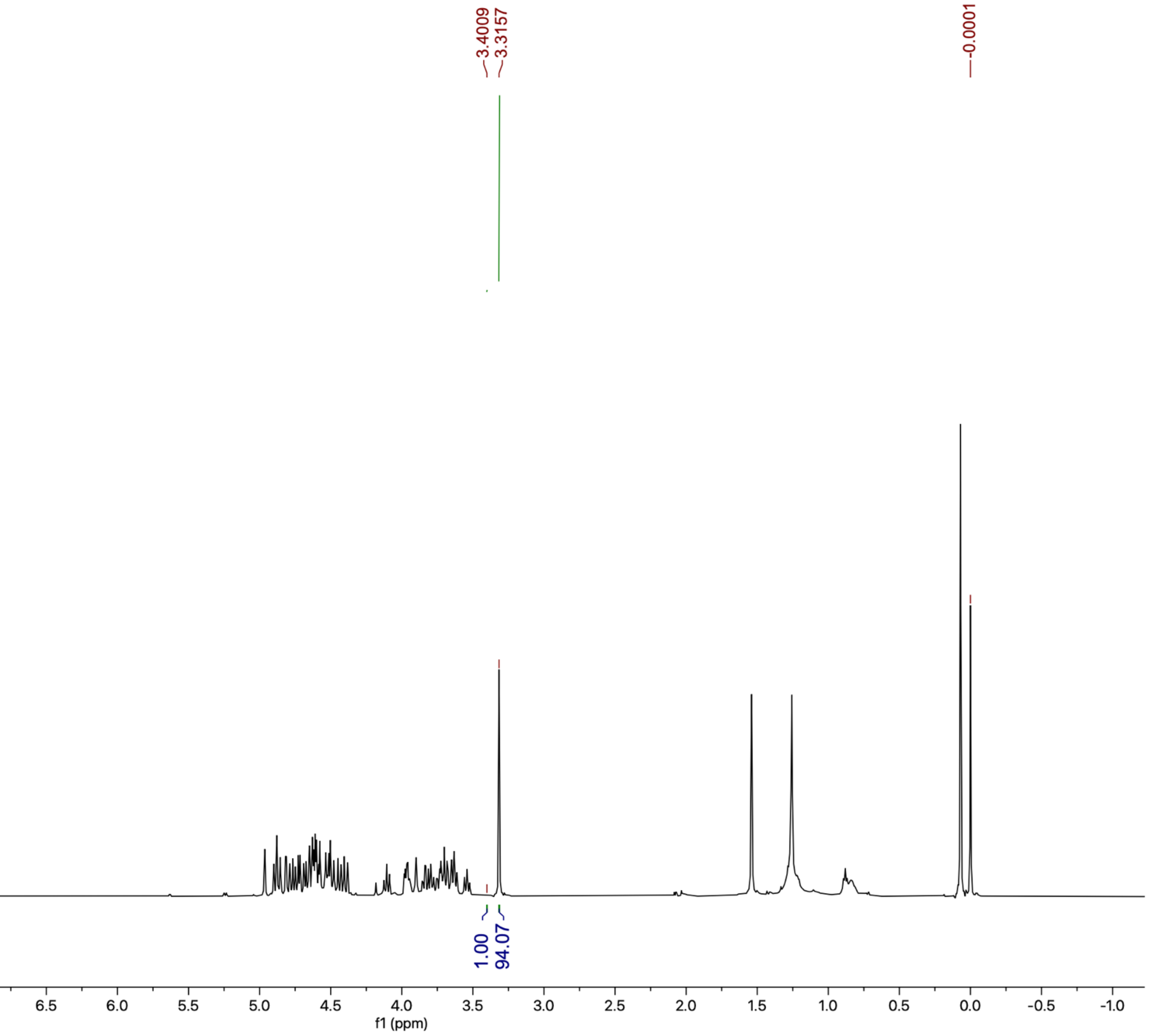

Scheme 4, Entry 11 (**23**, from **8f**), **purified**
^{1}H NMR, 400 MHz, $CDCl_3$ with 0.03% TMS

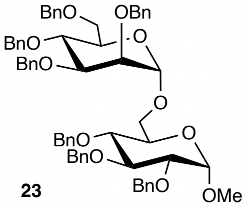

23

1,2-*cis*/1,2-*trans*, 1:>40

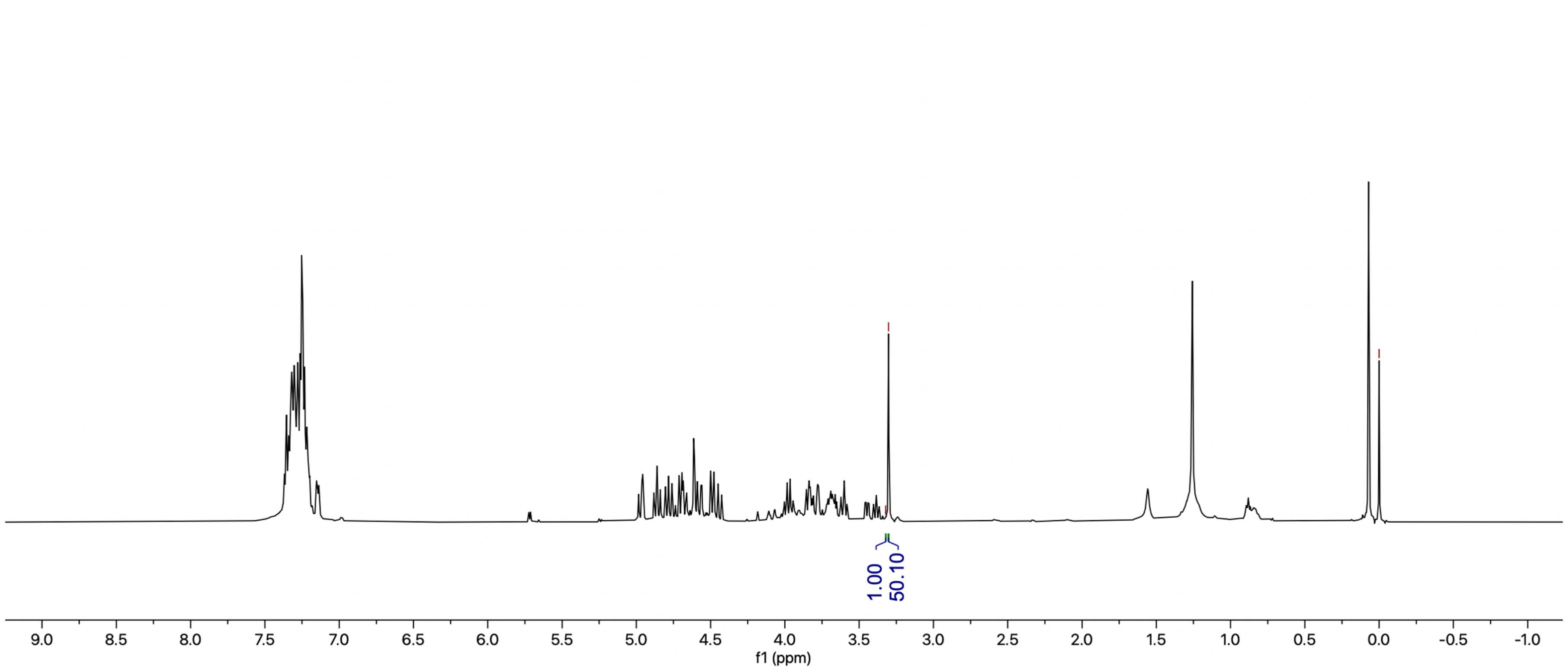

Scheme 4, Entry 12 (**24**, from **8f**), **purified**
^{1}H NMR, 400 MHz, $CDCl_3$ with 0.03% TMS

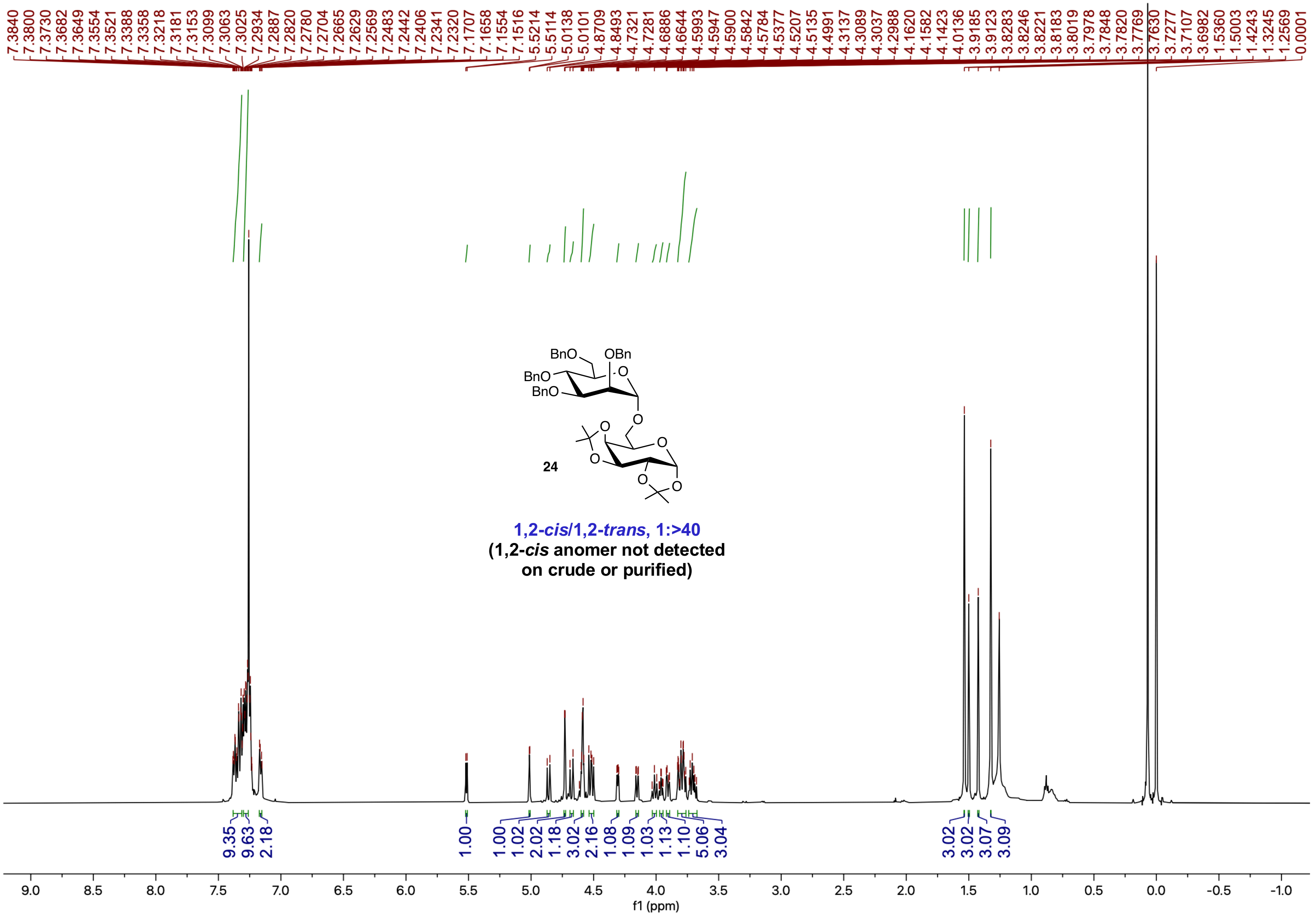

Scheme 4, Entry 13 (α**-25**, from β**-8g**), **purified**
^{1}H NMR, 400 MHz, $CDCl_3$ with 0.03% TMS

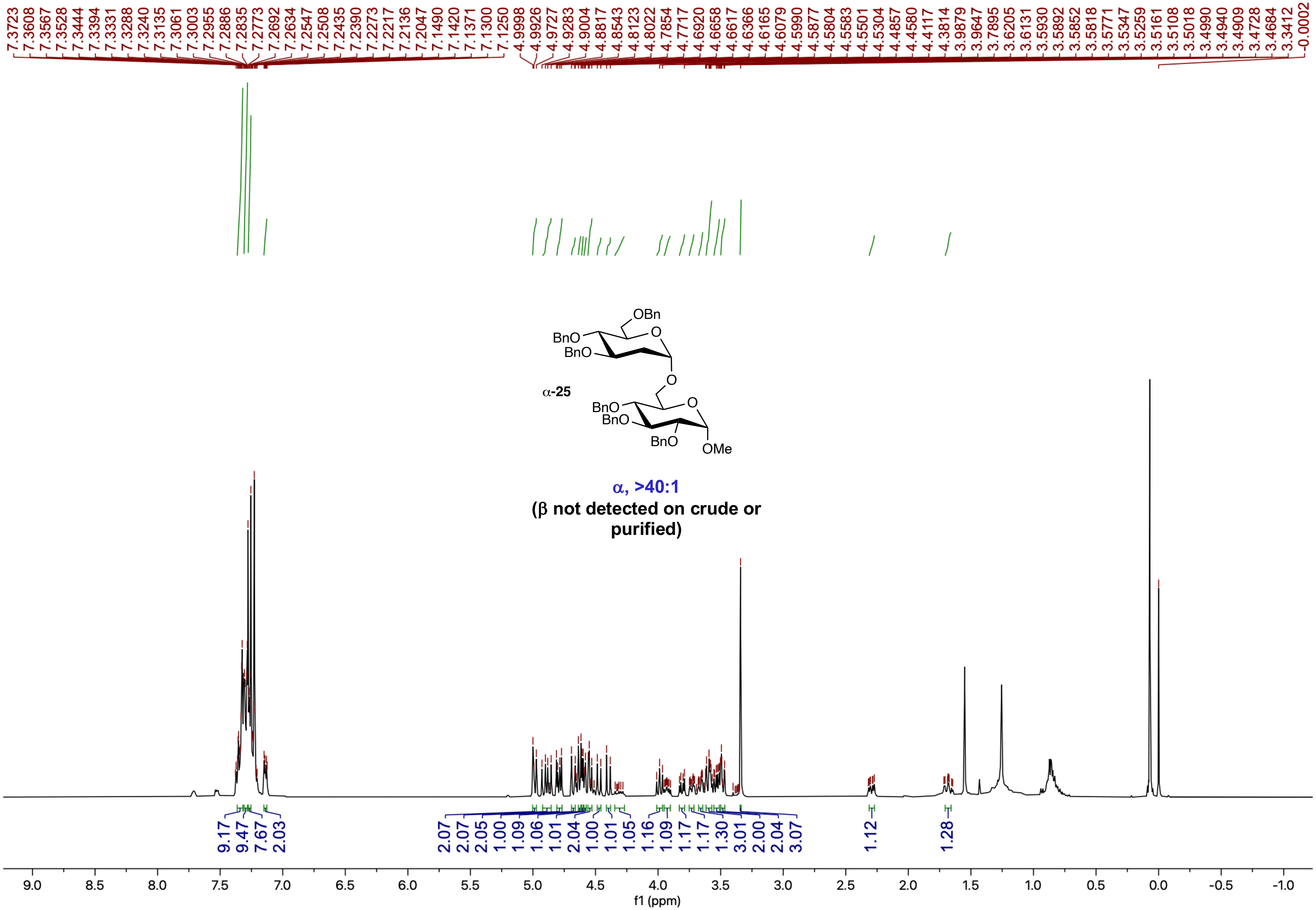

Scheme 4, Entry 14, (**β-25**, from **α-8g**), **purified**
^{1}H NMR, 400 MHz, $CDCl_3$ with 0.03% TMS

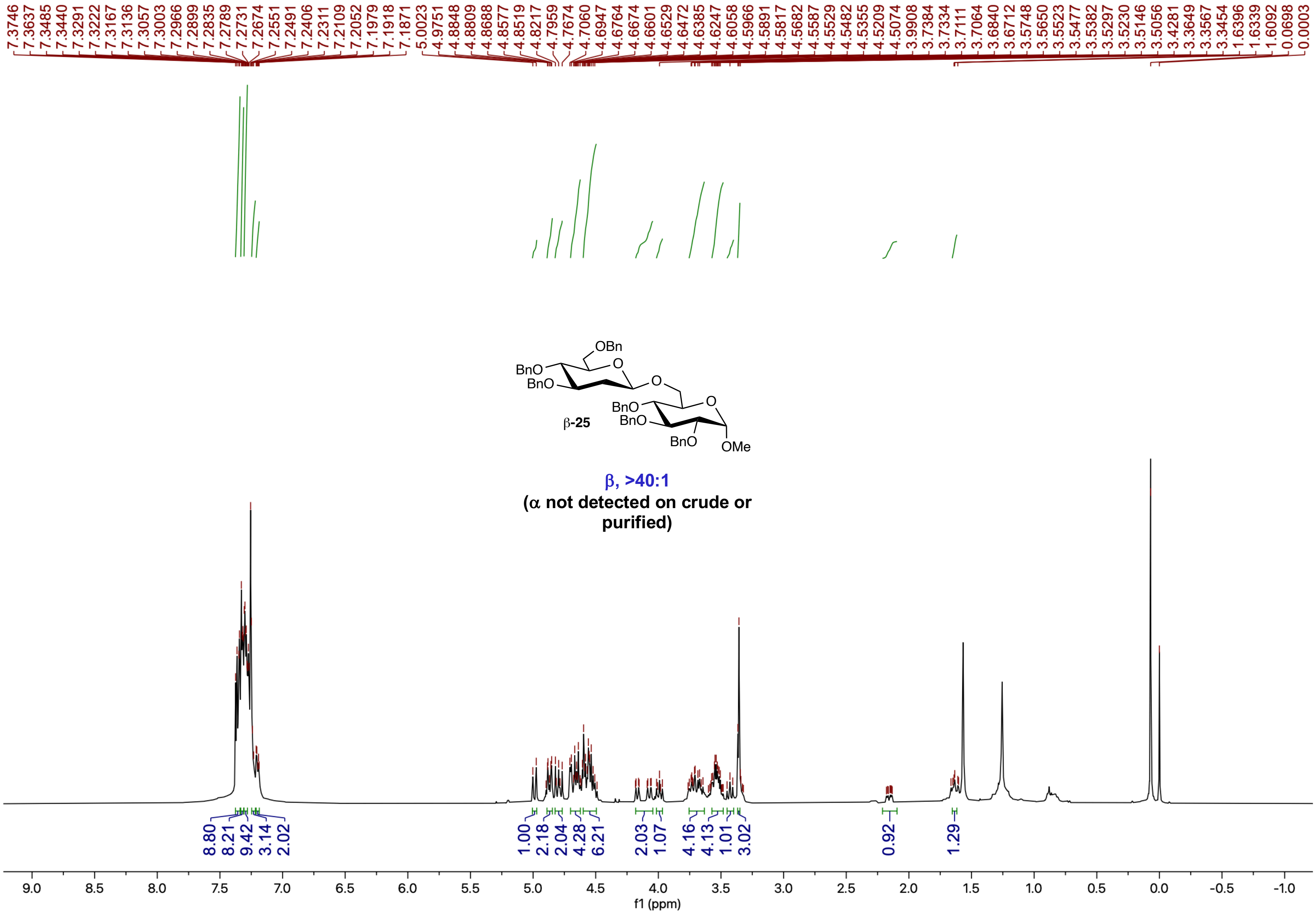

Scheme 4, Entry 15 (α-**26**, from β-**8g**), **purified**
^{1}H NMR, 400 MHz, $CDCl_3$ with 0.03% TMS

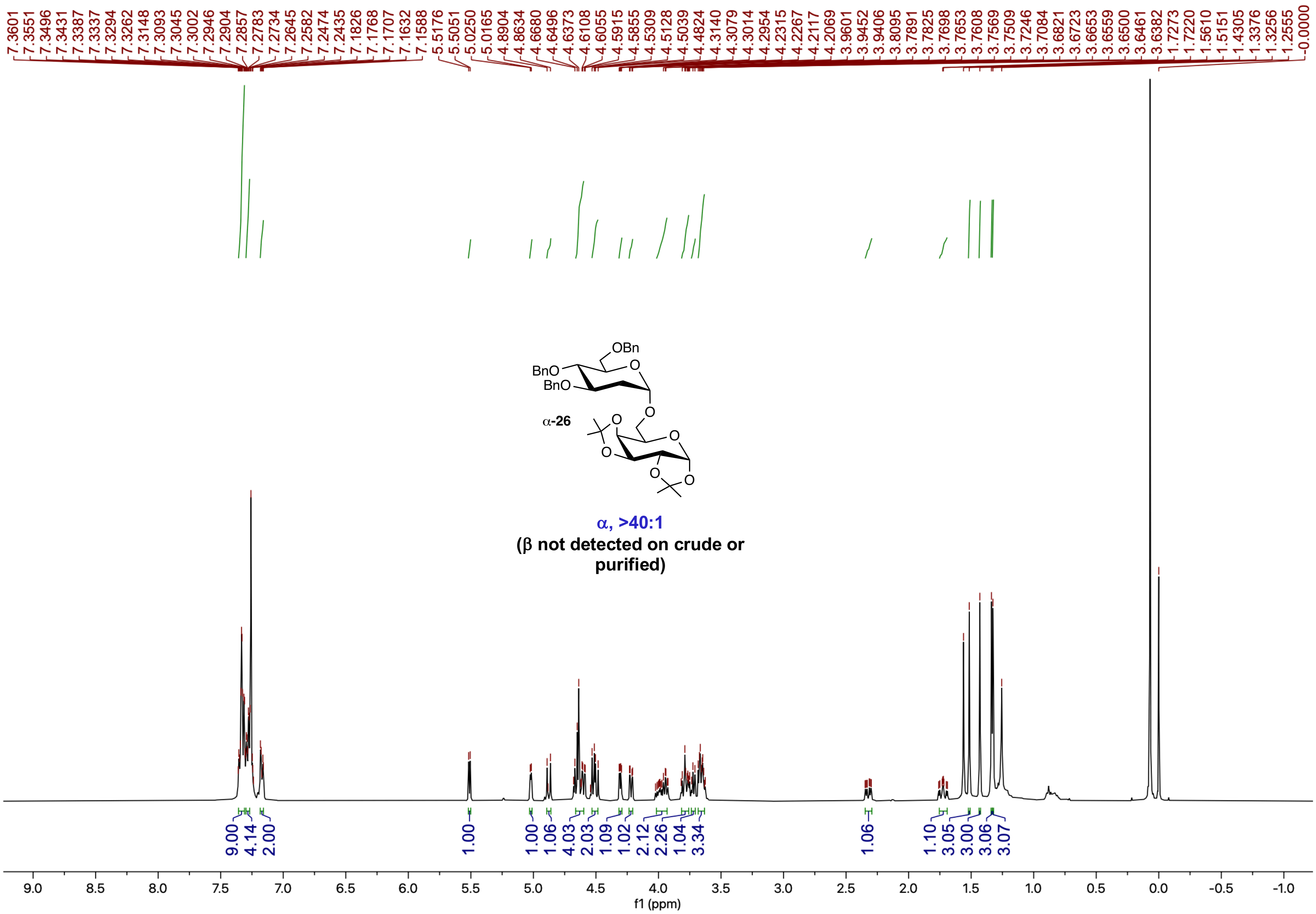

Scheme 4, Entry 16 (β-**26**, from α-**8g**), **purified**
^{1}H NMR, 400 MHz, $CDCl_3$ with 0.03% TMS

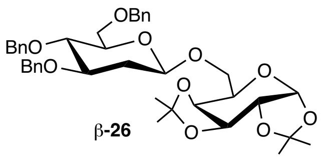

α:β, 1:9

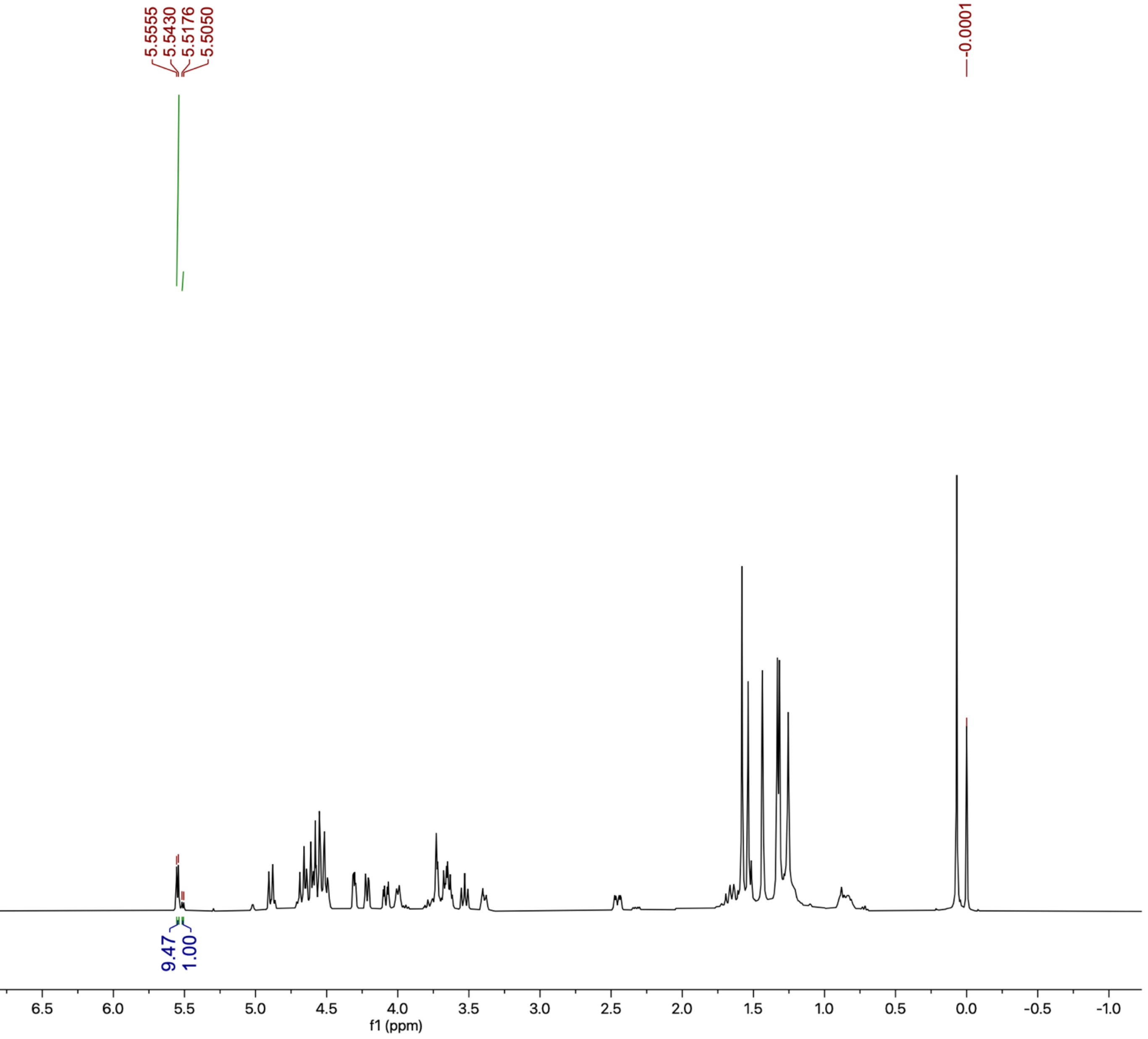

Scheme 4, Entry 17 (**27**, from **8h**), **purified**
^{1}H NMR, 400 MHz, $CDCl_3$ with 0.03% TMS

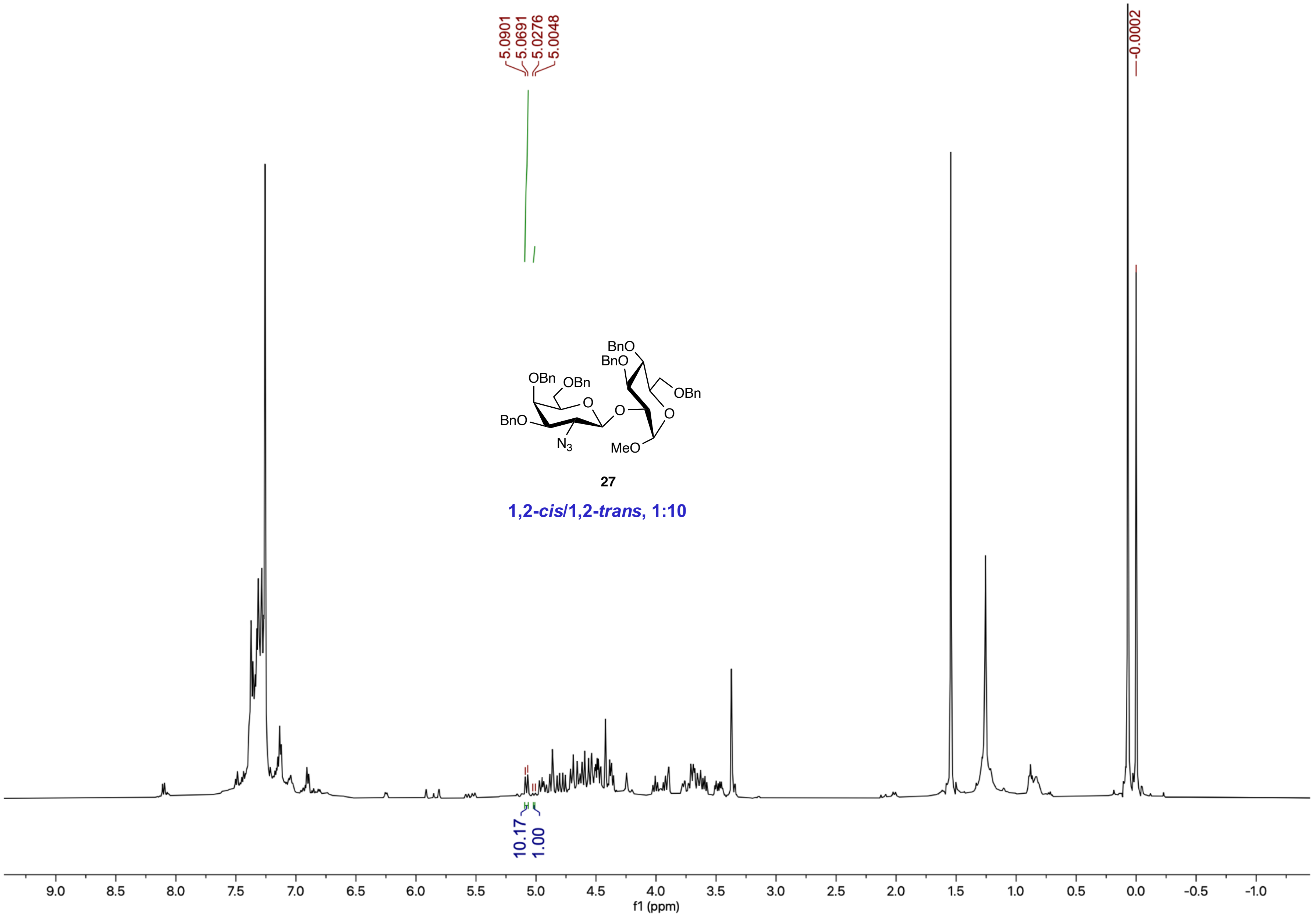

Scheme 4, Entry 18 (**28**, from **8h**), **purified**
^{1}H NMR, 400 MHz, $CDCl_3$ with 0.03% TMS

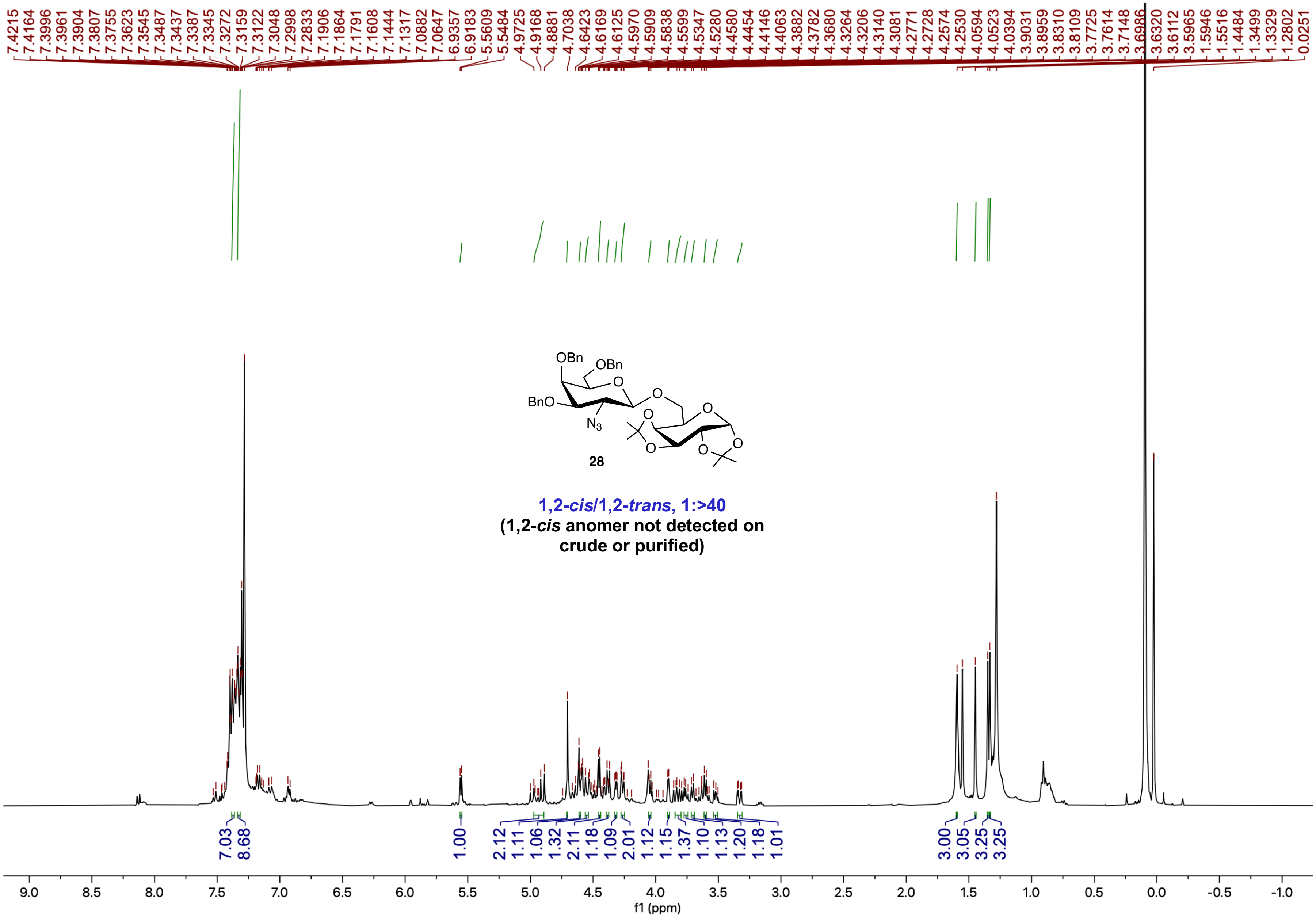

Scheme 3, 1 mmol scale *O*-glycosylation (**18**, from **8d**), **purified**

^{1}H NMR, 400 MHz, $CDCl_3$ with 0.03% TMS

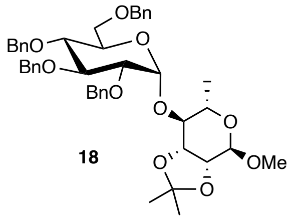

1,2-*cis*/1,2-*trans*, >40:1

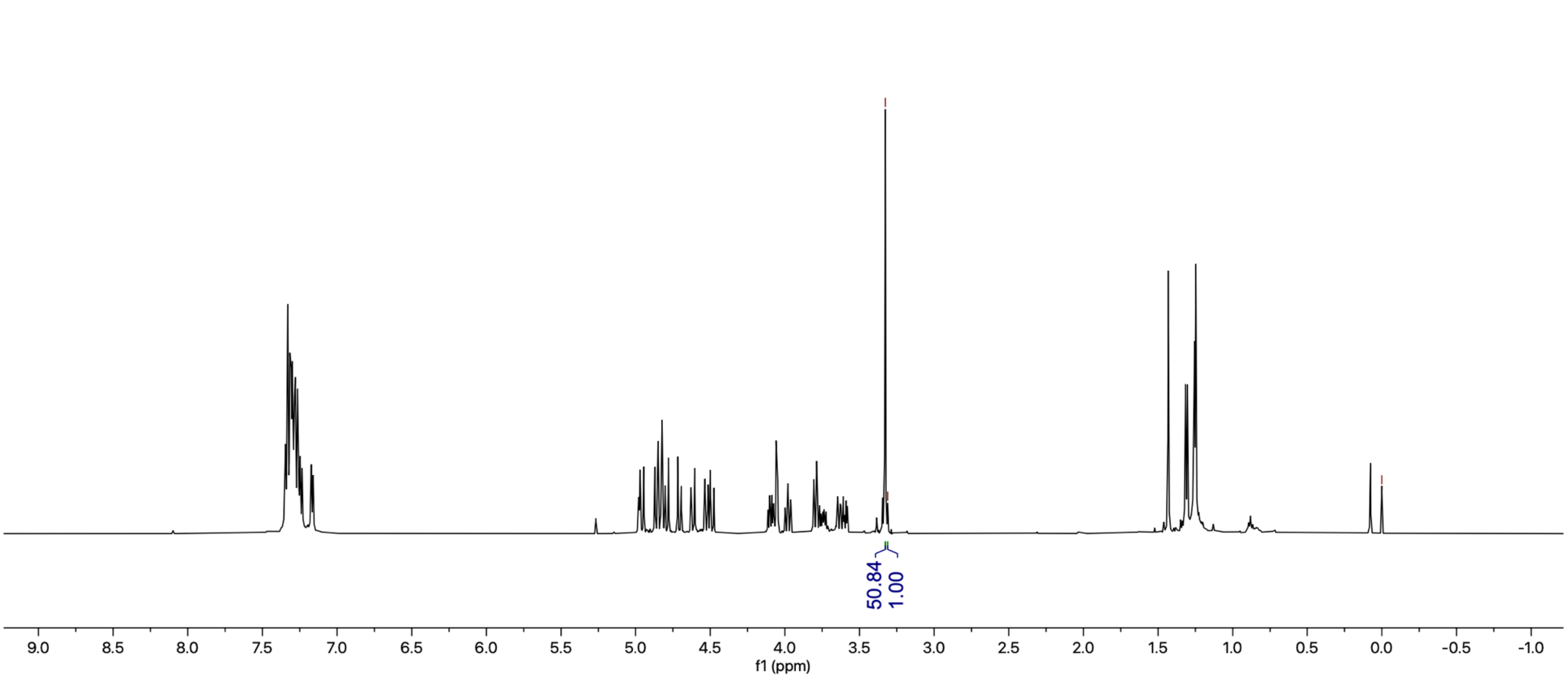

^{1}H NMR Estimation of Anomeric Ratios for the Optimization Study – Crude Reaction Mixtures

Scheme 2, Entry 1, crude

^{1}H NMR, 400 MHz, $CDCl_3$ with 0.03% TMS

	ppm	Hz	Intensity	Width	Area
1	3.3470	1673.9	41.6	1.10	462.61
2	3.3223	1661.6	8.2	1.16	80.55

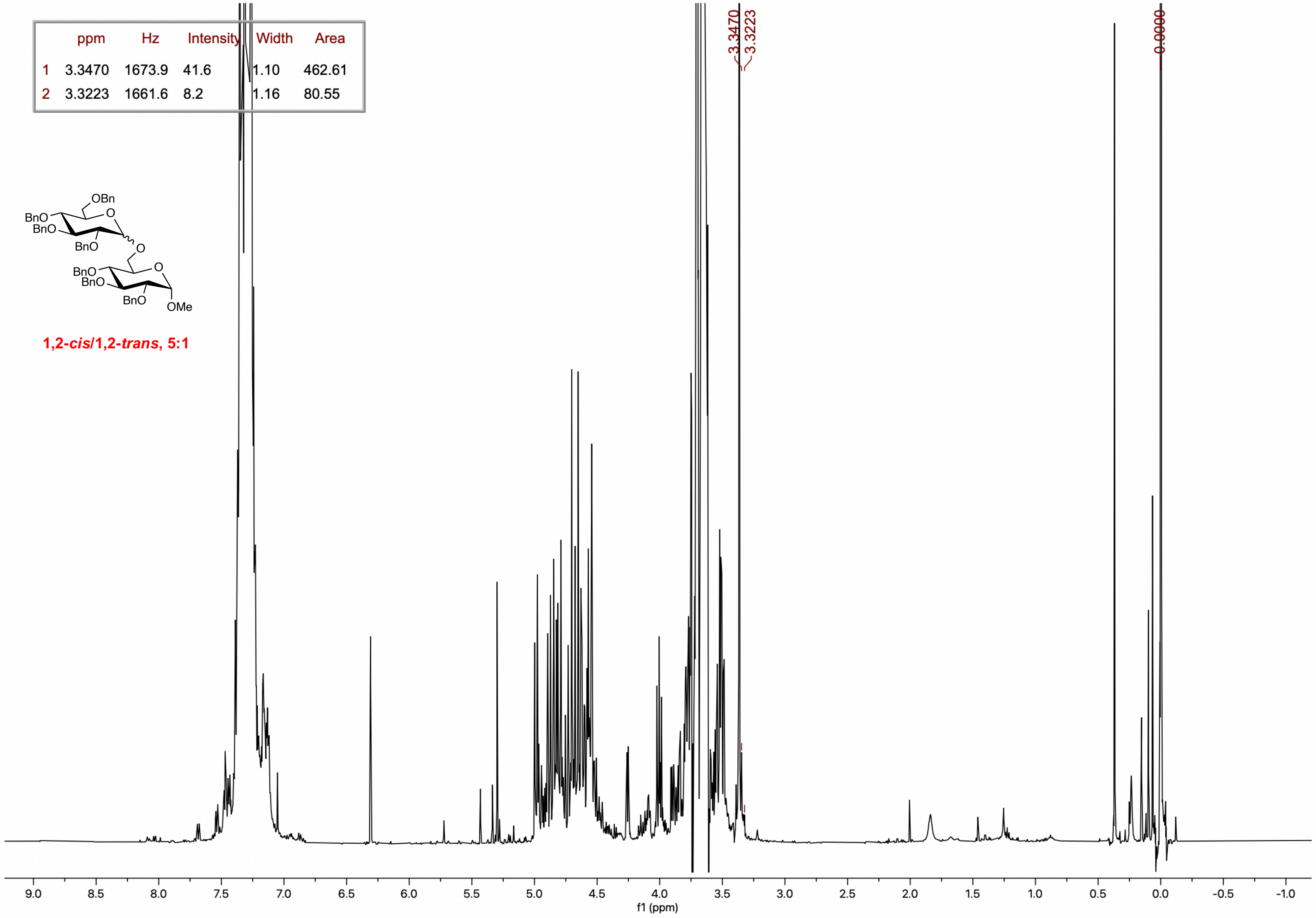

1,2-*cis*/1,2-*trans*, 5:1

Scheme 2, Entry 2, crude

^{1}H NMR, 400 MHz, $CDCl_3$ with 0.03% TMS

	ppm	Hz	Intensity	Width	Area
1	3.3469	1339.2	131.5	1.34	2297.73
2	3.3217	1329.1	35.6	1.82	906.38

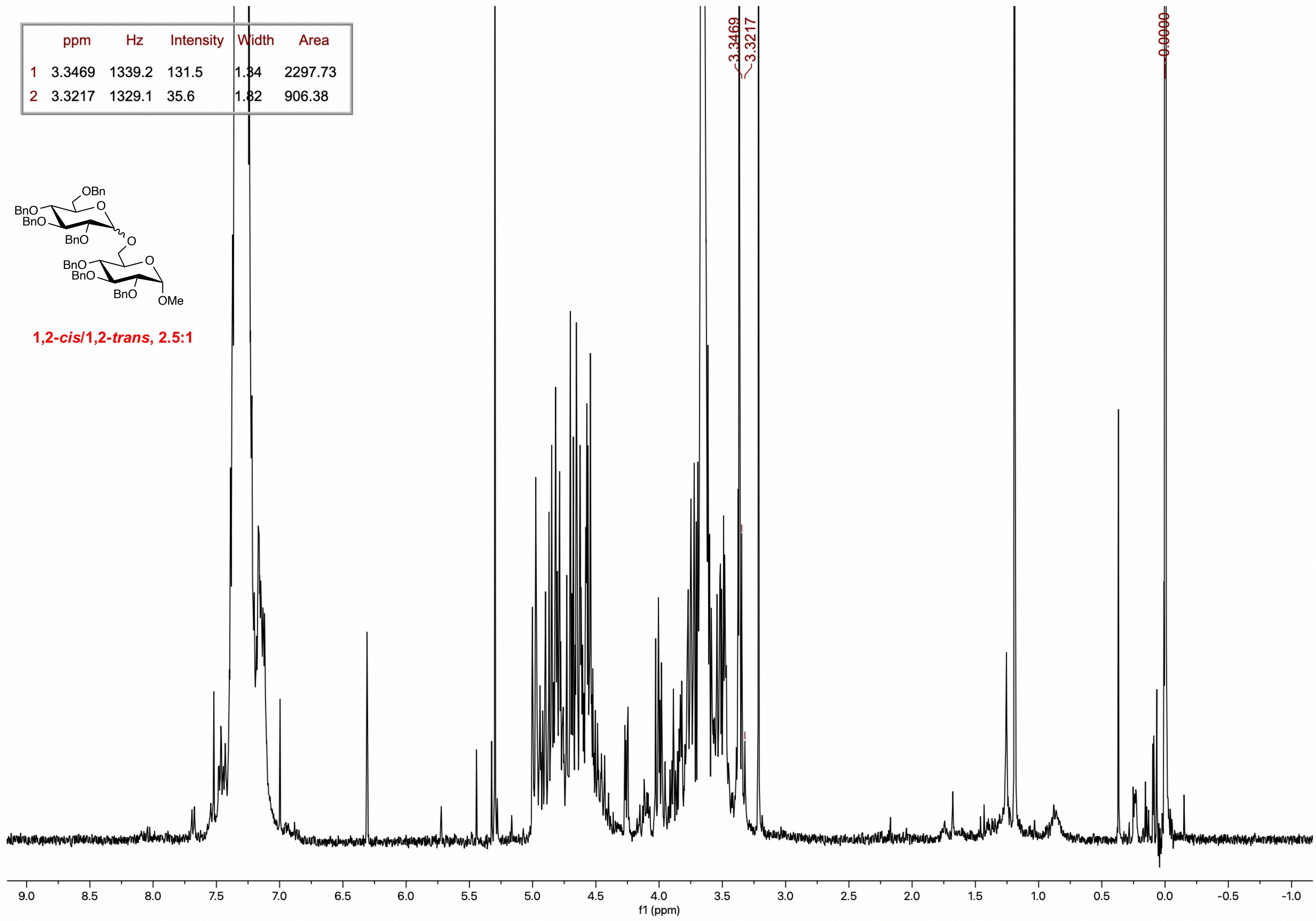

1,2-*cis*/1,2-*trans*, 2.5:1

Scheme 2, Entry 4, crude

^{1}H NMR, 400 MHz, $CDCl_3$ with 0.03% TMS

	ppm	Hz	Intensity	Width	Area
1	3.3468	1673.8	15.0	1.31	170.61
2	3.3218	1661.3	45.0	1.52	657.36

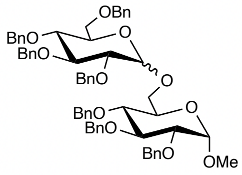

1,2-*cis*/1,2-*trans*, 1:4

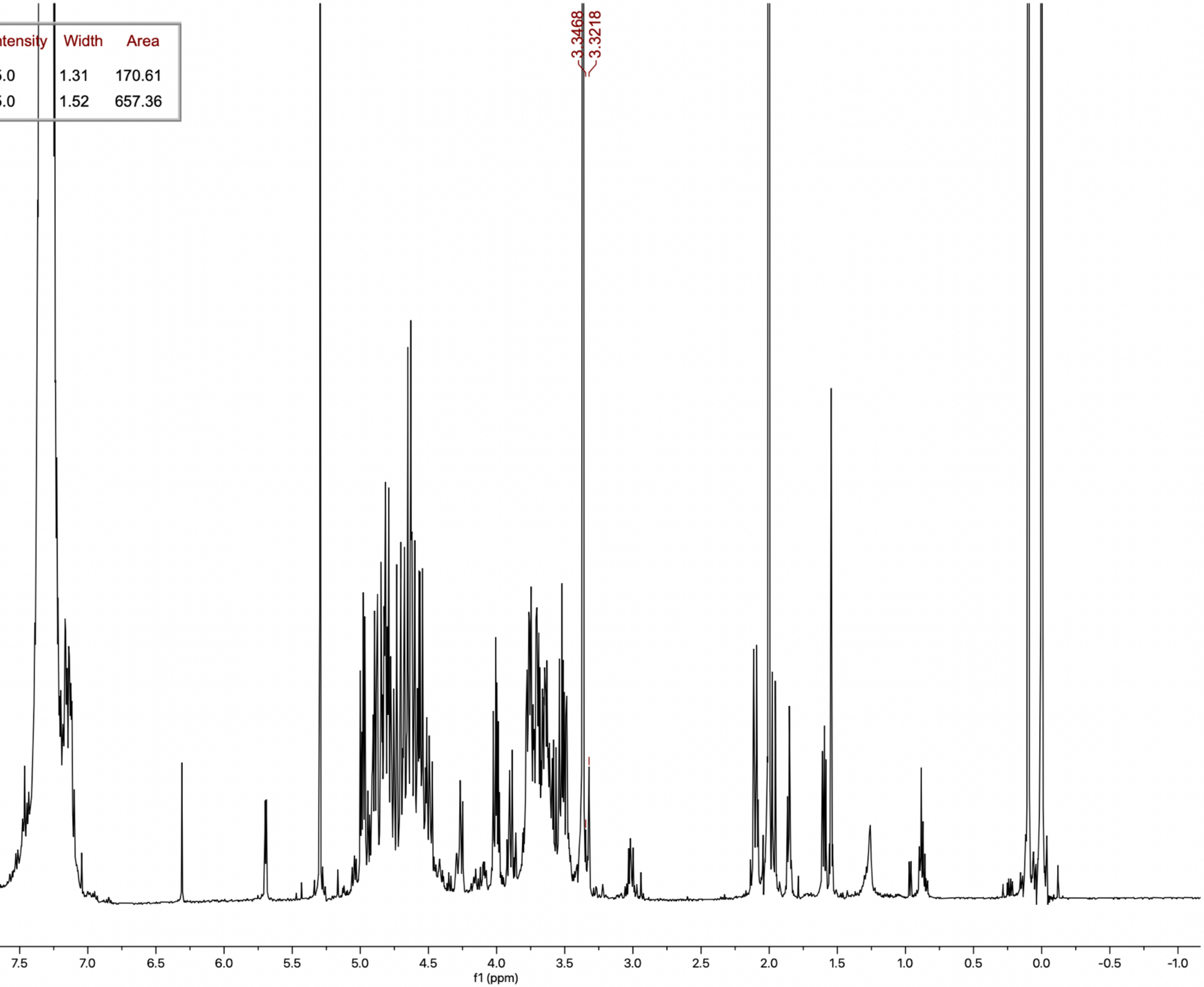

Scheme 2, Entry 5, crude

^{1}H NMR, 400 MHz, $CDCl_3$ with 0.03% TMS

	ppm	Hz	Intensity	Width	Area
1	3.3468	1673.9	216.4	1.23	2750.57
2	3.3218	1661.3	68.1	1.37	964.34

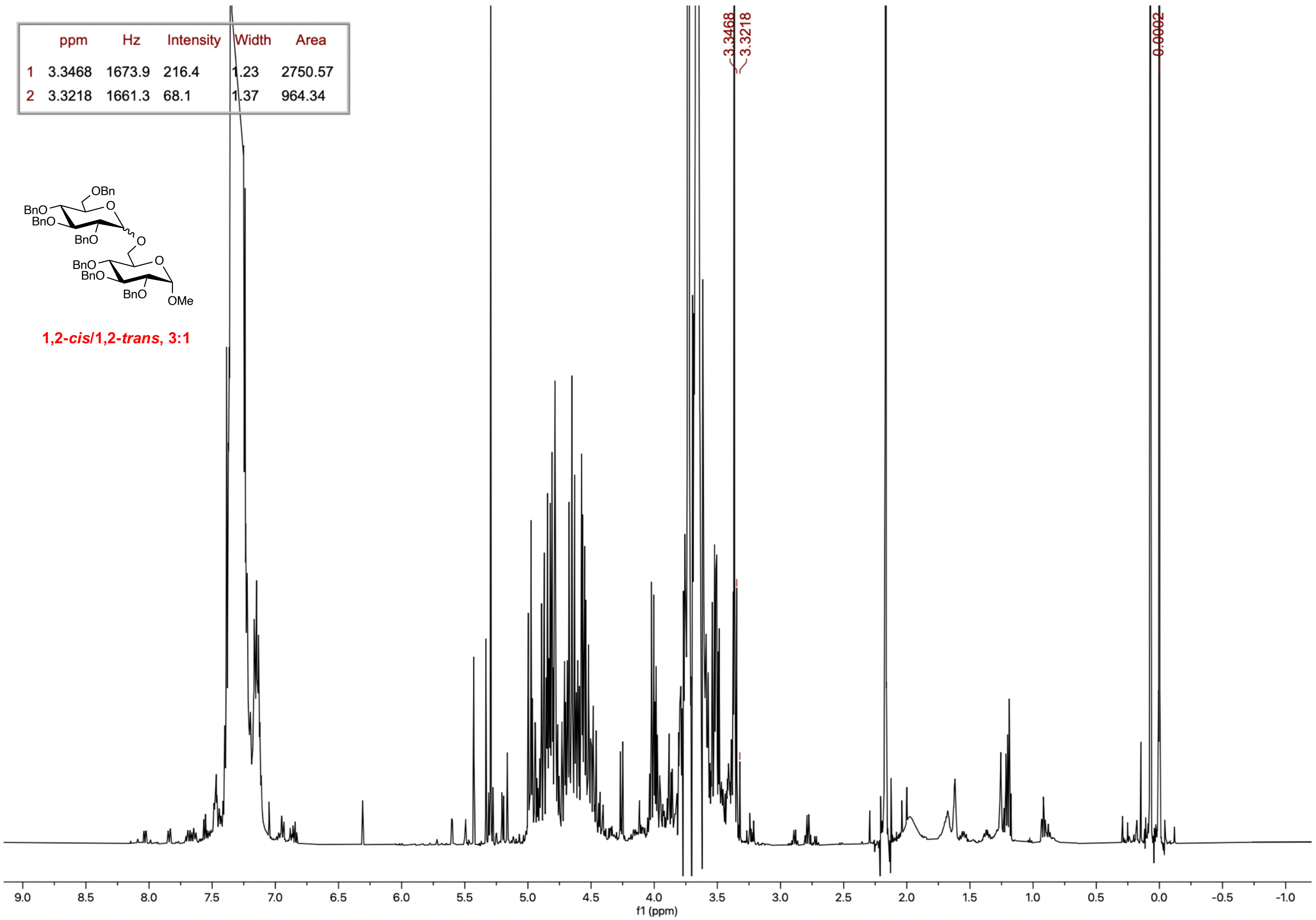

1,2-*cis*/1,2-*trans*, 3:1

Scheme 2, Entry 6, crude

^{1}H NMR, 400 MHz, $CDCl_3$ with 0.03% TMS

	ppm	Hz	Intensity	Width	Area
1	3.3471	1674.0	66.3	1.51	1031.18
2	3.3221	1661.5	22.6	1.61	386.48

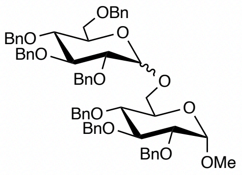

1,2-*cis*/1,2-*trans*, 3:1

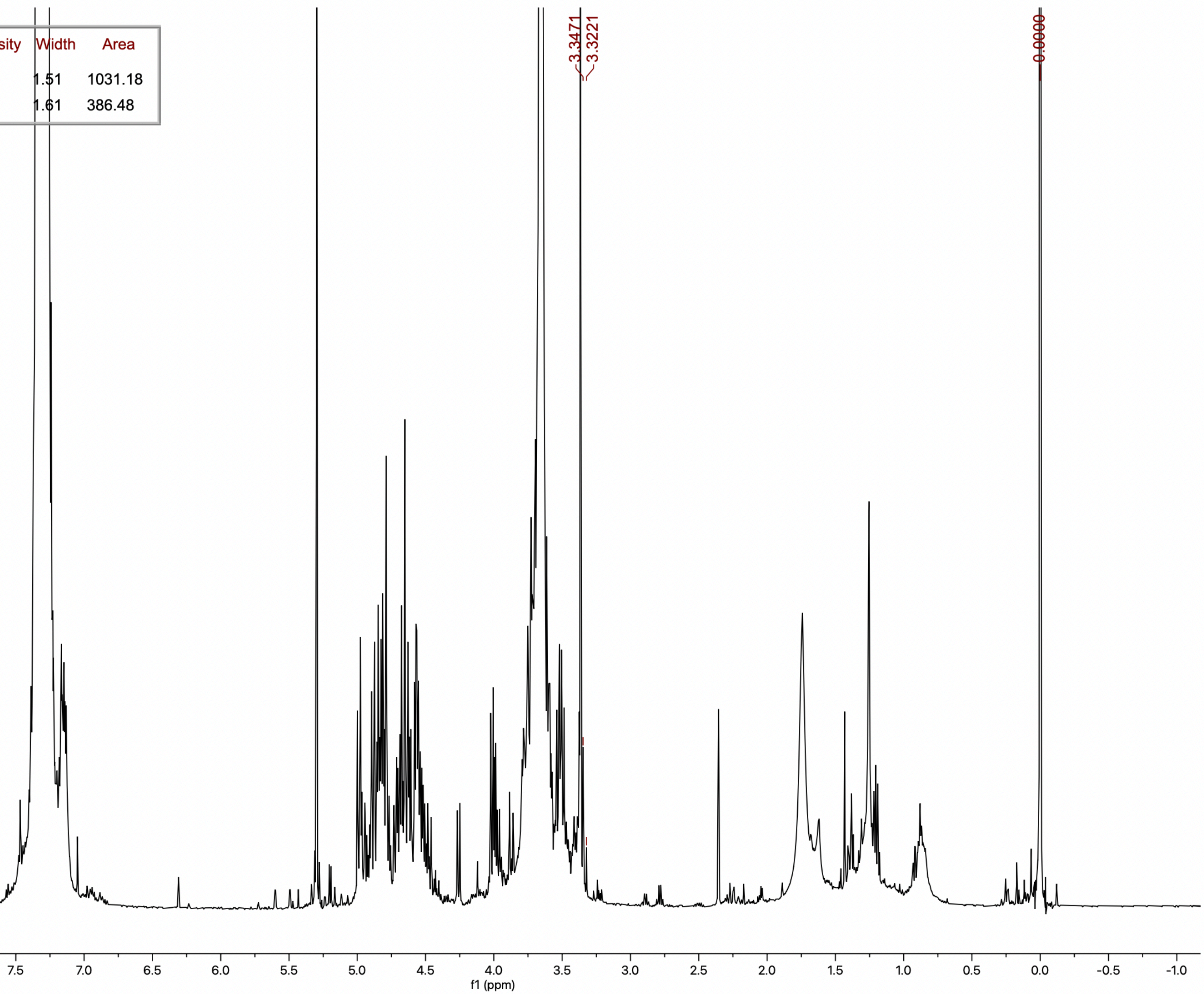

Scheme 2, Entry 7, crude

^{1}H NMR, 400 MHz, $CDCl_3$ with 0.03% TMS

	ppm	Hz	Intensity	Width	Area
1	3.3468	1673.8	253.4	2.20	5493.58
2	3.3222	1661.5	16.1	2.21	364.70

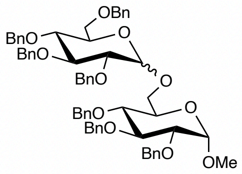

1,2-*cis*/1,2-*trans*, 15:1

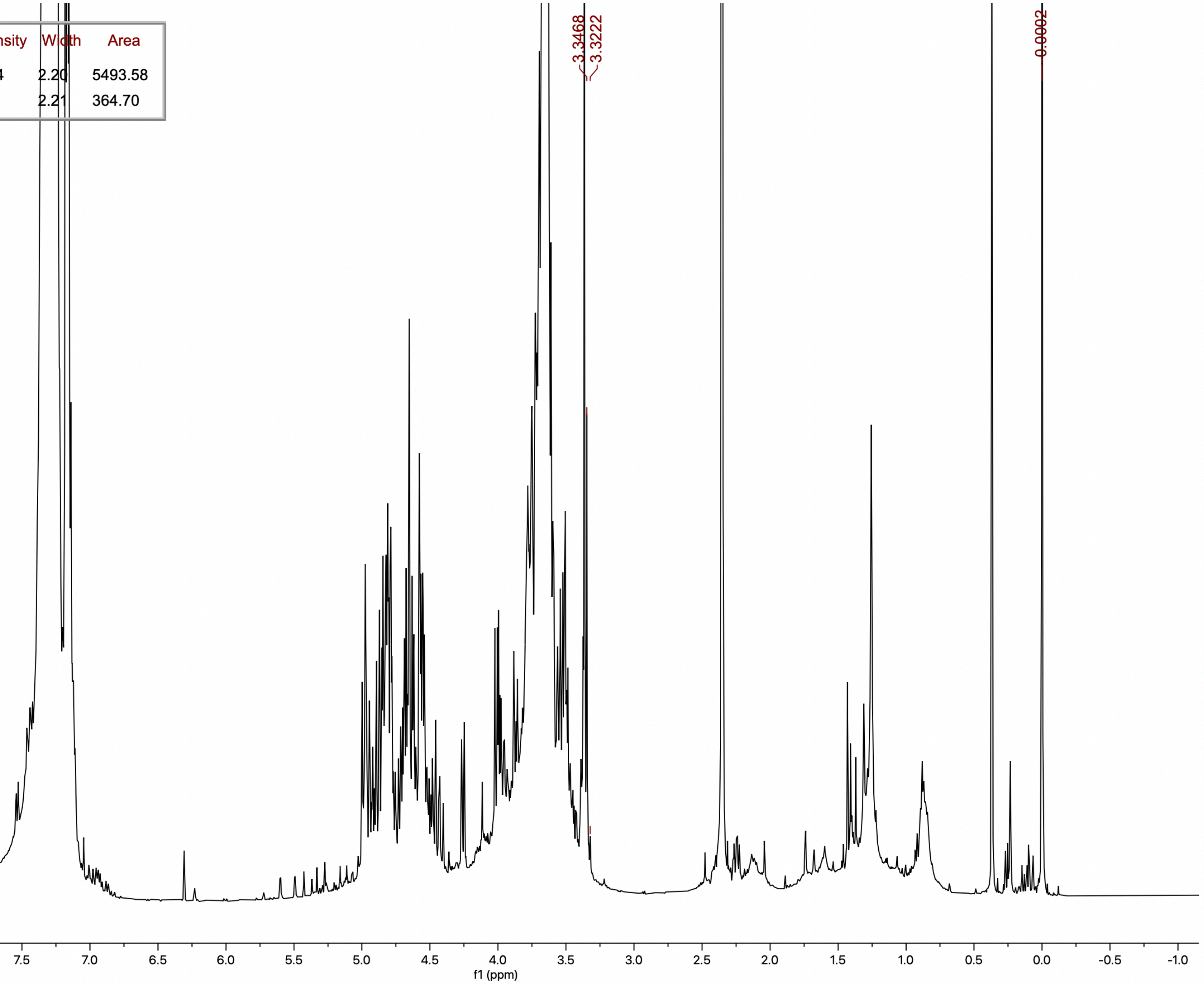

Scheme 2, Entry 8, crude

^{1}H NMR, 400 MHz, $CDCl_3$ with 0.03% TMS

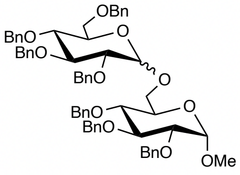

1,2-cis/1,2-trans, 15:1

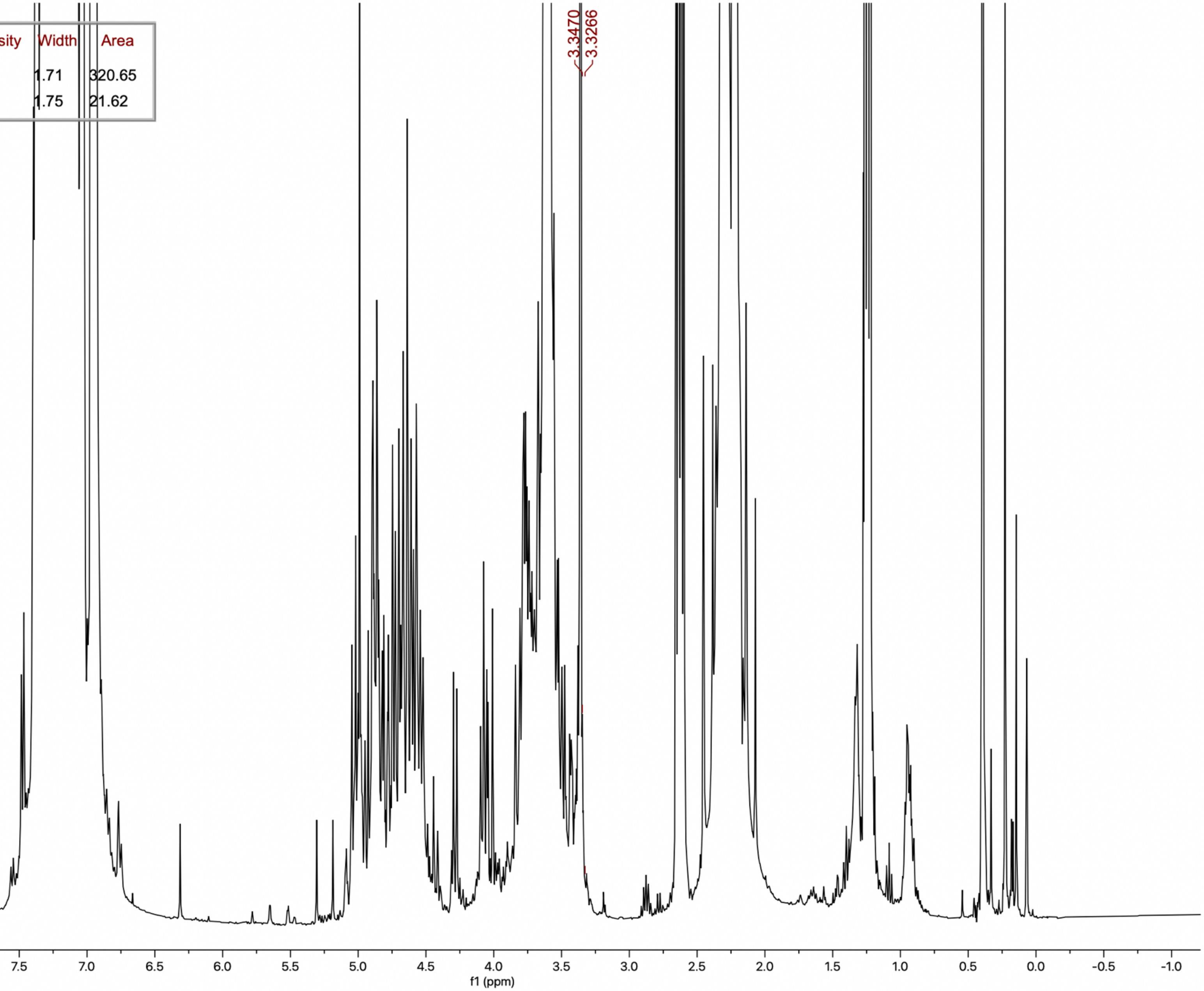

Scheme 2, Entry 9, crude

^{1}H NMR, 400 MHz, $CDCl_3$ with 0.03% TMS

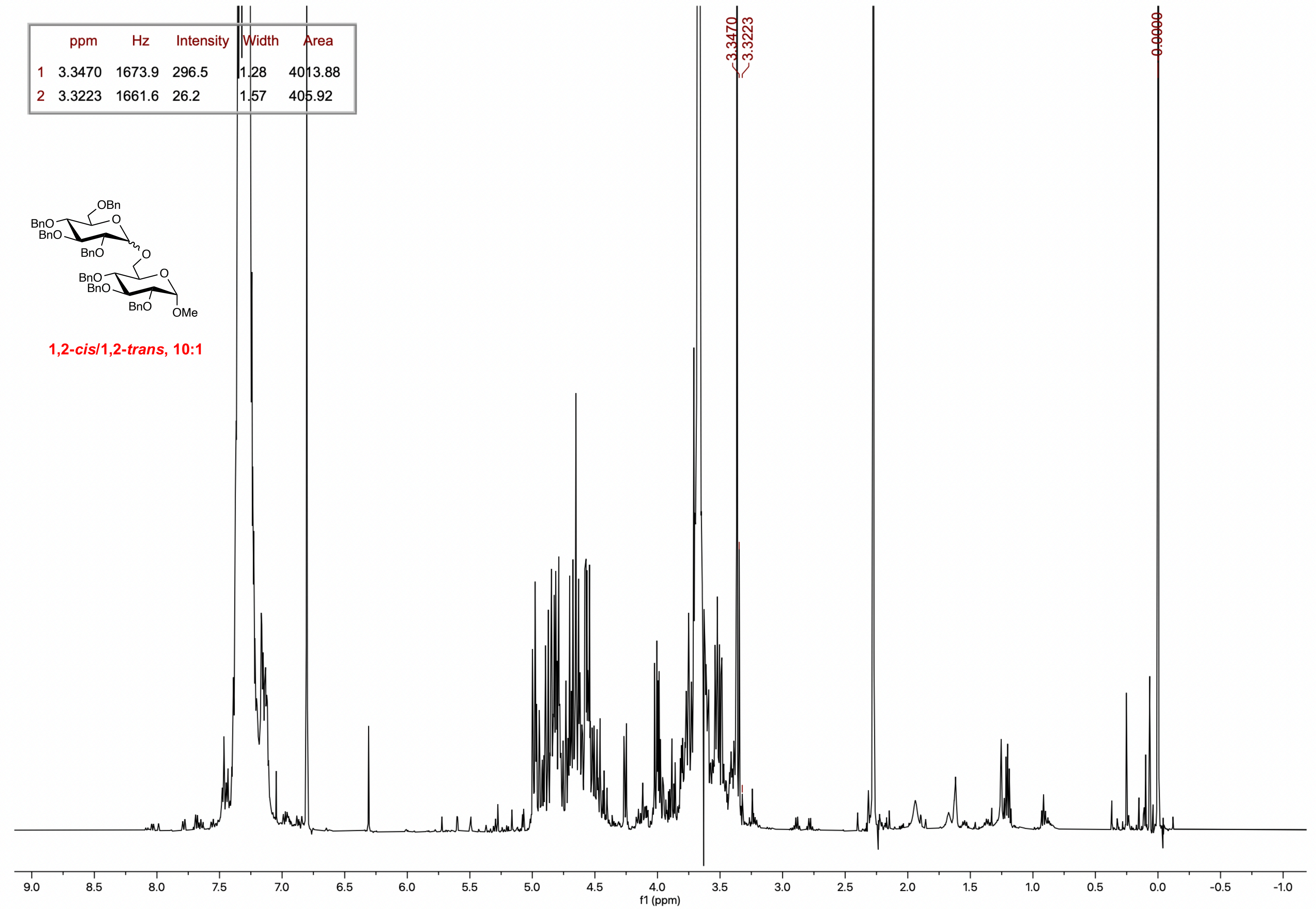

	ppm	Hz	Intensity	Width	Area
1	3.3470	1673.9	296.5	1.28	4013.88
2	3.3223	1661.6	26.2	1.57	405.92

1,2-*cis*/1,2-*trans*, 10:1

Scheme 2, Entry 10, crude

^{1}H NMR, 400 MHz, $CDCl_3$ with 0.03% TMS

	ppm	Hz	Intensity	Width	Area
1	3.3470	1339.3	189.7	1.33	3383.68
2	3.3223	1329.4	11.7	1.91	320.30

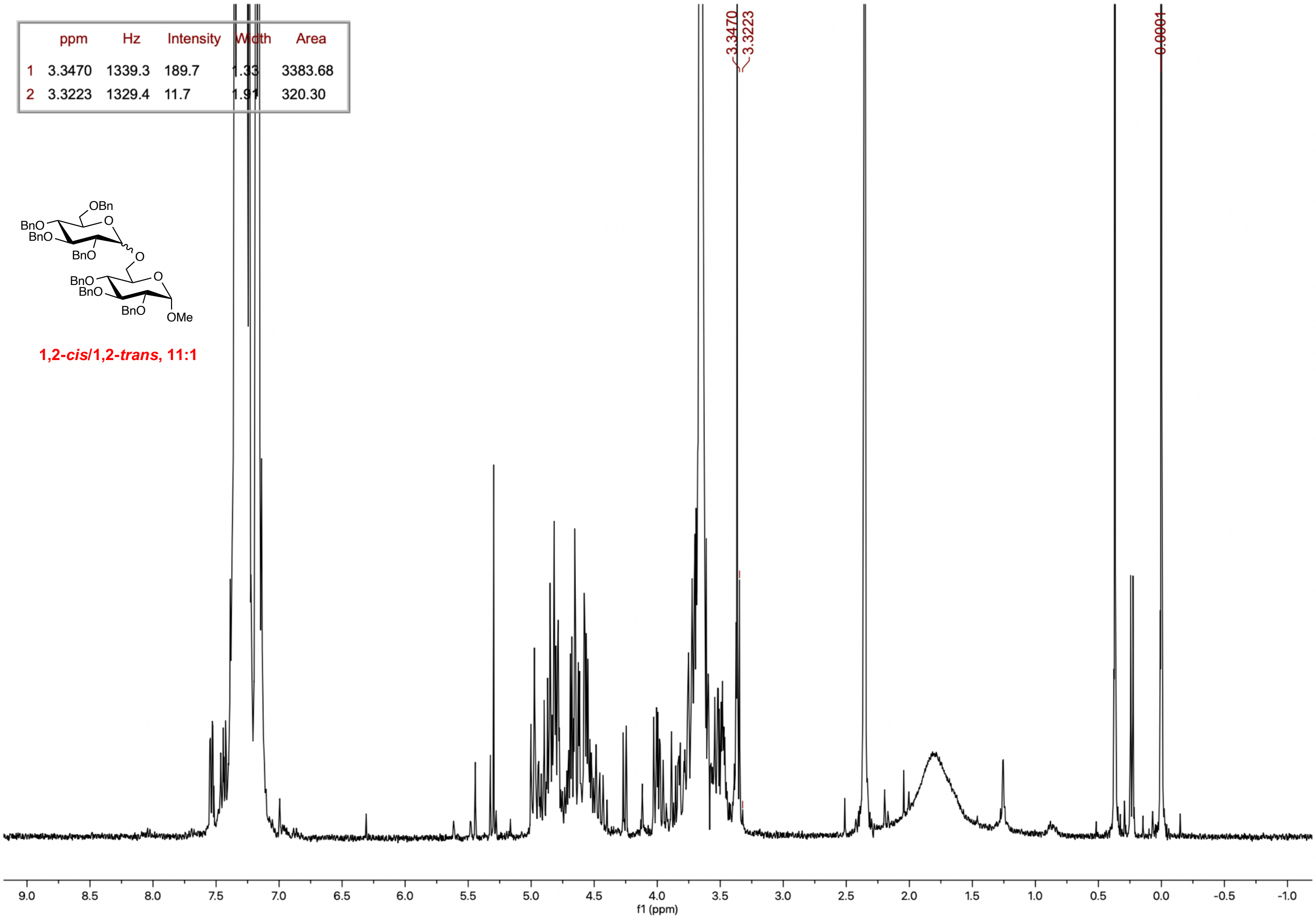

Scheme 2, Entry 12, crude

^{1}H NMR, 400 MHz, $CDCl_3$ with 0.03% TMS

	ppm	Hz	Intensity	Width	Area
1	3.3465	1673.7	18.7	1.35	268.45
2	3.3259	1663.4	4.7	0.45	18.94

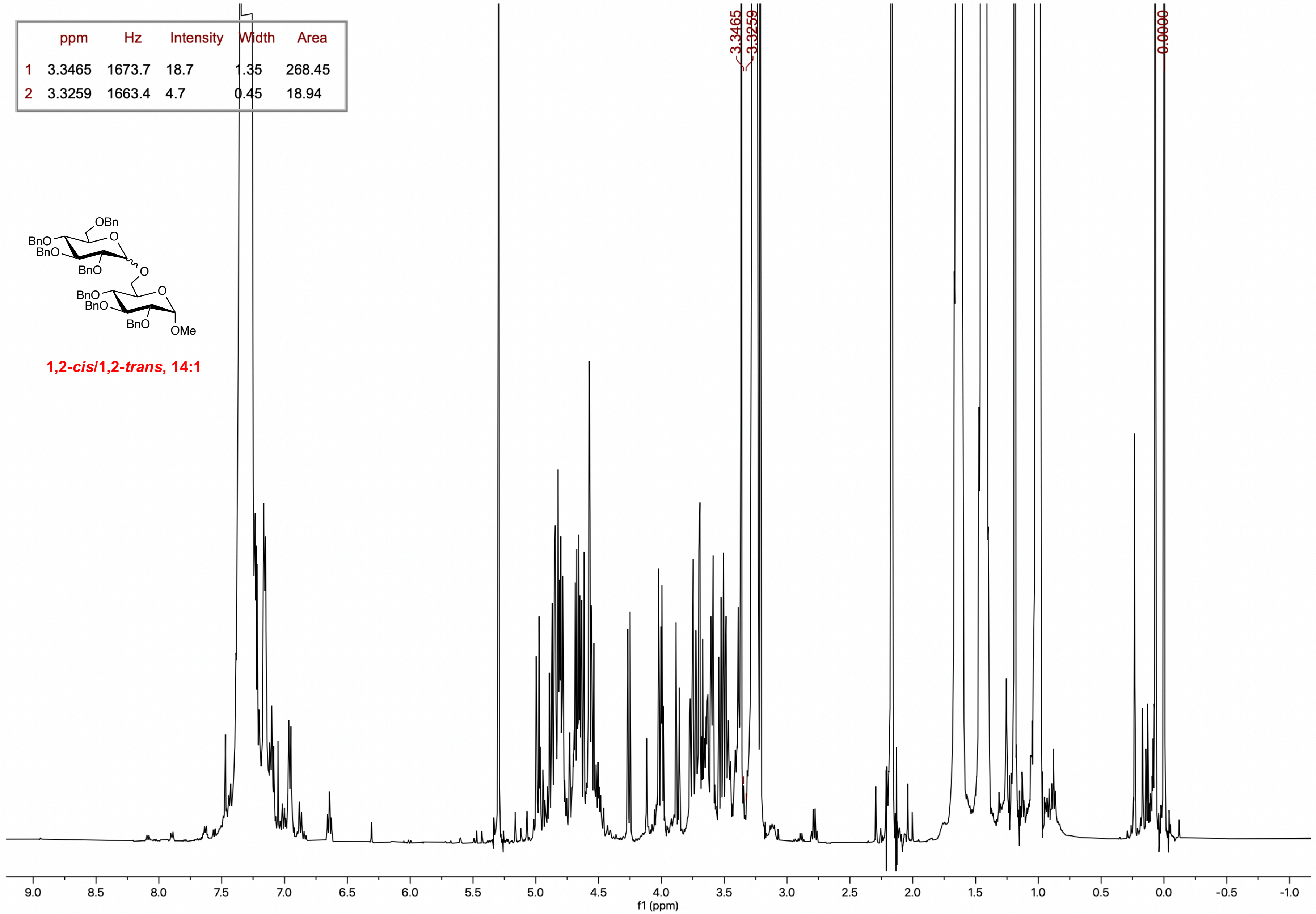

1,2-*cis*/1,2-*trans*, 14:1

Scheme 2, Entry 13, crude

^{1}H NMR, 400 MHz, $CDCl_3$ with 0.03% TMS

	ppm	Hz	Intensity	Width	Area
1	3.3532	1341.7	73.7	1.71	1623.58
2	3.3273	1331.4	6.9	3.42	300.24

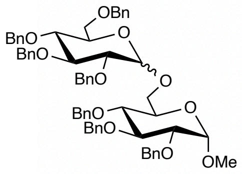

1,2-*cis*/1,2-*trans*, 5:1

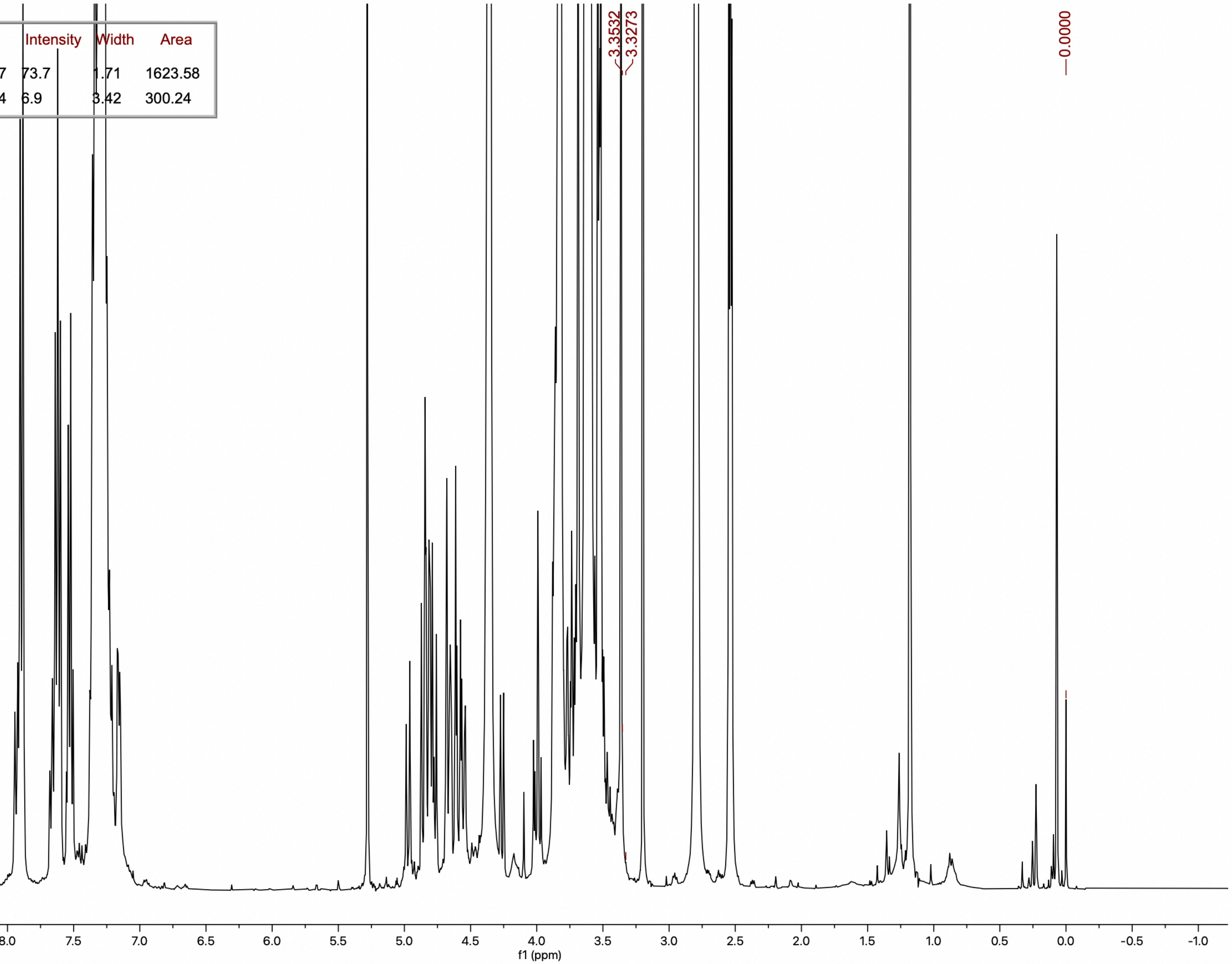

Scheme 2, Entry 16, crude

^{1}H NMR, 400 MHz, $CDCl_3$ with 0.03% TMS

	ppm	Hz	Intensity	Width	Area
1	3.3475	1339.4	197.1	1.23	3175.35
2	3.3224	1329.4	18.0	1.24	287.06

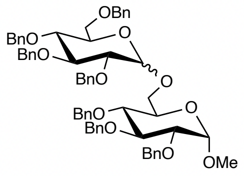

1,2-*cis*/1,2-*trans*, 11:1

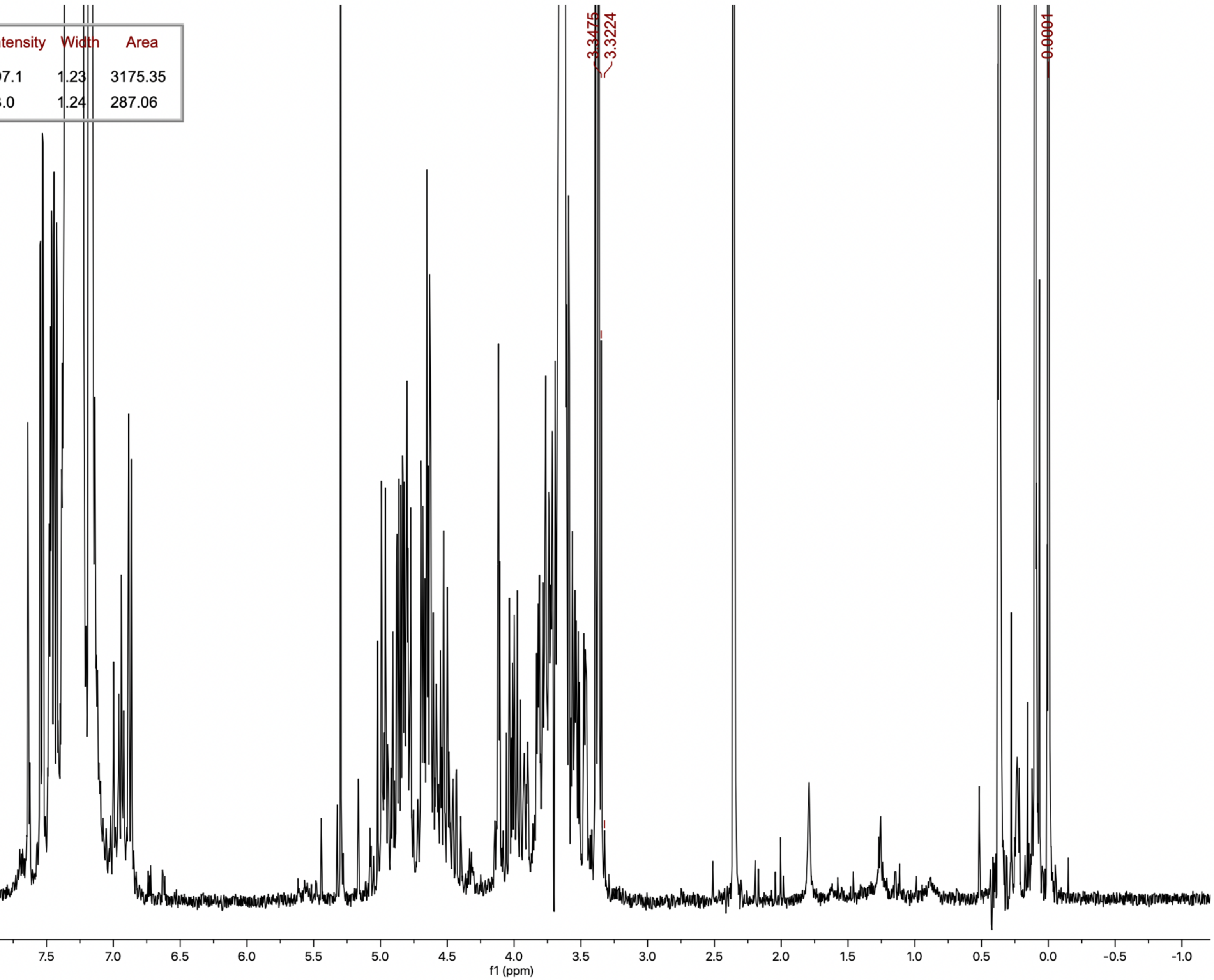

Scheme 2, Entry 17, crude

^{1}H NMR, 400 MHz, $CDCl_3$ with 0.03% TMS

	ppm	Hz	Intensity	Width	Area
1	3.3473	1674.1	220.3	1.34	3035.91
2	3.3221	1661.5	23.1	1.53	401.65

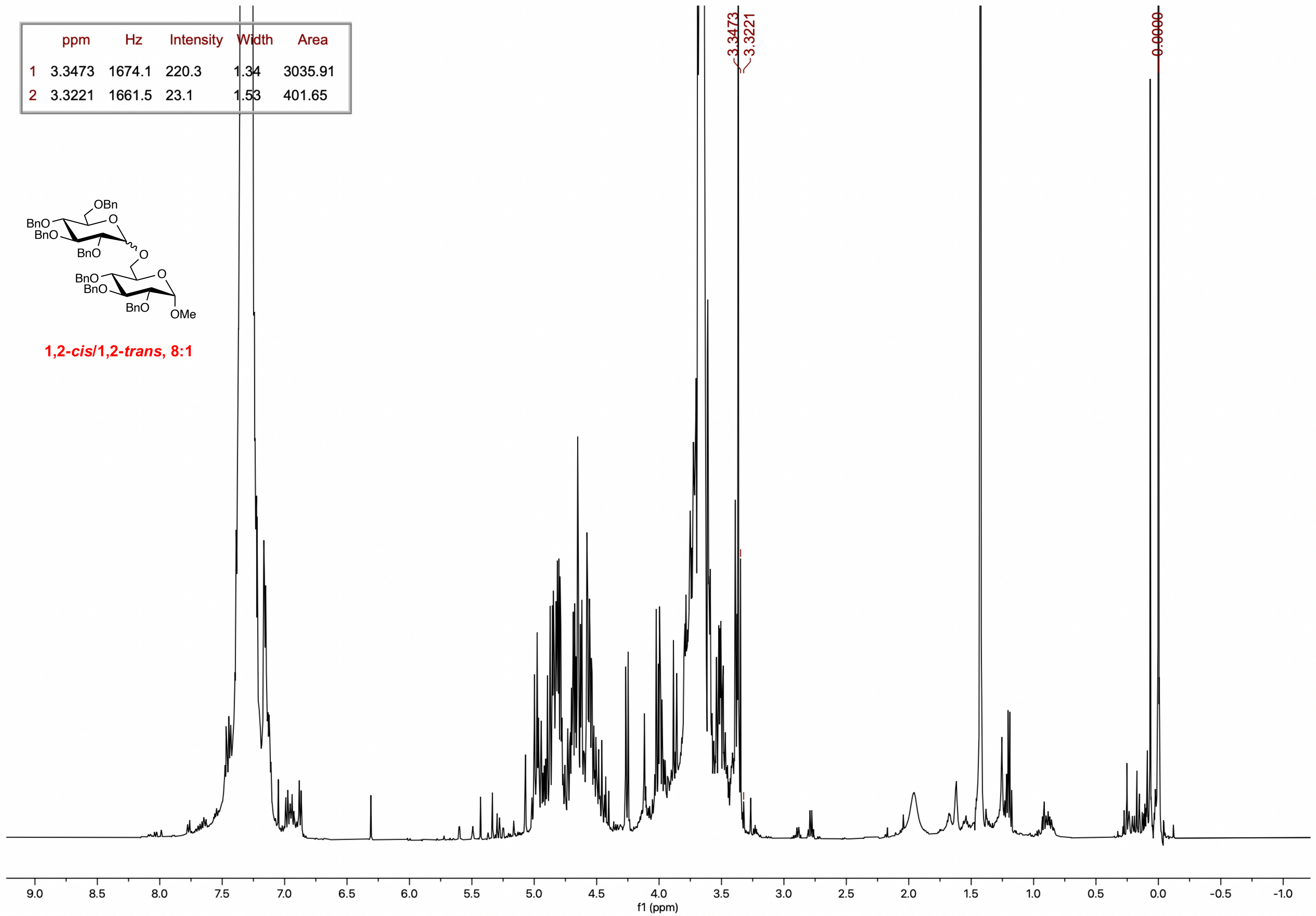

Scheme 2, Entry 18, crude

^{1}H NMR, 400 MHz, $CDCl_3$ with 0.03% TMS

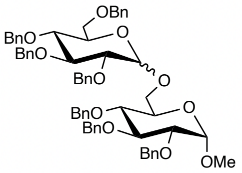

1,2-*cis*/1,2-*trans*, 25:1

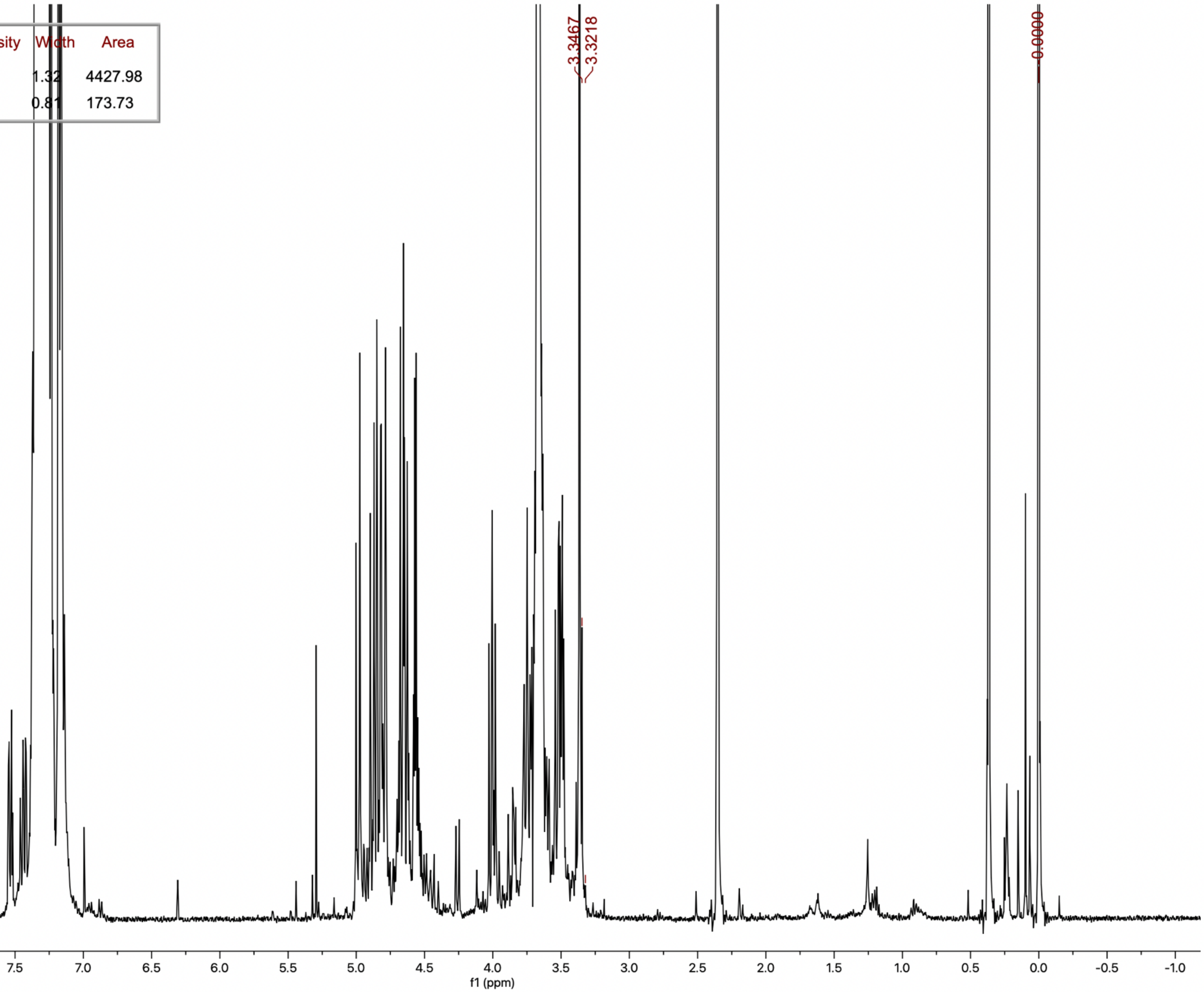

	ppm	Hz	Intensity	Width	Area
1	3.3467	1339.1	255.7	1.32	4427.98
2	3.3218	1329.2	16.9	0.81	173.73

Scheme 2, Entry 19, crude

^{1}H NMR, 400 MHz, $CDCl_3$ with 0.03% TMS

	ppm	Hz	Intensity	Width	Area
1	3.3459	1338.8	88.9	1.53	1791.92
2	3.3206	1328.7	9.6	1.37	161.70

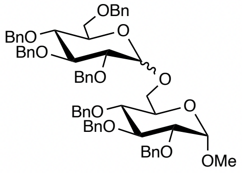

1,2-*cis*/1,2-*trans*, 11:1

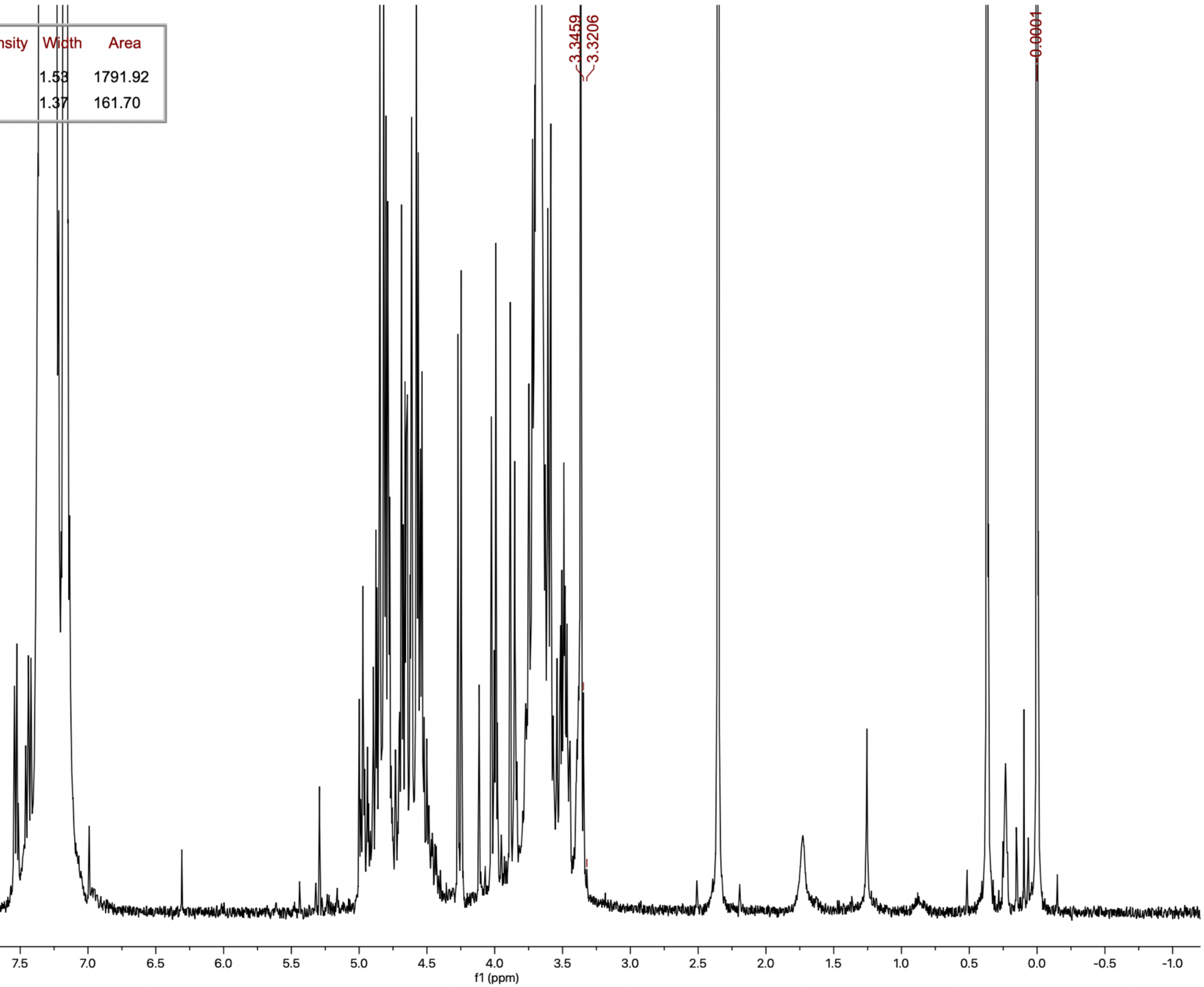

Scheme 2, Entry 21, crude

^{1}H NMR, 400 MHz, $CDCl_3$ with 0.03% TMS

	ppm	Hz	Intensity	Width	Area
1	3.3466	1339.1	620.9	2.88	19043.42
2	3.3221	1329.3	60.0	5.94	3841.21

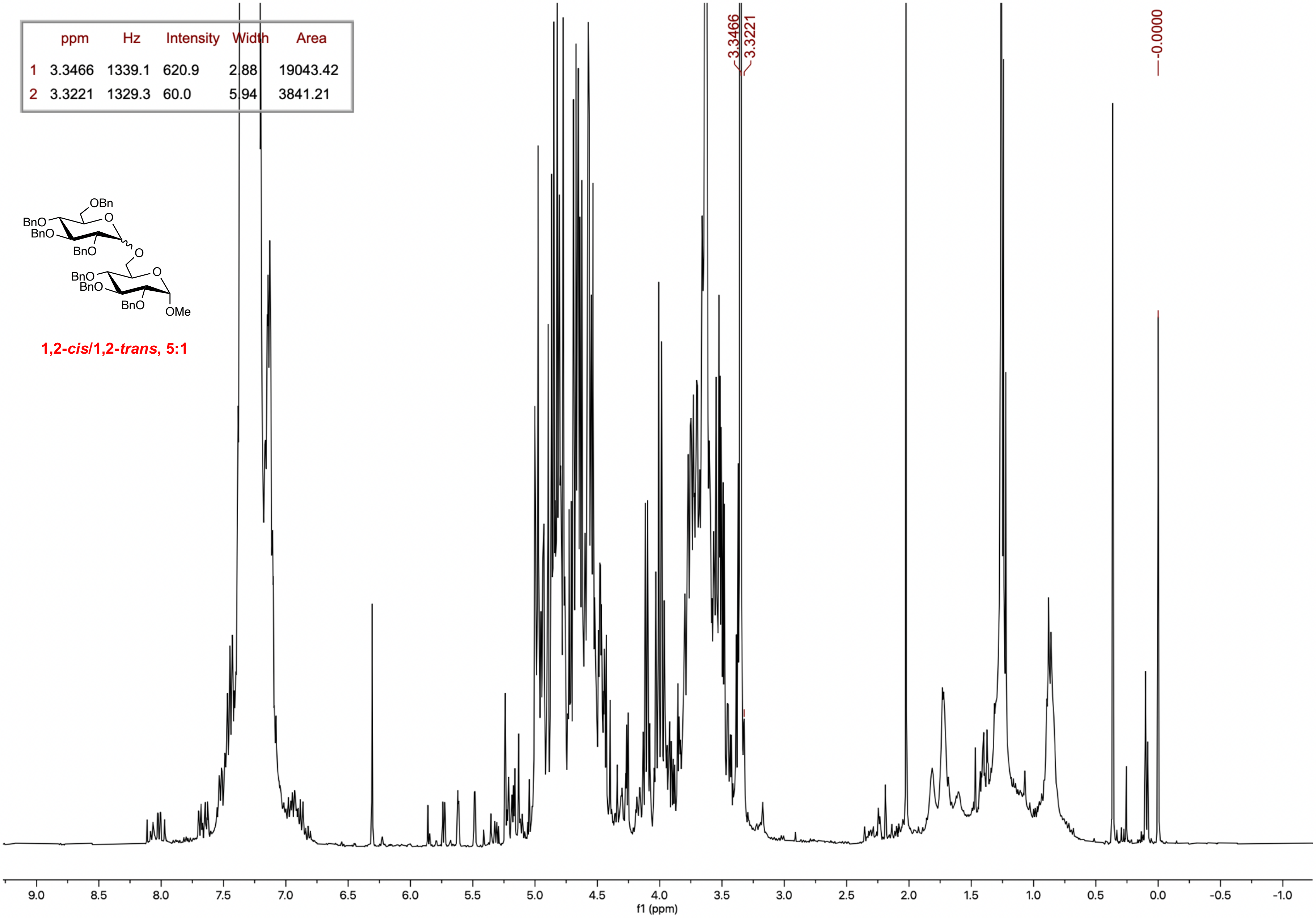

1,2-*cis*/1,2-*trans*, 5:1

Scheme 2, Entry 22, crude

^{1}H NMR, 400 MHz, $CDCl_3$ with 0.03% TMS

	ppm	Hz	Intensity	Width	Area
1	3.3460	1673.4	99.9	1.97	1579.51
2	3.3254	1663.1	9.9	3.09	354.96

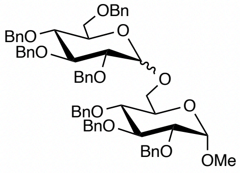

1,2-*cis*/1,2-*trans*, 4:1

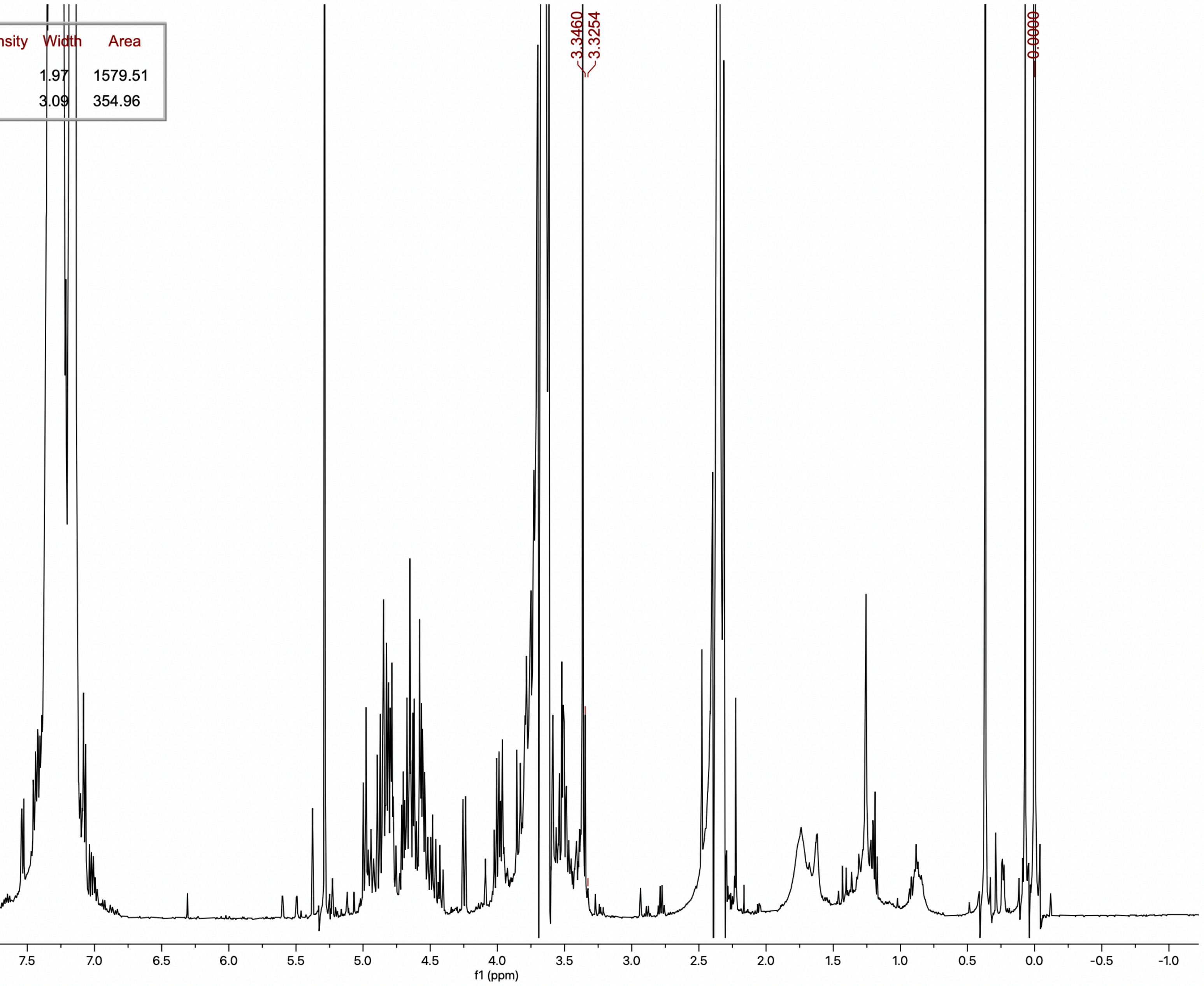

Scheme 2, Entry 23, crude

^{1}H NMR, 400 MHz, $CDCl_3$ with 0.03% TMS

	ppm	Hz	Intensity	Width	Area
1	3.3468	1339.2	244.3	1.47	4614.04
2	3.3218	1329.2	12.8	1.20	224.20

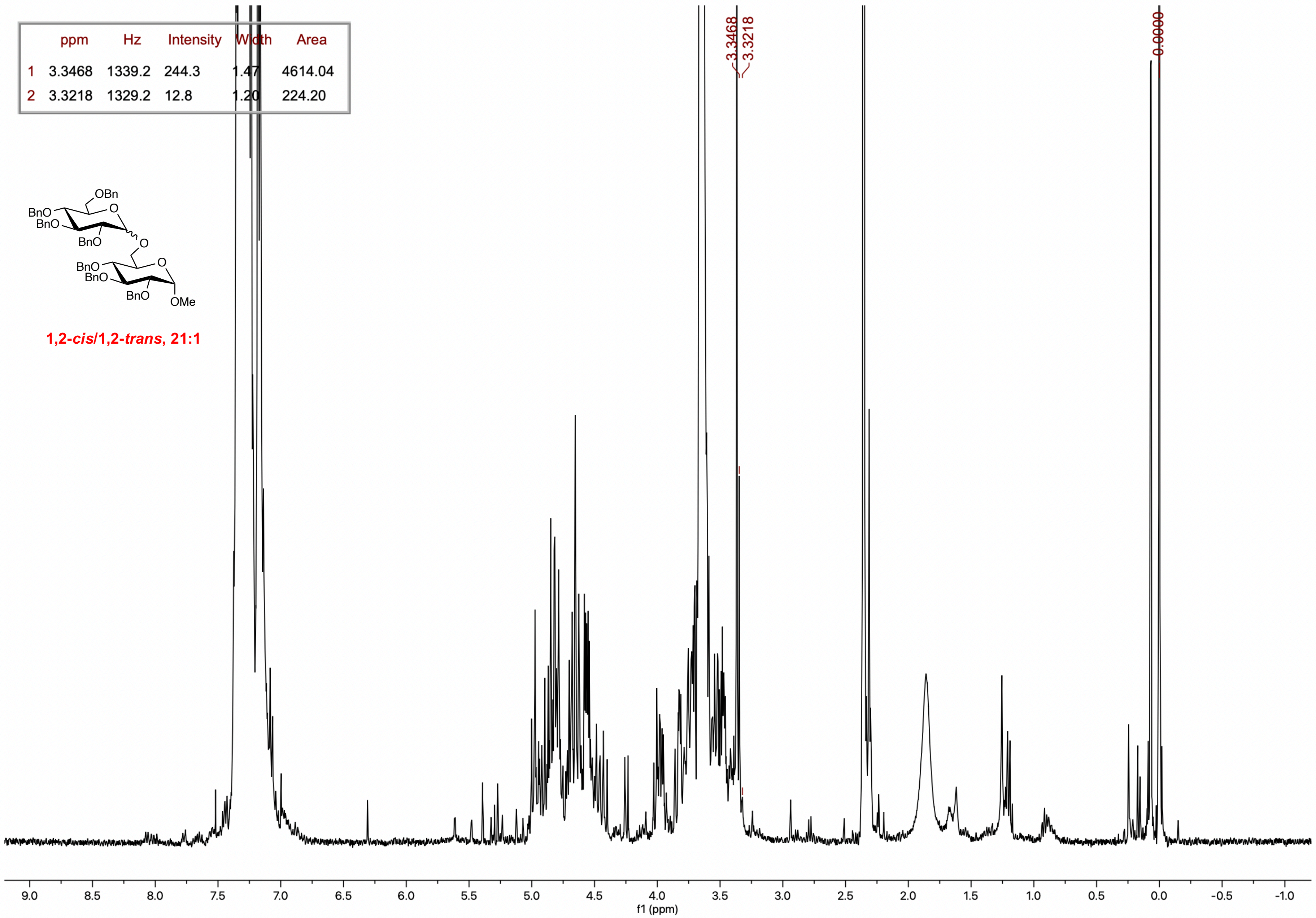

^{1}H NMR Estimation of Anomeric Ratios for the Substrate Scope Study – Crude Reaction Mixtures

Scheme 4, Entry 1 (**13**, from **8a**), **crude**
^{1}H NMR, 400 MHz, $CDCl_3$ with 0.03% TMS

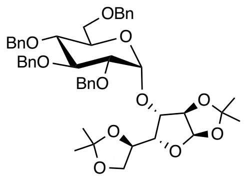

13

1,2-cis/1,2-trans, >40:1

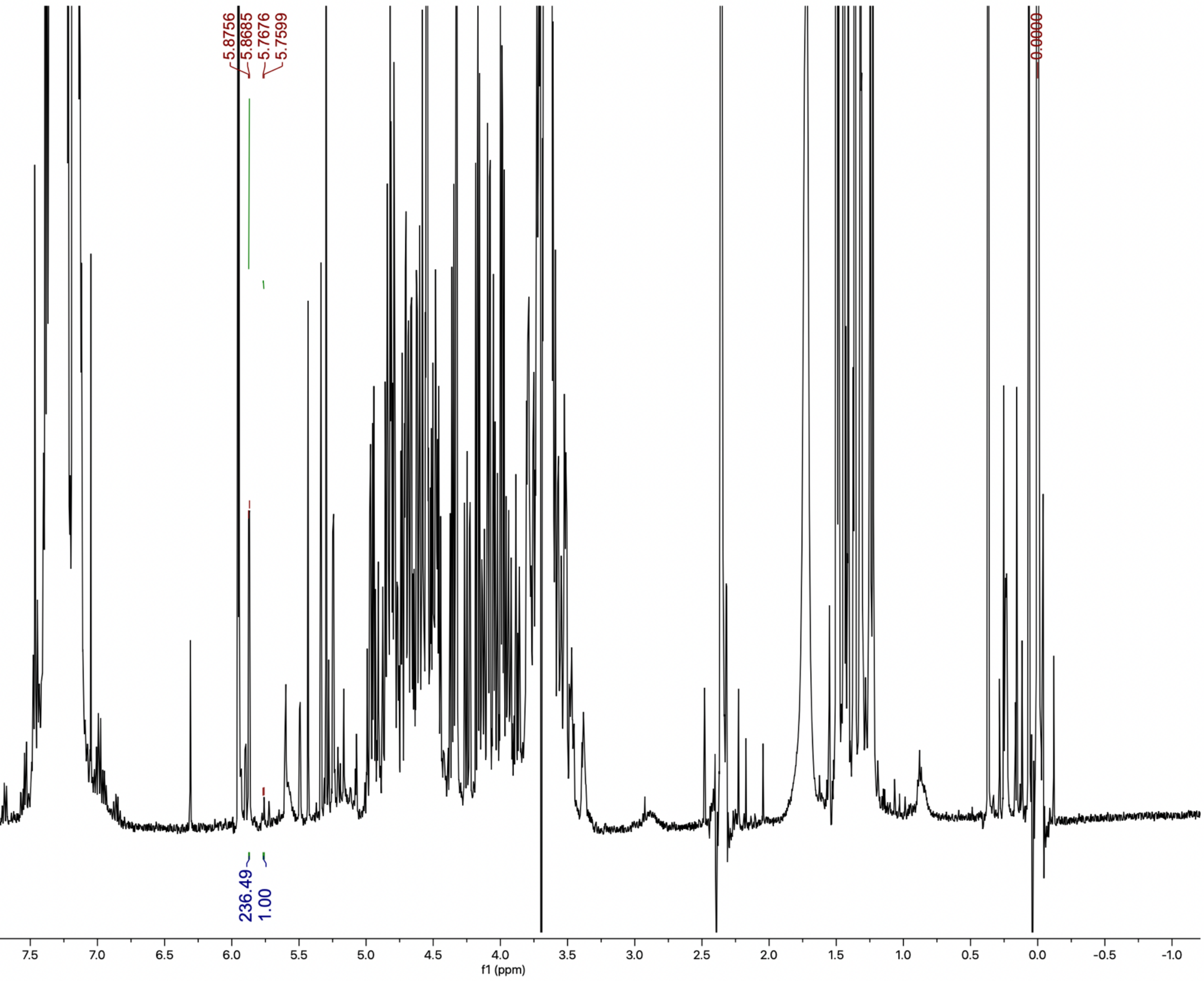

Scheme 4, Entry 1 (**13**, from **8d**), **crude**
^{1}H NMR, 400 MHz, $CDCl_3$ with 0.03% TMS

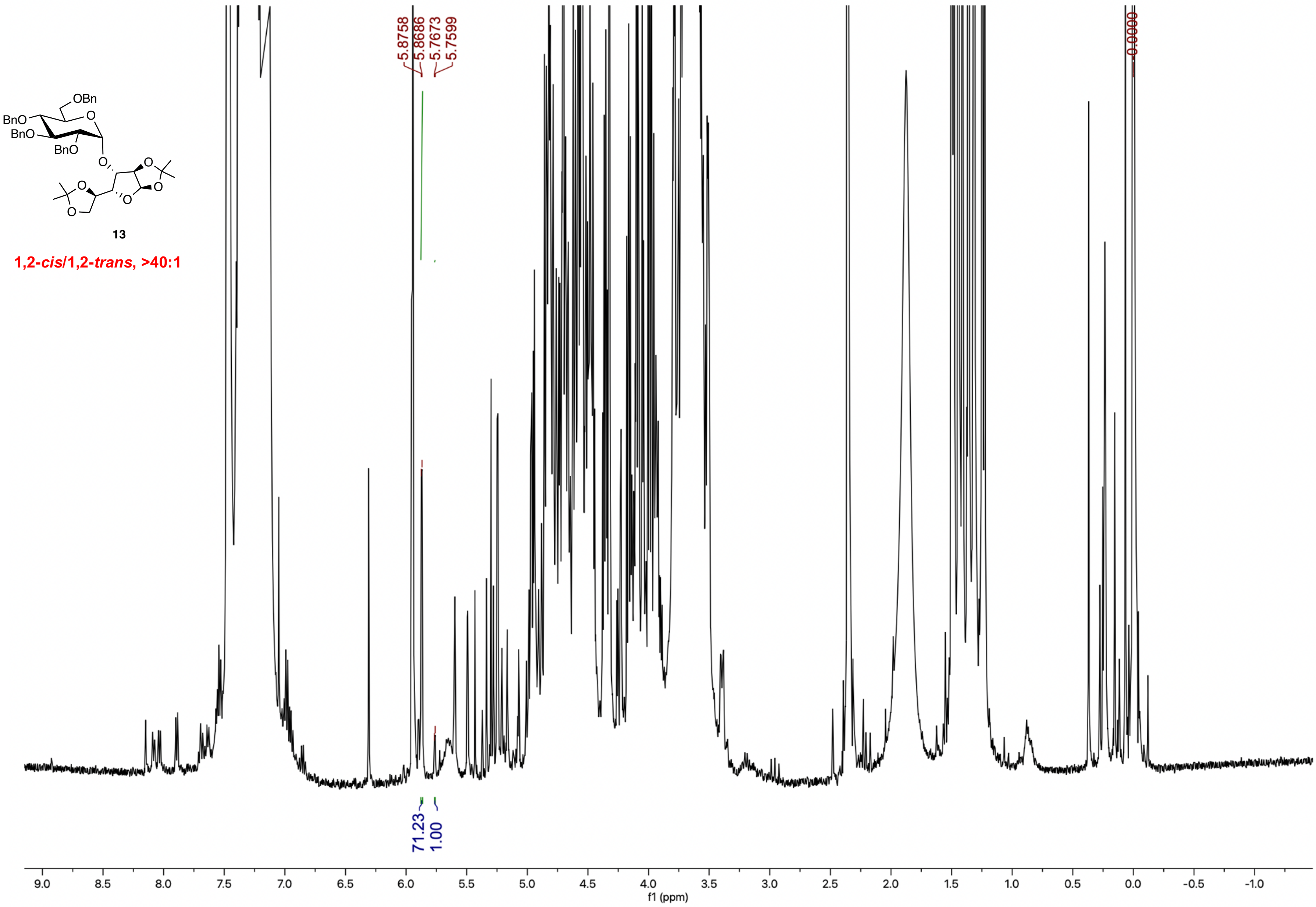

13

1,2-cis/1,2-trans, >40:1

Scheme 4, Entry 2 (**14**, from **8a**), **crude**

^{1}H NMR, 400 MHz, $CDCl_3$ with 0.03% TMS

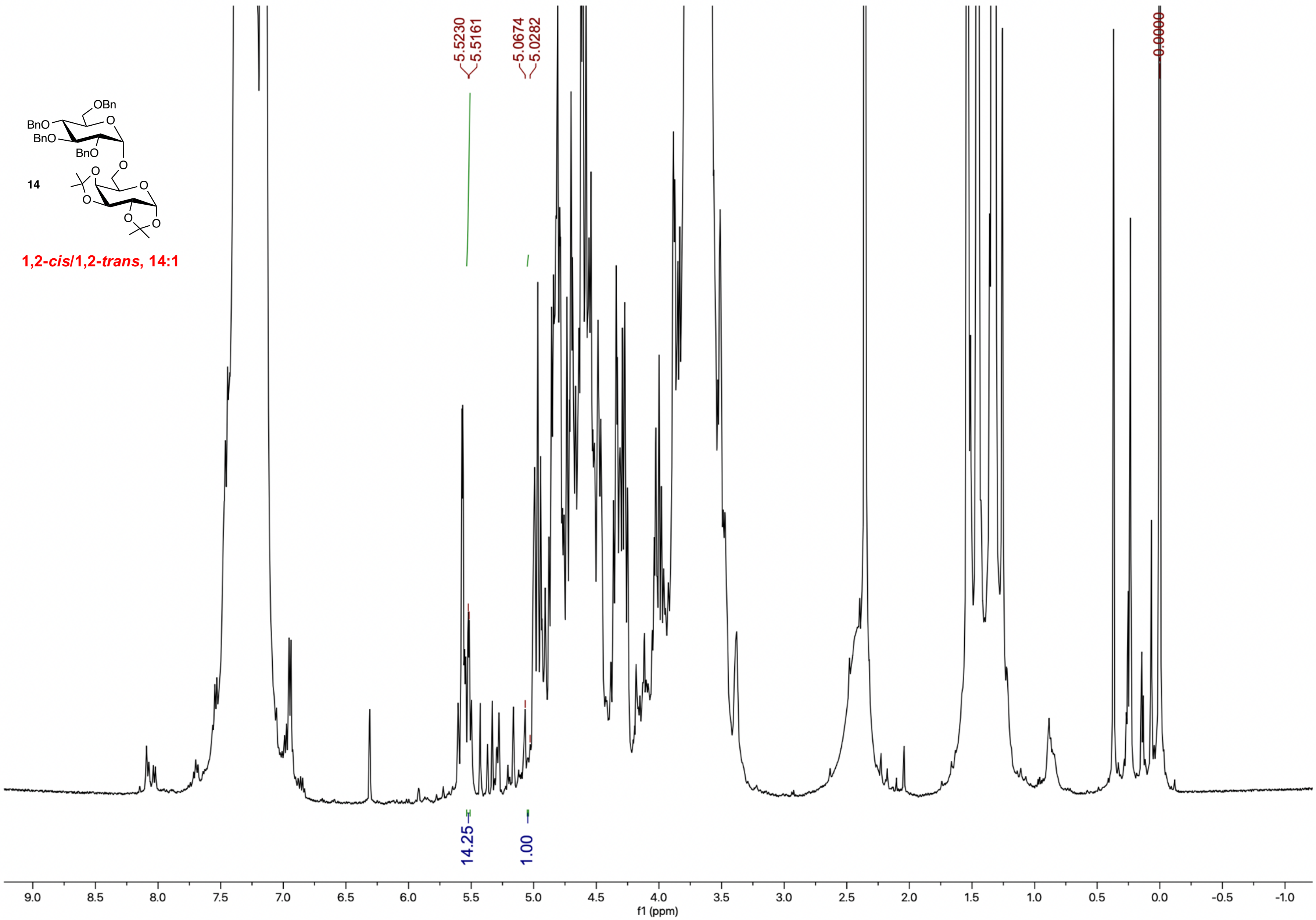

14

***1,2-cis/1,2-trans*, 14:1**

Scheme 4, Entry 2 (**14**, from **8d**), **crude**
^{1}H NMR, 400 MHz, $CDCl_3$ with 0.03% TMS

14

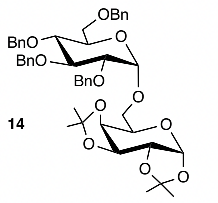

1,2-*cis*/1,2-*trans*, 24:1

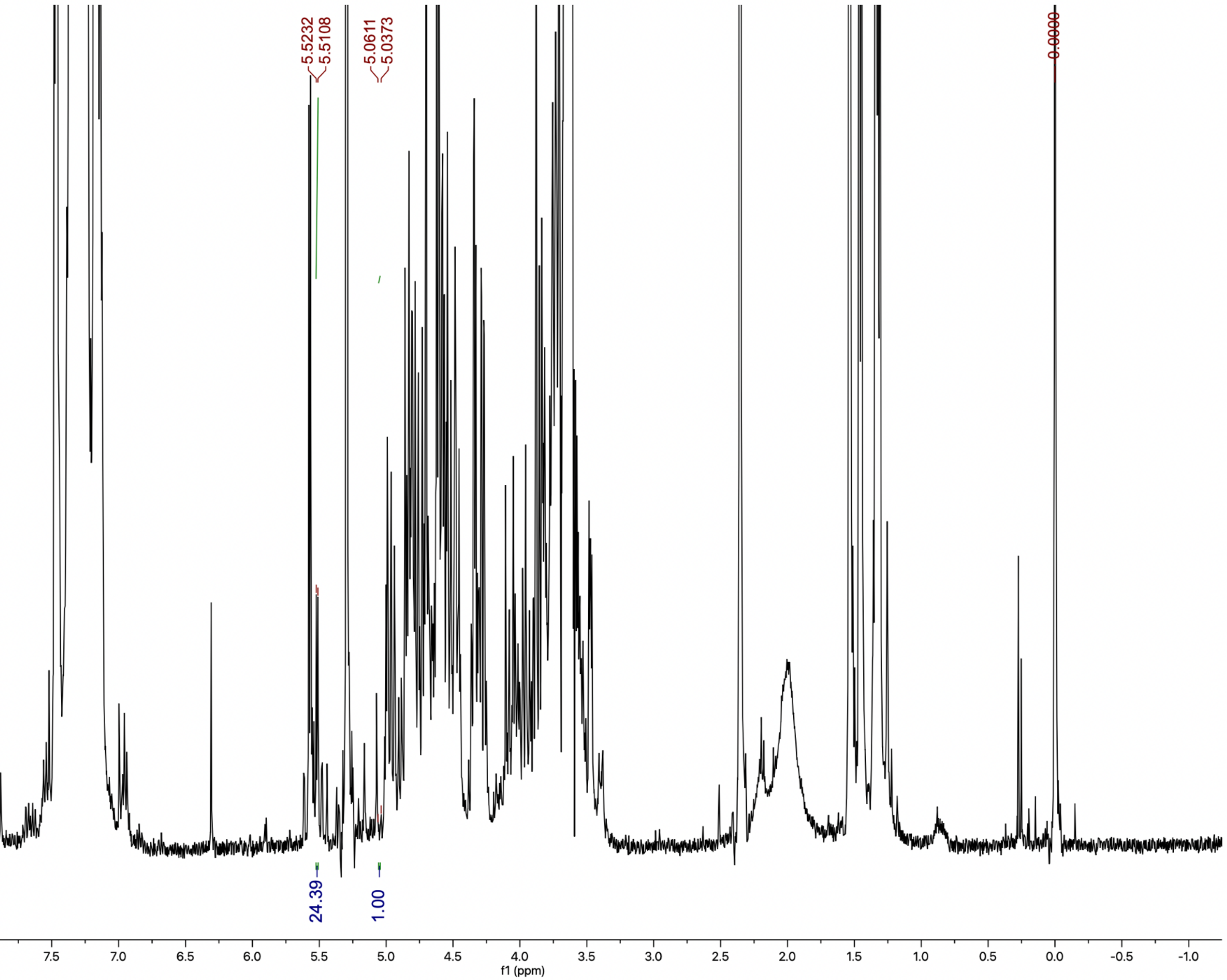

Scheme 4, Entry 3 (**15**, from **8a**), **crude**

^{1}H NMR, 400 MHz, $CDCl_3$ with 0.03% TMS

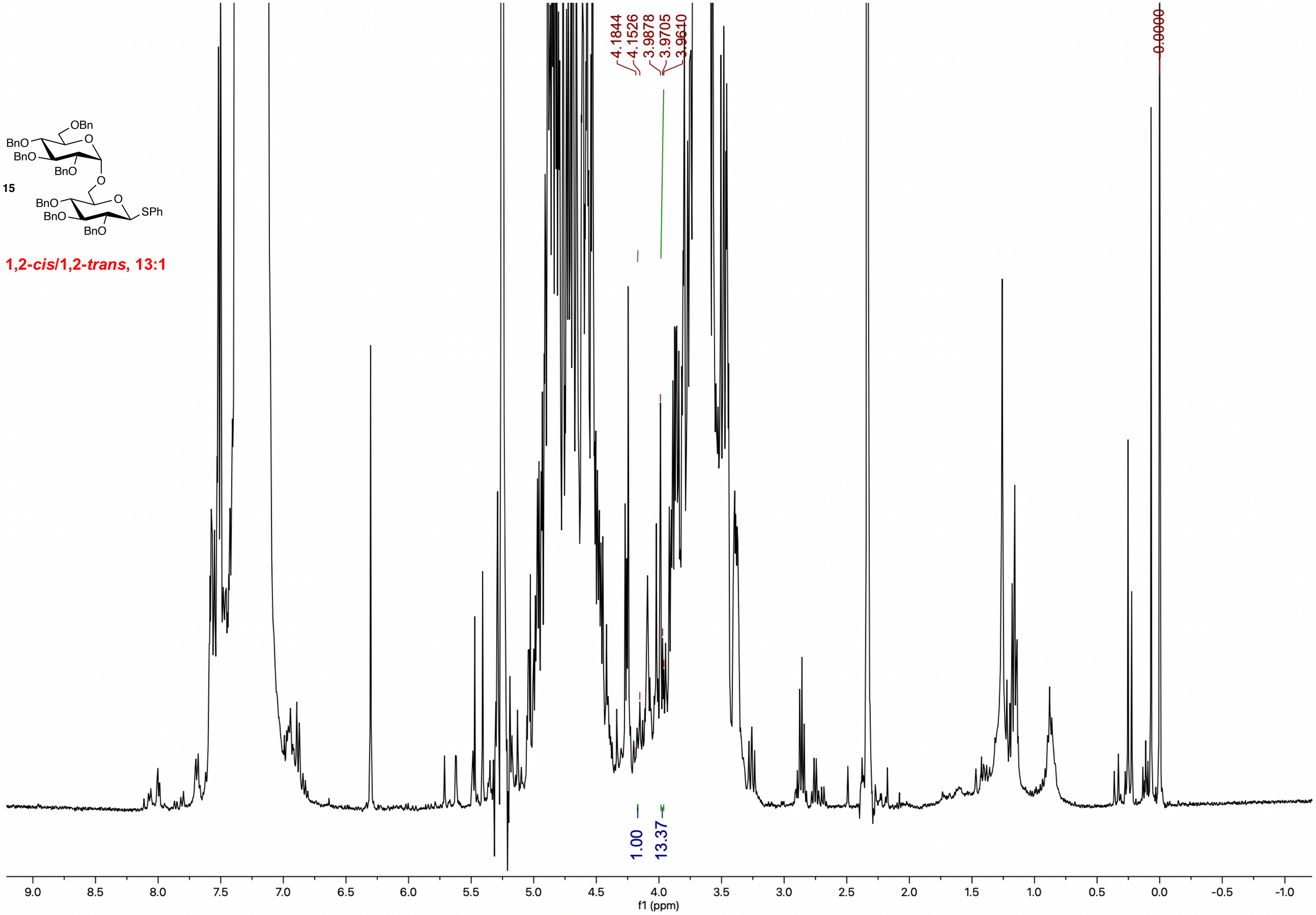

Scheme 4, Entry 3 (**15**, from **8d**), **crude**

^{1}H NMR, 400 MHz, $CDCl_3$ with 0.03% TMS

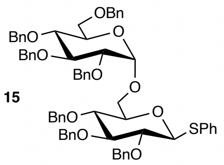

15

***1,2-cis/1,2-trans*, 34:1**

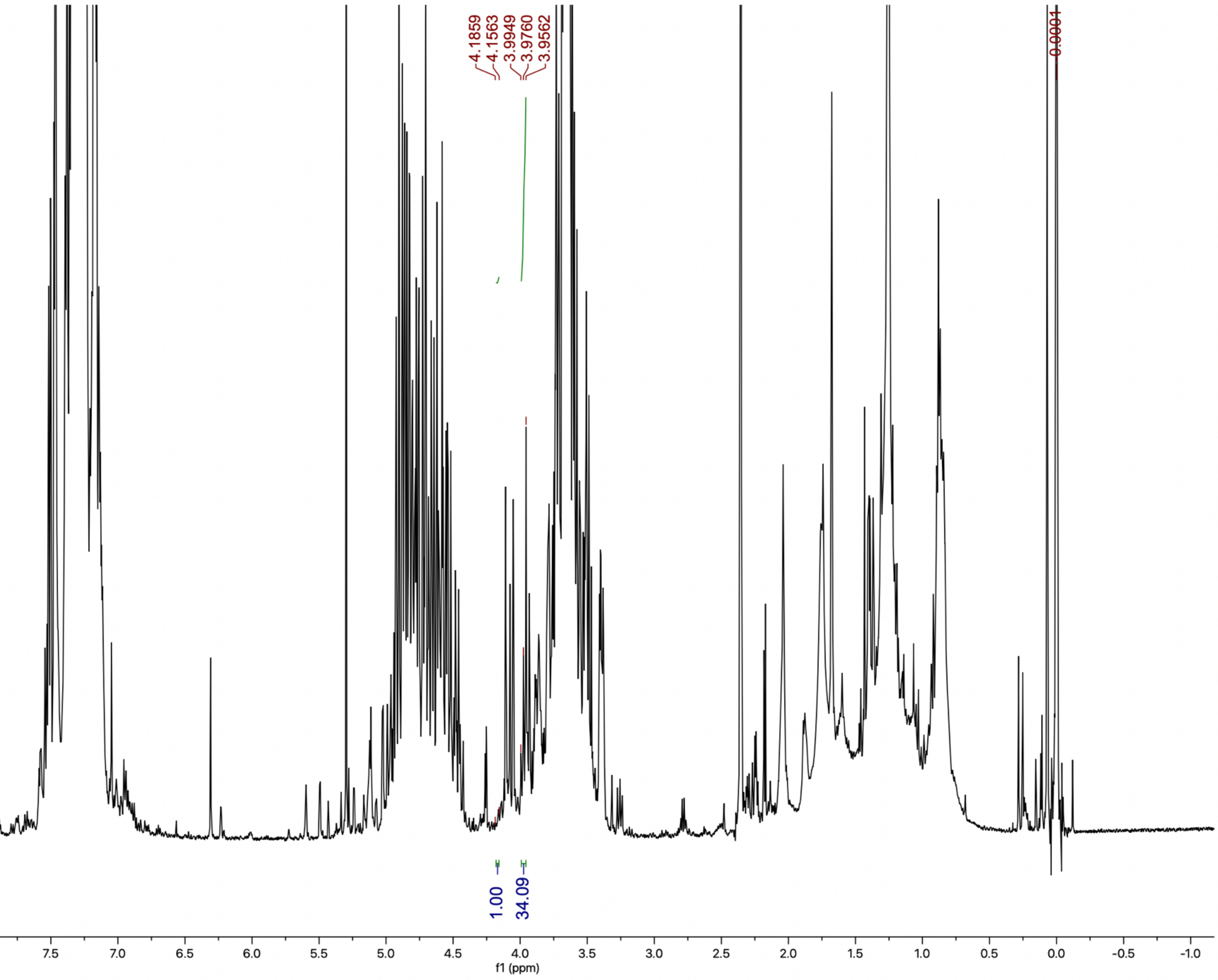

Scheme 4, Entry 4 (**16**, from **8a**), **crude**
^{1}H NMR, 400 MHz, $CDCl_3$ with 0.03% TMS

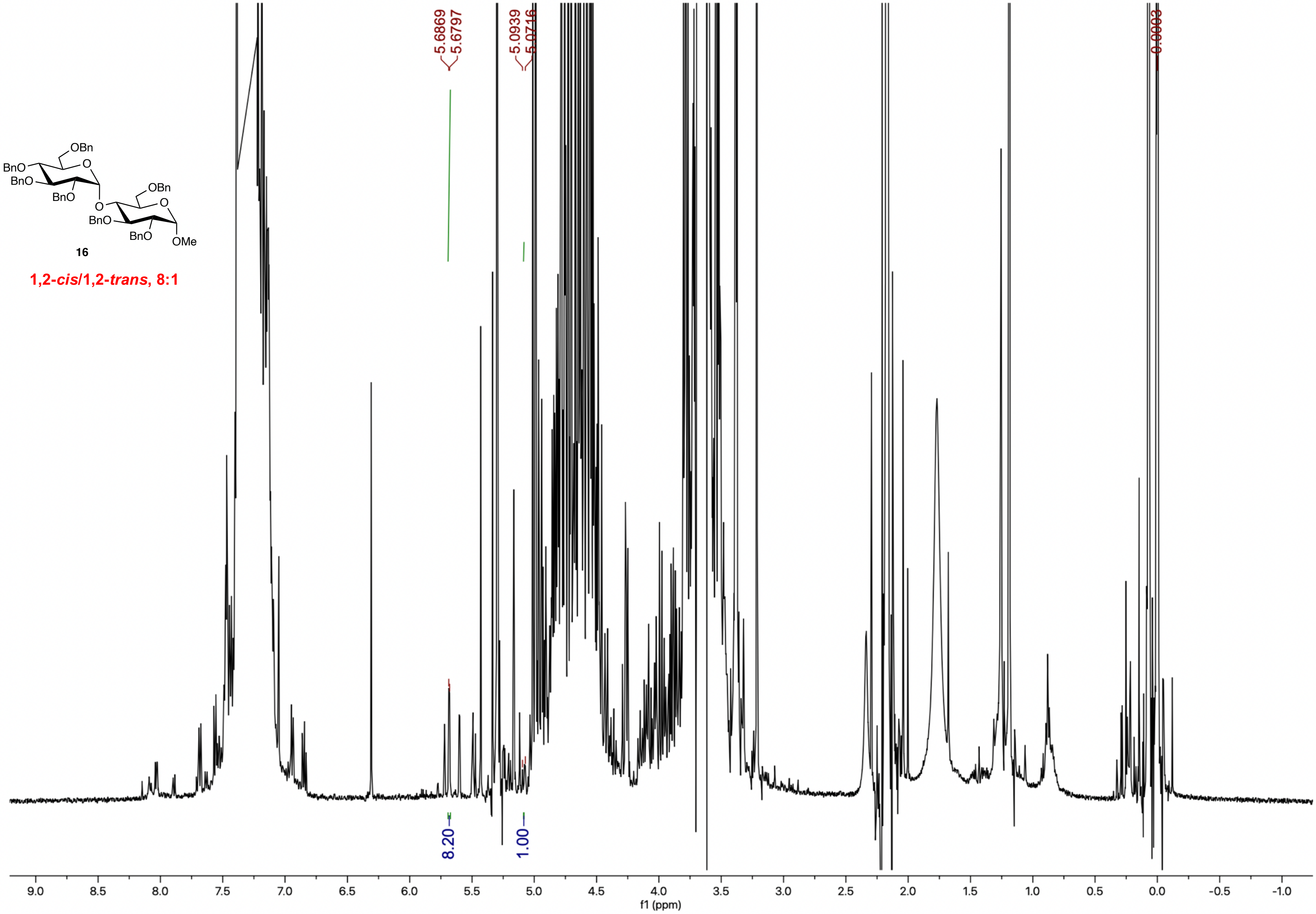

Scheme 4, Entry 4 (**16**, from **8d**), **crude**

^{1}H NMR, 400 MHz, $CDCl_3$ with 0.03% TMS

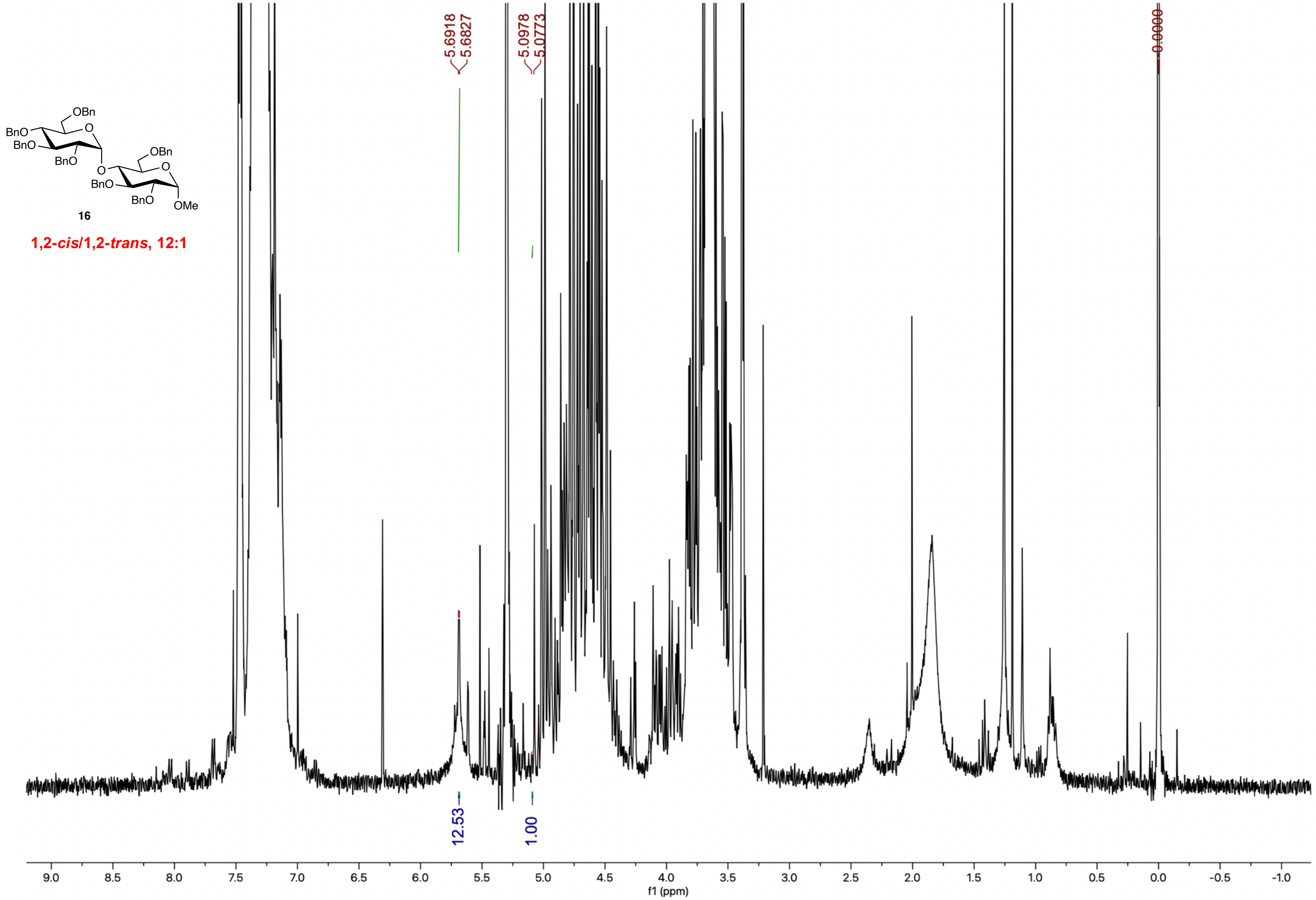

Scheme 4, Entry 5 (**17**, from **8a**), **crude**
^1H NMR, 400 MHz, $CDCl_3$ with 0.03% TMS

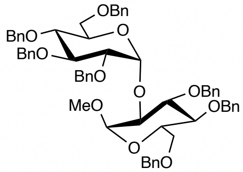

17

***1,2-cis/1,2-trans*, 21:1**

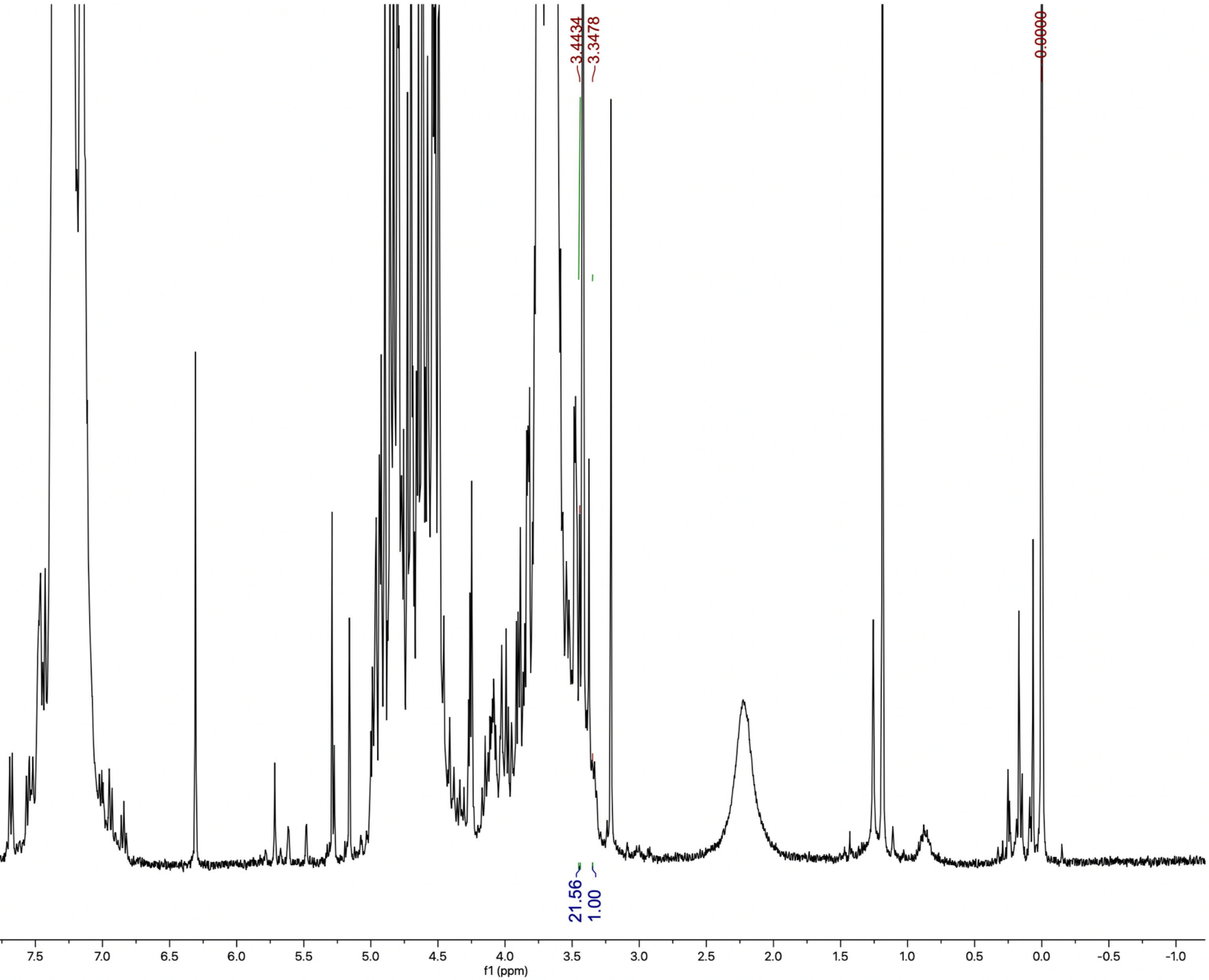

Scheme 4, Entry 5 (**17**, from **8d**), **crude**

^{1}H NMR, 400 MHz, $CDCl_3$ with 0.03% TMS

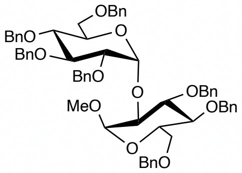

17

***1,2-cis/1,2-trans*, 38:1**

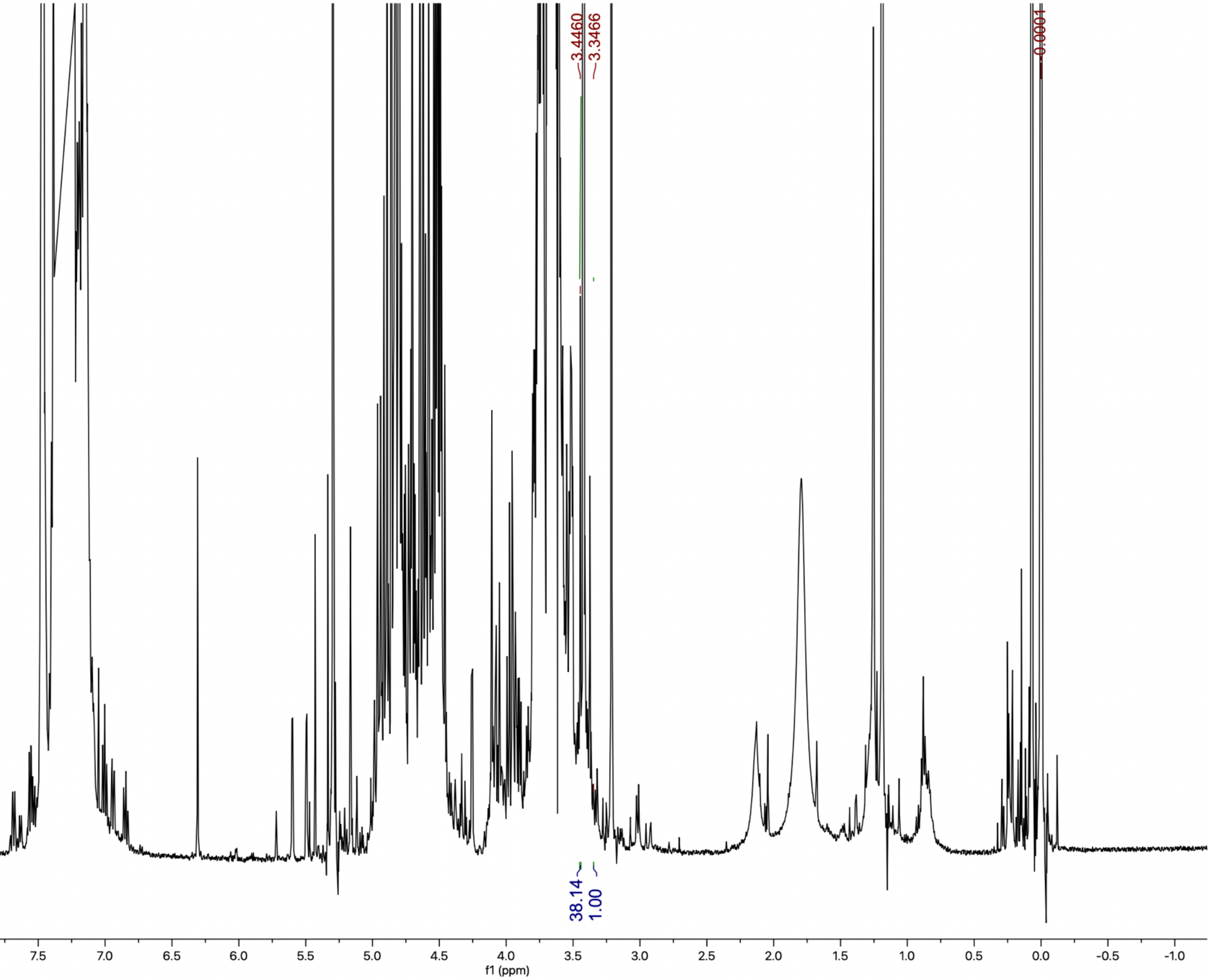

Scheme 4, Entry 6 (**18**, from **8a**), **crude**
^{1}H NMR, 400 MHz, $CDCl_3$ with 0.03% TMS

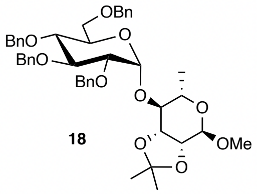

18

1,2-*cis*/1,2-*trans*, >40:1
(1,2-*trans* anomer not detected on crude or purified)

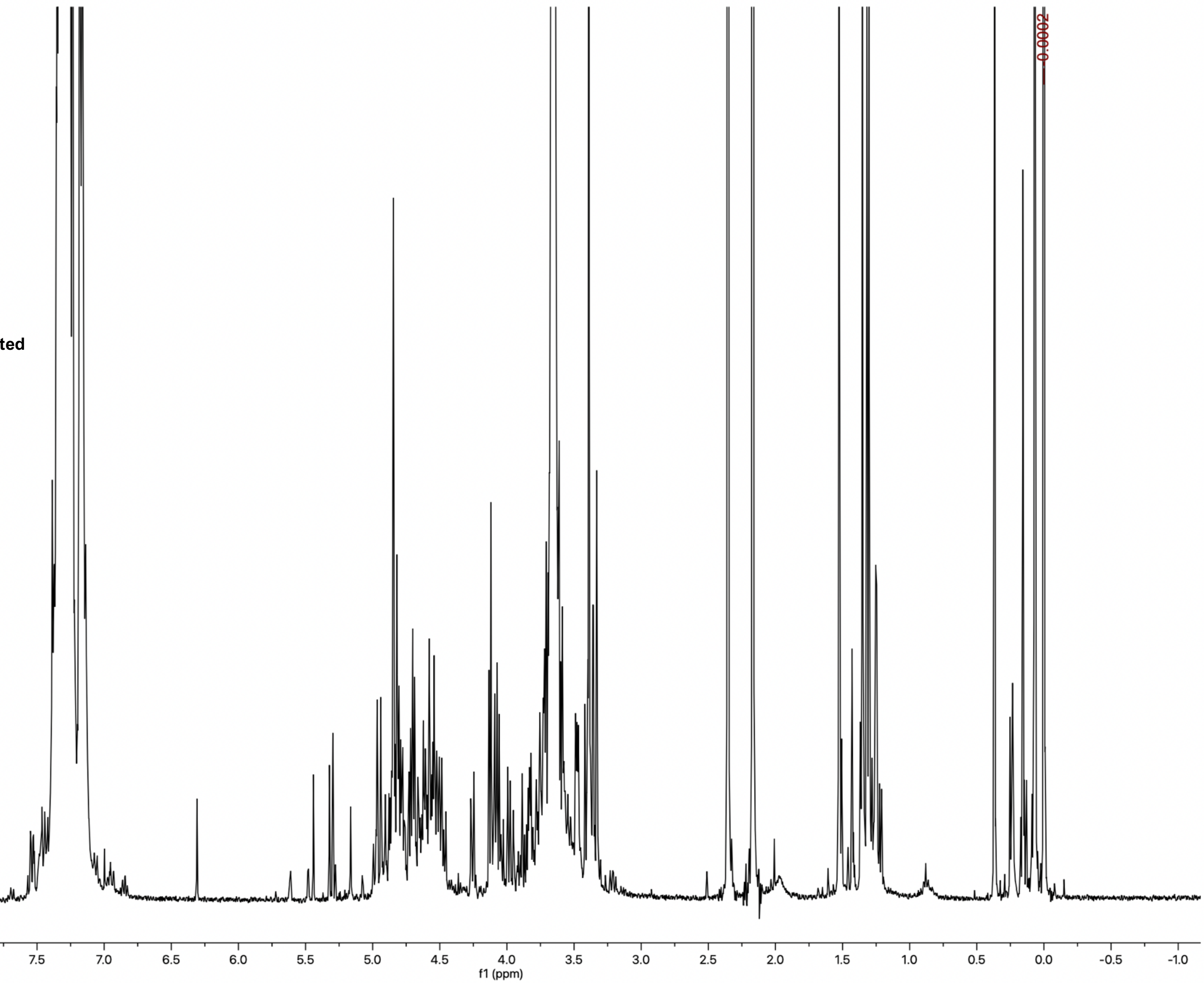

Scheme 4, Entry 6 (**18**, from **8d**), **crude**
^{1}H NMR, 400 MHz, $CDCl_3$ with 0.03% TMS

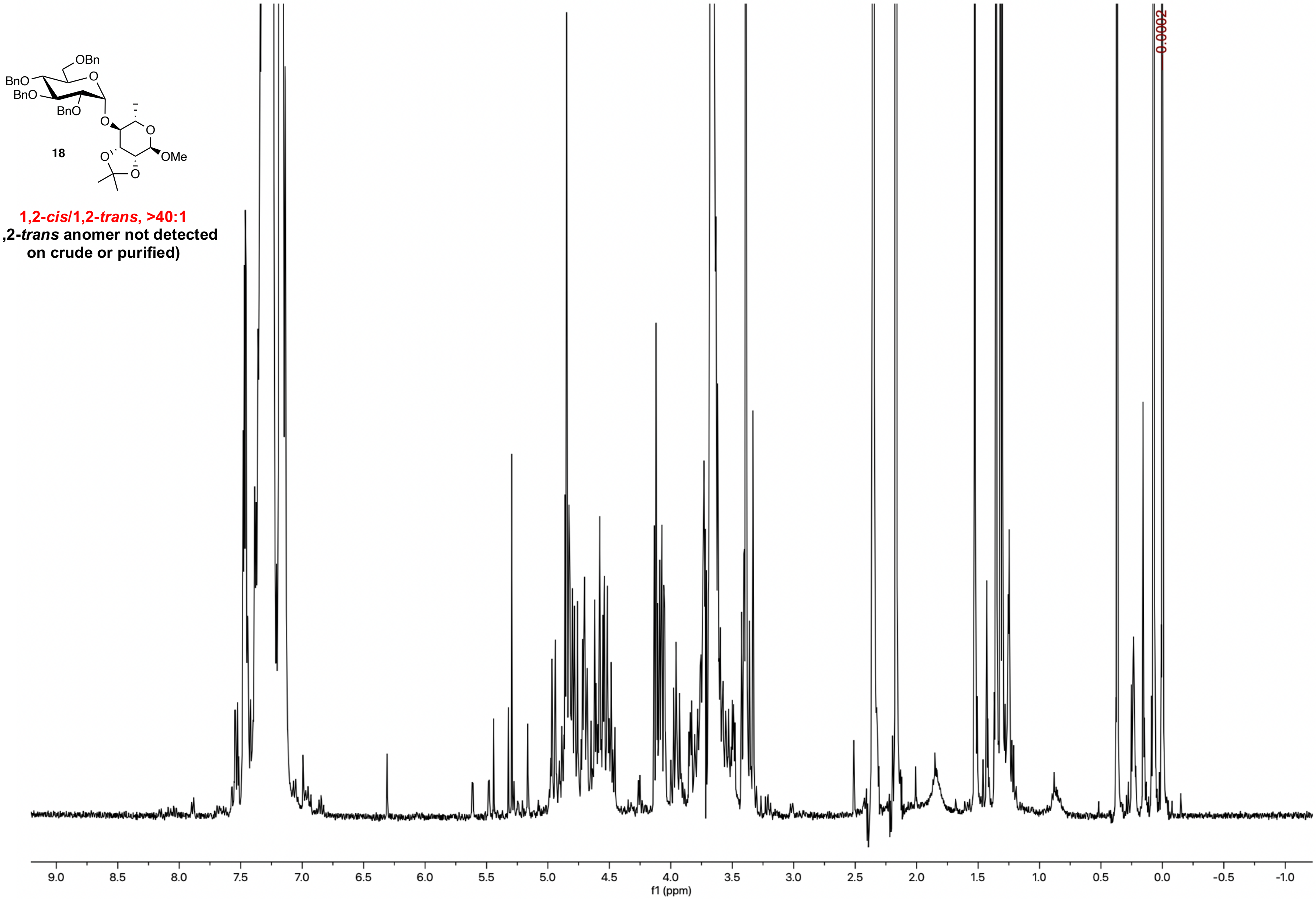

18

***1,2-cis/1,2-trans*, >40:1**
(1,2-*trans* anomer not detected on crude or purified)

Scheme 4, Entry 7 (**19**, from **8a**), **crude**

^{1}H NMR, 400 MHz, $CDCl_3$ with 0.03% TMS

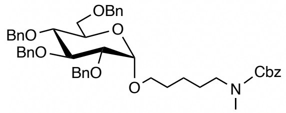

19

1,2-*cis*/1,2-*trans*, 17:1

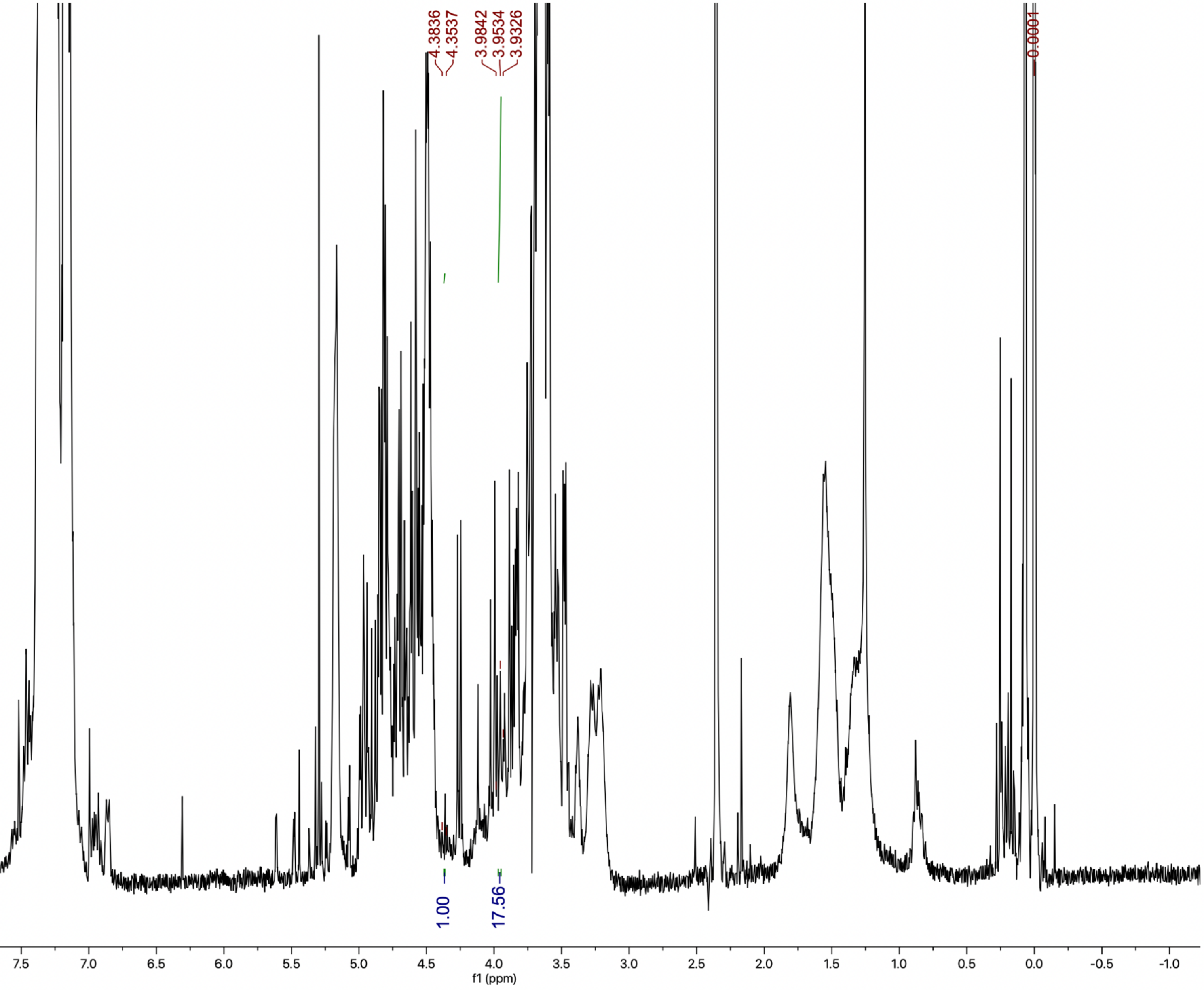

Scheme 4, Entry 7 (**19**, from **8d**), **crude**

^{1}H NMR, 400 MHz, $CDCl_3$ with 0.03% TMS

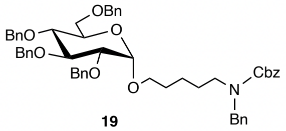

19

***1,2-cis/1,2-trans*, 30:1**

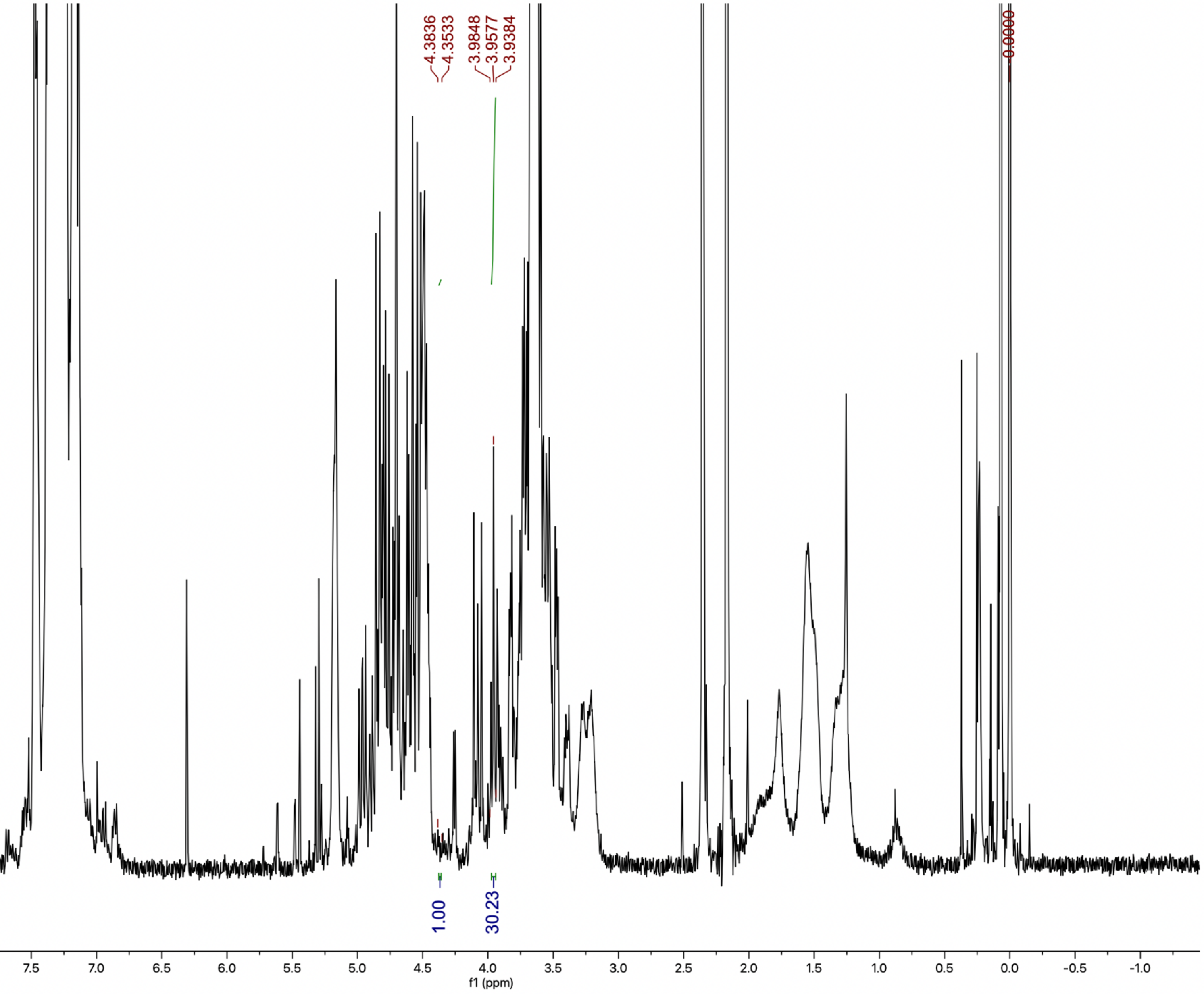

Scheme 4, Entry 8 (**20**, from **8e**), **crude**
^{1}H NMR, 400 MHz, $CDCl_3$ with 0.03% TMS

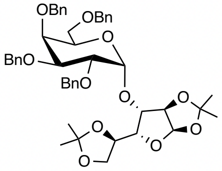

20

1,2-cis/1,2-trans, >40:1

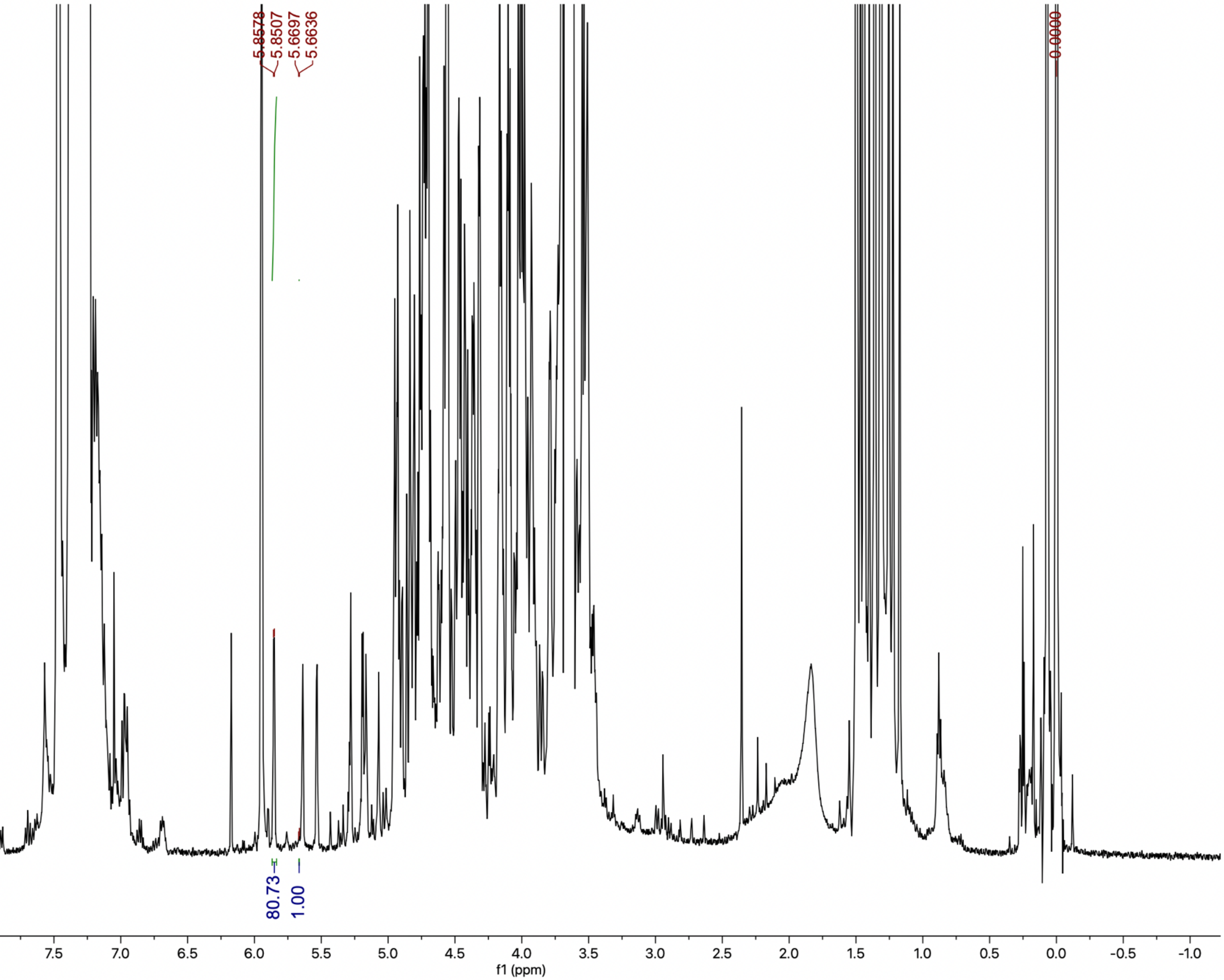

Scheme 4, Entry 9 (21, from 8e), crude

^{1}H NMR, 400 MHz, $CDCl_3$ with 0.03% TMS

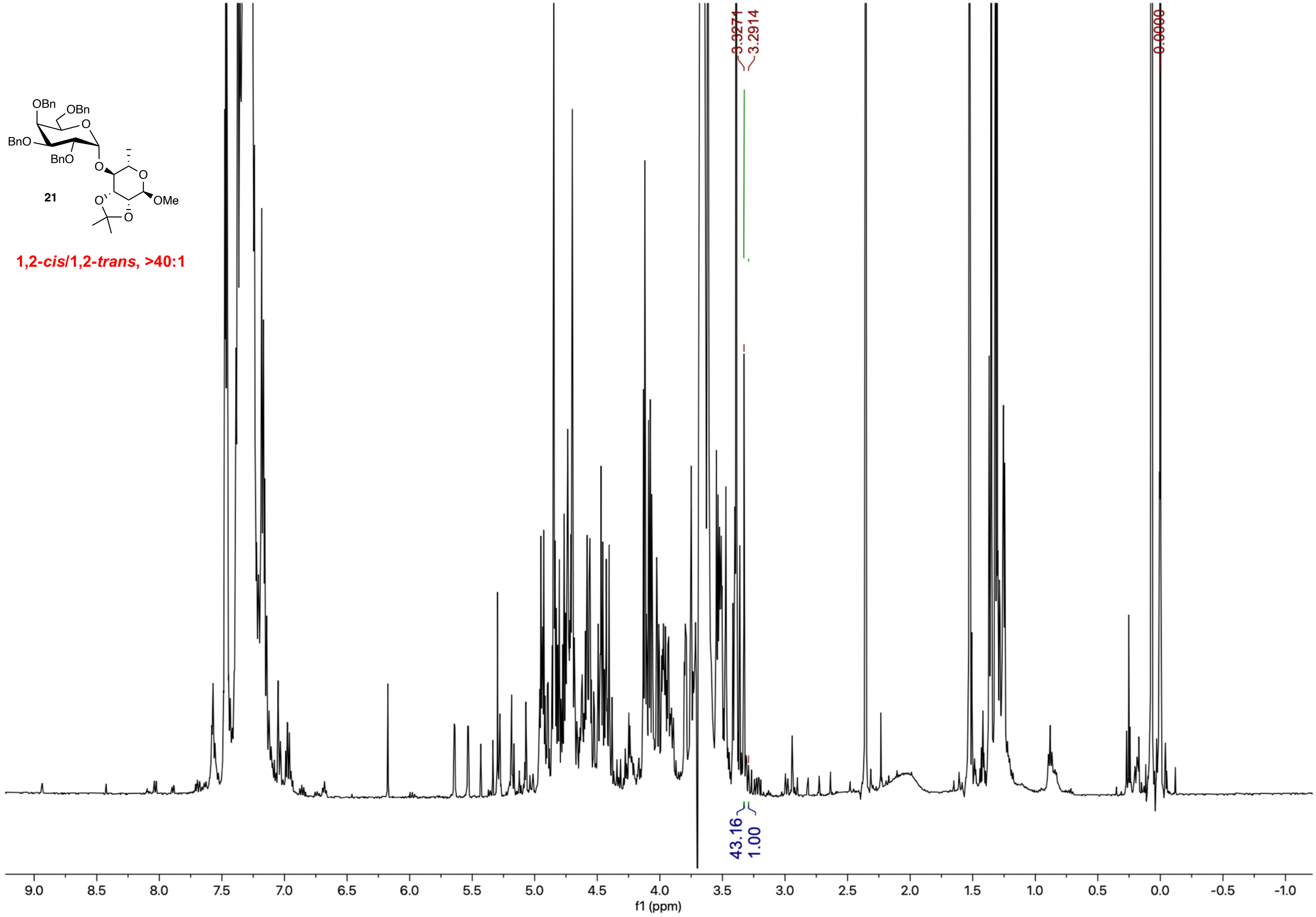

21

1,2-*cis*/1,2-*trans*, >40:1

Scheme 4, Entry 10 (**22**, from **8f**), **crude**
^{1}H NMR, 400 MHz, $CDCl_3$ with 0.03% TMS

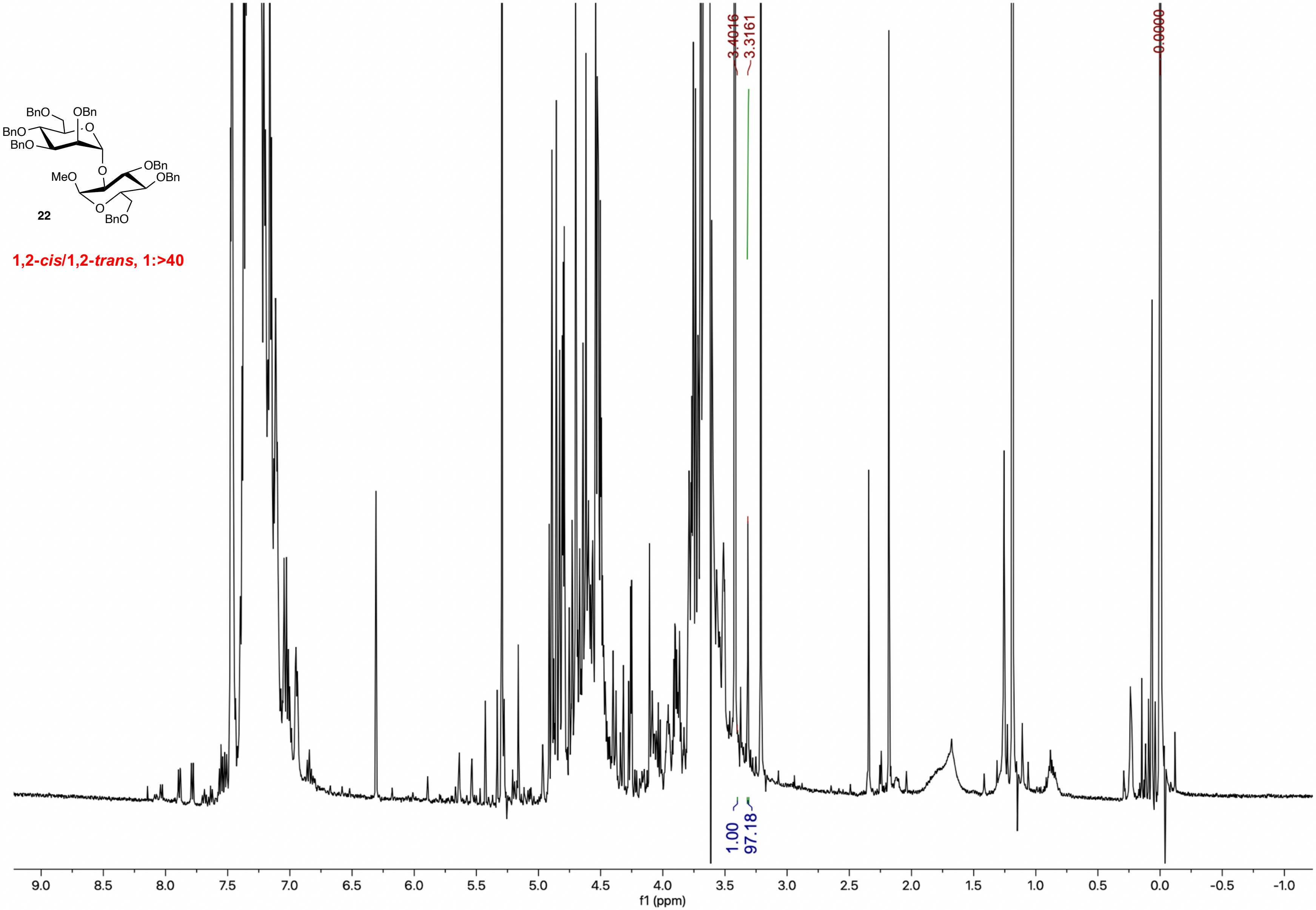

Scheme 4, Entry 11 (**23**, from **8f**), **crude**

^{1}H NMR, 400 MHz, $CDCl_3$ with 0.03% TMS

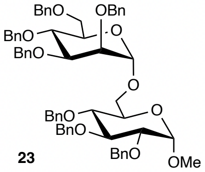

23

***1,2-cis/1,2-trans*, 1:>40**

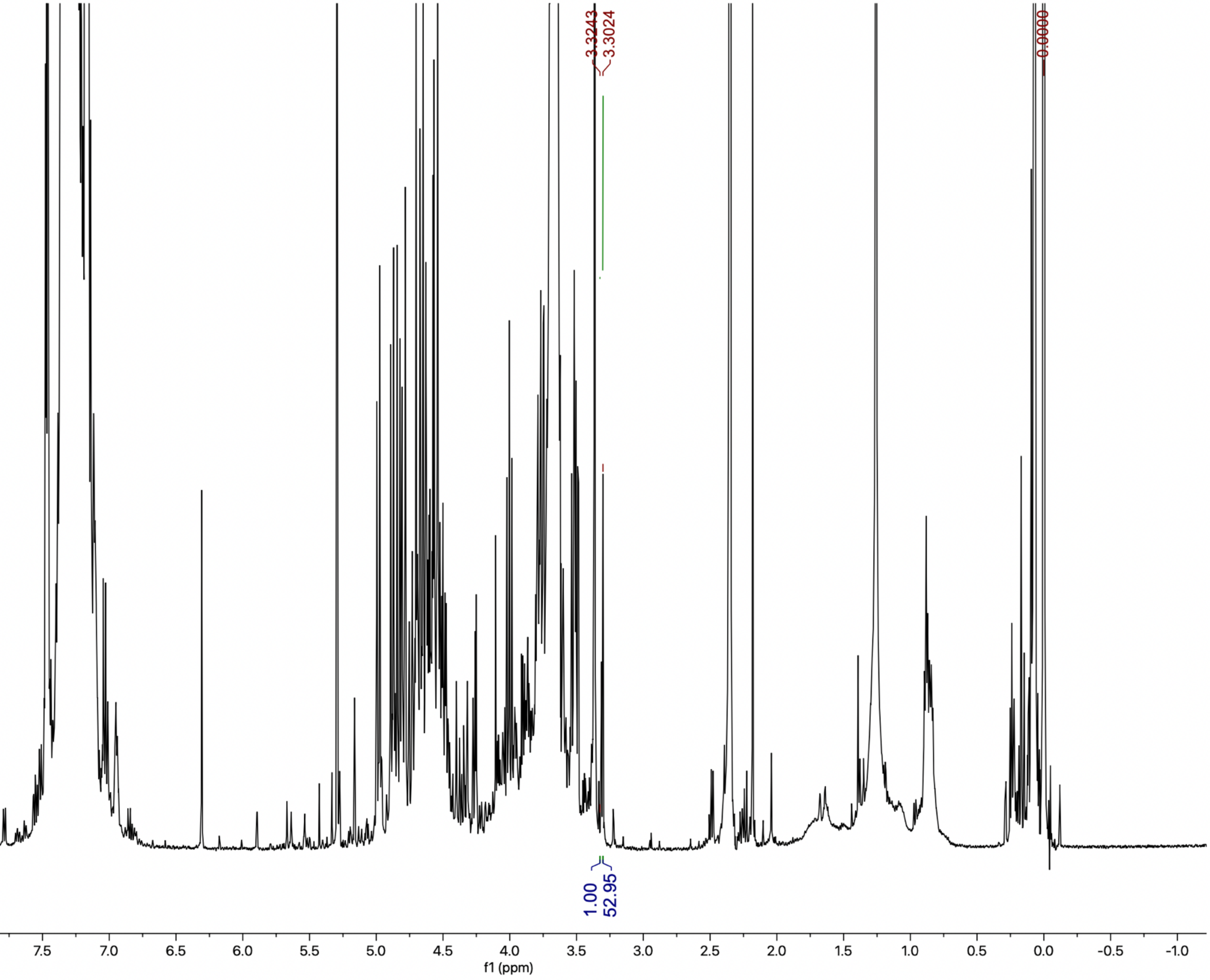

Scheme 4, Entry 12 (24, from 8f), crude
^{1}H NMR, 400 MHz, $CDCl_3$ with 0.03% TMS

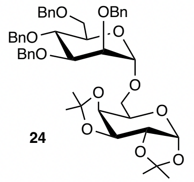

24

1,2-*cis*/1,2-*trans*, 1:>40
(1,2-*cis* anomer not detected on crude or purified)

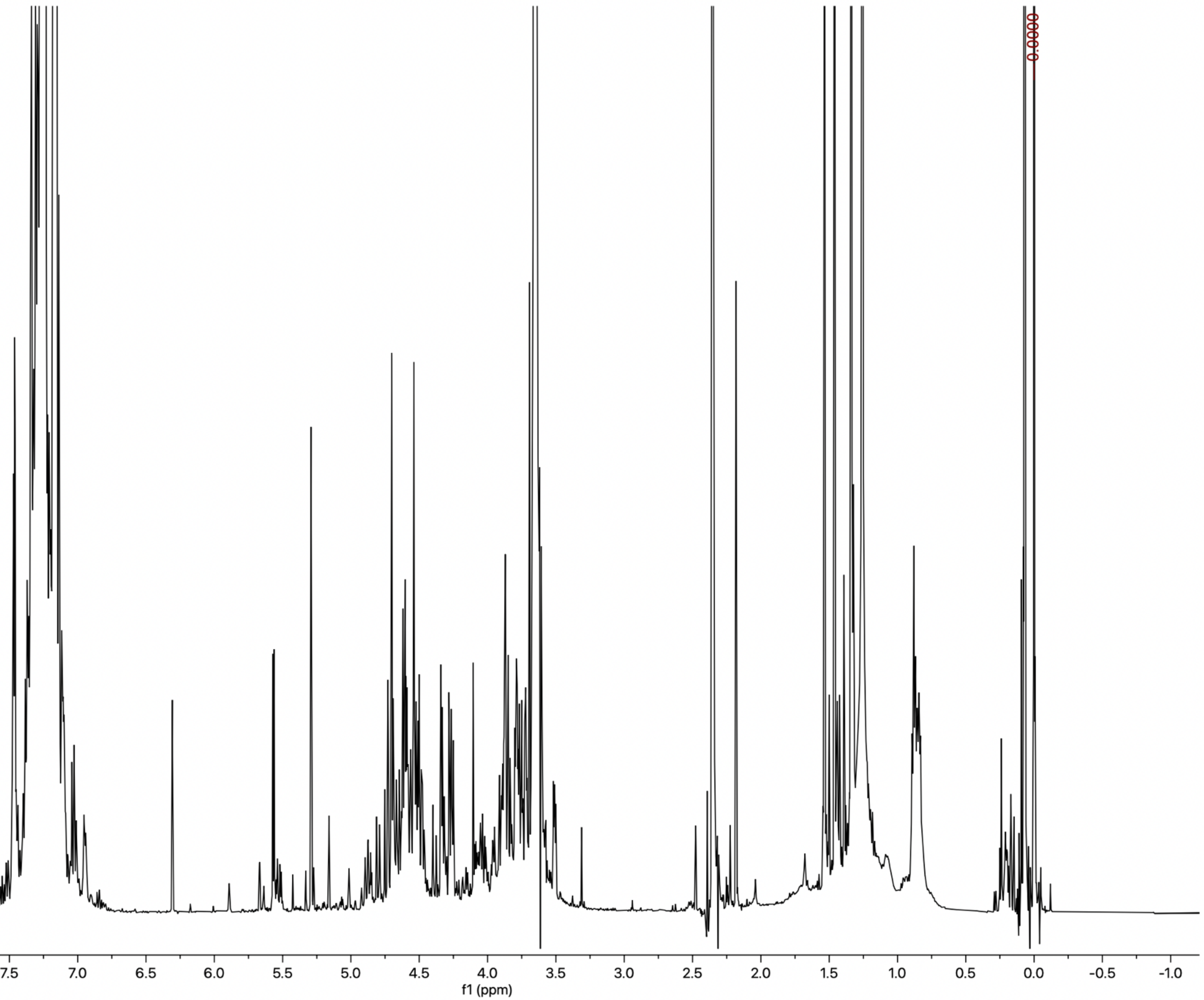

Scheme 4, Entry 13 (**α-25**, from **β-8g**), **crude**

^{1}H NMR, 400 MHz, $CDCl_3$ with 0.03% TMS

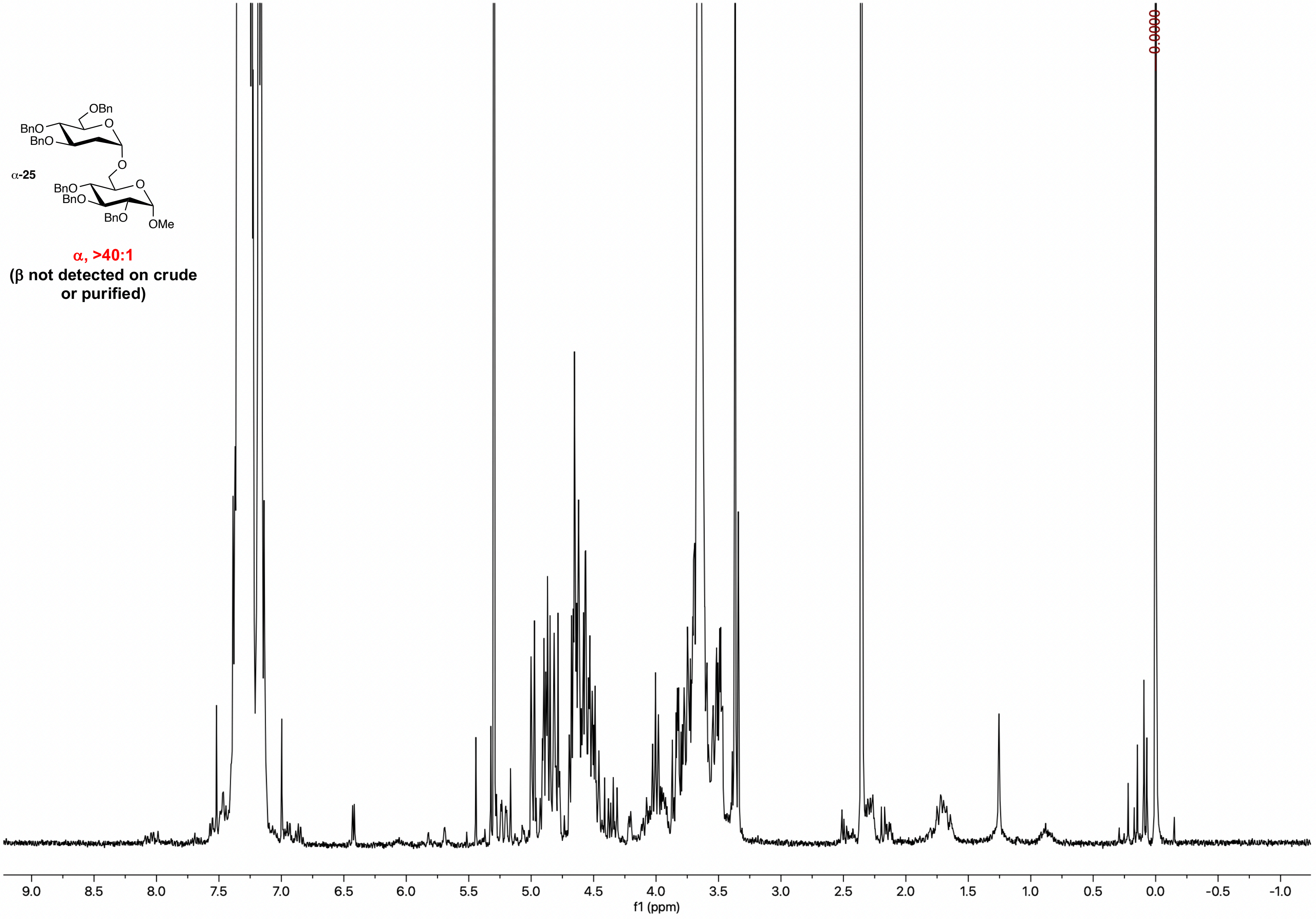

Scheme 4, Entry 14, (**β-25**, from **α-8g**), **crude**

^{1}H NMR, 400 MHz, $CDCl_3$ with 0.03% TMS

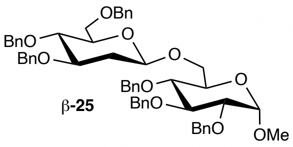

β-**25**

β, >40:1

(α not detected on crude or purified)

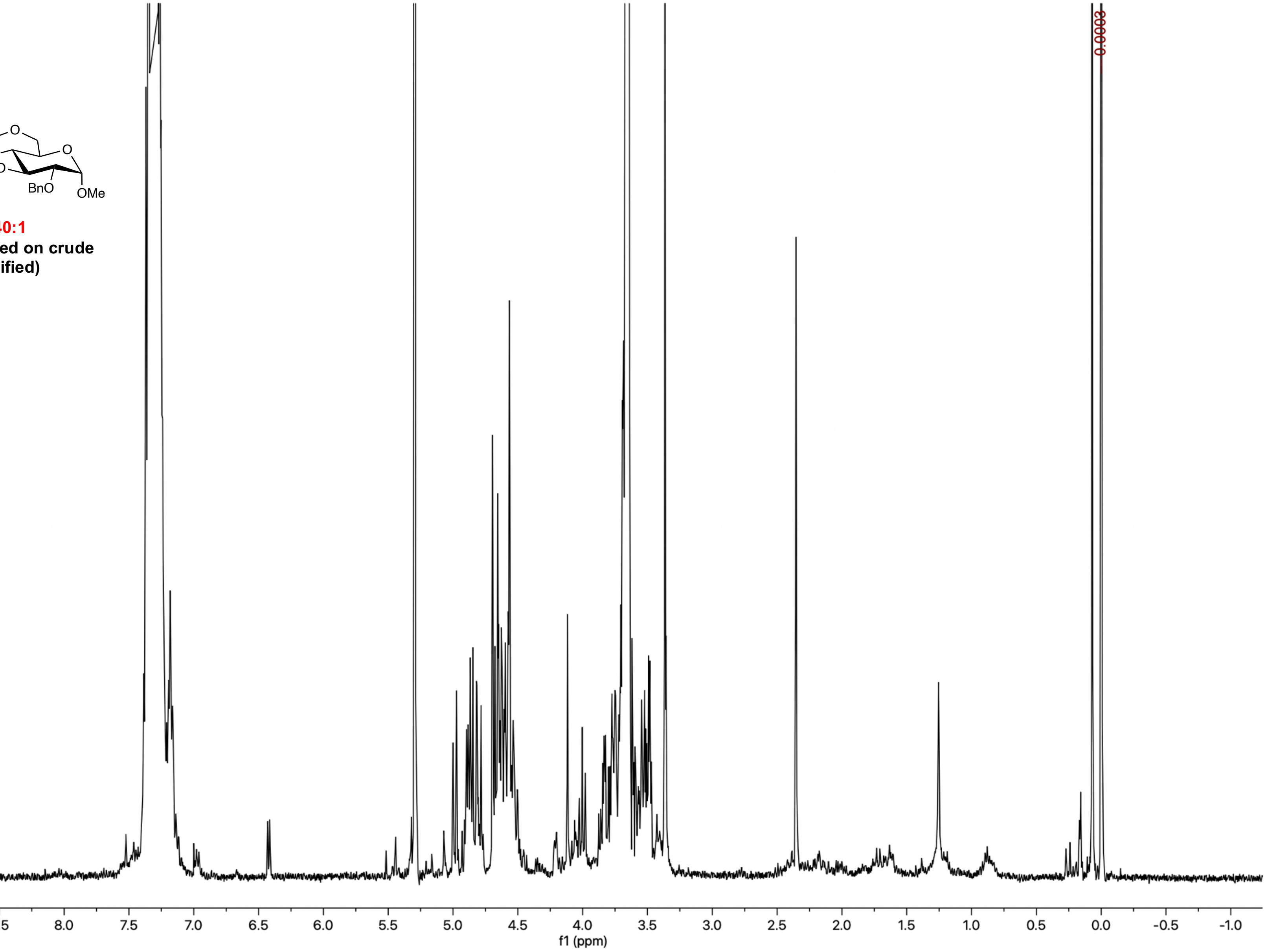

Scheme 4, Entry 15 (α**-26**, from β**-8g**), **crude**
^{1}H NMR, 400 MHz, $CDCl_3$ with 0.03% TMS

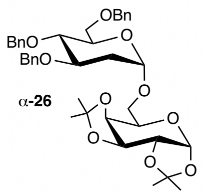

α, >40:1
(β not detected on crude or purified)

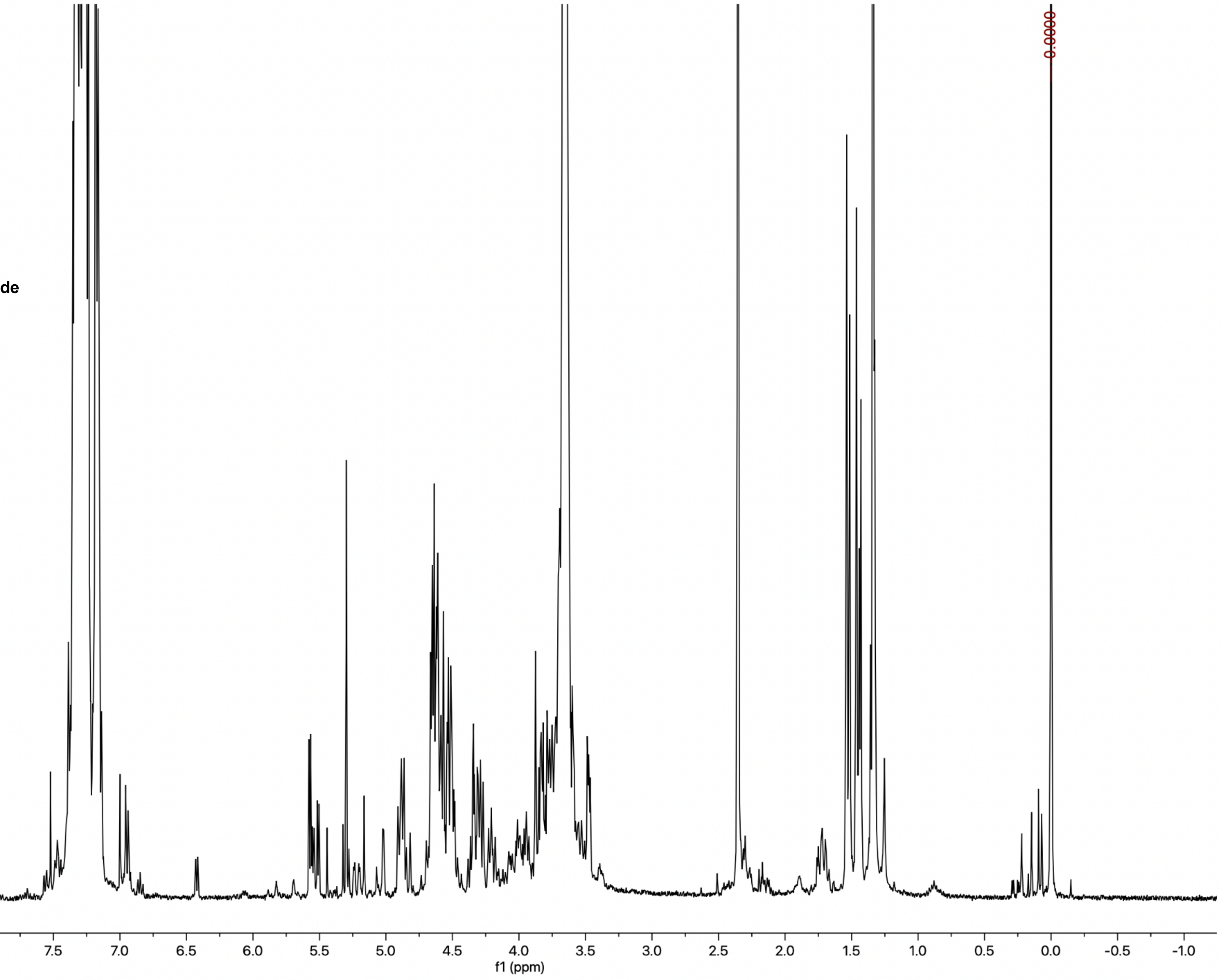

Scheme 4, Entry 16 (**β-26**, from **α-8g**), **crude**
^{1}H NMR, 400 MHz, $CDCl_3$ with 0.03% TMS

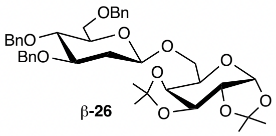

β-**26**

α:β, 1:11

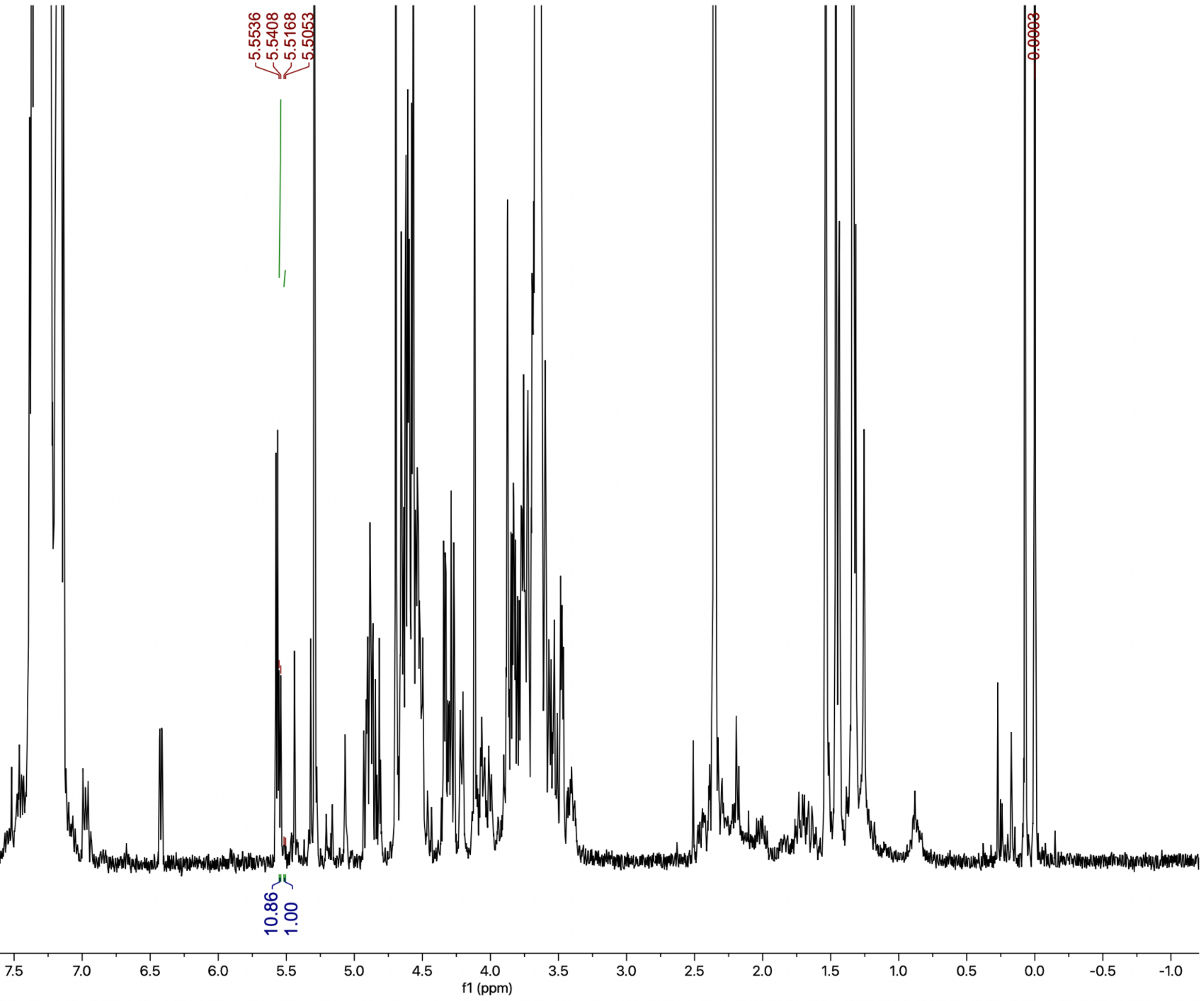

Scheme 4, Entry 17 (**27**, from **8h**), **crude**
^{1}H NMR, 400 MHz, $CDCl_3$ with 0.03% TMS

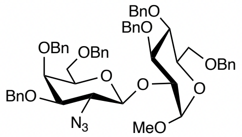

27

***1,2-cis/1,2-trans*, 1:9**

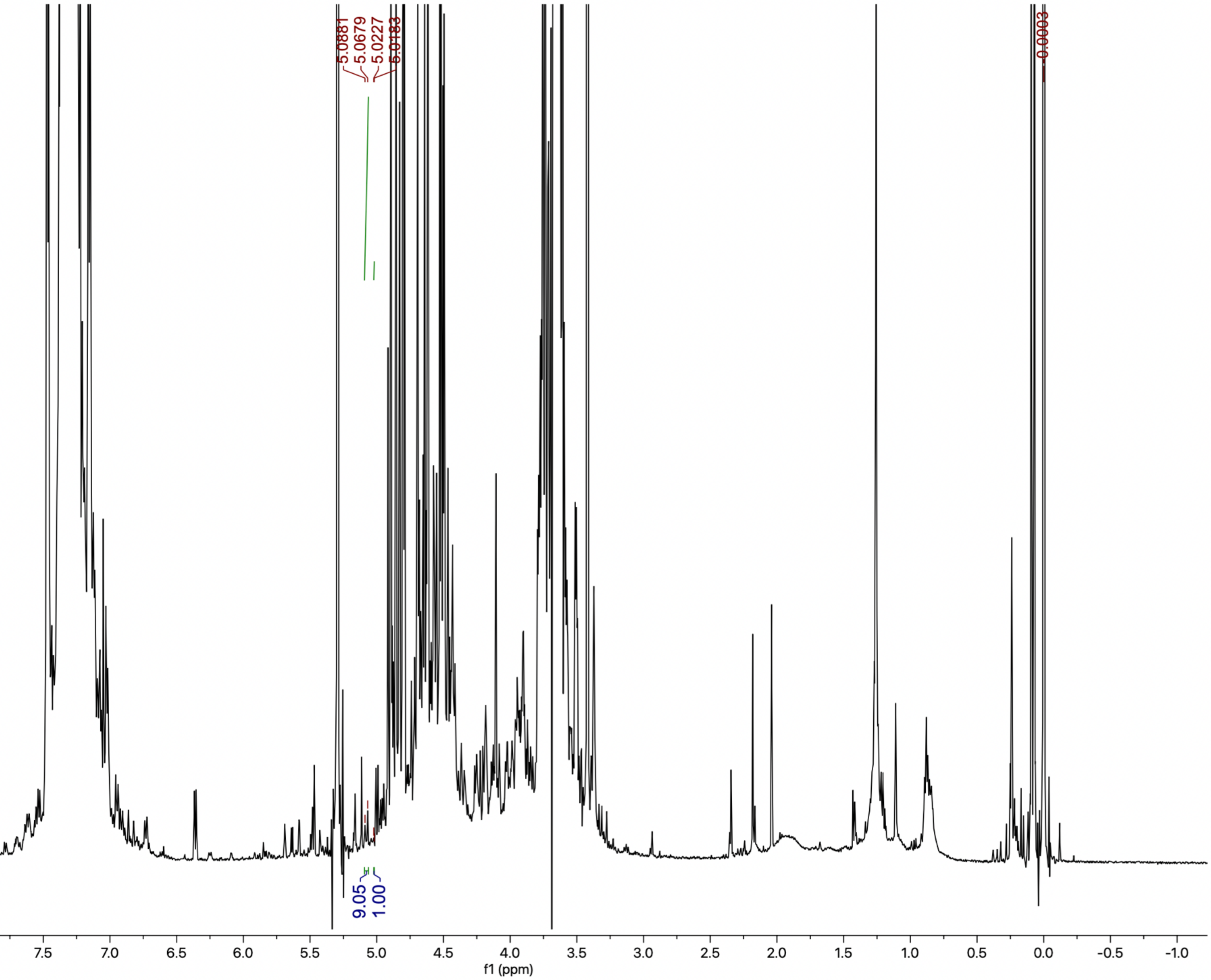

Scheme 4, Entry 18 (**28**, from **8h**), crude
^{1}H NMR, 400 MHz, $CDCl_3$ with 0.03% TMS

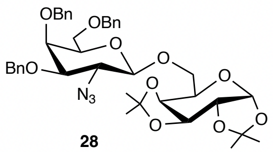

28

1,2-*cis*/1,2-*trans*, 1:>40
(1,2-*trans* anomer not detected on crude or purified)

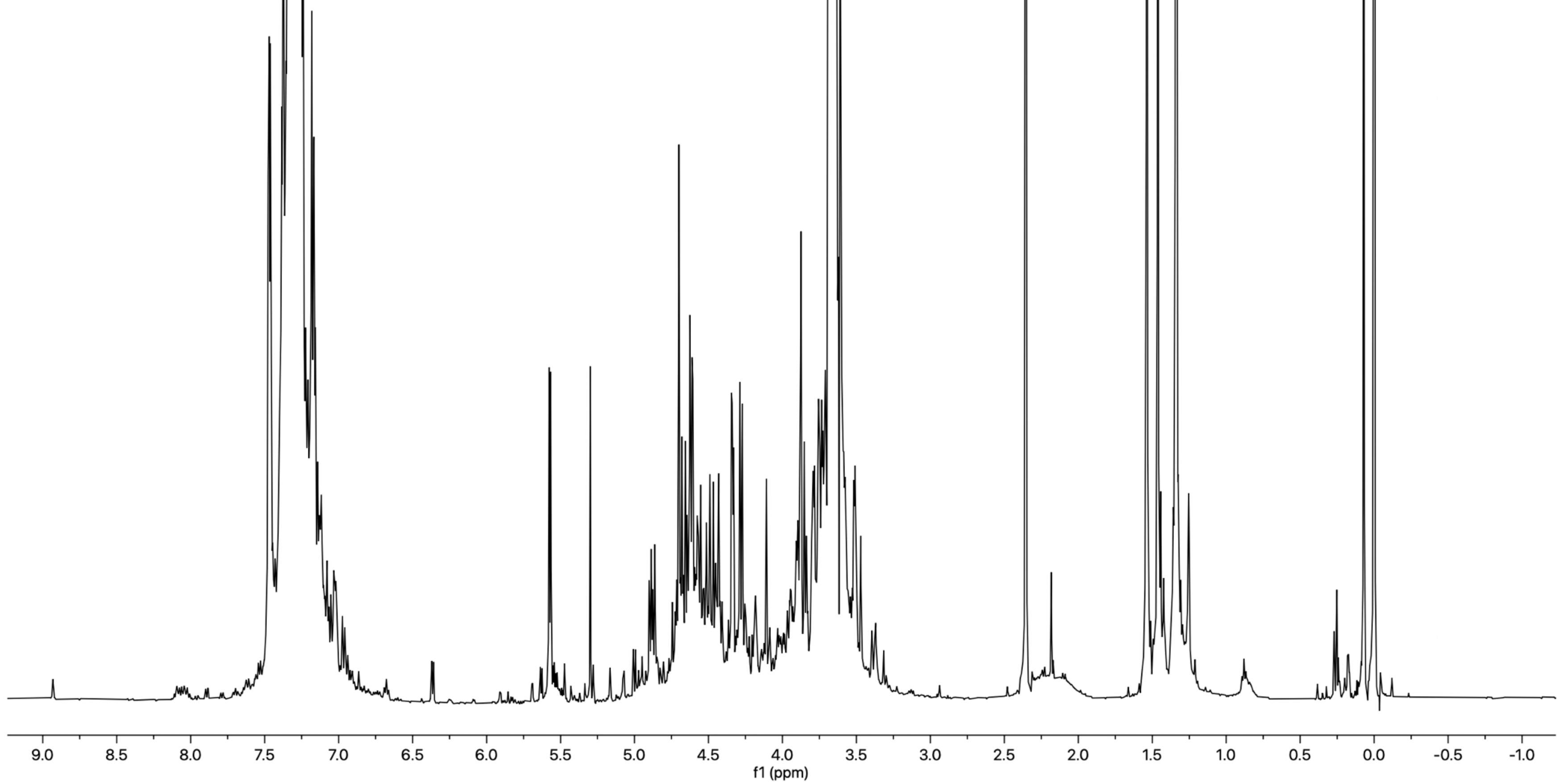

Supplement: Supplementary file 1 — ol3c03502_si_001.pdf [file ol3c03502_si_001.pdf]
